# Targeting PI4KA sensitizes refractory leukemia to chemotherapy by modulating the ERK/AMPK/OXPHOS axis

**DOI:** 10.7150/thno.76563

**Published:** 2022-10-03

**Authors:** Xiuxing Jiang, Xiangtao Huang, Guoxun Zheng, Guanfei Jia, Zhiqiang Li, Xin Ding, Ling Lei, Liang Yuan, Shuangnian Xu, Ning Gao

**Affiliations:** 1 College of Pharmacy, Army Medical University, 30 Gaotanyan Street, Shapingba District, Chongqing 400038, China.; 2 Department of Hematology, Southwest Hospital, Army Medical University, Chongqing 400038, China.; 3 Shanghai StoneWise AI Technology Co. Ltd. Shanghai 201210, China.; 4 Key Laboratory of Basic Pharmacology of Ministry of Education, Joint International Research Laboratory of Ethnomedicine of Ministry of Education, Zunyi Medical University, Zunyi, Guizhou 563006, China.

**Keywords:** Leukemia, chemoresistance, PI4KA, ERK/AMPK/OXPHOS axis

## Abstract

**Background:** The emergence of chemoresistance in leukemia markedly impedes chemotherapeutic efficacy and dictates poor prognosis. Recent evidence has revealed that phosphatidylinositol 4 kinase-IIIα (PI4KA) plays a critical role in tumorigenesis. However, the molecular mechanisms of PI4KA-regulated chemoresistance and leukemogenesis remain largely unknown.

**Methods:** Liquid chromatography-mass spectrometry (LC-MS), patient samples and leukemia xenograft mouse models were used to investigate whether PI4KA was an effective target to overcome chemoresistance in leukemia. Enzyme-linked immunosorbent assay (ELISA) and molecular mechanics/generalized born surface area (MM/GBSA) method were employed to identify cepharanthine (CEP) as a novel PI4KA inhibitor.

**Results:** High expression of PI4KA was observed in drug-resistant leukemia cells or in relapsed leukemia patients, which was correlated with poor overall survival. Depletion of PI4KA sensitized drug-resistant leukemia cells to chemotherapeutic drugs *in vitro* and *in vivo* by regulating ERK/AMPK/OXPHOS axis. We also identified cepharanthine (CEP) as a novel PI4KA inhibitor, which could undermine the stability of the PI4KA/TTC7/FAM126 complex, enhancing the sensitivity of drug-resistant leukemia cells to chemotherapeutic drugs *in vitro* and *in vivo*.

**Conclusions:** Our study underscored the potential of therapeutic targeting of PI4KA to overcome chemoresistance in leukemia. A combination of the PI4KA inhibitor with classic chemotherapeutic agents could represent a novel therapeutic strategy for the treatment of refractory leukemia.

## Introduction

Leukemia is a highly aggressive hematological malignancy characterized by a broad spectrum of molecular alterations that influence clinical outcomes and provide potential drug development targets 1, 2. However, the emergence of chemoresistance markedly impedes chemotherapeutic efficacy and dictates poor prognosis. Approximately 15%-30% of leukemia patients are resistant to chemotherapy, and 60%-80% of patients who achieve complete remission inevitably relapse and succumb to the disease 3-6. Therefore, prior evaluation of chemoresistance is crucial in therapeutic decision-making and prognosis.

Phosphatidylinositol 4 kinases (PI4K) phosphorylate the D4 position in the inositol headgroup of phosphatidylinositol (PI) to generate phosphatidylinositol-4-phosphate (PtdIns4P) 7, 8. PI4KIIIα (also called PI4KA), primarily localized at the plasma membrane, plays a critical role in PIP metabolism 9 and is the primary source of the PtdIns4P pool that is converted into the signaling lipids PI4, 5P_2_, PI3, and 5P_3_. It interacts to form complexes with the TTC7/FAM126 heterodimer, and the stimulatory effect of TTC7/FAM126 on PI4KA catalytic activity has been observed *in vitro*. PI4KA is an essential enzyme, and its genetic or pharmacological targeting results in embryonic lethality 10, 11.

Recent evidence has revealed that PI4KA plays a critical role in regulating tumorigenesis through interactions with its regulatory proteins. EFR3A, an adapter protein for PI4KA, was reported to bind preferentially to oncogenic KRAS, leading to sustained recruitment of KRAS effectors and signaling that promote tumorigenesis 12. CXCR4, a G-protein coupled chemokine receptor, regulates PtdIns4P production on the plasma membrane via interacting with the cellular PI4KA machinery, resulting in the activation of intracellular signaling pathways culminating in cell migration and tumor metastasis 13. A recent study has also revealed a requirement for PI4KA in chemoresistance. Overexpression of PI4KA is associated with a poor prognosis of human hepatocellular carcinoma, suggesting that PI4KA may have an underappreciated function in the constitutive chemoresistance of cancers recalcitrant to apoptotic induction 14. Therefore, exploring the novel PI4KA role in the regulation of chemoresistance and chemotherapy sensitization may provide novel therapeutic strategies for treating leukemia.

In this study, we first identified PI4KA as a novel target that overcomes chemoresistance in leukemia. Mechanistically, knocking out PI4KA resulted in the interruption of ERK/AMPK signaling, inhibiting oxidative phosphorylation (OXPHOS) and interference with mitochondrial metabolism. We also discovered that cepharanthine (CEP), a novel PI4KA inhibitor, could enhance the sensitivity of drug-resistant leukemia cells to chemotherapeutic drugs *in vitro* and *in vivo*. Our findings suggest that a combination of PI4KA inhibitor with classic chemotherapeutic agents could be a novel therapeutic strategy for treating drug-resistant leukemia.

## Results

### Identification of PI4KA as a novel target for overcoming chemoresistance in leukemia

We first tested the sensitivity of the parental and resistant leukemia cells to doxorubicin (DOX) by using the CCK-8 assay. Systematic dose-response assessments of K562/Adr and HL-60/Adr cells treated with DOX showed an ∼106- and 10-fold shift, respectively, in IC_50_ values compared with drug-sensitive parental K562 and HL-60 cells (**Figure S1**), suggesting that K562/Adr and HL-60/Adr cells may have acquired a stable mechanism of DOX resistance.

**Figure 1:**
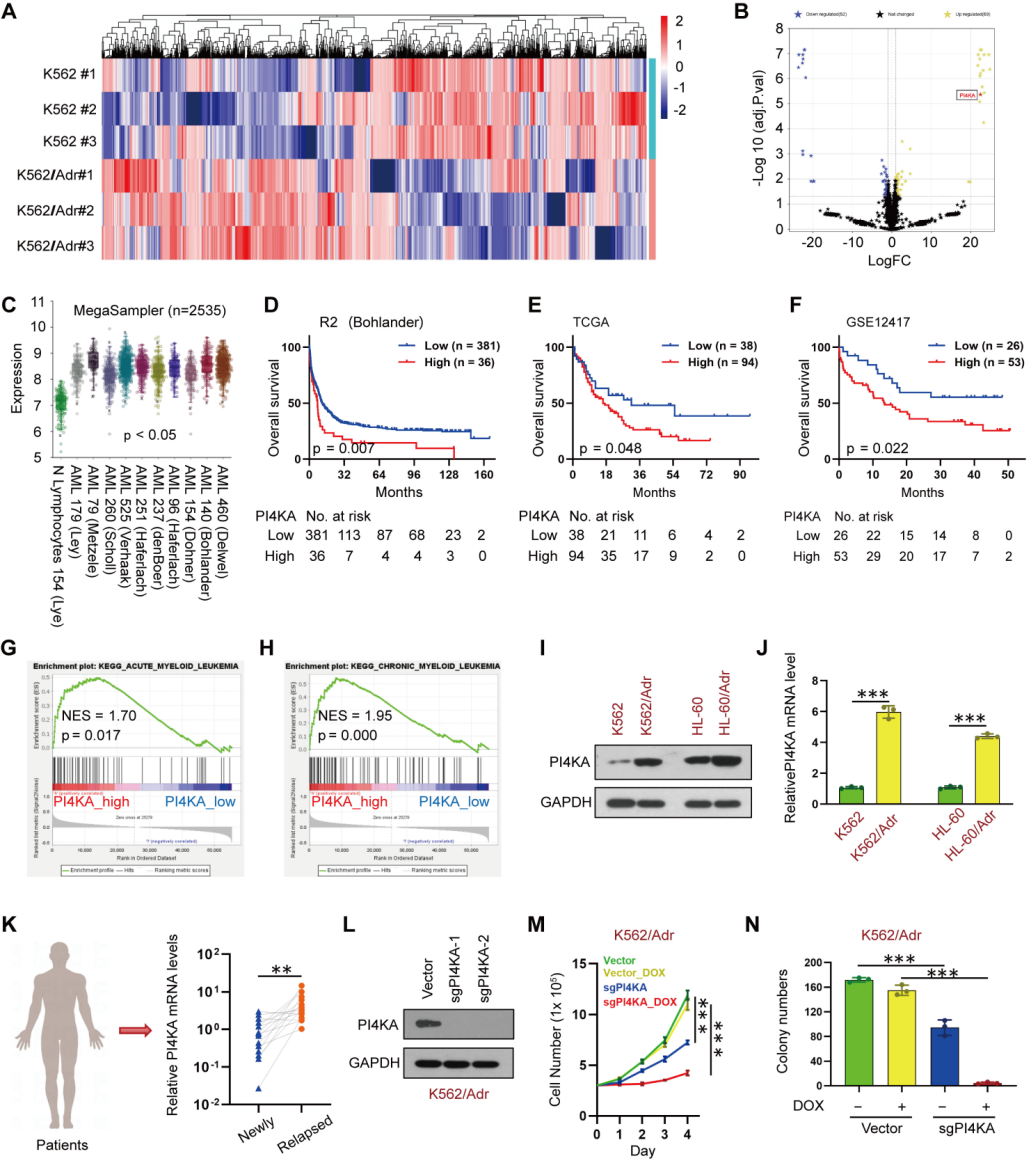
** PI4KA was identified as a novel target for overcoming chemoresistance in leukemia**. **(A, B)** Heatmap of all the DEPs and volcano plot of significant DEPs (n = 3). **(C)** Expression of PI4KA in leukemia compared with normal leukocytes were performed in R2 databases. **(D-F)** Overall survival analysis of high-PI4KA leukemia patients versus low-PI4KA leukemia patients in R2, TCGA and GSE12417 databases. **(G, H)** GSEA comparison of the PI4KA low and high expression subgroups of leukemia patients in the TCGA dataset. **(I, J)** Protein and mRNA levels of PI4KA in either sensitive leukemia cells or resistant leukemia cells (n = 3, ***p < 0.001). **(K)** RNA levels of PI4KA in the bone marrow from 15 patients with newly diagnosed and relapsed leukemia (n = 15, ***p < 0.001). **(L)** Knockout of PI4KA by CRISPR/Cas9 system. The protein level of PI4KA was analyzed by Western Blot in K562/Adrcells transfected with sgRNA targeting PI4KA (sgPI4KA-1, sgPI4KA-2). **(M)** Cell number was detected using Beckman Coulter Z2 Particle Counter (n = 3, ***p < 0.001). **(N)** Cell colony was detected by using soft agar assay (n = 3, ***p < 0.001).

We performed a quantitative proteomic analysis in paired K562 and K562/Adr leukemia cell lines to identify the potential target that might be associated with chemoresistance in leukemia (**Figure 1A** and **Table S2**). Among 69 upregulated differentially expressed proteins (DEPs), the expression of PI4KA was significantly increased in K562/Adr cells compared to the parental K562 cells (**Figure 1B**). The potential prognostic value of PI4KA expression for leukemia patients was explored by using the R2, TCGA, and GEO databases. The R2 genomic analysis indicated that the mRNA expression of PI4KA was significantly upregulated in leukemia patient datasets compared to the normal leukocytes/control dataset (**Figure 1C**). However, when compared to normal hematopoietic/myeloid progenitors, the expression did not differ between normal and acute myeloid leukemia in the Bloodspot (https://servers.binf.ku.dk/bloodspot/) tools (**Figure S2**). The data in Bloodspot showed that PI4KA was expressed at a higher level in progenitors than in mature blood cells. Additionally, the elevated expression of PI4KA was significantly associated with decreased overall survival of leukemia patients (**Figure 1D-F**).

Subsequently, we used Gene Set Enrichment Analysis (GSEA) to identify differential enrichment in gene sets between the PI4KA expression-high and PI4KA expression-low groups in the TCGA database. Remarkably, the PI4KA expression-high group genes were mainly enriched in both acute myelocytic leukemia and chronic myelocytic leukemia pathogenesis pathways (**Figure 1G-H**). We also examined PI4KA expression at protein and mRNA levels in leukemia- sensitive cells (K562, HL-60) and multidrug-resistant cells (K562/Adr and HL-60/Adr). In contrast to leukemia-sensitive cells, the expression of PI4KA at protein and mRNA levels was significantly upregulated in leukemia-resistant cell lines (**Figure 1I-J**). To confirm that the upregulation of PI4KA is associated with chemoresistance in leukemia, PI4KA expression at the mRNA level in the bone marrow (BM) mononuclear cells from 15 paired diagnosis-relapse patients was examined by using qRT-PCR. We found that PI4KA mRNA levels in BM of patients with relapsed leukemia were significantly higher than those in newly diagnosed leukemia (**Figure 1K**).

To ascertain whether PI4KA expression was associated with chemoresistance in leukemia, we used PI4KA sgRNAs to knock out the PI4KA gene in K562/Adr cells using CRISPR/Cas9 (**Figure 1L**). Notably, knockout of PI4KA significantly enhanced DOX-inhibited cell viability and colony formation in multidrug-resistant leukemia cells (**Figure 1M-N** and **Figure S3-S4**). These results demonstrated that PI4KA could be a novel target for chemoresistance in leukemia.

### Depletion of PI4KA enhances the sensitivity of resistant leukemia cells to DOX *in vivo*

**Figure 2:**
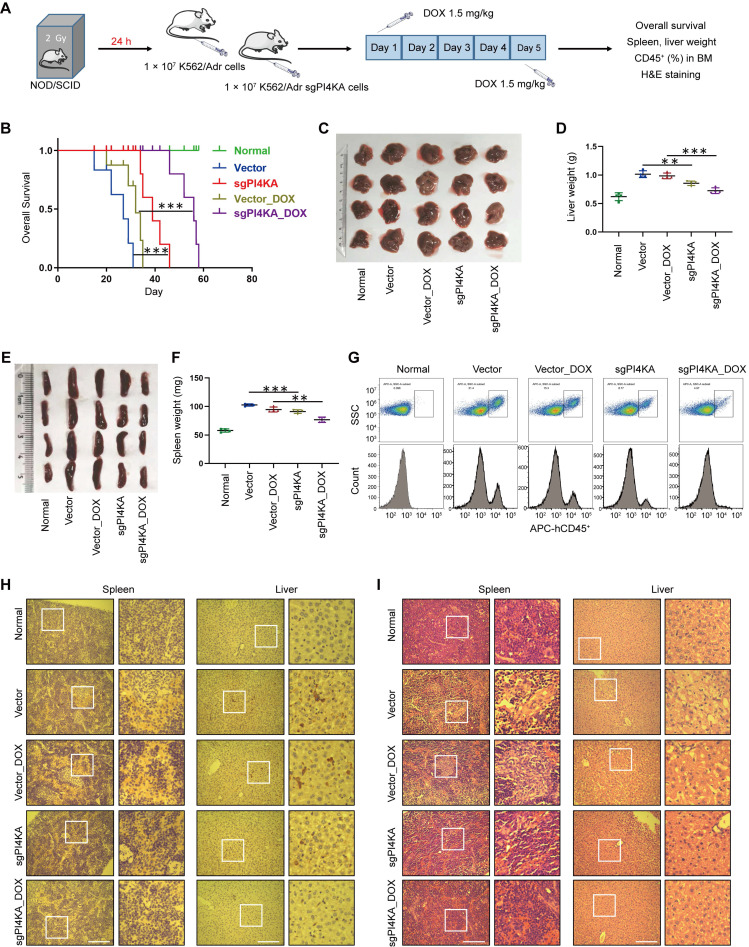
** Depletion of PI4KA sensitizes K562/Adr cells to DOX* in vivo*. (A)** Treatment regimen. **(B)** Comparison of the overall survival of different group (n = 5 mice per group, ***p < 0.001). **(C-F)** Spleens and livers were excised, measured and weighed at the end of the experiment (n = 4 mice per group, **p < 0.01, ***p < 0.001). **(G)** The percentage of human CD45^+^ cells in BM was determined by flow cytometry. **(H)** The histological sections of spleen and liver were stained for human CD45^+^. Scar bar, 100 µm. **(I)** The representative images of H&E staining for determination of morphology in sections of spleen and liver. Scar bar, 100 µm.

We subsequently assessed whether PI4KA knockout could inhibit leukemogenesis *in vivo* by using the drug-resistant leukemia xenograft mouse model. All NOD/SCID mice were irradiated at 2 Gy and transplanted by tail-vein injection with normal saline, vector-K562/Adr cells, or sgPI4KA-K562/Adr cells (**Figure 2A**). To confirm whether the cells that eventually grew out in the knockout cases *in vitro* and *in vivo* still lacked PI4KA, qRT-PCR was used to verify PI4KA depletion in resistant leukemia xenograft mouse primary cells (**Figure S5**). As shown in **Figure 2B**, mice transplanted with vector-K562/Adr cells had the shortest survival, whereas mice transplanted with sgPI4KA-K562/Adr cells had prolonged overall survival. Treating vector mice with DOX resulted in a minor increase in their survival time. However, a combination of sgPI4KA+DOX significantly increased the survival time of the mice. Severe hepatosplenomegaly was observed in mice transplanted with vector-K562/Adr cells and the DOX treatment slightly decreased the size and weight of the spleen and liver. Also, knocking out PI4KA significantly reduced the size and weight of the spleen compared to the vector control. Moreover, a combination of sgPI4KA+DOX further decreased the size and weight of the spleen and liver compared to that of vector mice treated with DOX (**Figure 2C-F**).

Flow cytometry analysis showed increased percentages of CD45^+^ cells in the BM of mice transplanted with vector-K562/Adr cells, and treatment with DOX modestly decreased the percentage of BM CD45^+^ cells. Knocking out PI4KA significantly reduced percentages of CD45^+^ cells in BM, and a combination of sgPI4KA+DOX further decreased percentages of CD45^+^ cells in the BM comparable to saline control mice (**Figure 2G**). Immunostaining of spleen and liver tissue slices with the proliferation marker CD45^+^ revealed that CD45^+^-positive cells were significantly higher in spleens and livers of mice transplanted with vector-K562/Adr cells compared to the saline control mice, whereas CD45^+^-positive cells were lower in the spleens and livers of mice transplanted with sgPI4KA-K562/Adr cells (**Figure 2H**). Hematoxylin and eosin (HE) staining of spleen and liver sections of mice transplanted with vector-K562/Adr cells showed infiltration with leukemic cells. In contrast, the number of leukemic cells in these organs was lower in mice transplanted with sgPI4KA-K562/Adr cells (**Figure 2I**). Furthermore, mice treated with sgPI4KA+DOX showed no pathological changes in morphology and no significant signs of necrosis and infiltration of inflammatory cells in the spleen and liver (**Figure 2H-I**). Together, these findings suggested that knockout of PI4KA could inhibit drug-resistant leukemogenesis and enhance the sensitivity of K562/Adr cells to DOX *in vivo*.

### PI4KA promotes chemoresistance in leukemia cells by regulating OXPHOS

**Figure 3:**
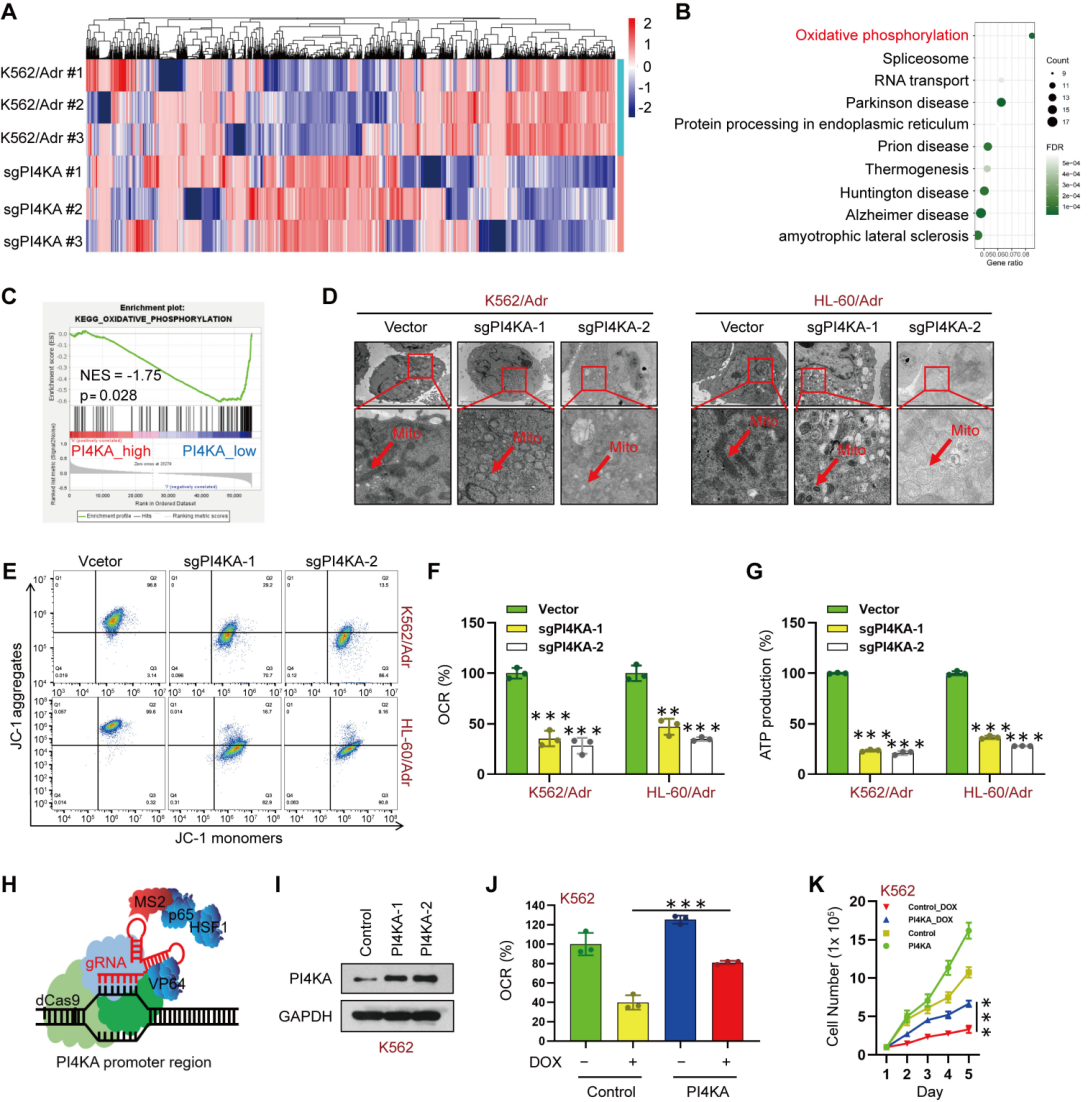
** Knocking out PI4KA inhibited OXPHOS in drug-resistant leukemia cells. (A)** Heatmap of all the differentially expressed proteins between K562/Adr and K562/Adr-sgPI4KA cells (n = 3). **(B)** KEGG enrichment analysis of signaling pathways. **(C)** GSEA comparison of the PI4KA low and high expression subgroups of leukemia patients in the TCGA dataset. **(D)** Transmission electron microscopy observation of mitochondrial morphologies. Scar bar, 1 µm. **(E)** Mitochondrial membrane potential was analyzed by the JC-1 aggregate/monomer fluorescence ratio. **(F, G)** The OCR and levels of ATP production were assayed by using commercially available assay kits (n = 3, ***p < 0.001, compared with vector). **(H, I)** Schematic illustration of CRISPR/Cas9-based Synergistic Activation Mediator (SAM) system. The protein level of PI4KA was analyzed by Western Blot in K562 cells transfected with dCas9 and guide RNA targeting PI4KA promoter (PI4KA-1, PI4KA-2). **(J)** The levels of OCR was by using commercially available assay kits (n = 3, ***p < 0.001). **(K)** Cells transfected with control or PI4KA plasmid and treated with DOX. Cell number was detected using Beckman Coulter Z2 Particle Counter (n = 3, ***p < 0.001).

We conducted quantitative proteomic analysis in paired K562/Adr and PI4KA knockout K562/Adr cell lines to explore the molecular mechanism underlying the PI4KA regulation of chemoresistance in leukemia (**Figure 3A** and **Table S3**). We performed the Kyoto Encyclopedia of Genes and Genomes (KEGG) analysis to evaluate signaling pathways in PI4KA knockout. As shown in **Figure 3B**, OXPHOS may play an essential role in chemoresistance regulation by PI4KA in leukemia cells. Remarkably, genes in the PI4KA expression-low group were mainly enriched in metabolic pathways such as oxidative phosphorylation as determined by the GSEA analysis in TCGA databases (**Figure 3C**). These results indicated that PI4KA might be a key regulator for OXPHOS in leukemia.

Since mitochondria are known to be involved in ATP synthesis signaling pathways through OXPHOS 15-17, we examined the effects of PI4KA knockout on the ultrastructural morphology of mitochondria by using transmission electron microscopy. As displayed in **Figure 3D**, knocking out PI4KA caused mitochondrial swelling and vacuolation in K562/Adr and HL-60/Adr cells, indicating that mitochondria might be an important therapeutic target of PI4KA. Knocking out PI4KA also decreased mitochondrial membrane potential in drug-resistant leukemia cells (**Figure 3E**). We next determined OXPHOS in drug-resistant leukemia cells. Knocking out PI4KA significantly decreased the mitochondrial oxygen consumption rate in both K562/Adr and HL-60/Adr cells (**Figure 3F**). Moreover, cellular ATP production significantly decreased in PI4KA knockout drug-resistant leukemia cells (**Figure 3G**). The precise contribution of PI4KA to OXPHOS and drug resistance was assessed by generating the PI4KA overexpressing K562 and HL-60 stable cell lines using the CRISPR-SAM system (**Figure 3H-I** and **Figure S6A**). Interestingly, overexpressing PI4KA significantly attenuated OXPHOS inhibition mediated by DOX in K562 and HL-60 cells (**Figure 3J** and **Figure S6B**) and decreased sensitivity to DOX compared with control cells (**Figure 3K** and **Figure S6C)**. Taken together, these findings suggested that PI4KA controls mitochondrial function and OXPHOS to regulate drug resistance and proliferation in leukemia cells.

### PI4KA regulates OXPHOS through ERK/AMPK signaling

**Figure 4:**
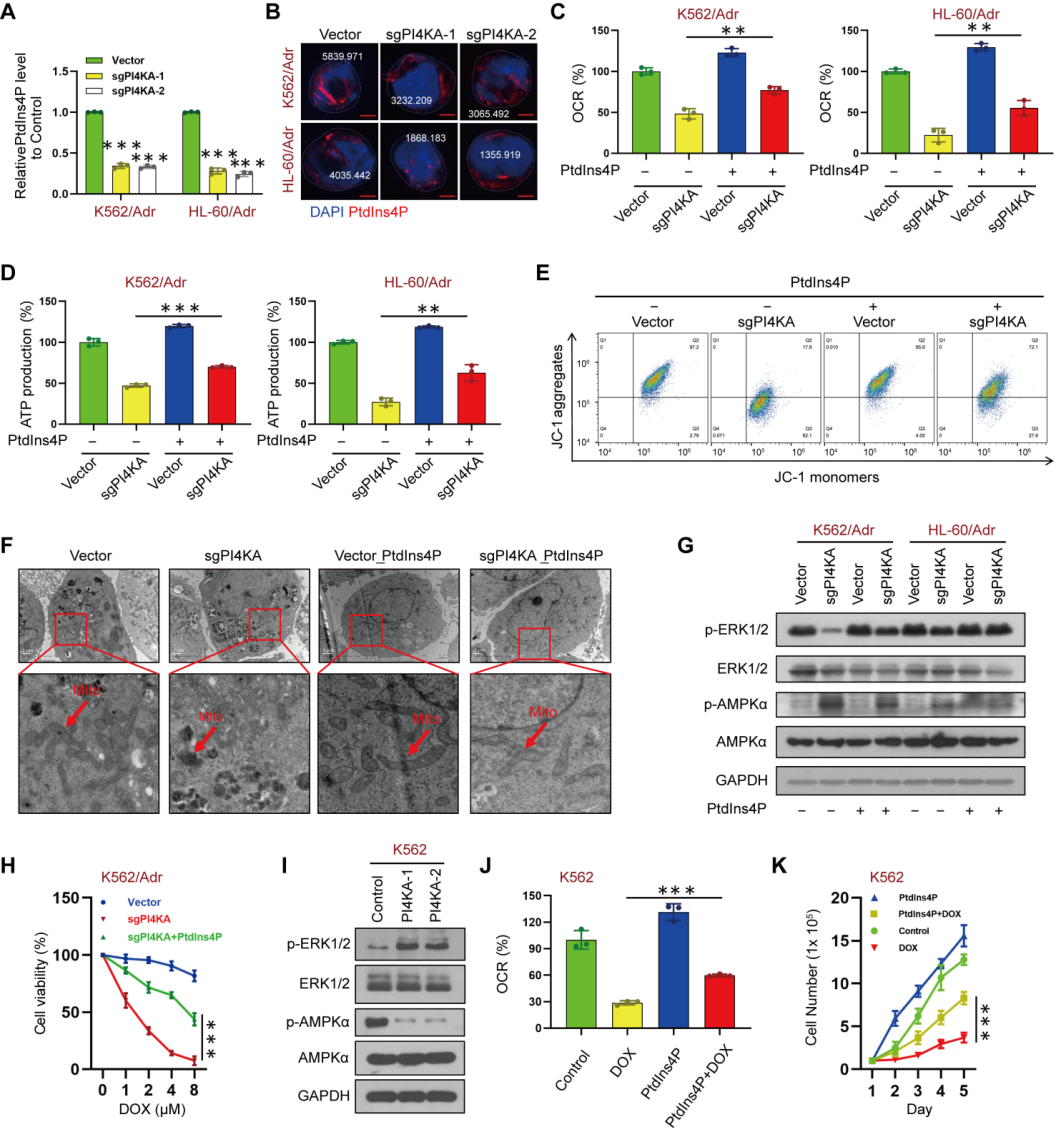
** PI4KA regulated OXPHOS via ERK/AMPK signaling. (A)** The levels of PtdIns4P were assayed by ELISA kit (n = 3, ***p < 0.001, compared with control). **(B)** PtdIns4P polarization was analyzed by confocal laser scanning microscope. And the average fluorescence intensity of PtdIns4P was marked. Scar bar, 2 µm. **(C, D)** Cells transfected with sgPI4KA. OCR and ATP production were determined by using commercially available assay kits (n = 3, **p < 0.01). **(E)** Mitochondrial membrane potential was analyzed by the JC-1 aggregate/monomer fluorescence ratio. **(F)** Transmission electron microscopy observation of mitochondrial morphologies. Scar bar, 1 µm. **(G)** The expression of ERK/AMPK signaling pathway related proteins was detected by Western blot. **(H)** Cell viability was assessed by CCK-8 kits (n = 3, ***p < 0.001). **(I)** The expression of ERK/AMPK signaling pathway related proteins was detected by Western blot. **(J)** The levels of OCR was assessed by using commercially available assay kits (n = 3, ***p < 0.001). **(K)** Cells treated with PtdIns4P or DOX. Cell number was detected using Beckman Coulter Z2 Particle Counter (n = 3, ***p < 0.001).

ELISA was used to assess the precise contribution of PI4KA to the plasma membrane pool of PtdIns4P, its role in OXPHOS and mitochondrial metabolism, and quantification of phosphoinositide levels. The results showed that knocking out PI4KA significantly decreased PtdIns4P levels in K562/Adr and HL-60/Adr cells (**Figure 4A**). Furthermore, immunofluorescence microscopy indicated that knocking out PI4KA impaired PtdIns4P polarization in the plasma membrane of drug-resistant leukemia cells (**Figure 4B** and **Figure S7**). Interestingly, increasing the PtdIns4P level by “PtdIns4P shuttling” rescued the PI4KA knockout-mediated inhibition of OXPHOS and reduced ATP (**Figure 4C-D**). “PtdIns4P shuttling” also rescued mitochondrial swelling and vacuolation and decreased mitochondrial membrane potential mediated by knocking out PI4KA (**Figure 4E-F** and **Figure S8**).

It has been shown that increased PtdIns4P promoted active KRAS accumulation and nanoclustering at the plasma membrane, and inactivation of PI4KA inhibited oncogenic KRAS signaling and transformation via dephosphorylation of ERK 12. Also, AMPK, which is regulated by ERK, is known to play a central role in the regulation of cellular metabolism 18-22. We tested the hypothesis that PI4KA regulates mitochondrial metabolism via ERK/AMPK signaling by investigating the effects of PI4KA on the phosphorylation of ERK/AMPK proteins by Western blotting. **Figure 4G** shows that knocking out PI4KA significantly decreased ERK phosphorylation while increasing AMPK phosphorylation. In contrast, “PtdIns4P shuttling” markedly rescued the phosphorylation of AMPK and dephosphorylation of ERK by knocking out PI4KA (**Figure 4G**). Meanwhile, we found that knockout of PI4KA resensitized the resistant cell lines to DOX and “PtdIns4P shuttling” could reverse this effect (**Figure 4H** and **Figure S9A)**. Overexpressing PI4KA significantly increased the phosphorylation of ERK and decreased the phosphorylation of AMPK in K562 and HL-60 cell lines (**Figure 4I** and **Figure S9B**). Interestingly, “PtdIns4P shuttling” partly rescued the inhibition of OXPHOS and cell proliferation mediated by DOX and decreased sensitivity of K562 and HL-60 cell lines to DOX (**Figure 4J-K** and **Figure S9C-D**). Collectively, these findings suggested that PI4KA plays a critical role in chemoresistance via the ERK/AMPK/OXPHOS signaling pathway by regulating the production and polarization of PtdIns4P in leukemia.

### Identification of CEP as a potential PI4KA inhibitor

**Figure 5:**
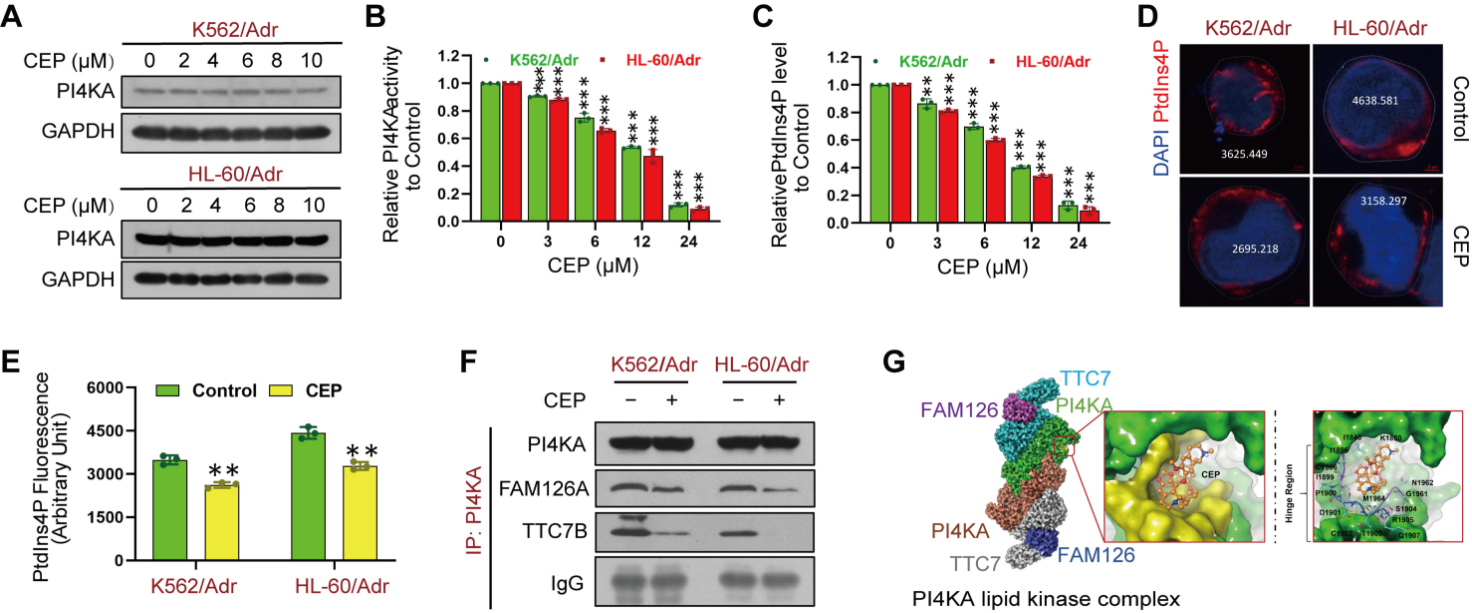
**CEP was identified as a novel PI4KA inhibitor. (A)** The expression of PI4KA was assayed by Western blot. **(B, C)** The activity of PI4KA and levels of PtdIns4P were assayed by ELISA kit (n = 3, **p < 0.01, ***p < 0.001, compared with control). **(D, E)** Cells were treated without or with CEP (6 µM), after which PtdIns4P polarization was analyzed by confocal laser scanning microscope. And the average fluorescence intensity of PtdIns4P was marked. Scar bar, 2 µm. (n = 3, ***p < 0.001, compared with control). **(F)** Cells were treated without or with CEP (6 µM) for 24 hours. The immunoprecipitated PI4KA and TTC7B, FAM126A proteins were examined by Western blot. **(G)** Binding modes of CEP to PI4KA.

The above results indicated that targeting PI4KA may be an effective way to overcome chemoresistance in leukemia. We screened 9 compounds in resistant leukemia cells and normal PBMCs to obtain a high-efficiency and low-toxicity PI4KA inhibitor. Cell viability was assayed by CCK-8 kit, and the expression and activity of PI4KA were determined by Western blotting and ELISA, respectively (**Figure S10**). Among the compounds screened, we identified CEP as a novel PI4KA inhibitor. Treating either K562/Adr or HL-60/Adr cells with CEP did not modify PI4KA expression at the protein level (**Figure 5A**) but significantly decreased kinase activity of PI4KA and the production of PtdIns4P both in leukemia-resistant cell lines and patient samples (**Figure 5B-C** and **Figure S11**). Moreover, CEP impaired PtdIns4P polarization in leukemia-resistant cells (**Figure 5D-E**).

There is increasing evidence that PI4KA interaction with TTC7/FAM126 is critical for PI4KA catalytic activity 23-26. We further investigated how CEP inhibited the activity of PI4KA by co-immunoprecipitating PI4KA, TTC7B, and FAM126A, followed by Western blot analysis. As displayed in **Figure 5F** and **Figure S12**, CEP did not change the overall levels of FAM126A and TTC7B. PI4KA was coimmunoprecipitated with TTC7B and FAM126A in control cells, but treatment with CEP decreased the interaction of PI4KA with FAM126A or TTC7B. Next, we used the induced fit docking method to predict the binding mode of CEP to PI4KA. **Figure 5G** shows that CEP could bind to the hinge region of PI4KA via non-canonical hydrogen bonding with the backbone of proline-1900 and aspartate-1901. The binding affinity of CEP was also calculated by using MM/GBSA approach. The delta G value of two ligands indicated that CEP might bind to PI4KA (**Figure S13**). From the binding conformation and key interaction with the crucial hinge region, it could be deduced that CEP can be accommodated in the binding pocket of PI4KA to destabilize the PI4KA/TTC7/FAM126 complex and inhibit PI4KA function.

### Pharmacological inhibition of PI4KA sensitizes resistant leukemia cells to chemotherapeutic drugs

**Figure 6:**
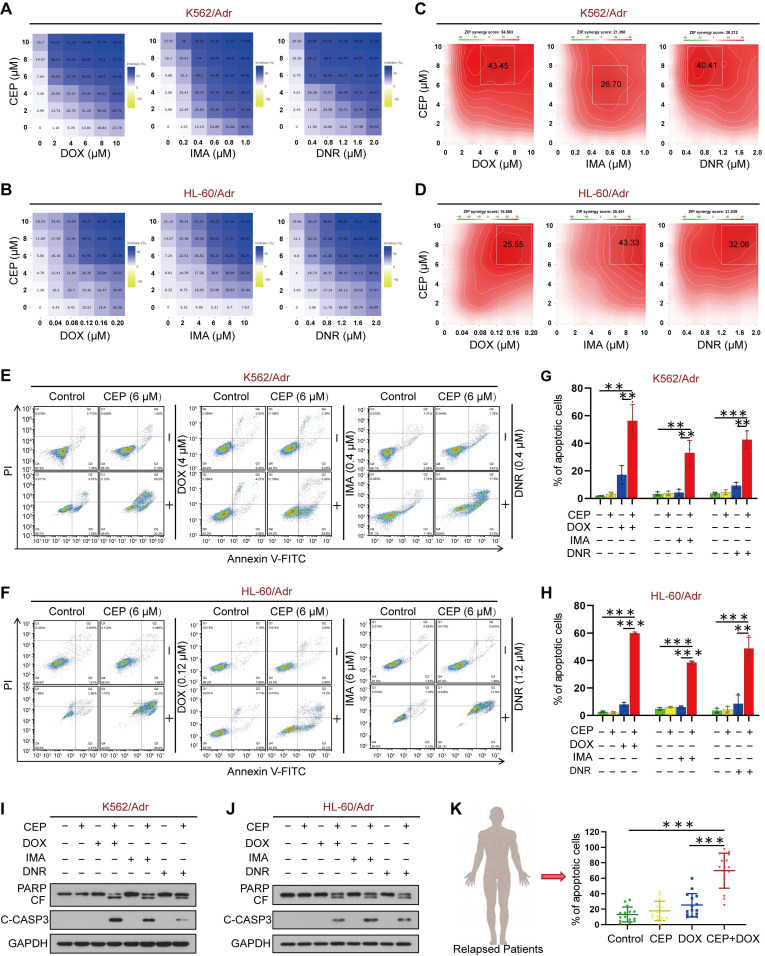
** Synergistic interaction between CEP and chemotherapeutic drugs *in vitro*. (A-D)** Dose-dependent inhibition matrix and ZIP synergy plot of drug-resistant leukemia cells (K562/Adr and HL-60/Adr) treated with various concentrations of drugs (DOX, IMA and DNR) as indicated for 48 hours. Value in the white box represents the averaged synergy score for the region of highest synergy. **(E**-**H)** After treatment with combination of CEP and different drugs (DOX, IMA and DNR) for 48 hours, cell apoptosis was evaluated by flow cytometry (n = 3, **p < 0.01, ***p < 0.001). **(I**-**J)** The expression of PARP and cleaved caspase-3 were determined by Western blot. **(K)** The primary cells derived form 15 patients with relapsed leukemia were treated without or with CEP or DOX alone or combination of CEP+DOX, after which cell apoptosis was determined by flow cytometry (n = 15, ***p < 0.001).

We next used the ZIP independence model to assess the synergistic effects of CEP and chemotherapeutic drugs on cell viability in K562/Adr and HL-60/Adr cells. The areas within a dose inhibition matrix revealed a synergistic effect across a wide range of doses (**Figure 6A-B**). We calculated the ZIP synergy score across all doses (ZIP Synergy scores>0 indicate synergism) to quantify the degree of the synergistic effect. The average ZIP synergy score in K562/Adr cells was 34.58 for DOX, 21.40 for imatinib (IMA), and 29.21 for daunorubicin (DNR) (**Figure 6C**). **Figure 6D** shows the average ZIP synergy score of 15.56 for DOX, 26.45 for IMA, and 21.03 for DNR in HL-60/Adr cells (**Figure 6D**). We also tested the combinatorial effects of a documented direct PI4KA inhibitor GSK-A1 and DOX in resistant leukemia cells. As shown in **Figure S14**, pretreatment with GSK-A1 could enhance DOX efficacy in resistant leukemia cells. However, GSK-A1 had higher cytotoxicity in normal cells than CEP (**Figure S15**).

To further explore the chemosensitizing characteristics of CEP, we investigated the synergistic effect of CEP and chemotherapeutic drugs on apoptosis in drug-resistant leukemia cells and primary leukemia cells from relapsed leukemia patients by using a flow cytometry assay. Treatment of K562/Adr and HL-60/Adr cells with CEP or chemotherapeutic drugs alone minimally increased apoptosis (~5% to ~10%), whereas combined treatment of these cells with CEP and chemotherapeutic drugs significantly increased apoptosis (~40% to ~50%) (**Figure 6E-H**). Consistent with these findings, combining CEP and chemotherapeutic drugs resulted in cleavage/activation of caspase 3 and degradation of PARP (**Figure 6I-J**). Furthermore, combination of CEP and chemotherapeutic drugs like DOX markedly increased apoptosis in primary cells from 15 relapsed leukemia patients (**Figure 6K** and **Figure S16**). These findings suggested that inhibition of PI4KA by CEP could enhance the sensitivity of drug-resistant leukemia cells to conventional chemotherapeutic drugs.

### Inhibition of PI4KA enhances the therapeutic efficacy of DOX in a drug-resistant leukemia xenograft mouse model

**Figure 7:**
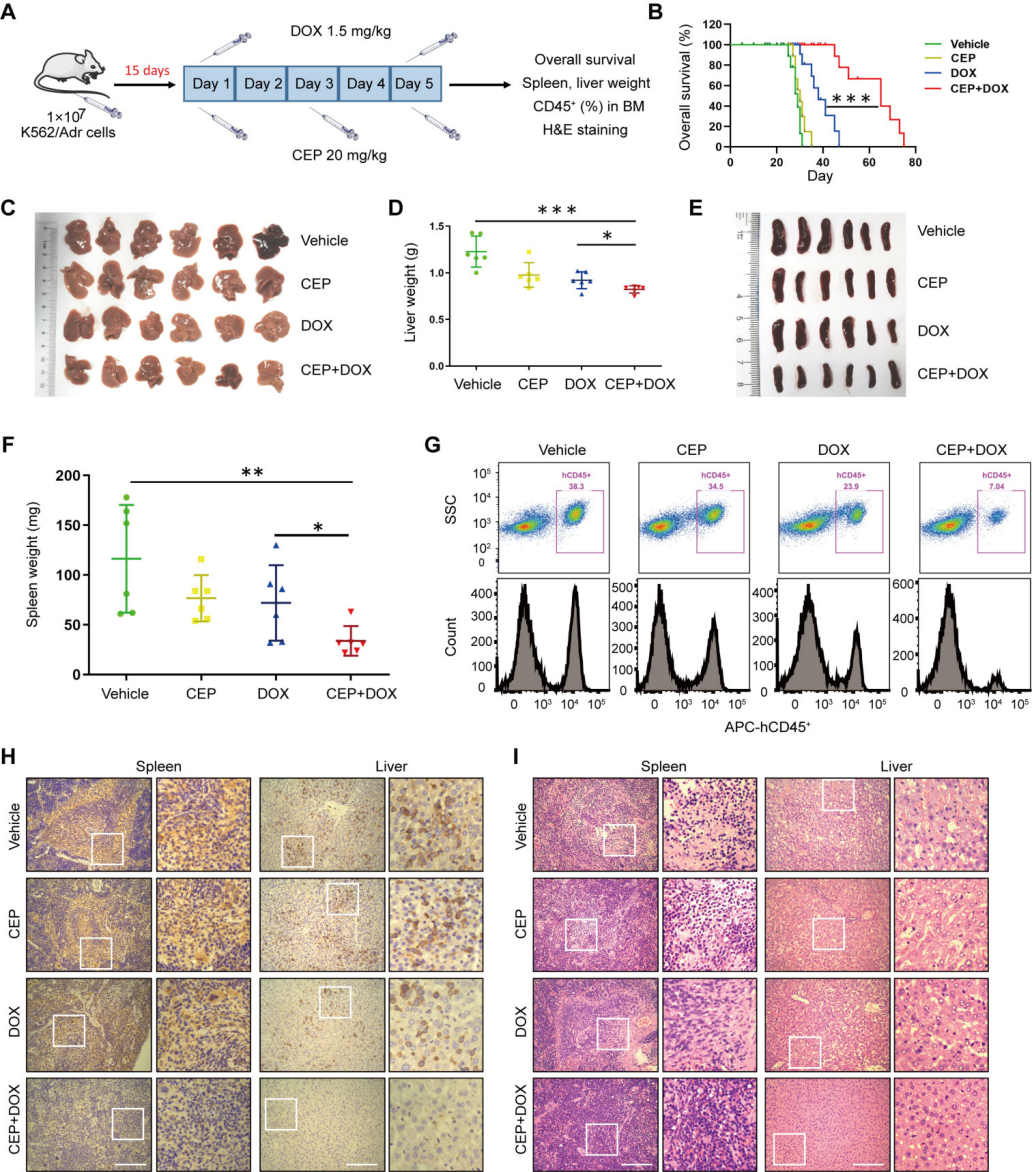
** The synergistic antileukemic interaction between CEP and DOX *in vivo*. (A)** Treatment regimen. **(B)** Comparison of the overall survival of mice between vehicle, CEP, DOX, and CEP+DOX (n = 8 mice per group, ***p < 0.001). **(C-F)** Spleens and livers were excised, measured and weighed at the end of the experiment (n = 6 mice per group, *p < 0.05, **p < 0.01, ***p < 0.001). **(G)** The percentage of human CD45^+^ cells in BM was determined by flow cytometry. **(H)** The histological sections of spleen and liver were stained for human CD45^+^. Scar bar, 100 µm. **(I)** The representative images of H&E staining for determination of morphology in sections of spleen and liver. Scar bar, 100 µm.

Next, we investigated whether our *in vitro* findings that inhibition of PI4KA by CEP-sensitized chemotherapeutic drug-induced cell death in drug-resistant leukemia cells could be replicated *in vivo*. We examined the antileukemic synergism of CEP and DOX in a drug-resistant leukemia xenograft mouse model. The NOD/SCID mice were irradiated at 2 Gy, and K562/Adr cells were transplanted by tail-vein injection. After 15 days of inoculation, mice received intraperitoneal injections of either vehicle, CEP (20 mg/kg), DOX (1.5 mg/kg), or a combination of CEP+DOX (**Figure 7A**). Kaplan-Meir survival analysis revealed that the median survival time of the vehicle control group was approximately 29 days. CEP treatment did not modify the median survival time (30 Days), and DOX treatment alone resulted in minor increases in the survival time (38 days). However, a combination of CEP+DOX significantly increased the median survival of mice to 65 days (**Figure 7B**). Mice transplanted with leukemia cells had severe splenomegaly and hepatomegaly, showing large spleens and livers in size and weight. Treating mice with either CEP or DOX alone resulted in a minor decrease in the size and weight of spleens and livers, whereas these parameters were significantly reduced by the combination treatment with CEP+DOX (**Figure 7C-F**).

To further examine whether CEP enhances the antileukemic efficacy of DOX *in vivo*, leukemia burden was determined by the percentage of human CD45^+^ cells in BM. Flow cytometry analysis showed CD45^+^ cells in BM of vehicle control mice, and CEP or DOX treatment alone modestly decreased percentages of BM in CD45^+^ cells, whereas the combination of CEP+DOX significantly reduced percentages of CD45^+^ cells in BM (**Figure 7G**). Immunostaining of spleen and liver tissue slides with the proliferation marker CD45^+^ revealed that CD45^+^-positive cells were significantly higher in the spleens and livers of mice transplanted with K562/Adr cells but substantially lower in mice treated with the combination of CEP+DOX (**Figure 7H**). The combination treatment group showed changes in morphology without significant signs of necrosis with infiltration of inflammatory cells and fibrosis in the spleen and liver (**Figure 7I**). These results indicated that inhibition of PI4KA enhances the antileukemic efficacy of chemotherapeutic drugs *in vivo*.

## Discussion

Poor outcomes in many leukemia patients are associated with chemo-refractory disease or relapse after initial chemotherapy. Chemoresistance is an important cause of chemotherapy failure in leukemia 27-29. Besides clinical and pathologic parameters, molecular biomarkers have been proposed to accurately identify patients with a high risk of recurrence 5, 30, 31. In this study, we demonstrated that PI4KA is involved in intrinsic chemoresistance of leukemia and leads to poor response to chemotherapeutic drugs and developed a PI4KA-targeting strategy to sensitize leukemia to adjuvant chemotherapy in proof-of-concept preclinical testing.

PI4KA is a lipid kinase and the primary source generating the PtdIns4P pool 32. Recent studies revealed that PI4KA is centrally involved in chemoresistance. RNA interference screening identified PI4KA as a mediator of resistance to cisplatin in medulloblastoma cell lines, suggesting that PI4KA has a functional role in the chemoresistance of cancers 33. We hypothesized that PI4KA regulates chemoresistance in leukemia, and inhibition of PI4KA activity may reverse chemoresistance in leukemia cells based on the following observations. First, overexpression of PI4KA was observed in drug-resistant leukemia cell lines. However, the parental/resistant cells used were not from the same source and, therefore could carry additional differences besides the chemoresistance selection features. Next, the bone marrow from paired diagnosis-relapse samples was used to assess the relative expression of PI4KA. In Bloodspot tools, normal (mature) blood cells express less PI4KA complicating the expression analyses since the leukemic cell content or blast percentage in each patient sample would affect the bulk PI4KA expression level. Therefore, it is essential to assess the leukemic cell content or blast percentage in patient samples by using a live cell counter or flow cytometer. The more rigorous approach would be to sort cells by flow cytometry and then perform follow-up experiments. Meanwhile, clinical sample database analysis has shown that high expression of PI4KA was associated with decreased overall survival of leukemia patients. Also, knockout of PI4KA could enhance the sensitivity of resistant leukemia cells to DOX *in vitro* and *in vitro*. Thus, our findings indicated that inhibition of PI4KA activity appears essential for chemoresistance in leukemia cells.

The molecular mechanisms underlying chemoresistance reversal in leukemia remain unclear. Many studies have reported that in chemotherapy-resistant human leukemia cells with high OXPHOS, inhibition of OXPHOS improved anti-leukemia drug efficacy and overcame drug resistance in leukemia 34-37. Inactivation of PI4KA inhibited oncogenic KRAS signaling and transformation via dephosphorylation of ERK, which modulated the activity of AMPK, a key regulator of mitochondrial metabolism 38-40. We speculated that PI4KA knockout plays a critical role in overcoming drug resistance through the interruption of ERK/AMPK-mediated OXPHOS and mitochondrial metabolism. The present results supported this hypothesis based on the following observations: knocking out PI4KA inhibited OXPHOS and mitochondrial metabolism, decreased phospho-ERK and increased phospho-AMPK levels, and “PtdIns4P shuttling” rescued inhibition of OXPHOS, phosphorylation of AMPK, and dephosphorylation of ERK by knocking out PI4KA in drug-resistant leukemia cells.

We also generated PI4KA-overexpressing stable K562 and HL-60 cell lines and found that PI4KA overexpression significantly attenuated OXPHOS inhibition mediated by DOX in both cells and decreased sensitivity to DOX compared to the control cells. Knockout of PI4KA could significantly increase the sensitivity of resistant leukemia cells to DOX by inhibiting OXPHOS. PI4KA appears to be a common essential gene 10, 11; DepMap (depmap.org) data also support the role of PI4KA as a common essential gene. Our results showed that regulation of OXPHOS by PI4KA might explain the general dependency on PI4KA. However, knocking out PI4KA in leukemia patients to treat refractory leukemia is difficult and impractical because of toxic side effects. Therefore, finding a highly effective and low toxic PI4KA inhibitor would be a reasonable alternative.

Next, we identified a natural product CEP as a novel PI4KA inhibitor. Since the discovery of TTC7 regulatory proteins in mammalian cells, an additional protein in the complex, FAM126, was identified subsequently 8. The cryo-EM structure of PI4KA/TTC7/FAM126 reveals that this complex formed a dimer of heterotrimers 26. This large complex has a dimer interface composed of the dimerization domain of PI4KA. Both TTC7/FAM126 regulatory proteins are required for the formation of a stable assembly by PI4KA, and their presence increases PI4KA activity 26. Mutations in TTC7A decreased its association with PI4KA, leading to decreased PI4KA stability 41. Consistent with these reports, our results indicated that CEP decreased PI4K interaction with either TTC7 or FAM126, inhibiting PI4KA activity and generating PtdIns4P. Importantly, inhibition of PI4KA by CEP enhanced the efficacy of chemotherapeutic agents in drug-resistant leukemia cells *in vitro* and *in vivo*.

**Figure 8:**
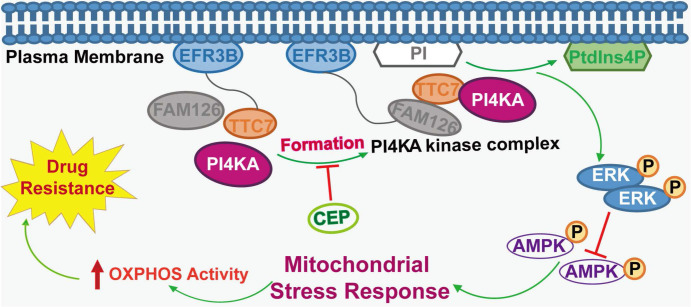
The model of PI4KA-mediated chemoresistance via modulating ERK/AMPK/ OXPHOS axis in leukemia.

In summary, we revealed a novel mechanism supporting the critical role of PI4KA in overcoming chemoresistance in leukemia. Overexpression of PI4KA upregulated OXPHOS by modulating the ERK/AMPK axis, leading to chemoresistance in leukemia (**Figure 8**). Our study highlighted the potential of therapeutic targeting of PI4KA to overcome chemoresistance in leukemia. Our findings also suggested that combining a PI4KA inhibitor with classic chemotherapeutic agents could represent a novel therapeutic strategy for treating refractory leukemia.

## Materials and methods

### Patients sample

The bone marrow (BM) specimens were obtained from 15 paired diagnosis-relapse leukemia patients after informed consent and with the approval of the Southwest Hospital Institutional Review Board. The patient information was shown in **Table S1**. Cells were obtained by gradient centrifugation using human BM lymphocytes isolation kit from Haoyang Biological Products Technology Co., Ltd. (Tianjin, China) and cultured in RPMI 1640 medium containing 20% FBS for further assays.

### Cell culture

The human leukemia cell lines K562, HL-60 were purchased from ATCC (American Type Culture Collection, USA), K562/Adr, HL-60/Adr cell lines were obtained from Zeye Biological Technology Co., Ltd. (Shanghai, China) and Chuan Qiu Biotechnology Co.,Ltd. (Shanghai, China). The species origin of the cell lines was confirmed with PCR. The identity of the cell lines was authenticated with short tandem repeat profiling. The cell lines were checked free of mycoplasma contamination by PCR. Cells were cultured in IMDM or RPMI 1640 medium (Gibco, USA) with 10% fetal bovine serum (Gibco, USA) in a 5% CO_2_ incubator at 37 °C.

### Compound reagents and antibodies

Doxorubicin (HY-15142) was from Medchem Express (NJ, USA). Imatinib (S2475) was from Selleck Chemicals (Houston, USA). Cepharanthine (A0653) was from Must BioTechnology (Chengdu, China). Daunorubicin (D8809) was from Sigma-Aldrich (Gillingham, UK).

Primary antibodies against phospho-ERK1/2 (sc-7383) were purchased from Santa Cruz Biotechnology (Dallas, USA). Cleaved-Caspase 3 (9661), PARP (9532), phospho-AMPKα (2535), AMPKα (2793), GADPH (2118) were purchased from Cell Signaling Technology (Boston, USA). ERK1/2 (343830) was from Zen-Bio.Co.Ltd. (Chengdu, China). PI4KA (12411-1-AP) was from Proteintech (Chicago, USA). TTC7B (ERP15995) was from Abcam (Cambridge, UK). FAM126A (bs-11554R) was from Bioss Biotechnology Co., Ltd. (Beijing, China). PtdIns(4)P (Z-P004) was from Echelon Biosciences Inc (Salt Lake City, USA). Secondary antibodies were horseradish peroxidase (HRP)-conjugated anti-rabbit (0741516), anti-mouse (0741802) IgG purchased from Kirkegaard and Perry Laboratories (Gaithersburg, USA) and goat anti-mouse Alexa Fluor 647 (A21235) were from Thermo Fisher Scientific (Massachusetts, USA).

### Soft agar and colony formation

Sustainment gel was mixed 0.6% agarose (Sigma-Aldrich) in IMDM or 1640 medium in 12-well plates. Cultivate gel was mixed 0.3% agarose in IMDM or 1640 medium with 10% FBS. One thousand cells were cultured in 1 ml cultivate gel above concretionary sustainment gel. Colonies were photographed and the number of colonies was counted after 30 days.

### Immunofluorescence

After drug treatment, cells were collected by centrifugation, attached to glass coverslips pre-coated with polylysine and fixed with cold-acetone for 30 minutes, permeabilized with 0.1% Triton X-100 (Thermo Scientific, USA) and blocked with goat serum (Beyotime Biotechnology, China) for 30 minutes. Cells were incubated with primary antibodies at 4 °C overnight and then incubated with secondary antibodies conjugated with Alexa Fluor 647 for 1 hours at room temperature. After washing three times with PBS, the nuclei were counterstained with 0.1 μg/ml DAPI (Sigma-Aldrich, USA) for 5 minutes. Cells were viewed using a laser-scanning confocal microscope (Zeiss, Germany). All images were analyzed by ImageJ software (MD, USA).

### Western blotting and immunoprecipitation

Cell protein lysates ranging from 15 μg to 30 μg were separated using SDS-PAGE and then transferred to PVDF membranes (Bio-Rad, USA). After blocking with 5% fat-free milk, membrane was probed at 4 °C with primary antibodies for 24 hours. Protein bands were detected by incubating with horseradish peroxidase-conjugated antibodies (Kirkegaard and Perry Laboratories, USA) and the signal was detected using Clarity Western ECL Substrate (Bio-Rad, USA). For immunoprecipitation, equal quantities of proteins were incubated with primary antibodies on a rocking platform at 4 °C. Protein A/G agarose beads (Beyotime Technology, China) were used to collect immune complexes and followed by 5 washes in ice-cold PBS, and finally subjected to western blot assay.

### Mitochondrial membrane potential (ΔΨm) detection

Cells were collected and resuspended in fresh medium. Cells were incubated with 0.5 mL JC-1 staining working solution at 37 °C in a CO_2_ incubator for 20 minutes according to the manufacturer's instructions (C2005, Beyotime, China). The fluorescence labeled cells were washed using ice-cold phosphate buffered saline (PBS) and measured by flow cytometry.

### Biochemical assay

ATP and PtdIns4P quantification, Oxygen consumption assay from the indicated samples were done using a commercially available kit following manufacturer's instructions (Beyotime S0026B, Echelon Biosciences K-4000E, Abcam, ab197243, respectively).

### Establishment of PI4KA knockout cells

PI4KA knockout cell lines were established using the CRISPR/Cas9 system. The procedure was slightly modified form the original protocol by the Zhang Lab. **Table S4** shows sgRNA sequences designed using the sgRNA designer (https://zlab.bio/guide-design-resources). Briefly, HEK293T cells (ATCC) were maintained in a humidified 37 °C incubator with 5% CO_2_. HEK293T cells were transfected with PEI (Polyethylenimin), VSVg plasmid, PAX2 plasmid and empty vector or dual gRNA expression vector for 48 hours. The supernatant was collected for infection. K562/Adr or HL-60/Adr cells were infected with virus for 48 hours and then subjected to selection with puromycin for 7 days. Absence of PI4KA expression was confirmed by western blot.

### PI4KA activity assay

Immunoprecipitation and PI4-Kinase Activity ELISA Kit (Echelon Biosciences, K-4000K) were used to analyse PI4KA activity. Transfer 400 μL cell lysate to 1.5 mL centrifuge tube with 2 μL PI4KA antibody. Incubate 3 hours to overnight at 4 °C with agitation and then add 60 μL Protein A+G agarose beads to mixture and incubate 2~3 hours at 4 °C. Briefly centrifuge to pellet beads and wash the bead complex 3 times with ice cold PBS. Centrifuge and discard solution after each wash. Wash 1~3 times with diluent and resuspend bead complex in diluent. Proceed immediately with the PI4-Kinase activity assay by adding 5 μL/well IP enzyme (with bead) to the colored mixing plate following the “PI4-Kinase Activity Assay Protocol” section.

### PtdIns4P “Shuttling”

The PtdIns4P Shuttling was performed at 37 °C as described previously using Shuttle PtdIns(4)P Kit (Echelon Biosciences, P-9004). Briefly, the optimized carriers and no-fluorescent PtdIns4P were mixed at a 1:1 molar ratio for 10 minutes at room temperature before adding to culture medium to a final PtdIns4P concentration of 10 μM.

### Cell viability and apoptosis assay

Cell cytotoxicity assay was performed using the cell counting Kit-8 (C0039, Beyotime) according to the standard procedures. Synergy plots and scores were calculated using Synergy Finder (synergyfinder.fimm.fi). Cell apoptosis was examined by flow cytometry according to the manufacturer's instructions (BD Biosciences PharMingen). Cells were washed twice with phosphate buffer saline (PBS) and stained with and 5 μL PI (P4170, Sigma)/2 μL Annexin V-FITC (556419, BD) for 20 minutes at room temperature in the dark, and then analyzed by fluorescence activated cell sorter (FACS).

### Quantitative reverse transcription PCR

RNAs were extracted from cultured cells using TRIzol reagent (Invitrogen) according to the manufacturer's protocol. Gene expression was verified by using AZpolarisTM Cdna Synthesis Kit (Azanno Biotech, Sweden) for reverse-transcription and RealMaster Mix (Tiangen Biotech, China) for reverse transcription PCR, according to the manufacturer's instructions. Primers were shown in **Table S4**.

### LC-MS analysis

The detection system consisted of 100 μg protein, ammonium bicarbonate and 20 mM DTT solution; reacted in 56 °C water for 30 minutes; after cooling to room temperature, added IAA and reacted in the dark for 10 minutes; added ice-cold acetone and then reacted at -20 °C overnight; centrifuged at 4 °C for 15 minutes at maximum speed; discard the supernatant, and evaporated to dryness at room temperature for 10 minutes; added ammonium bicarbonate solution and trypsin, and reacted in 37 °C water overnight. 10 μL peptides were extracted and subjected to LC-MS analyses on LTQ-Orbitrap spectrometer (Thermo Fisher, USA) coupled to an Ultimate 3000 series liquid chromatography system using label free quantification (LFQ). The analysis of MS/MS data was performed using Proteome Discoverer (1.4.0) software against the UniProtKB Homo sapiens (Human) database.

### Transmission electronic microscopy

Cells were treated and collected as indicated and then fixed in 2.5% glutaraldehyde at 4 °C overnight, washed 3 times with PBS, postfixed with 2% osmium tetroxide for 1.5 hours at room temperature. Cells were dehydrated through a series of ethanol concentrations and then embedded and stained with uranyl acetate/lead citrate after fixation. Finally, sections were examined under a transmission electron microscope at 60 kV (JEM-1400PLUS, Japan).

### Gene Set Enrichment Analysis

Raw Data was retrieved from the TCGA database. A Gene Set Enrichment Analysis (GSEA) was performed via GSEA software (version: 4.1.0) to explore molecular pathways involved in leukemia pathogenesis by PI4KA, as previously described 42. The significant gene sets were up to following standards: Nominal p-value < 0.05.

### Molecular docking

Molecular docking were performed as as described previously with minor modifications 43, 44. The structures of the ligands were obtained via ChemBioDraw and subjected to LigPrep (OPLS-2005 force fields) module to generate the 3D structures, as follwed by the minimization of the generated conformations. The crystallographic structure of PI4KA was downloaded from Protein Data Bank (PDB entry: 6BQ1). The docking procedure was ultilized to capture the binding poses using standard precision (SP) docking via Glide implemented in Maestro with default parameters. Simultaneously, the binding free energies of two ligands binding to PI4KA were calculated via Molecular mechanics/generalized born surface area (MM/GBSA) method based on the top 10 binding poses.

### Leukemia-xenograft mice studies

NOD/SCID mice (5 weeks old) were obtained from Yaokang Biotechnology Co., Ltd. (Chengdu, China). Mice were exposed to 2 Gy of irradiation. After 24 hours, K562/Adr or K562/Adr-sgPI4KA cells (1×10^7^) were injected into the tail vein of the mice. Survival time of each group was recorded. The percentage of CD45+ cells from bone marrow of mice was determined by flow cytometry analysis. Spleen and liver were extracted for weight comparison, organs were fixed in formalin. The immunohistochemical analyses and hematoxylin and eosin (H&E) staining were performed for histopathological evaluation and determination of CD45+ expression. Animal studies were performed according to federal guidelines. All animal experiments were carried out under protocols approved by the Army Medical University and Zunyi Medical University Institutional Animal Care and Use Committee.

### Patient data analysis

Patient data and gene expression datasets were obtained from R2: microarray analysis and visualisation platform (http://hgserver1.amc.nl/cgi-bin/r2/main.cgi). The resulting figures and P values were downloaded 45, 46. Download the complete gene expression profiles and clinical information of AML patients in R2, TCGA (https://cancergenome.nih.gov/) and GEO (https://www.ncbi.nlm.nih.gov/geo) (GSE12417). The AML patients were divided into two groups according to the expression values of PI4KA by the X-tile method and X-tile method was used to estimate the correlation between PI4KA expression and overall survival in this study 47-49. The relevant parameters were set as follows: add 95% Confidence Interval, Axis Units = Months. The remaining “n:s” at different time points for both curves and log-rank p-value were assessed by GraphPad Prism 8. The patient information in R2, TCGA and GSE12417 datasets were shown in **Table S1.**

### Generation of PI4KA-overexpressing Cells

PI4KA-overexpressing cells were established using CRISPR/Cas9 based Synergistic Activation Mediator (SAM) system 50 constructed by GeneChem Co. Ltd. (China). K562 or HL-60 cells delivered CRISPR-SAM system were further infected with lentiviruses containing sgRNA targeting to human PI4KA promoter. Oligos were designed using the Cas9 Activator Tool (http://crispr.mit.edu/). Three pairs of oligos were tested to select the target which induces the most efficient upregulation. **Table S4** shows the sgRNA sequence that was used for PI4KA-overexpressing. Lentivirus expressing dCas9-VP64, PI4KA-sgRNA-SV40-MS2-P65-HSF1-CMV-EGFP and negative control (vector) were constructed and provided by GeneChem Co. Ltd. (GCEL0245482). Cells were seeded into 24-well plates for transfection using HitransG viral infection reagent (REVG003, GeneChem Co. Ltd.).

### Statistical analysis

All the experiments were repeated at least three times, values are presented as mean ± standard deviation (SD). The statistical analyses were performed by GraphPad Prism using Student's t-test (independent-sample t test). Survival curves were calculated using the Kaplan-Meier analysis and the difference between survival curves was analyzed by log-rank test. P value<0.05 was considered to be signifcant. Statistical signifcance is represented by: *P<0.05; **P<0.01; ***P<0.001.

## Supplementary Material

Supplementary figures and tables.

## Abbreviations

DOX: Doxorubicin
IMA: Imatinib
DNR: Daunorubicin
PtdIns4P: Phosphatidylinositol-4-phosphate
PI4KA/PI4KIIIα: Phosphatidylinositol 4 kinases IIIα
BM: Bone marrow
ATCC: American Type Culture Collection
SDS-PAGE: Sodium dodecyl sulfate-polyacrylamide gel electrophoresis
MMP: Mitochondrial membrane potential
PBS: Phosphate buffered saline
LC-MS: Liquid Chromatograph Mass Spectromete
SAM: Synergistic Activation Mediator
GSEA: Gene Set Enrichment Analysis
KEGG: Kyoto Encyclopedia of Genes and Genomes
OXPHOS: oxidative phosphorylation
OCR: Oxygen consumption
DEPs: Differentially expressed proteins
PI: Propidium Iodide
PBMC: Peripheral blood mononuclear cell
ZIP: Zero Interaction Potency
