# Supplementary material for: Targeting PI4KA sensitizes refractory leukemia to chemotherapy by modulating the ERK/AMPK/OXPHOS axis
Source: Theranostics. 2022 Oct 3;12(16):6972–88. doi: 10.7150/thno.76563 (PMC9576605; doi:10.7150/thno.76563)
Supplement: Supplementary file 1 — Supplementary figures and tables. [file thnov12p6972s1.pdf]

Supplementary Materials for

Targeting PI4KA sensitizes refractory leukemia to chemotherapy by modulating the ERK/AMPK/OXPHOS axis

Xiuxing Jiang[1], Xiangtao Huang[2], Guoxun Zheng[3], Guanfei Jia[1], Zhiqiang Li[1], Xin Ding[1], Ling Lei[1], Liang Yuan[4], Shuangnian Xu[2] and Ning Gao[1,4]

Correspondence to: gaoning59@tmmu.edu.cn and xushuangnian@tmmu.edu.cn

This PDF file includes:

Figure S1 to S16
Table S1 to S4

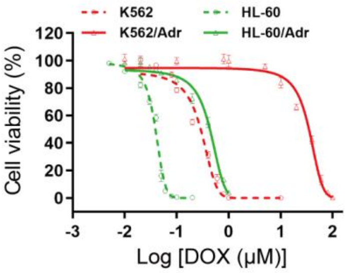

Cell line	IC_{50} (μM)
K562	0.2677 to 0.3101
K562/Adr	32.87 to 36.84
HL-60	0.03772 to 0.04005
HL-60/Adr	0.4103 to 0.4570

Figure S1. Cell viability was detected using CCK-8 assay (n = 3).

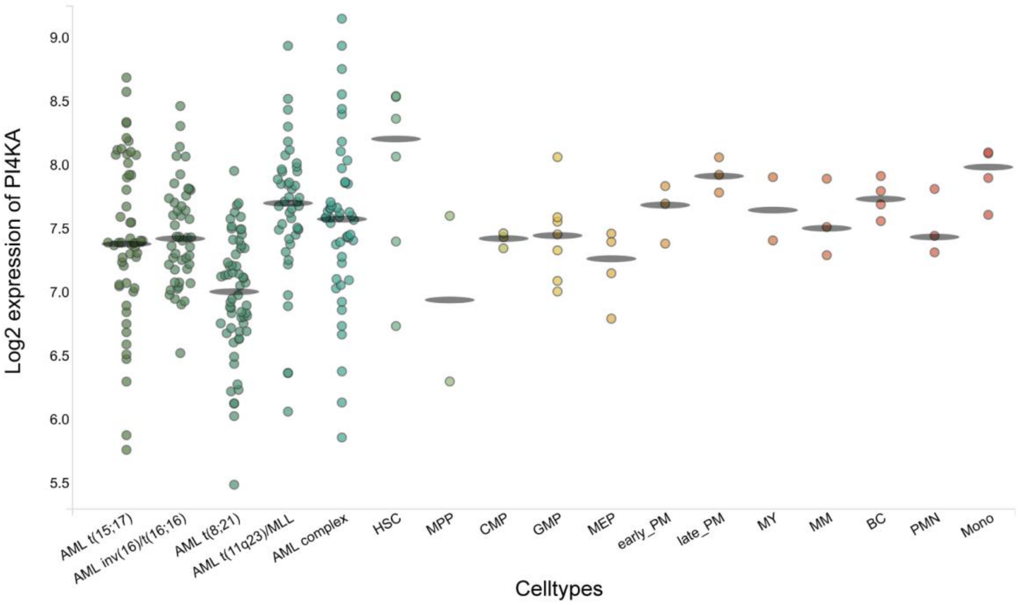

Figure S2. Expression of PI4KA in Bloodspot databases.

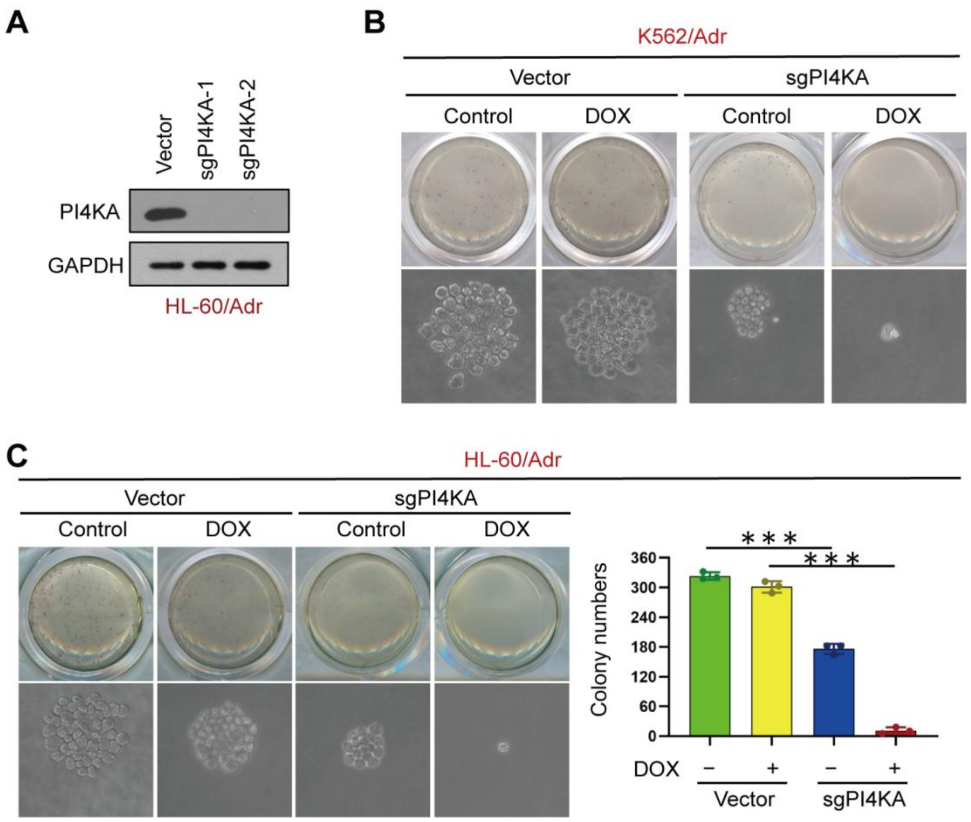

Figure S3. Knockout of PI4KA inhibited colony formation in K562/Adr and Hl-60/Adr cells. **(A)** Knockout of PI4KA by CRISPR/Cas9 system. The protein level of PI4KA was analyzed by Western Blot in HL-60/Adr cells transfected with sgRNA targeting PI4KA (sgPI4KA-1, sgPI4KA-2). **(B, C)** Cell colony was detected by using soft agar assay (n = 3, ***p < 0.001).

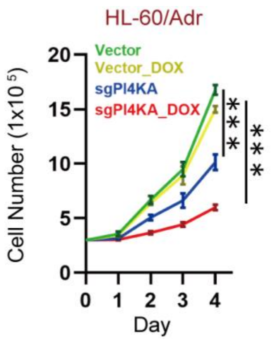

Figure S4. Cell number was detected using Beckman Coulter Z2 Particle Counter (n = 3, ***p < 0.001).

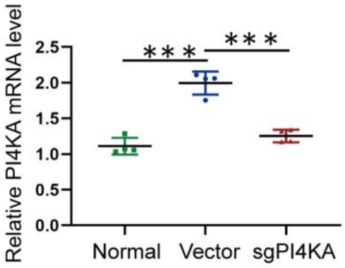

Figure S5. The expression of PI4KA at mRNA levels was determined by qRT-PCR analysis (n = 4, ***p < 0.001).

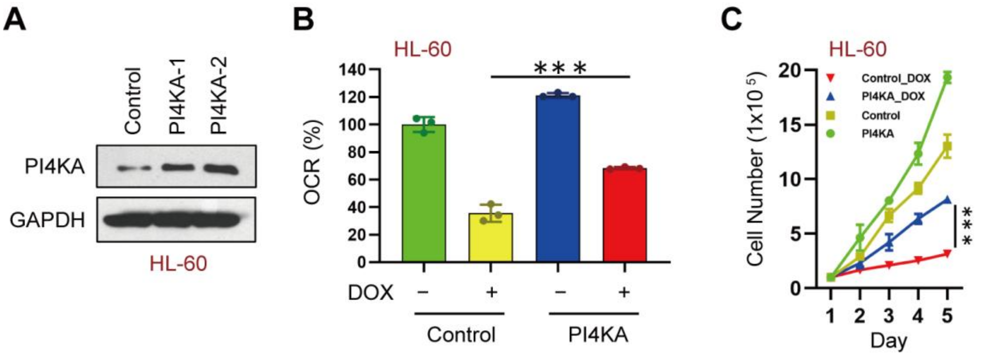

Figure S6. Overexpression of PI4KA decreased sensitivity to DOX in HL-60 cells. (A) The protein level of PI4KA was analyzed by Western Blot in HL-60 cells transfected with dCas9 and guide RNA targeting PI4KA promoter (PI4KA-1, PI4KA-2). **(B)** The levels of OCR was assayed by using commercially available assay kits (n = 3, ***p < 0.001). **(C)** Cells transfected with control or PI4KA plasmid and treated with DOX. Cell number was detected using Beckman Coulter Z2 Particle Counter (n = 3, ***p < 0.001).

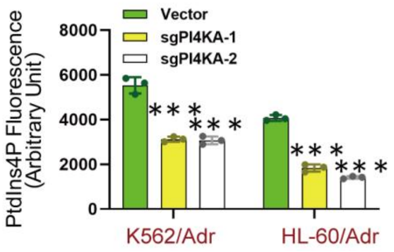

Figure S7. The fluorescence intensity of PtdIns4P (n = 3, ***p < 0.001, compared with vector).

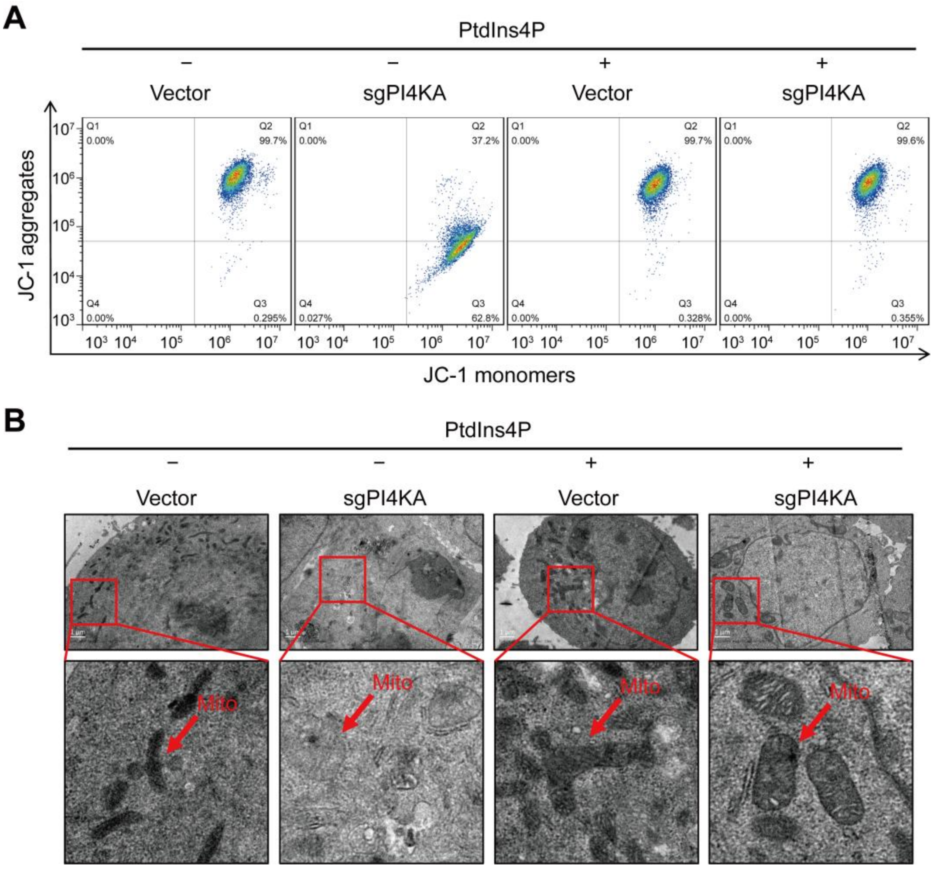

Figure. S8. Mitochondrial membrane potential (A) and transmission electron microscopy observation of mitochondrial morphologies (B) in HL-60/Adr cells. Scar bar, 1 μm.

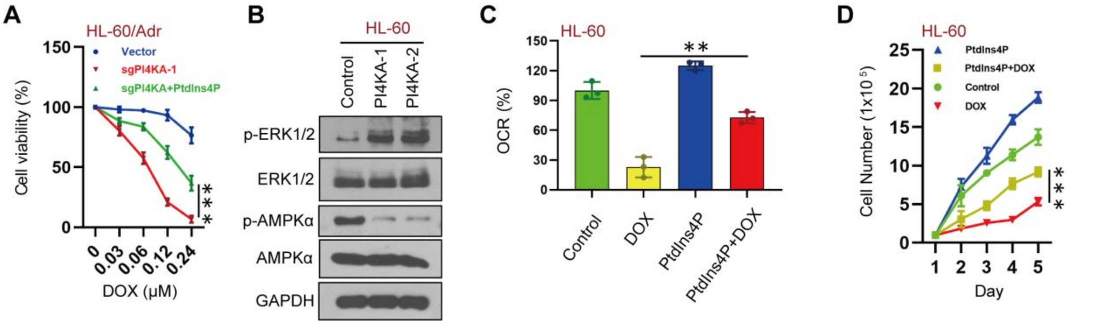

Figure S9. PI4KA regulated OXPHOS via ERK/AMPK signaling. (A) Cell viability was assessed by CCK-8 kits (n = 3, ***p < 0.001). **(B)** The expression of ERK/AMPK signaling pathway related proteins was detected by Western blot. **(C)** The levels of OCR was assessed by using commercially available assay kits (n = 3, **p < 0.01). **(D)** Cells treated with PtdIns4P or DOX. Cell number was detected using Beckman Coulter Z2 Particle Counter (n = 3, ***p < 0.001).

Compounds	Cepharanthine	Rutaecarpine	Solasodine	Gelsemine	Dauricine	Asiatic acid	Polyphyllin VI	Narcissin	Eupalinilide C
IC50-α (μM)	11.33	39.17	13.79	56.86	5.912	65.01	5.294	139.9	41.91
IC50-β (μM)	26.29	31.29	11.75	33.62	1.420	115.6	0.5390	474.7	16.45

Figure S10. $IC_{50\text{-}\alpha}$ values for the inhibition of PI4K and $IC_{50\text{-}\beta}$ values for inhibition of normal PBMC.

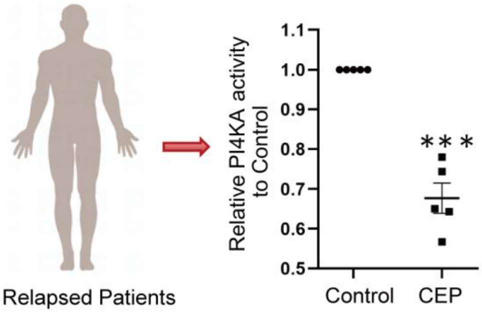

Figure S11. The activity of PI4KA was assayed by ELISA kit in patient samples (n = 5, ***p < 0.001, compared with control).

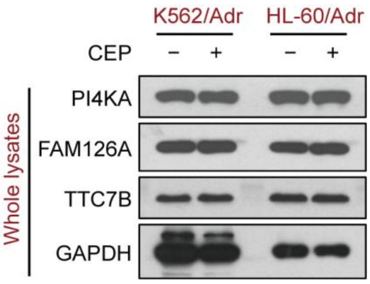

Figure S12. Cells were treated without or with CEP (6 μM), PI4KA and TTC7B, FAM126A proteins were examined by Western blot in whole lysates.

Title	dG_Bind	dG_Coulomb	dG_vdW	dG_Covalent	dG_Hbond	dG_Lipo	dG_Packing	dG_SelfCont	dG_Solv_GB
CEP_1	-45.21	-260.79	-48.91	10.92	1.13	-20.59	-0.63	4.19	269.46
CEP_2	-44.83	-130.28	-32.97	-5.35	-0.97	-13.97	-1.80	0.03	140.48
CEP_3	-44.83	-130.28	-32.97	-5.35	-0.97	-13.97	-1.80	0.03	140.48

Figure S13. The predicted binding free energy (dG_Bind) of CEP binding to PI4KA calculated by MM/GBSA method (dG_Bind: kcal/mol).

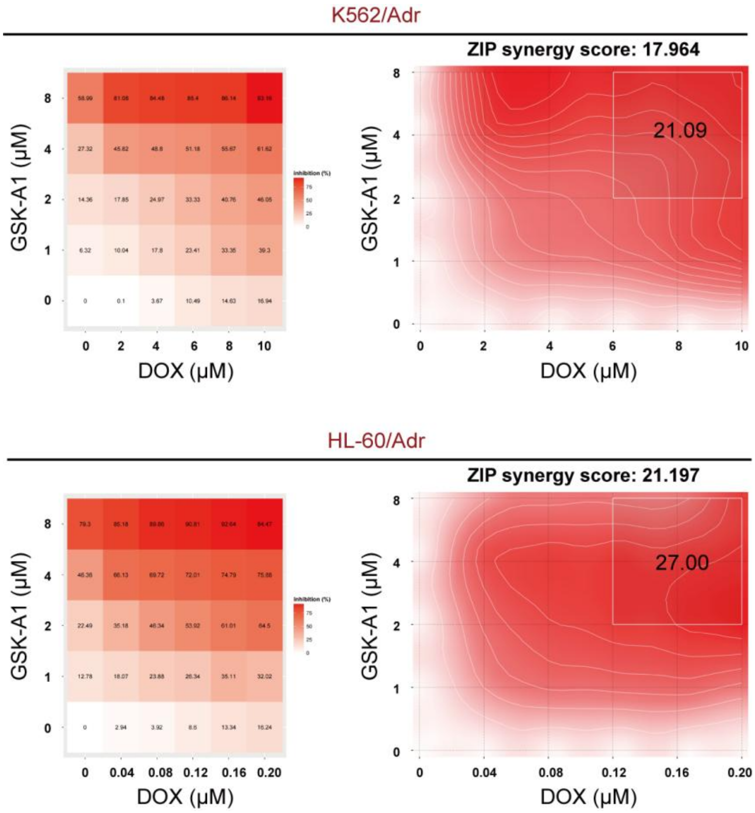

Figure S14. Dose-dependent inhibition matrix and ZIP synergy plot of resistant leukemia cells treated with various concentrations of DOX and GSK-A1 as indicated for 48 hours, value in the white box represents the averaged synergy score for the region of highest synergy.

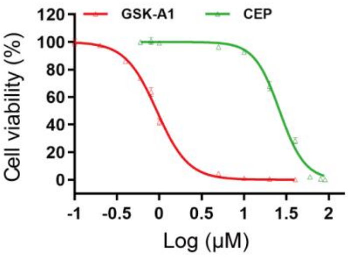

Drug	IC_{50} (μM)
CEP	25.28 to 27.28
GSK-A1	0.9072 to 0.9661

Figure S15. Cell viability was detected using CCK-8 assay in normal PBMC (n = 3)**.**

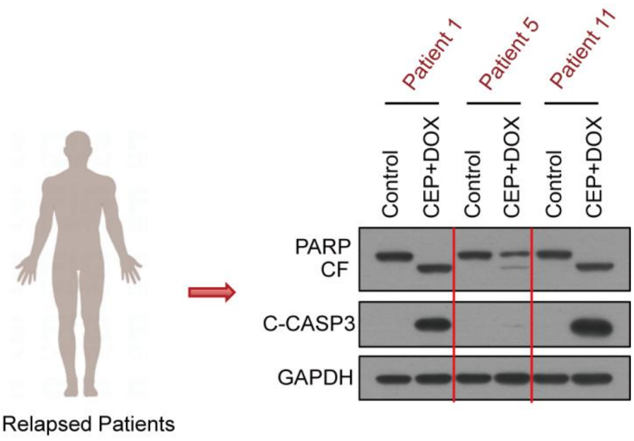

Figure S16. The expression of PARP and cleaved caspase-3 in patient samples were determined by Western blot.

Supplementary Tables

Table S1. The relevant clinical information of patient samples.

Information for 15 patients				
**Patient**	**Gender**	**Age**	**FAB Classification**	**WBC (×10^9/L)**
1	Female	30	M5	1.94
2	Female	70	M2	2.55
3	Female	41	M2	182.92
4	Female	49	M2	63.25
5	Female	55	M2	45.26
6	Female	47	M1	95.09
7	Female	60	M1	18.46
8	Male	49	M2	59.73
9	Female	56	M1	9.54
10	Male	46	M2	17.41
11	Female	53	M2	9.01
12	Male	49	M2	11.22
13	Female	44	M2	2.57
14	Female	53	M4	0.48
15	Female	20	M4	15.17
**Patient**	**Hb (g/L)**	**PLT(× 10^9/L)**	**Recurrence time**	**BM Blasts (%)**
1	62	5	2022.5.26	85
2	109	162	2022.5.11	32.58
3	116	17	2022.5.9	45.4
4	103	51	2022.5.7	91.81
5	73	8	2021.4.27	84.41
6	11	39	2021.10.12	86
7	121	161	2021.9.8	85.8

8	90	52	2021.7.23	62.6
9	133	53	2021.5.20	45.9
10	96	17	2021.4.20	89.9
11	116	167	2021.2.21	72.5
12	58	55	2021.2.21	84
13	65	16	2021.2.22	23.8
14	96	23	2022.5.7	22.1
15	41	62	2022.1.10	74.6

R2

ID	OS	OS_DAYS	PI4KA Expression	Group
GSM923713	1	2	11.28	High
GSM923965	1	1183	11.13	High
GSM923842	1	150	11.11	High
GSM923791	0	1531	11.01	High
GSM923865	0	4027	10.99	High
GSM923972	1	301	10.98	High
GSM923684	1	75	10.96	High
GSM923706	1	19	10.96	High
GSM923820	1	6	10.92	High
GSM923952	0	1436	10.92	High
GSM923688	1	215	10.89	High
GSM923835	1	918	10.89	High
GSM923958	1	4029	10.89	High
GSM923700	1	363	10.86	High
GSM923999	1	70	10.83	High
GSM923853	1	261	10.82	High
GSM923871	1	122	10.82	High
GSM923857	1	249	10.8	High
GSM923719	0	211	10.77	High
GSM923878	1	43	10.76	High
GSM924000	1	676	10.73	High
GSM923711	1	422	10.71	High
GSM923930	1	15	10.71	High
GSM923963	1	2978	10.71	High
GSM923724	1	225	10.7	High
GSM923696	1	239	10.68	High
GSM923917	1	231	10.68	High
GSM923978	1	51	10.68	High
GSM923720	1	192	10.67	High
GSM923695	1	104	10.64	High

GSM923870	1	292	10.64	High
GSM923891	1	124	10.64	High
GSM923923	1	8	10.64	High
GSM923631	1	66	10.61	High
GSM923823	1	8	10.6	High
GSM923967	1	230	10.6	High
GSM923710	1	430	10.59	Low
GSM923811	1	4	10.59	Low
GSM923945	1	4502	10.59	Low
GSM923732	1	2144	10.57	Low
GSM923928	0	3161	10.57	Low
GSM923597	0	3537	10.55	Low
GSM923799	0	2862	10.55	Low
GSM923913	1	873	10.55	Low
GSM923988	1	28	10.55	Low
GSM923821	1	2298	10.54	Low
GSM923872	1	111	10.54	Low
GSM923975	1	35	10.54	Low
GSM923641	0	155	10.53	Low
GSM923730	1	298	10.52	Low
GSM923968	1	52	10.52	Low
GSM923900	0	3642	10.51	Low
GSM923686	1	46	10.5	Low
GSM923854	0	3844	10.49	Low
GSM923718	0	1966	10.48	Low
GSM923826	0	2990	10.48	Low
GSM923969	1	970	10.48	Low
GSM923672	0	619	10.47	Low
GSM923877	1	258	10.47	Low
GSM923605	1	128	10.46	Low
GSM923869	1	62	10.46	Low
GSM923953	1	177	10.46	Low
GSM923781	0	4179	10.45	Low
GSM923882	0	4597	10.45	Low
GSM923918	1	265	10.45	Low
GSM923669	1	152	10.43	Low
GSM923973	1	97	10.43	Low
GSM923622	1	2	10.41	Low
GSM923962	1	149	10.41	Low
GSM923674	1	52	10.4	Low
GSM923685	1	82	10.4	Low
GSM923727	1	208	10.4	Low
GSM923897	1	163	10.4	Low
GSM923738	0	61	10.39	Low

GSM923855	1	626	10.38	Low
GSM923919	0	2812	10.38	Low
GSM923949	1	353	10.38	Low
GSM923979	1	74	10.37	Low
GSM923801	1	516	10.36	Low
GSM923802	0	1903	10.36	Low
GSM923888	1	125	10.36	Low
GSM923976	0	4480	10.36	Low
GSM923995	1	2	10.36	Low
GSM923689	1	369	10.35	Low
GSM923827	0	3648	10.35	Low
GSM923905	1	417	10.35	Low
GSM923966	0	4474	10.35	Low
GSM923848	0	2717	10.33	Low
GSM923908	1	340	10.33	Low
GSM923980	1	366	10.33	Low
GSM923725	1	215	10.32	Low
GSM923816	1	269	10.32	Low
GSM923621	0	2706	10.31	Low
GSM923600	1	30	10.3	Low
GSM923628	1	403	10.3	Low
GSM923859	0	3619	10.3	Low
GSM923860	1	108	10.3	Low
GSM923879	1	623	10.3	Low
GSM923707	1	1493	10.29	Low
GSM923873	1	37	10.29	Low
GSM923649	1	22	10.28	Low
GSM923747	0	3926	10.28	Low
GSM923762	1	1517	10.28	Low
GSM923868	0	4343	10.28	Low
GSM923617	1	420	10.27	Low
GSM923773	1	9	10.27	Low
GSM923844	1	170	10.27	Low
GSM923846	1	770	10.27	Low
GSM923867	1	283	10.27	Low
GSM923937	1	22	10.27	Low
GSM923715	1	257	10.26	Low
GSM923806	1	65	10.26	Low
GSM923931	0	3989	10.26	Low
GSM923861	1	121	10.25	Low
GSM923667	1	24	10.24	Low
GSM923668	1	35	10.24	Low
GSM923874	0	3004	10.23	Low
GSM923940	1	33	10.23	Low

GSM923943	1	305	10.23	Low
GSM923946	1	1008	10.23	Low
GSM923603	0	746	10.22	Low
GSM923716	1	763	10.22	Low
GSM923717	1	296	10.22	Low
GSM923838	1	244	10.22	Low
GSM923906	0	292	10.22	Low
GSM923708	1	463	10.21	Low
GSM923734	1	41	10.21	Low
GSM923901	1	240	10.21	Low
GSM923889	1	322	10.2	Low
GSM923910	1	63	10.2	Low
GSM923659	1	42	10.19	Low
GSM923974	1	1674	10.19	Low
GSM923646	1	35	10.18	Low
GSM923714	1	53	10.18	Low
GSM923942	1	324	10.18	Low
GSM923947	1	1221	10.18	Low
GSM923987	0	299	10.18	Low
GSM923990	1	729	10.18	Low
GSM923840	1	19	10.16	Low
GSM923911	1	97	10.16	Low
GSM923851	0	1426	10.15	Low
GSM923981	1	809	10.15	Low
GSM923770	0	2368	10.14	Low
GSM923776	1	15	10.14	Low
GSM923959	0	4061	10.14	Low
GSM923651	1	80	10.13	Low
GSM923657	0	2170	10.12	Low
GSM923664	1	862	10.12	Low
GSM923782	0	4166	10.12	Low
GSM923998	1	206	10.12	Low
GSM923775	0	3298	10.11	Low
GSM923833	1	61	10.11	Low
GSM923834	1	4	10.11	Low
GSM923977	1	621	10.1	Low
GSM923676	1	29	10.09	Low
GSM923705	0	4501	10.09	Low
GSM923858	1	3696	10.07	Low
GSM923994	1	109	10.07	Low
GSM923642	0	792	10.06	Low
GSM923909	1	794	10.06	Low
GSM923643	1	272	10.05	Low
GSM923650	0	3621	10.05	Low

GSM923698	1	72	10.05	Low
GSM923808	1	5	10.05	Low
GSM923822	0	99	10.05	Low
GSM923856	0	3709	10.05	Low
GSM923803	0	3093	10.04	Low
GSM923961	0	3169	10.04	Low
GSM923598	1	123	10.03	Low
GSM923612	1	83	10.03	Low
GSM923817	1	276	10.03	Low
GSM923924	1	187	10.03	Low
GSM923932	1	374	10.03	Low
GSM923632	1	493	10.02	Low
GSM923804	0	3098	10.02	Low
GSM923819	0	3625	10.02	Low
GSM923830	0	2023	10.02	Low
GSM923991	1	107	10.02	Low
GSM923925	0	3672	10.01	Low
GSM923620	0	892	10	Low
GSM923640	1	2128	10	Low
GSM923647	1	183	10	Low
GSM923777	0	3210	10	Low
GSM923824	1	418	10	Low
GSM923852	0	429	10	Low
GSM923701	1	303	9.99	Low
GSM923797	0	4348	9.99	Low
GSM923788	1	4	9.98	Low
GSM923813	1	2	9.98	Low
GSM923599	0	3395	9.97	Low
GSM923677	1	342	9.97	Low
GSM923778	0	3265	9.97	Low
GSM923845	1	88	9.97	Low
GSM923971	1	107	9.96	Low
GSM923712	1	43	9.95	Low
GSM923767	0	4431	9.95	Low
GSM923795	0	1302	9.95	Low
GSM923875	1	209	9.95	Low
GSM923920	1	273	9.95	Low
GSM923832	1	307	9.94	Low
GSM923944	1	683	9.94	Low
GSM923984	1	507	9.94	Low
GSM923692	1	230	9.93	Low
GSM923887	1	28	9.93	Low
GSM923662	0	3267	9.92	Low
GSM923616	1	294	9.91	Low

GSM923644	1	25	9.9	Low
GSM923805	1	43	9.9	Low
GSM923847	1	9	9.9	Low
GSM923884	1	717	9.9	Low
GSM923655	1	1075	9.89	Low
GSM923653	1	77	9.88	Low
GSM923751	0	3949	9.88	Low
GSM923761	0	3567	9.88	Low
GSM923800	0	3136	9.88	Low
GSM923903	1	177	9.88	Low
GSM923645	1	20	9.86	Low
GSM923623	0	3242	9.85	Low
GSM923675	1	30	9.85	Low
GSM923733	1	329	9.85	Low
GSM923739	1	138	9.85	Low
GSM923780	0	1523	9.85	Low
GSM923948	1	42	9.85	Low
GSM923682	1	254	9.84	Low
GSM923837	1	2423	9.84	Low
GSM923926	0	2968	9.84	Low
GSM923771	0	3580	9.83	Low
GSM923807	0	3564	9.83	Low
GSM923927	0	1954	9.83	Low
GSM923960	0	4362	9.83	Low
GSM923983	1	2035	9.83	Low
GSM923679	1	35	9.82	Low
GSM923828	1	309	9.82	Low
GSM923665	0	1444	9.81	Low
GSM923699	1	6	9.81	Low
GSM923709	0	3155	9.81	Low
GSM923785	1	1491	9.81	Low
GSM923787	0	3549	9.81	Low
GSM923611	1	317	9.8	Low
GSM923760	1	46	9.8	Low
GSM923606	0	1953	9.79	Low
GSM923666	1	19	9.79	Low
GSM923749	1	744	9.79	Low
GSM923810	1	358	9.79	Low
GSM923752	0	3425	9.78	Low
GSM923814	1	50	9.78	Low
GSM923829	0	3252	9.78	Low
GSM923841	0	1549	9.78	Low
GSM924003	1	412	9.78	Low
GSM923862	1	50	9.77	Low

GSM923619	1	595	9.76	Low
GSM923687	1	521	9.76	Low
GSM923763	0	3332	9.76	Low
GSM923996	1	20	9.76	Low
GSM924002	1	840	9.76	Low
GSM923792	0	3826	9.75	Low
GSM923794	0	3649	9.74	Low
GSM923880	1	32	9.74	Low
GSM923890	1	60	9.74	Low
GSM923904	1	259	9.73	Low
GSM924011	1	916	9.73	Low
GSM923637	1	62	9.72	Low
GSM923673	1	35	9.72	Low
GSM923729	0	1039	9.72	Low
GSM923726	1	98	9.71	Low
GSM923764	0	1329	9.71	Low
GSM923864	1	261	9.71	Low
GSM923936	1	119	9.71	Low
GSM923955	1	3163	9.71	Low
GSM923957	1	235	9.71	Low
GSM923755	0	3228	9.7	Low
GSM923866	1	466	9.7	Low
GSM923954	1	562	9.7	Low
GSM923627	1	33	9.69	Low
GSM923786	0	3711	9.69	Low
GSM923997	1	47	9.69	Low
GSM924009	1	323	9.69	Low
GSM923624	0	3141	9.68	Low
GSM923753	0	3785	9.68	Low
GSM923843	1	2432	9.68	Low
GSM923793	1	14	9.67	Low
GSM923935	1	1391	9.66	Low
GSM924010	1	303	9.66	Low
GSM923639	1	44	9.65	Low
GSM923663	1	648	9.65	Low
GSM923671	1	28	9.65	Low
GSM923722	1	187	9.65	Low
GSM923894	1	43	9.65	Low
GSM923985	1	813	9.65	Low
GSM923633	1	658	9.64	Low
GSM923693	1	63	9.64	Low
GSM923769	1	5	9.64	Low
GSM923784	1	1367	9.64	Low
GSM923885	1	237	9.64	Low

GSM923964	0	5023	9.64	Low
GSM923661	1	1902	9.63	Low
GSM924017	1	231	9.63	Low
GSM923635	0	393	9.62	Low
GSM923892	1	257	9.62	Low
GSM923915	1	316	9.62	Low
GSM923704	1	568	9.61	Low
GSM923728	1	40	9.61	Low
GSM923796	1	33	9.61	Low
GSM923754	1	53	9.6	Low
GSM923766	1	23	9.6	Low
GSM923638	1	657	9.59	Low
GSM923876	0	4214	9.59	Low
GSM923608	1	43	9.58	Low
GSM923736	1	99	9.58	Low
GSM923694	1	263	9.57	Low
GSM923951	1	194	9.57	Low
GSM923614	1	173	9.56	Low
GSM923812	1	345	9.56	Low
GSM923916	1	190	9.56	Low
GSM923912	1	34	9.54	Low
GSM923654	1	338	9.52	Low
GSM923731	0	657	9.52	Low
GSM923934	0	2968	9.52	Low
GSM923610	1	1	9.51	Low
GSM923626	0	3117	9.51	Low
GSM923681	1	184	9.51	Low
GSM924015	1	706	9.51	Low
GSM923779	1	336	9.5	Low
GSM923596	1	186	9.49	Low
GSM923656	0	542	9.49	Low
GSM923625	1	413	9.48	Low
GSM924005	1	506	9.48	Low
GSM923613	1	25	9.47	Low
GSM923757	0	2831	9.47	Low
GSM924013	1	42	9.47	Low
GSM923607	1	84	9.45	Low
GSM923660	1	393	9.44	Low
GSM923825	1	22	9.44	Low
GSM923774	1	50	9.42	Low
GSM923839	1	77	9.42	Low
GSM923772	0	1637	9.41	Low
GSM923601	1	29	9.4	Low
GSM923615	1	352	9.4	Low

GSM923815	1	4	9.4	Low
GSM923982	1	62	9.4	Low
GSM923836	1	1104	9.39	Low
GSM923721	0	2664	9.38	Low
GSM923798	1	1422	9.38	Low
GSM923895	1	40	9.38	Low
GSM923636	1	519	9.37	Low
GSM923789	0	3936	9.37	Low
GSM923921	1	489	9.36	Low
GSM923938	0	1222	9.36	Low
GSM923818	1	76	9.35	Low
GSM923886	0	4415	9.35	Low
GSM923648	1	47	9.34	Low
GSM923783	1	764	9.34	Low
GSM923956	1	646	9.34	Low
GSM923683	1	118	9.33	Low
GSM923765	1	1161	9.33	Low
GSM923899	1	602	9.33	Low
GSM924006	1	152	9.33	Low
GSM923602	1	827	9.31	Low
GSM923634	0	68	9.31	Low
GSM923933	0	3024	9.29	Low
GSM923742	0	4083	9.28	Low
GSM924007	1	13	9.27	Low
GSM923697	1	372	9.25	Low
GSM923741	1	74	9.25	Low
GSM923831	1	287	9.25	Low
GSM924001	1	122	9.25	Low
GSM923896	1	52	9.24	Low
GSM923907	1	31	9.24	Low
GSM924016	1	242	9.24	Low
GSM923914	1	253	9.23	Low
GSM923630	1	434	9.21	Low
GSM923744	1	27	9.21	Low
GSM924004	1	255	9.21	Low
GSM923703	1	547	9.2	Low
GSM923609	1	493	9.19	Low
GSM923809	1	650	9.19	Low
GSM923658	1	46	9.18	Low
GSM923898	0	3852	9.17	Low
GSM923993	1	620	9.17	Low
GSM923680	1	146	9.16	Low
GSM923743	1	876	9.15	Low
GSM923750	1	24	9.15	Low

GSM923745	1	225	9.12	Low
GSM923902	1	110	9.11	Low
GSM923992	1	22	9.11	Low
GSM923941	1	149	9.1	Low
GSM924008	1	139	9.08	Low
GSM923629	1	425	9.06	Low
GSM923790	0	3724	9.03	Low
GSM923758	0	2717	9	Low
GSM923922	1	20	9	Low
GSM923970	0	4588	9	Low
GSM923748	0	1556	8.98	Low
GSM923740	0	4484	8.96	Low
GSM923618	0	3379	8.89	Low
GSM923746	1	76	8.88	Low
GSM924014	0	316	8.86	Low
GSM923604	1	232	8.85	Low
GSM923652	0	1437	8.83	Low
GSM923939	1	695	8.82	Low
GSM923863	1	308	8.8	Low
GSM923881	1	100	8.8	Low
GSM923768	1	17	8.77	Low
GSM923989	1	113	8.76	Low
GSM923893	1	444	8.75	Low
GSM923691	1	521	8.74	Low
GSM923850	1	252	8.71	Low
GSM923678	1	194	8.69	Low
GSM924012	1	619	8.62	Low
GSM923735	1	1935	8.55	Low
GSM923849	1	611	8.4	Low
GSM923690	1	348	8.15	Low
GSM923883	1	595	8.15	Low
GSM923950	1	324	8.14	Low
GSM923759	1	65	8.11	Low
GSM923756	0	566	7.85	Low
GSM923986	1	289	7.32	Low

TCGA

ID	OS	OS_DAYS	PI4KA Expression	Group
TCGA-AB-2835-03A	0	1673	20.08	High
TCGA-AB-3011-03A	0	1885	19.92	High
TCGA-AB-3002-03A	1	1431	19.9	High
TCGA-AB-2928-03A	1	59	19.83	High
TCGA-AB-2873-03A	0	273	19.77	High

TCGA-AB-2882-03A	1	365	19.71	High
TCGA-AB-2900-03A	1	184	19.7	High
TCGA-AB-2936-03A	0	59	19.66	High
TCGA-AB-2810-03A	1	31	19.65	High
TCGA-AB-2825-03A	1	212	19.63	High
TCGA-AB-2920-03B	1	366	19.61	High
TCGA-AB-2885-03A	1	214	19.58	High
TCGA-AB-2884-03A	1	731	19.58	High
TCGA-AB-2979-03B	0	671	19.58	High
TCGA-AB-2929-03A	1	123	19.54	High
TCGA-AB-2919-03A	0	61	19.54	High
TCGA-AB-2939-03A	0	455	19.52	High
TCGA-AB-2908-03A	1	31	19.5	High
TCGA-AB-2851-03A	1	242	19.5	High
TCGA-AB-2836-03A	1	518	19.5	High
TCGA-AB-2956-03A	1	183	19.48	High
TCGA-AB-2966-03A	1	854	19.48	High
TCGA-AB-2973-03A	1	609	19.47	High
TCGA-AB-2934-03A	0	28	19.46	High
TCGA-AB-2924-03A	0	90	19.44	High
TCGA-AB-2949-03B	0	699	19.43	High
TCGA-AB-3012-03A	0	1887	19.4	High
TCGA-AB-2991-03A	0	1826	19.39	High
TCGA-AB-2925-03A	1	243	19.38	High
TCGA-AB-2847-03A	1	608	19.38	High
TCGA-AB-3001-03A	0	1581	19.37	High
TCGA-AB-2952-03B	1	31	19.36	High
TCGA-AB-2874-03A	0	396	19.36	High
TCGA-AB-3008-03A	1	822	19.36	High
TCGA-AB-2898-03A	0	393	19.35	High
TCGA-AB-2811-03B	1	243	19.34	High
TCGA-AB-2901-03A	0	59	19.33	High
TCGA-AB-2880-03A	1	424	19.32	High
TCGA-AB-2971-03A	1	792	19.31	High
TCGA-AB-2817-03A	1	273	19.3	High
TCGA-AB-2821-03A	1	822	19.3	High
TCGA-AB-2981-03B	0	487	19.3	High
TCGA-AB-2994-03A	0	1798	19.3	High
TCGA-AB-2822-03A	1	973	19.29	High
TCGA-AB-2870-03A	1	153	19.28	High
TCGA-AB-2866-03A	1	153	19.28	High
TCGA-AB-2867-03A	1	184	19.28	High
TCGA-AB-2830-03A	1	275	19.27	High
TCGA-AB-2893-03A	1	212	19.26	High

TCGA-AB-2992-03A	1	1706	19.26	High
TCGA-AB-2927-03A	1	90	19.23	High
TCGA-AB-2948-03A	1	580	19.23	High
TCGA-AB-2877-03A	0	638	19.23	High
TCGA-AB-2983-03A	1	335	19.22	High
TCGA-AB-2895-03A	1	153	19.22	High
TCGA-AB-2959-03A	1	489	19.2	High
TCGA-AB-2846-03A	1	1402	19.2	High
TCGA-AB-3000-03A	0	1249	19.2	High
TCGA-AB-2970-03A	1	305	19.18	High
TCGA-AB-2896-03B	1	214	19.18	High
TCGA-AB-2990-03B	0	457	19.17	High
TCGA-AB-2933-03A	1	122	19.16	High
TCGA-AB-2872-03A	0	638	19.16	High
TCGA-AB-3009-03A	1	577	19.16	High
TCGA-AB-2813-03A	1	31	19.13	High
TCGA-AB-3007-03A	0	1581	19.12	High
TCGA-AB-2899-03A	1	671	19.11	High
TCGA-AB-2871-03A	0	153	19.11	High
TCGA-AB-2869-03A	0	243	19.1	High
TCGA-AB-2818-03A	1	303	19.1	High
TCGA-AB-2955-03A	1	489	19.09	High
TCGA-AB-2911-03A	0	1186	19.08	High
TCGA-AB-2849-03A	0	2220	19.08	High
TCGA-AB-2845-03B	1	304	19.07	High
TCGA-AB-2826-03A	1	731	19.06	High
TCGA-AB-2859-03A	1	304	19.05	High
TCGA-AB-2865-03A	1	61	19.04	High
TCGA-AB-2844-03A	1	122	19.03	High
TCGA-AB-2814-03A	1	792	19.03	High
TCGA-AB-2862-03A	0	1430	19.03	High
TCGA-AB-2998-03A	1	31	19.02	High
TCGA-AB-2843-03A	1	215	19.01	High
TCGA-AB-2839-03A	1	486	19	High
TCGA-AB-2987-03A	1	184	18.99	High
TCGA-AB-2913-03A	0	1216	18.98	High
TCGA-AB-2888-03B	0	334	18.98	High
TCGA-AB-2856-03A	1	150	18.97	High
TCGA-AB-2976-03A	1	915	18.97	High
TCGA-AB-2881-03A	0	393	18.95	High
TCGA-AB-2965-03A	1	335	18.94	High
TCGA-AB-2897-03A	0	243	18.94	High
TCGA-AB-2857-03A	1	306	18.93	High
TCGA-AB-2841-03B	1	1401	18.93	High

TCGA-AB-2840-03A	1	28	18.91	High
TCGA-AB-2876-03A	0	1219	18.9	Low
TCGA-AB-2916-03A	0	882	18.89	Low
TCGA-AB-2996-03A	0	1581	18.88	Low
TCGA-AB-2889-03A	0	304	18.88	Low
TCGA-AB-2812-03A	1	366	18.87	Low
TCGA-AB-2828-03A	0	2284	18.86	Low
TCGA-AB-2988-03B	1	30	18.85	Low
TCGA-AB-2942-03A	0	641	18.83	Low
TCGA-AB-2834-03A	1	245	18.83	Low
TCGA-AB-2995-03A	0	1551	18.81	Low
TCGA-AB-2886-03A	0	181	18.8	Low
TCGA-AB-2982-03B	0	150	18.79	Low
TCGA-AB-2815-03A	1	822	18.78	Low
TCGA-AB-2917-03A	0	1216	18.78	Low
TCGA-AB-2883-03A	0	730	18.77	Low
TCGA-AB-2875-03A	0	212	18.77	Low
TCGA-AB-2963-03A	1	1642	18.76	Low
TCGA-AB-2805-03A	1	577	18.72	Low
TCGA-AB-2894-03A	1	181	18.72	Low
TCGA-AB-2935-03A	1	61	18.64	Low
TCGA-AB-2863-03A	1	31	18.63	Low
TCGA-AB-2892-03A	0	943	18.62	Low
TCGA-AB-2914-03A	0	792	18.62	Low
TCGA-AB-2912-03A	1	274	18.59	Low
TCGA-AB-2937-03A	1	215	18.59	Low
TCGA-AB-2980-03A	0	699	18.56	Low
TCGA-AB-2999-03B	0	1735	18.55	Low
TCGA-AB-2977-03B	1	31	18.51	Low
TCGA-AB-2806-03A	1	945	18.51	Low
TCGA-AB-2858-03A	1	577	18.5	Low
TCGA-AB-2808-03A	0	2861	18.5	Low
TCGA-AB-2853-03A	1	89	18.49	Low
TCGA-AB-2878-03A	1	365	18.43	Low
TCGA-AB-2950-03A	0	306	18.35	Low
TCGA-AB-2938-03A	1	304	18.3	Low
TCGA-AB-2984-03A	0	1157	17.91	Low
TCGA-AB-2986-03A	1	212	17.9	Low
TCGA-AB-2819-03A	0	2496	17.72	Low

GSE12417

ID	OS	OS_DAYS	PI4KA Expression	Group

GSM316658	0	1056	1686.714403	High
GSM316730	1	40	1541.372669	High
GSM316729	1	210	1499.223753	High
GSM316728	1	539	1488.867858	High
GSM316727	0	1441	1428.217514	High
GSM316724	0	1405	1341.842846	High
GSM316725	0	1518	1341.842846	High
GSM316726	0	406	1341.842846	High
GSM316723	1	72	1278.290482	High
GSM316722	1	113	1260.691879	High
GSM316720	1	4	1243.335562	High
GSM316721	1	256	1243.335562	High
GSM316719	1	1118	1217.748086	High
GSM316718	0	1183	1192.687193	High
GSM316717	1	1278	1160.073099	High
GSM316716	1	396	1152.059888	High
GSM316715	0	1168	1136.199139	High
GSM316713	1	14	1067.484939	High
GSM316714	0	1508	1067.484939	High
GSM316711	1	114	1060.111282	High
GSM316712	0	1080	1060.111282	High
GSM316710	1	1	1038.294507	High
GSM316709	1	624	1031.122483	High
GSM316707	0	1085	1009.902289	High
GSM316708	1	606	1009.902289	High
GSM316706	1	132	989.1188008	High
GSM316704	1	301	982.2864582	High
GSM316705	1	403	982.2864582	High
GSM316703	0	1188	975.50131	High
GSM316702	1	271	962.0712952	High
GSM316701	1	21	955.4257833	High
GSM316700	0	1427	948.8261754	High
GSM316698	1	4	942.2721542	High
GSM316699	0	241	942.2721542	High
GSM316697	1	2	929.299615	High
GSM316696	0	1175	903.8878682	High
GSM316694	1	303	891.4437768	High
GSM316695	1	500	891.4437768	High
GSM316693	1	33	885.2861249	High
GSM316692	1	52	867.0671999	High
GSM316690	1	473	861.0779292	High
GSM316691	0	1019	861.0779292	High
GSM316689	0	457	855.1300295	High
GSM316688	1	308	849.2232149	High

GSM316687	1	787	831.7464539	High
GSM316686	1	388	809.0023033	High
GSM316685	1	259	803.4141162	High
GSM316684	1	33	797.8645296	High
GSM316683	1	5	792.3532767	High
GSM316682	1	36	776.0468821	High
GSM316681	0	1092	770.6863347	High
GSM316680	1	96	760.0760682	High
GSM316679	1	624	754.8258393	High
GSM316677	0	1448	739.2917481	Low
GSM316678	1	538	739.2917481	Low
GSM316674	0	1202	699.4126115	Low
GSM316675	0	937	699.4126115	Low
GSM316676	0	919	699.4126115	Low
GSM316673	0	141	680.2871368	Low
GSM316672	1	330	675.5880503	Low
GSM316671	0	1173	670.9214228	Low
GSM316670	1	169	661.6846493	Low
GSM316668	1	253	652.5750412	Low
GSM316669	0	448	652.5750412	Low
GSM316667	0	1300	648.0673761	Low
GSM316666	1	516	634.7303424	Low
GSM316665	0	979	608.8740429	Low
GSM316663	0	1133	544.9575334	Low
GSM316664	1	342	544.9575334	Low
GSM316662	0	1383	512	Low
GSM316661	0	1374	504.9511447	Low
GSM316660	0	1280	487.750655	Low
GSM316659	1	74	481.0356476	Low
GSM316657	1	36	458.2528363	Low
GSM316656	1	471	455.0874528	Low
GSM316655	1	398	404.5011517	Low
GSM316654	0	1403	352.1387054	Low
GSM316653	1	815	326.2875206	Low
GSM316652	0	1304	306.5545484	Low

Table S2. All the DEPS between K562 and K562/Adr cells.

ID	logFC	AveExpr	t	P.Value	adj.P.Val	B
GRHPR	24.84572868	12.42286434	125.0229461	2.86E-09	4.23E-07	8.332256939
SQSTM1	24.54995485	12.27497742	189.5651435	4.25E-10	1.11E-07	8.468233918
SYMPK	23.42032846	11.71016423	72.58633632	3.45E-08	3.64E-06	7.905090596
NTPCR	23.38034759	11.69017379	187.0911641	4.52E-10	1.11E-07	8.465379171
GTPBP1	23.26981277	11.63490638	38.3989777	6.34E-07	5.63E-05	6.683222111
RNF114	23.13582293	11.56791147	120.5034147	3.39E-09	4.70E-07	8.314368407

FDX1	22.89932325	11.44966162	82.50638	1.92E-08	2.13E-06	8.043447776
MBD3	22.73843137	11.36921568	247.0407988	1.26E-10	7.02E-08	8.51270441
PDLIM4	22.6031044	11.3015522	69.33306582	4.25E-08	4.29E-06	7.848325362
EXOSC2	22.44671618	11.22335809	112.0639458	4.72E-09	6.17E-07	8.275389032
AAMP	22.42291605	11.21145803	252.5866498	1.14E-10	7.02E-08	8.515493844
AP3D1	22.33459352	11.16729676	104.9397091	6.38E-09	7.87E-07	8.235476291
PI4KA	22.32596841	11.1629842	68.54735522	4.48E-08	4.32E-06	7.833581547
C8orf82	22.2708634	11.1354317	164.3819163	8.17E-10	1.65E-07	8.43306654
CTH	22.25428443	11.12714221	58.98620888	8.91E-08	8.24E-06	7.613875146
CUL4A	21.87216447	10.93608224	213.0890589	2.49E-10	1.10E-07	8.490705588
RB1	21.80721702	10.90360851	139.4641308	1.73E-09	2.96E-07	8.378762644
ZNF598	19.85823284	12.24274571	9.147612993	0.00041068	0.013018565	0.626041249
MYBBP1A	19.46937789	11.96626157	9.199331758	0.000400665	0.012885146	0.654142987
AK3	18.48313258	14.86176927	3.514598404	0.01966648	0.118265364	-3.846913336
TPD52L1	18.1304532	13.64292844	4.237347512	0.009927423	0.079881106	-3.060113522
GSN	17.76155561	14.57248589	3.332303744	0.023664994	0.130556347	-4.058158597
NDUFAF2	17.58748842	14.48545229	3.302493284	0.024404507	0.132729416	-4.093186574
NDUFB6	17.06359781	16.10526734	2.410550903	0.065446129	0.239250347	-5.19675231
IER3IP1	16.9838339	15.79288324	2.488731853	0.059729815	0.228518032	-5.096492358
STOM	16.90925283	15.6364548	2.517474154	0.057769071	0.22439954	-5.059753626
MGST1	16.8505038	15.89041348	2.415102931	0.065097227	0.239156865	-5.1909026
EPB41L2	16.70169921	15.25870246	2.586896315	0.053321593	0.217901685	-4.971317908
MTOR	16.59547135	8.297735674	3.697424566	0.016420825	0.108206126	-3.640220683
ACOX1	16.59346799	15.66061708	2.410905446	0.06541888	0.239250347	-5.196296645
VMP1	16.53588716	16.12758777	2.250595478	0.079111588	0.257755316	-5.403056518
PGLS	16.45531365	16.3779648	2.158034321	0.088419997	0.261829082	-5.522880133
SDCBP	16.41827025	15.75714013	2.32572876	0.072338689	0.252008826	-5.305991015
GAR1	16.18995096	8.094975478	2.138153173	0.090570107	0.261829082	-5.548634134
SPTLC2	16.16244334	14.93005348	2.524472716	0.057302525	0.22439954	-5.050818607
TACO1	16.15441127	15.33177462	2.380843559	0.067774344	0.244072348	-5.23496206
TP53RK	15.93180097	15.01047143	2.419562668	0.064757386	0.238302886	-5.18517286
CPSF2	15.90795335	15.15383133	2.362977202	0.069218345	0.246541745	-5.257968389
ENSA	15.87444033	16.22042898	2.043934904	0.10155286	0.268435569	-5.6706625
NDUFB1	15.84396988	15.64745091	2.193739192	0.084695746	0.261829082	-5.476638332
AK6	15.8267774	15.14453529	2.33261923	0.071750032	0.251312478	-5.297101886
QKI	15.78602616	15.91870865	2.104326477	0.094358946	0.263575798	-5.592454906
NIBAN2	15.77187729	15.39131414	2.24700326	0.079452292	0.257755316	-5.407702795
MTHFD1L	15.77050093	15.40197383	2.244492056	0.079691416	0.25791477	-5.410951103
MVK	15.76614567	15.14696186	2.320875822	0.072756453	0.252008826	-5.312253036
GTF2F1	15.74101386	15.32284356	2.260040974	0.078223296	0.257646807	-5.390841502
PRAME	15.65467998	15.38932829	2.214527968	0.082605845	0.260003361	-5.449724633
MRPS16	15.65267249	15.66361137	2.136883005	0.090709377	0.261829082	-5.550279574
TOMM34	15.64306135	15.44100094	2.196457771	0.084419218	0.261829082	-5.473118282
PPP4R2	15.63421488	15.02686773	2.317118875	0.073081682	0.252008826	-5.317101635

OSTF1	15.62055536	7.810277679	2.139964054	0.090371949	0.261829082	-5.546288235
AGPAT1	15.51294367	15.15665615	2.242032977	0.079926335	0.25791477	-5.414132179
F11R	15.4509883	14.97048625	2.279664527	0.076412368	0.256131486	-5.365474382
NCAPH	15.44477256	15.13839878	2.227170825	0.081362263	0.259574428	-5.433361763
GCLM	15.43759692	15.47129382	2.130599232	0.091401774	0.261829082	-5.558419952
RAP1GDS1	15.40130024	14.99983604	2.257061937	0.078502278	0.257646807	-5.394693695
MRPL15	15.38480552	15.24830105	2.178254749	0.086289612	0.261829082	-5.496690233
LACTB2	15.35725197	7.678625987	2.138617216	0.090519284	0.261829082	-5.54803299
IMPA1	15.35275571	14.78764943	2.31001514	0.073700991	0.252770479	-5.326271346
NUCB2	15.33941508	14.88446925	2.27488727	0.07684898	0.257071048	-5.371648536
TM9SF4	15.32825269	14.68287582	2.33548895	0.07150642	0.251064472	-5.293400491
CRK	15.31170744	14.85958341	2.270393801	0.07726214	0.257424457	-5.377456729
RNF20	15.29678386	15.27461445	2.146013598	0.089713349	0.261829082	-5.538451477
MEPCE	15.2678824	15.13006505	2.178944282	0.086217949	0.261829082	-5.495797227
CSTF3	15.23402608	14.83177475	2.258912951	0.078328806	0.257646807	-5.392300112
RGS10	15.22642978	14.75258088	2.280956205	0.076294782	0.256131486	-5.363805164
DHPS	15.20710391	14.70030547	2.290015577	0.075475604	0.255305434	-5.352099775
ADGRA3	15.20227005	15.25831998	2.124314227	0.092100004	0.261829082	-5.566561971
DDRGK1	15.19376641	15.08063164	2.172120811	0.086929948	0.261829082	-5.504634512
UQCR11	15.17915741	14.95163689	2.204832094	0.083573531	0.261533105	-5.462276022
PPIP5K2	15.1608054	14.9739553	2.193240093	0.08474662	0.261829082	-5.477284587
ATG4B	15.15137454	14.78544756	2.24847682	0.079312339	0.257755316	-5.405796799
RPS6KA1	15.14487448	14.99864756	2.173947778	0.086738691	0.261829082	-5.50226829
SPCS1	15.09188029	15.00037965	2.157542652	0.0884725	0.261829082	-5.523517007
NOL9	15.07974523	14.87811142	2.198433031	0.084218912	0.261829082	-5.47056078
LSS	15.07535248	14.57446802	2.29201245	0.075296335	0.255087889	-5.349520125
DTYMK	15.04735546	14.7491018	2.227962055	0.081285114	0.259574428	-5.432337861
NUP50	15.02252181	15.00638924	2.14441984	0.089886354	0.261829082	-5.540516061
PRKACA	15.01797148	15.09566591	2.117130009	0.09290517	0.261829082	-5.575868851
POLDIP3	15.01088632	14.6909756	2.235076396	0.080594995	0.259188832	-5.423132241
SLK	15.00846185	14.96671154	2.151200117	0.089152826	0.261829082	-5.531732864
MMP24OS	15.00607274	7.503036369	2.139730963	0.090397429	0.261829082	-5.546590191
ANKHD1	14.99940777	15.04106592	2.128125104	0.091675954	0.261829082	-5.561625104
PPP1R7	14.97755981	14.63692053	2.241732965	0.079955047	0.25791477	-5.414520289
TMED5	14.97557277	7.487786386	2.139271455	0.090447682	0.261829082	-5.547185459
PDLIM7	14.96131882	14.7466169	2.202831056	0.083774769	0.261533105	-5.464866641
MRPL39	14.93906446	14.79559889	2.181085594	0.085995815	0.261829082	-5.493024084
SRSF10	14.93537938	14.92856894	2.140291697	0.090336146	0.261829082	-5.545863792
SCCPDH	14.89204833	14.9643271	2.118838983	0.092712954	0.261829082	-5.573654949
ANKMY2	14.81791498	14.72729345	2.16408445	0.087776669	0.261829082	-5.515043402
RUFY1	14.80832792	7.404163959	2.139248807	0.09045016	0.261829082	-5.547214797
HIBADH	14.752162	14.79997633	2.125697568	0.091945831	0.261829082	-5.564769898
ARIH1	14.73525686	7.36762843	2.139966207	0.090371713	0.261829082	-5.546285445
CUL4B	14.7324847	14.76293012	2.130662727	0.091394749	0.261829082	-5.558337698

P4HA1	14.71391499	14.42575101	2.226559526	0.081421921	0.259574428	-5.434152833
NDRG3	14.67009894	7.33504947	2.138692206	0.090511074	0.261829082	-5.547935845
PES1	14.66310345	14.92687002	2.06564638	0.098901158	0.265979027	-5.642552991
OSBPL3	14.65823011	7.329115054	2.139671174	0.090403966	0.261829082	-5.546667645
SMARCA4	14.62792113	14.93469024	2.053421793	0.100384961	0.267091401	-5.658381254
MCMBP	14.58597987	14.7331453	2.097381394	0.095157734	0.263614246	-5.601451347
NDUFB4	14.58499163	14.74597137	2.087121528	0.096351082	0.265120257	-5.614740856
GID8	14.4982795	14.92838913	2.019230765	0.104662679	0.271706248	-5.702631775
PLCB3	14.49598999	7.247994997	2.139958989	0.090372502	0.261829082	-5.546294796
SSBP3	14.47358521	14.41862099	2.154796269	0.088766398	0.261829082	-5.527074519
APOOL	14.35303002	7.17651501	2.139688714	0.090402048	0.261829082	-5.546644922
MRE11	14.33439456	15.02035062	1.952593738	0.113566987	0.283237697	-5.788748877
CPSF3	14.3086789	14.89747687	1.976485466	0.110285436	0.278882146	-5.757896434
PNPO	14.26737996	14.70464957	2.015468464	0.105145123	0.271706248	-5.70749884
NOP2	14.20050719	7.100253595	2.139866705	0.090382589	0.261829082	-5.546414345
USP48	14.1147893	7.05739465	2.137178151	0.090676995	0.261829082	-5.549897227
PTPN23	13.99059005	6.995295024	2.139959595	0.090372436	0.261829082	-5.546294011
SLC25A24	13.76919256	14.43024878	1.951015154	0.113787414	0.283237697	-5.790786282
CLN6	12.50305069	8.456691348	1.742019476	0.14732618	0.342800903	-6.058664815
RRP12	12.47064372	8.507867555	1.729725567	0.149593578	0.346319292	-6.074264959
PML	12.33509788	8.497355723	1.714667273	0.152419667	0.349400043	-6.093341789
SART1	12.19229475	15.53367581	2.06336225	0.09917661	0.265979027	-5.645510685
ABCB1	10.94518291	22.05686883	1.412322587	0.22205214	0.449759711	-6.466369044
SLC38A2	10.92029366	12.60464272	1.286094201	0.259580986	0.497848062	-6.614133295
COPS4	10.14744852	18.15648988	1.609067679	0.17382807	0.378161262	-6.226019567
PPID	10.08960072	19.81472344	1.461519994	0.20888454	0.430375853	-6.407279971
GLRX5	9.915231744	20.4013684	1.373155872	0.233105047	0.46432684	-6.512843376
DCXR	9.716503504	19.94724636	1.377860842	0.231749907	0.462874026	-6.507288621
CCNT1	9.574626943	10.38287318	1.086848726	0.330961648	0.531148164	-6.83327157
AP3M1	9.30578764	19.00623605	1.385352887	0.229607585	0.459836852	-6.498427322
PFDN1	9.215445786	19.50720889	1.323226825	0.247958797	0.484629268	-6.571291057
DDAH2	9.213414273	18.69553349	1.399133941	0.225716486	0.455746025	-6.482076869
PCYT2	9.13003538	18.89555522	1.363245072	0.235984416	0.46921991	-6.524518351
ISG15	9.018756435	10.10493793	1.048477418	0.346562001	0.536595541	-6.873076901
LRPAP1	8.711974793	4.355987396	1.322758664	0.24810223	0.484629268	-6.571834627
ME2	8.68260417	19.15251588	1.251612625	0.270827559	0.508862281	-6.653403607
PURA	8.642667823	11.35812569	0.877651864	0.423770904	0.597425436	-7.038951457
NAPG	8.580217139	18.87530313	1.258852609	0.268429271	0.507821402	-6.645200662
LSR	8.573662675	18.49844204	1.290753902	0.258094998	0.496284922	-6.608787849
CDC45	8.572342374	18.61455467	1.279398928	0.261730121	0.499380171	-6.621798035
COPS5	8.571193429	18.48358362	1.281643456	0.2610078	0.49928992	-6.619230594
PSMD9	8.511111827	19.41224782	1.201573172	0.287950743	0.527180644	-6.709451173
HMOX2	8.467127075	18.83167503	1.240669484	0.274490176	0.513569731	-6.665758263
ABHD16A	8.457168585	11.49454178	0.845329649	0.43983827	0.611680331	-7.068036813

LAGE3	8.435336676	11.97264883	0.805051946	0.460517128	0.627695029	-7.103158445
SLC43A3	8.433215295	11.35858981	0.853958824	0.435502806	0.608552094	-7.060348898
WBP11	8.402400652	19.16164205	1.201605419	0.287939396	0.527180644	-6.70941543
CLPTM1	8.370355006	20.16145256	1.119554637	0.318152566	0.531148164	-6.798681927
TMEM205	8.299752611	19.64650816	1.145848333	0.308174841	0.531148164	-6.770452359
UBE2O	8.299486951	19.0997211	1.18628128	0.293377607	0.531148164	-6.726344462
TIMM8A	8.291772671	19.73869531	1.137885063	0.311166855	0.531148164	-6.779040607
CEP43	8.279127674	19.53746971	1.150546257	0.306421785	0.531148164	-6.765370207
TMEM70	8.270141535	11.14898137	0.853123402	0.435921075	0.60875322	-7.061095671
CHMP6	8.269981872	11.19923007	0.848800909	0.43809022	0.611013323	-7.064951017
NUP54	8.260587514	18.60827902	1.220382798	0.281400998	0.520357345	-6.688518601
XRN2	8.21977199	18.16673199	1.248937907	0.271718581	0.509673315	-6.656428243
TBCB	8.216733907	19.16410839	1.167539717	0.300154994	0.531148164	-6.74689212
SLC35A4	8.211723549	19.06850898	1.17397145	0.297813359	0.531148164	-6.739860282
STAG2	8.190559154	11.33565609	0.828312046	0.4484863	0.617364205	-7.083030584
DYNLL2	8.179947674	19.50866802	1.132250517	0.313299521	0.531148164	-6.785097189
POP1	8.178452444	18.58828715	1.206307235	0.286289304	0.52545572	-6.704198544
BAX	8.163058241	11.36107897	0.822951137	0.45123757	0.618966868	-7.087707369
MRPL45	8.162846341	20.35585924	1.0733285	0.336387402	0.531148164	-6.847394744
FHOD1	8.159640375	18.73372846	1.191190989	0.291625184	0.531148164	-6.720932958
PGRMC1	8.121647809	20.33518875	1.066266983	0.339251889	0.53195473	-6.854729274
MRPL11	8.107219863	18.68512979	1.184132702	0.294147497	0.531148164	-6.728708926
BMP15	8.106158815	12.02110979	0.766417668	0.481036335	0.637802859	-7.135630051
PSMG2	8.070287345	19.4344575	1.119984081	0.317987327	0.531148164	-6.798223834
NFU1	8.064873118	18.8768146	1.162189192	0.302115609	0.531148164	-6.75272598
COA6	8.045122123	19.38208796	1.117911415	0.31878554	0.531148164	-6.800433846
LYRM7	8.041425521	19.77279239	1.091823123	0.328984712	0.531148164	-6.828049124
WDR4	8.028935402	18.75360179	1.164442447	0.301288539	0.531148164	-6.750270946
DNAJA3	8.022658204	19.48435025	1.102052899	0.324951679	0.531148164	-6.817265519
TIMM17A	8.00887606	19.64588223	1.094542838	0.327908217	0.531148164	-6.825187888
ARPC5L	8.002185825	18.70713998	1.164282757	0.301347088	0.531148164	-6.750445021
EXOSC7	8.001423004	18.96543757	1.140016225	0.31036359	0.531148164	-6.776745446
ENOPH1	7.985667979	19.12874149	1.128574039	0.314698062	0.531148164	-6.789039949
POLRMT	7.985504069	18.98680986	1.139577059	0.310528967	0.531148164	-6.777218603
RAP2B	7.981574134	18.50289639	1.176014564	0.297072968	0.531148164	-6.737622204
MCCC2	7.958703264	19.15360054	1.122113115	0.317169249	0.531148164	-6.795951298
SH3GLB2	7.952878022	18.43258619	1.176674472	0.296834184	0.531148164	-6.736898881
NAT10	7.949736974	18.99103529	1.132673535	0.313138958	0.531148164	-6.78464307
NDUFS2	7.949514296	18.72991181	1.152211511	0.305802532	0.531148164	-6.763566012
RNASEH2B	7.932605077	11.5432072	0.782906241	0.472197238	0.634874809	-7.121920504
DNAAF5	7.917666253	18.54476611	1.15948384	0.303111319	0.531148164	-6.755670184
RHAG	7.916788853	10.90767225	0.832490518	0.446350824	0.616336327	-7.079369797
PPP2R5D	7.91363383	18.94122327	1.128805521	0.314609842	0.531148164	-6.788791914

PDXDC1	7.897862617	18.80480048	1.13555744	0.312046284	0.531148164	-6.781544609
RRBP1	7.881904466	19.09690833	1.112192288	0.320997253	0.531148164	-6.806519833
VPS4A	7.863008465	18.94252071	1.120678946	0.317720121	0.531148164	-6.797482403
OGDH	7.848479313	18.98217497	1.111213111	0.321377278	0.531148164	-6.80756003
WDR12	7.839655865	19.48424717	1.076886367	0.334952144	0.531148164	-6.843688379
ATP6V1D	7.835547951	11.41023343	0.782170647	0.472588978	0.634874809	-7.12253688
SAMM50	7.823667614	18.80884992	1.123611121	0.316594761	0.531148164	-6.794350856
RDH13	7.791143609	11.02402262	0.807918639	0.459021259	0.626040672	-7.100700926
EXOSC9	7.788894578	19.1279388	1.094068679	0.328095672	0.531148164	-6.825687018
XPNPEP1	7.786425496	11.16757119	0.795546493	0.465503562	0.63177517	-7.11126007
PMM2	7.780824862	18.20389487	1.162654876	0.30194451	0.531148164	-6.752218804
STAT1	7.759455318	18.83751015	1.108970684	0.322249076	0.531148164	-6.809940217
SNW1	7.743993052	19.13567098	1.084599982	0.331858753	0.531148164	-6.835627844
PAIP1	7.740587067	19.31533949	1.072208406	0.336840358	0.531148164	-6.848560075
PPP1R18	7.725465968	3.862732984	1.070000961	0.337734582	0.531148164	-6.850854543
LSM5	7.724633739	11.75747146	0.744277147	0.49309624	0.646678816	-7.153683153
SLC25A22	7.72151793	18.70134269	1.113394314	0.320531281	0.531148164	-6.805242183
PTDSS1	7.72086745	11.23272223	0.880154264	0.422546515	0.596836866	-7.036667106
TIMM23	7.72079098	19.59564747	1.049312929	0.346215654	0.536595541	-6.872219382
PYCR3	7.717584357	18.62655664	1.117674226	0.318876997	0.531148164	-6.800686604
MRPL37	7.714059486	19.01077273	1.088986839	0.330110644	0.531148164	-6.831028555
MRPL49	7.712936474	19.30388818	1.068213102	0.338460344	0.531148164	-6.852710809
NDUFA2	7.708016396	11.4107893	0.767912096	0.4802302	0.637802859	-7.134396747
MANF	7.702966803	18.15348498	1.149562775	0.306788037	0.531148164	-6.766435073
PKN1	7.688935718	18.19207487	1.145500859	0.308304858	0.531148164	-6.770827796
FAM114A2	7.681623425	10.61656933	0.829569051	0.447843062	0.617364205	-7.081930742
NUBP2	7.679572877	19.44007731	1.050699792	0.345641412	0.536595541	-6.870795067
NDUFV1	7.659261668	18.80241762	1.09318118	0.32844679	0.531148164	-6.82662092
TIMM8B	7.657477509	19.43503859	1.047939123	0.346785299	0.536595541	-6.873629153
UQCR10	7.652567956	20.55123045	0.977030008	0.377293266	0.557833489	-6.944811992
YIF1B	7.644954753	11.53438594	0.751685481	0.489036615	0.642493931	-7.147688356
FAM120A	7.639781562	17.84808452	1.15951049	0.303101496	0.531148164	-6.755641199
TWF2	7.629447208	11.68240306	0.738922197	0.496045846	0.6483441	-7.157987383
PCBD1	7.625624914	3.812812457	1.069999215	0.33773529	0.531148164	-6.850856357
NDUFA3	7.617693253	3.808846627	1.069999073	0.337735348	0.531148164	-6.850856504
SRSF9	7.614243416	18.76830668	1.088960874	0.330120967	0.531148164	-6.831055809
TMEM214	7.613061859	18.2449195	1.127983587	0.314923188	0.531148164	-6.789672494
PRMT3	7.604275891	18.58248633	1.098006227	0.326541839	0.531148164	-6.82153827
GPKOW	7.578564687	11.02820337	0.78288864	0.472206609	0.634874809	-7.121935258
PRRC1	7.57639873	3.788199365	1.069998328	0.33773565	0.531148164	-6.850857278
MRPS25	7.571542233	11.22703043	0.766488745	0.480997971	0.637802859	-7.135571436
NDUFS6	7.569192146	11.24881341	0.764574855	0.482031771	0.637802859	-7.137148312
RCOR1	7.542115301	19.13210655	1.049749595	0.34603476	0.536595541	-6.871771048
DOHH	7.54018582	18.82952417	1.071251002	0.337227944	0.531148164	-6.849555569

EIF1AD	7.537452333	11.31640653	0.75585705	0.486761431	0.641403572	-7.144292369
SPTBN1	7.529532028	19.49105356	1.01564254	0.360410375	0.547413899	-6.906441165
CD2BP2	7.496983569	11.15490853	0.763539494	0.482591708	0.637802859	-7.138000087
MRPS22	7.483959664	18.82669173	1.059671849	0.341946269	0.533599698	-6.861553098
DNAJC21	7.476145843	10.86248787	0.781947004	0.472708128	0.634874809	-7.12272419
GNPAT	7.470791496	3.735395748	1.069996366	0.337736446	0.531148164	-6.850859316
BROX	7.468795038	18.9150706	1.05221133	0.345016482	0.536128553	-6.869241404
MRPL50	7.466032578	11.43879964	0.738789501	0.496119099	0.6483441	-7.158093731
WAC	7.463244951	3.731622475	1.069996222	0.337736504	0.531148164	-6.850859465
EXOSC8	7.446872916	18.58611414	1.07161047	0.337082375	0.531148164	-6.849181862
RNASEH2C	7.446102784	10.78157642	0.787282666	0.469871587	0.634874809	-7.11824424
EMC1	7.423700253	19.4336339	1.009400124	0.363095801	0.548723914	-6.912708881
MEMO1	7.415034048	19.11646871	1.029188044	0.35464122	0.541975805	-6.892757274
CTNNBL1	7.404714086	10.99396159	0.765388268	0.481592201	0.637802859	-7.136478503
TTI1	7.398392584	10.73598807	0.785351108	0.470896968	0.634874809	-7.119868703
TDP2	7.379586372	3.689793186	1.069994603	0.33773716	0.531148164	-6.850861147
PC	7.364810896	3.682405448	1.069994311	0.337737279	0.531148164	-6.85086145
BIRC6	7.319851831	3.659925915	1.069993413	0.337737643	0.531148164	-6.850862384
MRPS28	7.301780856	3.650890428	1.069993047	0.337737792	0.531148164	-6.850862764
EIF2D	7.263531838	18.87894468	1.014522363	0.36089102	0.547413899	-6.907567681
PSME3IP1	7.252438292	11.10487467	0.739194072	0.495895786	0.6483441	-7.157769442
WASF2	7.236289679	10.68693835	0.769410986	0.479422662	0.637802859	-7.133157901
DCAF7	7.236078248	11.34350509	0.72000655	0.506566732	0.655817724	-7.172994904
C19orf25	7.210343426	18.66483602	1.021932366	0.357721623	0.545181512	-6.900101217
EMC2	7.196489964	11.05391398	0.736696688	0.497275445	0.649090713	-7.159769027
SEPHS1	7.194377627	18.90533558	1.005122917	0.364945577	0.549026601	-6.916989261
BMP2K	7.193605854	3.596802927	1.069990799	0.337738703	0.531148164	-6.850865099
RNF40	7.190521624	17.98155287	1.069471862	0.337949223	0.531148164	-6.85140408
SUMF2	7.189636537	3.594818268	1.069990715	0.337738738	0.531148164	-6.850865187
USP47	7.176197789	11.32084519	0.714659879	0.509569232	0.658935971	-7.177180843
ARMT1	7.166812161	19.3669601	0.969276291	0.380762136	0.560608089	-6.952400668
AK4	7.164145854	3.582072927	1.069990169	0.337738959	0.531148164	-6.850865753
SMARCA5	7.12845082	3.56422541	1.069989395	0.337739273	0.531148164	-6.850866557
PRPF38A	7.124414946	3.562207473	1.069989307	0.337739308	0.531148164	-6.850866649
DHX29	7.091264814	10.6849984	0.752764464	0.488447392	0.642099978	-7.146811382
HLTF	7.078059099	3.53902955	1.069988283	0.337739724	0.531148164	-6.850867713
NIF3L1	7.071360252	3.535680126	1.069988133	0.337739784	0.531148164	-6.850867868
PAAF1	7.064286575	10.6288968	0.75400985	0.487767942	0.641586878	-7.145797941
PARD3	7.036791777	3.518395888	1.069987354	0.3377401	0.531148164	-6.850868677
PFKM	7.001604032	11.13028228	0.708784996	0.512882888	0.661678563	-7.181751565
LIG1	6.990913954	18.01583596	1.029894163	0.354342649	0.541892721	-6.892040834
DCUN1D5	6.954168934	18.50316216	0.98941481	0.371807255	0.554090193	-6.932609085
CUL5	6.92962391	3.464811955	1.069984865	0.33774111	0.531148164	-6.850871264

YKT6	6.8651289	3.43256445	1.06998331	0.337741741	0.531148164	-6.850872879
PPP6R3	6.850614119	18.50590937	0.970678844	0.380132701	0.560470745	-6.95103091
METTL2B	6.835231915	18.77224511	0.949873942	0.389558449	0.568330177	-6.971214535
OGFOD1	6.822997483	18.95881816	0.939053819	0.394536183	0.572580635	-6.981595852
AP1G1	6.765487522	19.16948053	0.915628702	0.405490886	0.580440797	-7.003793665
TIAL1	6.739027367	18.73556179	0.938447332	0.39481673	0.572613283	-6.982175369
NRAS	6.664539575	11.04110163	0.676356149	0.531446289	0.677593933	-7.206432506
UBTF	6.612028049	19.11993266	0.890730671	0.417402621	0.59334812	-7.026961487
RIPK1	6.479865596	10.84674966	0.668824882	0.535823016	0.679500156	-7.212029559
DIABLO	6.42706083	18.7294243	0.885812702	0.419788269	0.59483408	-7.031484733
EEA1	6.34175495	10.38063776	0.68585381	0.525961932	0.672906917	-7.199301125
CFAP298	6.319019876	18.41314952	0.883361264	0.420981478	0.595596578	-7.033732809
KIF11	6.205358624	18.4012795	0.867794493	0.428621297	0.602350006	-7.047904767
CDK11A	6.144017909	19.55400164	0.797069877	0.464701686	0.631072853	-7.109966555
AGK	6.062409527	19.20813922	0.800989787	0.462643121	0.629432916	-7.10662955
NRBP1	5.860567423	17.06977106	0.866112022	0.429453526	0.602756087	-7.049425702
ATP6AP1	5.28498719	10.23071442	0.562672715	0.600073428	0.738895184	-7.285285012
MVD	5.22309667	18.57911266	0.674453221	0.532549837	0.677985133	-7.207851563
GINS1	5.049461165	4.86013626	0.697571202	0.519250014	0.667564184	-7.190391852
NELFCD	4.716800094	4.641306872	0.685486632	0.526173227	0.672906917	-7.199578328
ABCG2	4.651253873	25.24726855	12.54436689	0.000101427	0.006237778	2.187918485
SRI	4.642032003	26.07444467	22.13010394	7.82E-06	0.000642882	4.77472056
GRK2	4.577701843	2.288850921	1.069878499	0.337784251	0.531148164	-6.85098175
GSK3A	4.330457647	12.61189556	0.482751494	0.651452842	0.780547439	-7.333140272
EPS15L1	3.658268361	20.22461093	1.57330586	0.18174657	0.391549163	-6.270445856
KDM3B	3.574347299	20.42599172	1.487249452	0.202304445	0.423104207	-6.376084805
FDXR	3.438077758	24.0234154	9.414996848	0.000361954	0.012459833	0.769630116
NAA25	2.936325998	6.027960854	0.358092556	0.736179138	0.85126707	-7.394369254
CHERP	2.807173517	21.22160955	0.932886732	0.397396545	0.574574216	-6.987476871
OXCT1	2.625491149	23.69207975	26.01008806	3.75E-06	0.000320144	5.411513481
HMGCS1	2.604333558	25.26540281	13.3402674	7.70E-05	0.005026156	2.486326045
MRPL12	2.561379281	25.33461654	8.270149871	0.000637302	0.016637327	0.123592508
FAM98A	2.547964033	21.8300867	1.471837544	0.206221124	0.425678766	-6.394793805
CWC27	2.462396408	5.749903519	0.316836834	0.765293554	0.875319435	-7.410864464
SNF8	2.450455589	5.802929637	0.312609339	0.768303587	0.876087184	-7.41244598
AK1	2.443541539	24.46255531	2.335782188	0.071481578	0.251064472	-5.293022294
GFUS	2.37876196	23.25910163	11.16310315	0.000170588	0.008404568	1.615324671
NDUFA4	2.29002982	15.77666594	0.218977329	0.836086845	0.926272211	-7.442195683
CPNE3	2.268138623	23.35108685	7.022407283	0.001286381	0.027185526	-0.686607195
MPI	2.259357621	8.404801592	0.249530503	0.813750078	0.912436293	-7.433607191
TMEM167A	2.20789964	18.19608708	0.407700434	0.701843483	0.821842052	-7.372021497
H1-10	2.104469109	23.13658995	4.843286658	0.0059221	0.060005206	-2.461074991
UFM1	1.936041552	24.93413047	4.721249478	0.006546475	0.064277112	-2.57749266

DYNLL1	1.935126915	24.974783	5.493939232	0.00357433	0.045126058	-1.873918491
NAXE	1.899931819	23.94045592	5.580788842	0.00335314	0.044027317	-1.799593344
STXBP2	1.833410269	23.5283895	1.473165009	0.205880867	0.425372108	-6.393185052
CNDP2	1.809350558	24.6959594	5.823342122	0.002816654	0.042376182	-1.596754142
HSPBP1	1.751404277	23.66925654	10.21970691	0.000252243	0.011194533	1.177815138
EIF2S2	1.7108751	24.78717444	12.39975361	0.000106821	0.006237778	2.131366264
ITPA	1.702316383	23.76420568	1.884358206	0.12351915	0.301196698	-5.876667931
RPS26	1.678728822	25.95130219	5.179240669	0.004535799	0.051351725	-2.151050431
KIF4A	1.677834566	16.31430745	0.173694741	0.869517705	0.947502671	-7.452901409
UBE2E2	1.622651533	14.83919012	0.165219103	0.875812933	0.952194463	-7.454634779
TTC4	1.601646358	23.1720501	5.106245584	0.004801014	0.052739851	-2.217136637
SSR3	1.572357423	24.545279	4.573947683	0.007406883	0.067588943	-2.720803729
HDGF	1.552526201	26.21387029	7.035070771	0.00127657	0.027185526	-0.677739556
NPC2	1.528147226	23.59112188	5.490897062	0.003582388	0.045126058	-1.876538524
ACY1	1.519816771	23.41173127	7.342717705	0.001063668	0.024577287	-0.466619543
MRPS35	1.506400273	7.682824046	0.166243166	0.875051738	0.951833239	-7.454429894
NUP188	1.496481565	13.8048366	0.161707557	0.878424272	0.954565847	-7.455327835
UQCRB	1.48930954	23.36209192	6.697824696	0.001571786	0.03142156	-0.918895863
PSMG3	1.485177226	23.74448552	2.546698138	0.05584844	0.222092632	-5.022472129
NUP35	1.469906811	22.69198359	9.445163496	0.000356907	0.012459833	0.78556976
RPS28	1.457931378	26.76519381	4.703327829	0.00664459	0.064662285	-2.594764333
THUMPD1	1.416087613	23.61737372	10.77797014	0.000199348	0.009411767	1.44168209
PCK2	1.40494627	23.52667784	5.761738808	0.002942548	0.042399436	-1.647621952
GALE	1.403299644	23.58465544	5.771654504	0.002921837	0.042376182	-1.63940479
CA1	1.402253158	24.04488151	5.727560578	0.003015291	0.042642192	-1.676032716
SMC2	1.401229398	22.96512554	1.016588712	0.360004815	0.547413899	-6.905489028
ISYNA1	1.400365997	25.08068781	8.311764425	0.000623579	0.016637327	0.148576956
NOSIP	1.390600521	23.53919666	8.770563901	0.000493614	0.015212915	0.416231288
NQO1	1.386943223	24.334799	12.15124022	0.000116928	0.006652904	2.032414472
DNAJB1	1.370418042	24.54688098	8.341901039	0.000613863	0.016637327	0.166594909
MRPL4	1.366592169	22.75467005	4.870459593	0.005792836	0.059043347	-2.435433948
SLC1A5	1.350393547	25.91646506	3.470651035	0.020553926	0.119709088	-3.897372101
TMED10	1.347754311	23.36885523	3.545739582	0.019064441	0.115584687	-3.811339375
CTSD	1.346185627	24.01370167	3.478531976	0.02039143	0.119075217	-3.888301504
VPS33B	1.322214044	7.901483536	0.141346254	0.893600066	0.967739652	-7.459055966
NCAPD2	1.316671259	22.84065343	4.396933325	0.008623879	0.074819745	-2.897159266
STRBP	1.301695835	22.49558959	1.7375106	0.148153637	0.343883808	-6.064388865
EFHD2	1.290927501	23.29766776	1.129194329	0.314461714	0.531148164	-6.788375237
CT45A10	1.290862622	23.18787299	5.187381659	0.004507313	0.051290906	-2.143722924
SRP19	1.280966754	22.95231357	8.479189238	0.000571846	0.016637327	0.247885032
LRRC47	1.280338201	23.6806595	2.809422566	0.041451942	0.186198098	-4.691193082
OSGEP	1.277937897	23.22414321	10.54367926	0.00021973	0.010157952	1.332753559
SERPINB9	1.27779978	23.4851823	5.168901106	0.004572288	0.051420303	-2.160369197
HNRNPUL	1.261752319	22.9376579	2.535160696	0.056598085	0.224269913	-5.037181591

2						
ANK1	1.248917623	24.11157782	5.797947071	0.002867757	0.042376182	-1.617670885
EEF1D	1.244165762	26.73539426	3.950057972	0.012906614	0.093193597	-3.363180838
RIDA	1.243562936	22.89092209	11.28375826	0.00016263	0.008201743	1.668389813
ATP5MF	1.239695077	24.83539411	1.413135891	0.221828059	0.449759711	-6.465398496
DLAT	1.232005422	24.32613126	5.475761149	0.003622803	0.045162923	-1.889590936
AP3B1	1.230130543	14.8865672	0.124904022	0.905893953	0.977238056	-7.461703954
CDC37	1.229704279	25.98087532	10.45262816	0.000228327	0.010339958	1.289723405
PLEC	1.226807339	23.43091471	4.008303275	0.012226067	0.092108371	-3.300713989
POLR1C	1.22006173	22.67324639	9.46270375	0.000354012	0.012459833	0.794813964
MBOAT7	1.216681502	24.0533339	9.946183795	0.000284319	0.011903841	1.042927782
GNPDA1	1.207347328	23.72013695	2.741423808	0.044730681	0.193862072	-4.776202354
CHMP5	1.205374063	22.53124652	2.50197171	0.058817597	0.226631173	-5.079560433
PRDX5	1.19859053	25.21376872	6.801822446	0.001472745	0.029981849	-0.843405396
GSS	1.196072728	24.37596484	2.974655361	0.034557737	0.168245682	-4.487041688
WDR82	1.190556329	15.00573567	0.119924441	0.909623431	0.979356814	-7.462441833
DYNC1I2	1.183979119	24.24805289	2.845732869	0.039812698	0.183037089	-4.646029652
VDAC1	1.175236496	26.51435469	4.187079403	0.01038509	0.082596827	-3.112233966
CLTB	1.174308665	23.16801926	2.6857743	0.047631821	0.202480863	-4.846172675
ATP5PD	1.17010498	25.07638949	4.593206398	0.00728711	0.067095841	-2.701890861
SUPT16H	1.168879	23.31403499	6.020042999	0.002455605	0.039485423	-1.437203903
DYNC1H1	1.168783985	25.42893358	8.433400848	0.000585462	0.016637327	0.220916264
ATP6V1B2	1.160952762	24.49417255	6.636716443	0.001633712	0.032367925	-0.963733749
ARHGEF1	1.151413895	22.62775196	5.23518857	0.004344256	0.050736342	-2.100865596
LAP3	1.144488376	25.33163807	8.35816857	0.000608694	0.016637327	0.176294696
CAST	1.144485048	24.17648685	5.536201739	0.003464591	0.044697248	-1.837636766
MGST3	1.135604128	24.62477131	5.685600929	0.003107553	0.043088383	-1.711097686
PUF60	1.130785096	25.47299513	8.843457847	0.000476106	0.014879996	0.457484861
SRSF2	1.127004575	25.44546895	4.299287264	0.009395683	0.077616364	-2.996414758
LONP1	1.116882769	25.3473103	6.083844094	0.002350571	0.038922012	-1.386372033
CAPNS1	1.112046415	24.972443	4.460309492	0.008162842	0.072415932	-2.833493176
PSMG4	1.103677591	15.1826154	0.109928869	0.917117712	0.982657752	-7.463832925
HMGA1	1.098921994	24.99868454	4.290055371	0.009472774	0.077616364	-3.005872329
CHCHD4	1.09649833	23.30864462	2.430248687	0.063951025	0.236512206	-5.171449402
FKBP3	1.09505309	23.82769123	4.646977455	0.006964617	0.065485103	-2.649366659
NEDD8	1.09066495	7.453185331	0.1104261	0.916744664	0.982657752	-7.463766568
FKBP1A	1.089214546	25.11725926	9.260814395	0.00038914	0.012698543	0.687343468
AHSA1	1.084505003	25.90180496	3.669756966	0.016869585	0.1097013	-3.671165555
SNRPF	1.076516354	24.63457108	7.875750325	0.00078725	0.019598683	-0.119370291
PDCD10	1.066632135	24.24567582	2.709040204	0.046393967	0.198741724	-4.816877153
PSMA4	1.061822181	26.35128822	2.185760346	0.085513018	0.261829082	-5.486970211
COPZ1	1.059169119	23.9508098	1.652732034	0.164628666	0.366043097	-6.171410282
COX7A2L	1.058615041	22.84937611	1.285190409	0.259870132	0.497972212	-6.615169049
DPM1	1.046983319	23.14408253	2.881955218	0.038250045	0.177195928	-4.601141026

DECR1	1.046374668	24.47630576	2.576793838	0.053944433	0.218834911	-4.984159513
SSR4	1.04352402	24.42886795	5.263823985	0.004249947	0.049897526	-2.075335312
HSPA4L	1.041339728	26.09123827	2.836107219	0.040240006	0.184108398	-4.657986217
SKP1	1.031923742	24.4437021	5.347259149	0.003988727	0.047564717	-2.001541349
CPT1A	1.023558306	23.07623875	3.365511121	0.022871404	0.128915829	-4.019297988
APEH	1.020491513	23.50997284	3.965924994	0.012716926	0.093131547	-3.346111856
CANX	1.011693266	27.19003753	11.11017746	0.000174227	0.008404568	1.591850084
CLNS1A	1.011335602	24.38448565	9.787967054	0.00030513	0.011983694	0.963128417
VIM	1.007388454	26.10663232	7.311726958	0.001083071	0.024577287	-0.487518761
AUP1	1.007058933	15.21477245	0.100049573	0.924534656	0.989171843	-7.465089548
RAD50	1.002164075	23.12417724	2.182606702	0.085838396	0.261829082	-5.491054189
M6PR	0.996925675	23.78969737	4.137294695	0.010862959	0.08457862	-3.164230611
ATP6V1E1	0.989184802	23.22625427	3.051744807	0.031793737	0.158104138	-4.393043056
VAT1	0.98526863	25.34322039	3.875655714	0.0138414	0.096113013	-3.443739471
DDT	0.982227519	23.81274189	2.873883446	0.038592166	0.178408366	-4.61112947
MTDH	0.979337927	23.85166999	7.524931742	0.000957682	0.022850495	-0.345353962
SUCLG2	0.976513546	23.30729157	5.940042826	0.002595351	0.040844568	-1.501573171
PGM2	0.974052164	23.30597094	2.029044084	0.103415366	0.27114534	-5.689934674
HSPA4	0.972275752	26.79228421	5.789355244	0.002885296	0.042376182	-1.624764303
ARHGDIA	0.969267986	26.40944011	7.551333277	0.000943401	0.022754425	-0.328008698
IPO9	0.966947314	23.23523171	2.225610826	0.081514602	0.259574428	-5.435380549
STIP1	0.963311274	27.19458525	9.397158968	0.000364979	0.012459833	0.760180351
RANGAP1	0.963214163	24.49682951	8.276886123	0.000635056	0.016637327	0.127645028
COX5B	0.962740809	24.48844333	3.904026976	0.013475907	0.095192709	-3.412919358
NAA50	0.960929409	23.59022926	8.439370267	0.000583665	0.016637327	0.224440247
TBCD	0.958662793	22.37928634	5.35106293	0.00397728	0.047564717	-1.998198022
WARS1	0.952873091	26.2436741	4.232373423	0.009971639	0.079881106	-3.065253891
MGST2	0.946892583	24.47045253	1.253334042	0.270255538	0.508648889	-6.651455317
DIAPH1	0.938483576	25.21909857	5.446443015	0.003702642	0.045645352	-1.914953115
UBE2M	0.937970927	24.58475867	3.214531859	0.026746318	0.139367113	-4.197322211
HDGFL2	0.935806337	23.03427129	1.834711188	0.131332674	0.314371285	-5.940400128
RBM3	0.932635466	25.44319745	4.660731967	0.006884851	0.065351182	-2.635997303
MCFD2	0.923017393	23.6519031	2.218975665	0.082166021	0.260003361	-5.443967791
BTF3L4	0.918092878	24.60317792	3.591922377	0.018210474	0.114473204	-3.758860156
ATXN10	0.9161834	23.5067615	4.391413388	0.008665468	0.074819745	-2.902732461
GMFB	0.916018334	14.93168628	0.092774873	0.930001933	0.991495591	-7.465939603
COPS8	0.911643476	23.00873147	5.893064004	0.002681846	0.041824071	-1.539704556
RALA	0.911289356	22.76555991	4.439129265	0.008313613	0.072916632	-2.854704623
LARP4B	0.908511494	14.28418928	0.096184634	0.927438758	0.990696335	-7.465549124
FABP5	0.908385932	24.7140982	2.450120999	0.062480776	0.234437591	-5.145950262
PABPN1	0.90228969	23.03754064	1.096543699	0.327118225	0.531148164	-6.823080258
ATP6V1H	0.899721943	23.1352126	3.29067827	0.024704935	0.133282652	-4.107106653
SLC9A3R1	0.899500607	24.17835397	5.413322917	0.00379537	0.046433648	-1.943731465
ERP44	0.897377284	24.74502227	3.734523844	0.01584069	0.105874971	-3.598914412

LRRFIP1	0.895647269	22.79873882	2.865101389	0.038968331	0.179772819	-4.622006356
GPI	0.88749986	27.11922409	9.787494142	0.000305195	0.011983694	0.962887898
PFDN6	0.886850525	24.39988542	10.08507659	0.000267451	0.011412944	1.11190051
KPNA2	0.872558053	26.16163845	5.789951422	0.002884075	0.042376182	-1.624271824
C1QBP	0.871762636	27.90443389	3.536043268	0.019249579	0.116389141	-3.82239977
ACSL3	0.867807232	23.58658514	3.222042498	0.026536583	0.138878959	-4.188385443
CLIC2	0.863630555	25.24191799	6.295500147	0.002038615	0.036296778	-1.220863308
HSPH1	0.858286878	26.2283141	7.901272611	0.000776341	0.019576133	-0.103300893
ZW10	0.852908163	22.73369216	3.950735101	0.012898452	0.093193597	-3.362451618
CCAR2	0.851600634	24.06983224	4.557660636	0.007509992	0.068019072	-2.736839947
SEPTIN8	0.839199672	23.75013008	2.826572154	0.040668407	0.184736741	-4.669841688
RAB18	0.83764732	22.78657612	5.610414803	0.003281447	0.043601978	-1.774447141
ARHGAP1	0.837141624	23.06952523	2.776210229	0.04301826	0.190154421	-4.732644866
PRDX6	0.835335335	27.82717747	4.089889182	0.011342132	0.087389549	-3.214093251
LARP1	0.829613706	24.38554747	2.945101855	0.035689065	0.170433059	-4.523293322
CKMT1B	0.82412541	25.07687855	6.282313556	0.002056542	0.036296778	-1.231037132
RPL34	0.818085737	25.70145891	6.725942981	0.00154424	0.031151523	-0.89838425
LSM14A	0.817343487	22.64015866	5.366008344	0.003932683	0.047564717	-1.985079322
NIT2	0.816722726	22.24981682	3.625347013	0.017620038	0.112452715	-3.721085616
UNC45A	0.809835666	22.87239603	1.863441297	0.126750142	0.30738641	-5.903546919
ACTR3	0.808391165	24.93498896	3.08857923	0.030562607	0.153783279	-4.348422544
NUP62	0.806317145	24.37026763	2.164217351	0.087762594	0.261829082	-5.514871261
MT-CO2	0.805075691	24.98266837	3.566136065	0.018681691	0.114832887	-3.788121194
STUB1	0.802956522	23.3730145	6.309769516	0.002019427	0.036296778	-1.209874389
MRPL22	0.801604645	15.0445495	0.080592243	0.939167626	0.997612715	-7.467219999
SRSF1	0.799038747	26.10913987	3.500703062	0.019942194	0.118874246	-3.862835138
NUP85	0.798737536	22.83976051	4.469391826	0.008099178	0.072177015	-2.824417582
CARS1	0.795964661	23.55893014	3.239861171	0.026046503	0.137285487	-4.167216855
SSR1	0.794140151	24.18399501	2.765620996	0.043531594	0.191217674	-4.745888979
RBM27	0.788708594	22.76842359	2.433050336	0.063741451	0.236512206	-5.167852735
HNRNPK	0.788651637	28.03889527	3.227142534	0.026395241	0.138716084	-4.182321776
DNAJC9	0.780171798	23.63991384	3.498969285	0.019976909	0.118874246	-3.864823864
HYPK	0.775112685	23.95476038	3.381910274	0.022490834	0.128296042	-4.000169041
PLIN3	0.774834394	25.31265679	7.308000762	0.001085432	0.024577287	-0.490037046
FERMT3	0.774128029	26.17492155	3.050134718	0.031848828	0.158104138	-4.394997859
ASF1A	0.77085074	22.48115543	2.946094312	0.035650397	0.170433059	-4.522074023
NPLOC4	0.76593565	23.07108047	3.359958506	0.02300194	0.129218495	-4.02578419
H2BC12	0.764904151	26.09808977	6.41278014	0.001887143	0.035696381	-1.131165731
CCDC47	0.764294052	23.52315223	1.508950893	0.196913007	0.414563532	-6.349627442
MTA2	0.763952349	24.23840672	1.37849828	0.231566891	0.462874026	-6.506535451
SRP68	0.762118902	23.60850588	5.622814248	0.003251985	0.043601978	-1.763953682
RTRAF	0.761735414	23.46923145	1.302955234	0.254241618	0.492305779	-6.594748617
G3BP2	0.761352853	24.27051821	2.498834194	0.059032365	0.226631173	-5.083571566
FDFT1	0.756715328	23.46013793	2.01519282	0.105180562	0.271706248	-5.707855405

BTF3	0.756512264	26.00484452	1.496884749	0.199892985	0.41924625	-6.364353969
PCNP	0.750353098	22.96117024	3.579218897	0.018440872	0.114832887	-3.773262507
KDM1A	0.749689009	22.56998851	1.531716344	0.191409543	0.406973212	-6.321736582
CKB	0.742874944	27.55687786	3.30278478	0.024397149	0.132729416	-4.09284341
NDUFAB1	0.742725988	25.1955158	6.077092264	0.002361429	0.038922012	-1.391730441
FASN	0.742708547	27.78382591	5.989622236	0.002507659	0.040032335	-1.461597596
CIRBP	0.742253496	23.61254528	2.320136412	0.072820336	0.252008826	-5.313207242
AHCY	0.740011974	26.88370403	5.651710427	0.00318454	0.043088383	-1.739570077
MCTS1	0.739645378	23.89709378	6.24492421	0.002108408	0.036296778	-1.259982462
LRBA	0.73592736	22.94085614	2.116470946	0.092979412	0.261829082	-5.576722636
GRPEL1	0.734975957	23.86204501	3.799174423	0.014885746	0.101013668	-3.52744569
HBZ	0.734390065	28.7550693	6.051564092	0.002403026	0.038922012	-1.412034883
TCOF1	0.733893733	23.40574527	2.054841299	0.100211448	0.266949824	-5.656543453
ECI1	0.73297603	23.76793764	3.367829299	0.022817159	0.128915829	-4.016591436
GALNT2	0.73148229	22.63822647	1.28037513	0.261415737	0.499380171	-6.620681651
SRM	0.731163451	25.79109472	2.924723914	0.036493641	0.172296574	-4.548357997
SERPINH1	0.723572749	25.94521499	3.653678098	0.017136922	0.110543107	-3.689203863
TPI1	0.717820708	27.99087793	3.032073966	0.032474313	0.159425885	-4.416950258
ACTBL2	0.715948232	30.37641859	2.413410156	0.065226734	0.239236567	-5.193077783
UFD1	0.715673775	23.87538815	3.553985913	0.018908611	0.115584687	-3.80194444
SNX3	0.713411837	24.42101772	3.371992506	0.022720112	0.128915829	-4.011732806
RAB27A	0.708595767	23.42286018	3.274154176	0.025132264	0.134058879	-4.126610081
PDCL3	0.707792659	22.72327587	4.9506428	0.005430438	0.05710968	-2.360359098
GFM1	0.705079828	23.33401358	3.185019059	0.027589074	0.142704327	-4.232519714
HK2	0.704768116	23.06325496	1.770854429	0.14214571	0.333426355	-6.021990807
SLC17A8	0.698373158	7.885957115	0.066940812	0.949451319	1	-7.468441517
TXNL1	0.69679747	25.31191463	3.520073732	0.019559051	0.117938952	-3.840647721
MAT2A	0.69413078	25.9559663	4.682546685	0.006760545	0.064662285	-2.614848499
MTHFD1	0.692216467	24.2996571	4.688579785	0.006726637	0.064662285	-2.609011458
CTTN	0.6889663	24.51709598	3.724804979	0.01599033	0.10655418	-3.609714616
IFITM1	0.684767412	27.42780915	5.164422241	0.004588202	0.051420303	-2.16441018
MACROD1	0.682796969	15.04189742	0.068556015	0.948233937	1	-7.468308755
ATP5PB	0.679945655	25.37396454	1.485962161	0.202628776	0.423383479	-6.377650088
HBE1	0.678290678	28.53452258	5.659570766	0.003166484	0.043088383	-1.732954332
EIF2B2	0.676024187	23.01801329	1.848505995	0.129111166	0.312088972	-5.922715046
ACTN4	0.672069987	26.37828337	5.521850785	0.003501398	0.044911	-1.849932551
PKLR	0.670703512	26.47823059	6.132951997	0.002273373	0.03821677	-1.347546848
FKBP2	0.669517524	23.76936447	1.950519606	0.113856704	0.283237697	-5.791425833
CKS1B	0.667814956	24.72864845	8.075770686	0.000706439	0.018018249	0.005265342
VAMP3	0.666486801	23.01699703	1.287611102	0.259096367	0.497348476	-6.612394148
TTC1	0.666313766	23.73217439	3.679727763	0.016706238	0.109354402	-3.659999934
MTHFD2	0.666027364	23.77138156	1.69265471	0.156649538	0.355061618	-6.121163817
FAF2	0.665339934	23.27369068	2.22879294	0.081204185	0.259574428	-5.431262663
QDPR	0.664728894	23.22989847	2.019744562	0.104596977	0.271706248	-5.701967069

PSME2	0.663831987	24.6351516	3.24495599	0.025908291	0.137208828	-4.161172899
SEC61B	0.660461579	24.21113285	2.675819913	0.048172718	0.204337029	-4.858725035
APRT	0.658923261	25.34222525	5.44926564	0.003694865	0.045645352	-1.912506763
DDB1	0.658347552	24.56526228	4.787651275	0.006197579	0.06194787	-2.513891578
UNC13D	0.656533664	21.76195392	2.218638054	0.082199317	0.260003361	-5.444404757
LMNB1	0.655290464	25.58464294	4.194419713	0.010316739	0.082348359	-3.104599346
LETM1	0.654955048	24.34615448	3.573815434	0.018539896	0.114832887	-3.779396205
AIMP2	0.654662217	24.78348612	3.97417959	0.012619533	0.093032371	-3.33724735
ISOC2	0.654386203	24.71136412	5.614953461	0.003270626	0.043601978	-1.770604023
FLII	0.652614039	14.30711566	0.069006207	0.947894655	1	-7.468271189
NCAPG	0.650649179	22.88765283	1.579321636	0.1803897	0.389003639	-6.262992633
SAMSN1	0.650122967	23.48534074	1.418150886	0.220451152	0.44822097	-6.459409103
OLA1	0.649772586	25.75187541	2.133292439	0.09110432	0.261829082	-5.554931001
NDUFV2	0.649600976	22.91302145	2.605526171	0.052193936	0.215676619	-4.947662811
TERF2IP	0.644338937	23.24495543	2.25659081	0.078546498	0.257646807	-5.395302937
DNMT1	0.643714898	23.71567519	1.609292745	0.173779347	0.378161262	-6.225739086
DBI	0.641662649	26.37801904	6.232178937	0.002126445	0.036296778	-1.269882736
SEC23B	0.641515018	22.69877395	2.114281345	0.093226526	0.26186033	-5.579559152
SH3GL1	0.640711586	23.46896214	3.672128273	0.016830569	0.1097013	-3.668508683
ACOT7	0.638538346	24.1611968	3.951815403	0.012885442	0.093193597	-3.36128836
ATP5MG	0.638249211	25.1120877	4.625313075	0.007092479	0.06612694	-2.670478999
RARS1	0.63104487	24.97028548	6.390927204	0.001914317	0.035696381	-1.1477727
CAPN2	0.630903577	23.64025807	2.101170743	0.094721006	0.263614246	-5.596542789
RPS17	0.630369125	26.96146312	5.795204511	0.002873342	0.042376182	-1.619934224
PPIG	0.629827844	7.716763257	0.061703239	0.953399975	1	-7.468850288
SRSF7	0.628543151	26.06765306	1.966866033	0.111594439	0.280540174	-5.770322027
MPC2	0.627990271	14.64955012	0.064827029	0.951044725	1	-7.468610488
TTLL12	0.626678454	24.4149305	2.842235362	0.039967366	0.183238813	-4.650372758
NCL	0.624918666	27.30914635	5.131690366	0.004706524	0.052043914	-2.194021219
PRKCB	0.623824222	24.19771093	4.09140193	0.011326463	0.087389549	-3.21249679
SLC25A13	0.619903838	23.63272196	4.035812622	0.011919293	0.09088973	-3.271392702
LMNA	0.619455095	26.02123173	4.018608569	0.012110076	0.091714195	-3.289716276
SRSF3	0.619063793	26.04872548	1.834045933	0.131440808	0.314371285	-5.941252504
EIF3J	0.617452021	24.53819168	2.589195168	0.053180986	0.217901685	-4.968397129
CPD	0.615007749	7.172632775	0.064816226	0.951052869	1	-7.468611337
NUP37	0.614417481	22.53026355	1.673405268	0.160446526	0.360161167	-6.145426627
GSE1	0.61402233	22.45004754	2.028316534	0.103507295	0.27114534	-5.690876128
GRN	0.613828237	24.03824603	2.252899258	0.078893923	0.257709698	-5.400076966
PDHA1	0.610988809	23.6616884	2.716021271	0.046029614	0.19832954	-4.808098643
ACTR1A	0.610831335	24.49958171	2.956174764	0.03526033	0.169368669	-4.509697012
JPT1	0.609619488	26.06940774	2.660836417	0.048999908	0.206320294	-4.877639229
ALDH9A1	0.608832309	23.48446265	4.313698635	0.009276811	0.077219962	-2.981676572
CALR	0.606800923	27.84672576	2.822251844	0.040864207	0.185056482	-4.675217089
ELAC2	0.606027233	22.44357577	2.900695623	0.037468895	0.175585062	-4.577983123

BAG3	0.601632886	22.39635527	2.721717549	0.045734695	0.197442196	-4.800939797
STOML2	0.598947276	24.60624086	3.702920824	0.016333338	0.108206126	-3.634087607
TPM4	0.594529071	25.61200667	6.412612895	0.001887349	0.035696381	-1.131292644
TXNDC17	0.593702873	25.09208958	2.882565291	0.038224327	0.177195928	-4.60038643
GYPC	0.593316271	15.71183025	0.057019298	0.956932561	1	-7.469187702
SEPTIN11	0.591668844	24.20743837	4.915544277	0.005585662	0.057825299	-2.393113918
SACM1L	0.588792912	22.34954604	1.960446518	0.112477146	0.282337994	-5.778611582
PUS7	0.588530476	23.45751818	1.15924456	0.303199528	0.531148164	-6.755930409
GSTP1	0.587089223	28.80949434	2.459542967	0.061796778	0.233209268	-5.133870695
UBQLN4	0.586742425	23.68317415	0.696347364	0.519948241	0.668074781	-7.191328103
H2AC20	0.586075522	26.42737773	5.189950934	0.004498367	0.051290906	-2.141412162
ATP6V1G1	0.576464883	23.29900492	1.686677281	0.157818697	0.35553268	-6.128704884
SPC24	0.573380745	23.30255189	2.065002161	0.098978764	0.265979027	-5.643387193
SYPL1	0.572259224	23.68240714	1.039636608	0.3502451	0.539740714	-6.882124899
CCDC124	0.570479361	22.91279238	2.805467608	0.041635046	0.186642762	-4.696122176
PYCR1	0.570450979	24.77102591	3.390873469	0.022285915	0.127784099	-3.989731214
COPG1	0.570119786	23.68508903	3.294502279	0.024607237	0.133179166	-4.102599022
UBQLN1	0.569856497	24.23882147	2.764488105	0.04358692	0.191217674	-4.747306689
SUMO2	0.569516834	26.83574575	5.487758729	0.003590724	0.045126058	-1.879242558
UBXN1	0.566486261	24.21132673	2.897862823	0.037585806	0.175585062	-4.581480745
HSPA9	0.565878174	27.59974721	6.313676117	0.002014212	0.036296778	-1.206869565
SNX2	0.565458486	23.11582598	3.667455824	0.016907546	0.1097013	-3.673744651
SAR1B	0.563760404	24.5541204	1.303337996	0.254121613	0.492305779	-6.594307219
AFDN	0.561181117	22.25144374	2.192034342	0.08486966	0.261829082	-5.478845863
ADSS2	0.558321266	23.95132818	3.165581806	0.028160736	0.143983116	-4.255770927
IPO4	0.558265433	23.68514065	2.970344924	0.034720177	0.168586592	-4.49232171
HMBS	0.557883089	25.05538205	5.15572763	0.004619284	0.051508496	-2.172262192
ZC3H15	0.556545323	23.9009771	0.847270798	0.438860083	0.611318596	-7.066312353
YBX3	0.554551984	25.73973337	2.601157567	0.052455958	0.216356453	-4.953206752
CYRIB	0.548350939	24.37767516	2.541787775	0.056166133	0.222956439	-5.02873105
NUDCD2	0.545615704	24.20529912	1.354784994	0.238469128	0.472466961	-6.534456223
NARS1	0.543166614	25.99700864	3.210341695	0.026864153	0.139605519	-4.20231164
H2AC12	0.54212704	26.50080787	2.974218877	0.034574146	0.168245682	-4.48757624
SMARCC2	0.541104807	22.8770196	1.544940819	0.188282806	0.402504379	-6.30547338
ATL3	0.539819263	23.66964955	1.621746635	0.17110462	0.37394601	-6.210202221
NTMT1	0.539345388	23.45672399	2.455957388	0.062056096	0.23339403	-5.138466846
GFPT1	0.537965913	23.93480528	3.979198607	0.012560742	0.093032371	-3.331862637
VPS35	0.536687593	24.60747999	3.059690661	0.031523446	0.157546231	-4.383401368
PCNA	0.536284102	26.68762923	3.37665205	0.022612059	0.128656819	-4.006298071
TMED9	0.535398019	23.96202611	2.628269842	0.050853196	0.212025573	-4.918830975
TBCA	0.533875702	25.63865785	4.825754404	0.006007317	0.060591982	-2.47767278
SUB1	0.533190187	26.65992369	3.563255444	0.018735204	0.114843693	-3.791396404
GLRX3	0.530380296	25.04566535	2.360019076	0.069460673	0.247008387	-5.261779357
UAP1	0.530205366	24.79707945	2.101825504	0.094645762	0.263614246	-5.595694629

SRP72	0.527849	24.44909811	2.557298691	0.055169392	0.221370512	-5.00896777
TOMM22	0.525887021	24.66293946	2.560376322	0.05497397	0.221370512	-5.005049018
PPA2	0.525706059	24.62019798	0.759007827	0.48504813	0.639905946	-7.141717704
EIF3A	0.523788494	25.17766057	5.321057523	0.004068647	0.048023022	-2.024620592
EIF3G	0.522613088	25.2558313	3.341559084	0.023440666	0.130363002	-4.047310765
RSL1D1	0.519639942	23.87509915	1.598292475	0.176176834	0.381773823	-6.239435144
PGAM1	0.518277047	27.40945771	2.124139927	0.092119449	0.261829082	-5.566787771
SLC25A5	0.516492554	27.53678899	2.319516732	0.072873922	0.252008826	-5.314006956
VPS28	0.516277776	22.38197185	3.580272345	0.018421638	0.114832887	-3.772067224
GET3	0.513475681	23.43617125	1.398294068	0.2259518	0.455806403	-6.483075189
PREP	0.510805218	23.28880702	1.621509708	0.171155116	0.37394601	-6.210498101
ACADVL	0.510772048	24.03636276	2.937005904	0.036006281	0.170757404	-4.533244646
SMN2	0.510612381	23.64760952	1.543254117	0.188678764	0.402962635	-6.307550098
SYAP1	0.508593094	23.07664343	3.620313509	0.017707516	0.112587331	-3.72676305
UBAC1	0.505785505	24.12631918	1.477828469	0.204689878	0.425165331	-6.387529351
VAPB	0.505658072	25.05053426	1.506045446	0.197626539	0.415394433	-6.353177118
ECHS1	0.504909394	24.65628427	2.396373162	0.06654609	0.240890331	-5.214980687
CTSC	0.499906621	23.60566867	2.159589389	0.088254157	0.261829082	-5.520865821
EXOSC10	0.496450089	22.35320031	2.671971252	0.048383687	0.204337029	-4.863581032
TCEA1	0.491981	23.68624481	2.758841897	0.043863844	0.191602105	-4.754374671
DCTN2	0.489134844	23.70156041	1.537673566	0.189994718	0.405087043	-6.314415967
TMCO1	0.488661623	23.23653028	0.528533955	0.621717939	0.755113359	-7.306519817
DNAJA1	0.488622081	25.63366538	2.313150822	0.073426915	0.252581018	-5.322223419
UQCRC1	0.4878932	25.73418873	4.01913715	0.012104162	0.091714195	-3.28915262
PLOD3	0.486860596	14.69004119	0.050125474	0.962133884	1	-7.469635943
CUTA	0.482907694	25.48431253	2.28173208	0.076224246	0.256131486	-5.362802544
SLC25A6	0.481763352	27.45736852	1.955702404	0.113134224	0.283237697	-5.784736254
SCFD1	0.480859962	22.20358334	2.712297874	0.046223543	0.198741724	-4.812780036
C8orf33	0.476277057	22.25046881	1.641179086	0.167013758	0.369495044	-6.185895971
SFXN1	0.475052145	24.78310877	3.801547299	0.014851993	0.101013668	-3.524834965
MSN	0.473895313	27.3444152	3.947674875	0.012935389	0.093193597	-3.365747823
RPL11	0.473831021	26.92564244	0.970121818	0.380382579	0.560470745	-6.951575068
RBMX	0.473822466	23.83448646	3.242312121	0.025979909	0.137260517	-4.164308824
PARK7	0.473661891	27.58022425	3.060449324	0.031497776	0.157546231	-4.382481254
LMAN2	0.473086629	24.34376176	1.210693965	0.284757574	0.524379301	-6.699321793
RO60	0.472334452	23.60373507	1.529728325	0.191884013	0.407065607	-6.32417757
GPS1	0.467806415	22.92220361	0.831151919	0.447034082	0.616895913	-7.08054403
PGP	0.46594493	22.88203845	1.216385459	0.282781415	0.5220399	-6.692981013
TPM3	0.465051594	26.83474953	4.372940623	0.008806376	0.075260205	-2.921416229
PLK1	0.463510525	23.04985733	4.657824994	0.006901619	0.065351182	-2.638820634
MATR3	0.463468659	24.45847503	3.830581033	0.014446141	0.099552756	-3.492962027
ATP5PO	0.462673716	25.47066118	3.807748999	0.014764197	0.100805392	-3.518015773
BOLA2B	0.462323323	24.75134739	1.491031771	0.20135444	0.421514626	-6.371482984
ISOC1	0.460661909	23.64195361	1.802696854	0.136642876	0.323252178	-5.981365068

MT-ATP6	0.460537622	24.443269	2.26130675	0.078105085	0.257646807	-5.389204821
PREB	0.459292508	7.424505282	0.046783824	0.964655934	1	-7.469832485
KRTCAP2	0.457292926	23.69170354	1.225058011	0.279794324	0.518544617	-6.683290036
ECH1	0.454390384	23.80002369	2.317688873	0.073032236	0.252008826	-5.316365969
GRWD1	0.452427285	23.43043375	3.754971731	0.015531145	0.104119668	-3.576239575
SEC22B	0.45217249	23.62685775	2.818406785	0.041039364	0.185066934	-4.680003119
DNPEP	0.452164194	22.80506077	1.231547802	0.277578011	0.516465703	-6.676015443
CREB5	0.448379859	7.610221193	0.044559742	0.966334786	1	-7.469955792
HSD17B10	0.448192963	26.10199528	2.569376676	0.054406878	0.220308142	-4.993593909
RBM8A	0.443807344	24.31736767	4.493929161	0.007930087	0.070955091	-2.799958708
UQCRC2	0.442971737	25.24339296	2.956108508	0.035262877	0.169368669	-4.509778319
TUFM	0.442631609	27.00347563	2.755934918	0.044007191	0.191850602	-4.758015121
WDR1	0.441580754	25.64270449	2.549793437	0.055649206	0.221818279	-5.018527875
RER1	0.441143116	24.11487136	3.478951187	0.020382828	0.119075217	-3.887819281
ANXA5	0.434918663	24.98131542	5.217624746	0.004403334	0.050987939	-2.116576812
EDF1	0.433761818	25.2862717	1.320449692	0.248810788	0.485067254	-6.574514261
RPS15	0.430037322	26.01942973	0.561453052	0.600838729	0.739058282	-7.28606367
PPIF	0.429785723	24.70180186	0.876119119	0.424522241	0.598104669	-7.040348361
TMX1	0.426478394	23.55661156	2.796716006	0.042043473	0.187322612	-4.707036152
BCLAF1	0.42642299	22.75259863	2.040836776	0.101937397	0.268644992	-5.674672697
P4HB	0.423534983	26.41986353	2.583441075	0.05353371	0.217965692	-4.975708881
SF3A1	0.423426143	24.30540129	2.097840063	0.095104757	0.263614246	-5.600857213
DDX5	0.423302901	26.66797642	3.624445583	0.017635667	0.112452715	-3.722102075
GSTO1	0.42147938	26.3734811	3.275703605	0.025091835	0.134058879	-4.124779543
MTPN	0.419625225	26.49685561	1.183425459	0.294401321	0.531148164	-6.729486735
FAH	0.417683443	24.09851014	1.256344198	0.269257962	0.508575784	-6.648045309
SF3B2	0.415150838	24.2452691	4.324730343	0.009187011	0.076928213	-2.970415648
IGF2R	0.414861934	23.62838797	1.995480801	0.107748144	0.274717623	-5.73334698
PDIA6	0.414292298	26.30280122	3.31663738	0.024050367	0.131447694	-4.076550164
LMAN1	0.412955969	25.26383372	1.845939879	0.129521426	0.312400048	-5.926006272
RPS6KA3	0.412505798	14.94881568	0.041748239	0.968457344	1	-7.470103079
UBA1	0.412249897	25.79233023	4.235365072	0.009945018	0.079881106	-3.062161787
PDCD5	0.411724806	24.63836783	0.658784812	0.541695853	0.684523404	-7.219410892
CLUH	0.408865458	23.53579419	2.066323895	0.098819611	0.265979027	-5.641675666
DNPH1	0.408602784	23.99207536	1.731290031	0.149303062	0.346189649	-6.072281006
G3BP1	0.408157541	25.90557653	2.671523484	0.048408299	0.204337029	-4.864146101
RAB13	0.407474227	25.38466787	2.42272063	0.064517927	0.237816078	-5.181116434
TBC1D10B	0.402958561	7.321667226	0.041626235	0.968549459	1	-7.470109254
YWHAE	0.40202401	28.27626069	1.520317453	0.194145904	0.409797753	-6.335718836
MAGEB2	0.401777409	23.64263388	2.004108936	0.106616183	0.273188579	-5.722190917
LARS1	0.401171627	25.1832145	3.481010089	0.020340641	0.119075217	-3.885451296
YWHAZ	0.400880908	28.54488778	2.78859932	0.04242631	0.188287962	-4.717166648
VTA1	0.400310938	23.66404058	0.986215732	0.373217893	0.555074065	-6.935770739
MT-CO1	0.397849797	25.80003622	1.373617421	0.232971776	0.46432684	-6.512298812

FSCN1	0.397780144	27.31334946	2.816714593	0.041116719	0.185066934	-4.682110007
HNRNPH1	0.395520081	26.90405025	4.724379193	0.006529517	0.064277112	-2.574481086
POLR2G	0.39342248	7.206199566	0.041292556	0.968801394	1	-7.470126048
HDLBP	0.393224505	23.32175882	3.072297839	0.031099952	0.156133019	-4.368121805
PDHB	0.392054567	23.36740567	0.957292519	0.386175518	0.565252952	-6.964050819
FLNB	0.391909434	24.74918061	1.324629134	0.247529633	0.484380598	-6.569662361
ASNS	0.390339237	25.68558757	2.528425466	0.057040873	0.22439954	-5.04577404
NDUFS1	0.389793488	22.77059895	2.628427975	0.050844009	0.212025573	-4.918630695
HK1	0.387459061	22.97316922	2.398872301	0.066350728	0.240890331	-5.211766574
RPN1	0.384607492	24.75716793	2.376963824	0.068085076	0.244072348	-5.239956323
TAGLN2	0.384337868	27.9720868	2.58521914	0.053424437	0.217920637	-4.973449145
AAGAB	0.383633421	15.16208467	0.038253206	0.971096358	1	-7.470272806
RHOT2	0.382606796	23.23225389	2.893117413	0.037782579	0.176133492	-4.587342182
HSPD1	0.3802182	28.63962027	3.320865577	0.023945651	0.13119852	-4.071582798
PPP2R1A	0.380170668	25.79854511	2.762388595	0.043689662	0.191217674	-4.749934439
POLD2	0.378953052	14.73566614	0.038906292	0.970603195	1	-7.470242217
MRPS6	0.378207233	22.87340222	0.941080529	0.393599858	0.571595605	-6.979657433
SEPTIN6	0.376539651	23.94182145	2.567257498	0.054539814	0.220358652	-4.99629039
ATP5F1D	0.374632532	25.52550564	0.811772933	0.457015865	0.623688933	-7.097386457
CTBP1	0.374627273	23.33891043	1.474428522	0.205557513	0.425165331	-6.391653325
AKR1A1	0.374486037	23.16380159	1.96769937	0.111480391	0.280540174	-5.769245775
STK26	0.370602104	23.33044569	1.667892717	0.161551041	0.361372741	-6.152362855
EIF3F	0.369973881	24.7984178	2.762911544	0.043664046	0.191217674	-4.749279867
CSNK2B	0.36983648	24.69122452	2.514844766	0.057945442	0.22439954	-5.063111641
CISD2	0.367013391	23.76713809	1.411990868	0.222143598	0.449759711	-6.466764834
MMGT1	0.365931175	7.701268527	0.035941579	0.972842048	1	-7.470376921
RPL13A	0.364953223	26.18019165	2.008026968	0.106106339	0.272931743	-5.717123991
ARL6IP4	0.36493737	7.425226506	0.037175692	0.97191005	1	-7.470322144
SLC19A1	0.364015277	7.362751637	0.037396252	0.971743489	1	-7.470312159
POLD1	0.362003282	23.1178847	0.316324674	0.765657964	0.875319435	-7.411057147
CLPP	0.358192759	23.93577831	1.923466289	0.117707819	0.290537986	-5.826317936
HSPA1A	0.35755684	29.77223479	2.920470207	0.036664173	0.172375113	-4.553596971
USP5	0.356006768	24.19404082	1.709033071	0.153491078	0.35040813	-6.100470443
PSMC3	0.355820739	25.26681246	2.437402182	0.063417419	0.236112842	-5.162267092
HIKESHI	0.35527843	7.331640368	0.036654002	0.972304023	1	-7.470345525
HSPB1	0.35498545	27.99460172	3.61711862	0.017763303	0.112619338	-3.730368706
HSPE1	0.354305572	27.33248573	1.297188082	0.256056208	0.494478246	-6.601392061
HSPB11	0.353766397	24.07319853	2.061889483	0.099354652	0.265979027	-5.647417702
TPD52L2	0.353583075	24.58688947	1.608119545	0.174033474	0.378237296	-6.227201033
CAB39	0.351801766	22.96066694	0.598501327	0.577861686	0.718980556	-7.261760894
PGK1	0.351143169	27.71531917	2.795836193	0.042084782	0.187322612	-4.708133867
PRKCSH	0.349233861	25.91209813	3.508869279	0.019779621	0.118624266	-3.853474357
ERH	0.348039987	25.0008089	0.710474327	0.511928485	0.661216128	-7.180440341
SND1	0.348014832	26.04027662	2.127544019	0.091740477	0.261829082	-5.56237788

SORD	0.34777691	23.47610835	1.621544299	0.171147743	0.37394601	-6.210454904
UGP2	0.347470118	23.20635444	1.301056058	0.254837842	0.493012355	-6.596937855
RAB7A	0.345767771	24.79727685	1.530896897	0.191604975	0.406973212	-6.322742862
RANBP2	0.345315368	23.75551205	2.521938696	0.057470966	0.22439954	-5.054053283
TMEM33	0.343971476	23.70399522	1.405982259	0.223806526	0.452301167	-6.4739277
TIMM13	0.341625602	24.6769955	1.688024334	0.157554445	0.35553268	-6.127005987
ANP32E	0.341471823	25.02427216	0.690145445	0.523496649	0.67107976	-7.196052194
CLINT1	0.334864834	23.07525603	2.477347284	0.060526749	0.230224616	-5.11106305
ACAT1	0.333707825	24.95768434	2.331990051	0.071803565	0.251312478	-5.297913465
BZW2	0.332768467	24.6120117	1.417696307	0.220575619	0.44822097	-6.459952343
CHMP4B	0.331001043	23.8328087	1.663542908	0.162428034	0.362603428	-6.157832166
IDI1	0.329116384	23.59915818	0.550378782	0.607814757	0.746397868	-7.293066208
TOMM70	0.322530572	24.12883784	1.474416781	0.205560516	0.425165331	-6.39166756
DHCR7	0.3220744	23.85838335	2.687061113	0.047562397	0.202480863	-4.844550808
REXO2	0.320778881	23.77308393	2.293574875	0.075156393	0.255087889	-5.347501833
OTUB1	0.319393276	24.17073554	1.483180524	0.203331351	0.424133321	-6.381030818
DNAJA2	0.319334679	24.43483335	2.020609934	0.104486417	0.271706248	-5.700847506
PPP1R14B	0.318317357	24.31996515	1.354850622	0.238449758	0.472466961	-6.534379232
ENO1	0.317347394	30.1276117	3.175936684	0.027854522	0.143076815	-4.24337733
ZC3H18	0.31424486	23.63761308	0.96125937	0.384376577	0.564108218	-6.960205032
IDH3A	0.314042317	23.92644256	1.267481832	0.265596518	0.504587906	-6.635393874
PRPF19	0.310954258	25.14048367	1.423687578	0.218940607	0.446124158	-6.452787126
C9orf78	0.310669826	23.78705543	1.07336797	0.33637145	0.531148164	-6.847353667
PPP1R12A	0.3085834	14.50170915	0.032194688	0.97567199	1	-7.470531907
PSMD5	0.308546175	24.79830881	2.064509897	0.099038108	0.265979027	-5.644024623
ELP1	0.308545298	22.55414931	0.593626492	0.580852888	0.720573535	-7.265034929
EEF2	0.306879904	28.80299142	1.581177284	0.179973203	0.388483012	-6.260691909
PRPSAP2	0.306574339	7.152994089	0.032421166	0.975500924	1	-7.470523023
PIN1	0.304590969	23.42657654	0.743411989	0.493571922	0.646920316	-7.154380205
HIGD2A	0.300491208	8.029171676	0.028311875	0.978605029	1	-7.470674539
EIF3B	0.299324208	25.91682065	2.023398204	0.104131033	0.271706248	-5.697240053
CLIC4	0.29785011	25.00650411	3.172124733	0.027966803	0.143321792	-4.247937971
HSP90AA1	0.297605287	30.42719378	3.179991046	0.027735667	0.142796854	-4.238529006
RPS16	0.296434543	27.19876227	1.868962318	0.125888821	0.305631612	-5.896455943
PTRH2	0.296375486	23.3495409	1.791759251	0.138507761	0.326619258	-5.995333794
MRPS18B	0.295581145	23.33529926	1.638848414	0.167499144	0.370199802	-6.188815158
PTPA	0.293573993	24.16214823	2.51495624	0.057937953	0.22439954	-5.062969265
HNRNPH3	0.293269722	23.78129791	2.44780147	0.062650446	0.234437591	-5.148925069
TOMM7	0.292281206	23.82398169	2.076687893	0.097581117	0.265979027	-5.628254168
NUDT21	0.28983651	25.23559374	0.851365009	0.436802472	0.609600431	-7.062665744
SUGT1	0.288375413	24.32468842	1.663884828	0.162358922	0.362603428	-6.157402371
HEXB	0.286653775	15.24083043	0.028458555	0.978494221	1	-7.470669484
STIM1	0.286362012	21.58288953	0.706281761	0.514299422	0.662735434	-7.183689922
RPL10	0.283334452	27.0410008	1.7263089	0.150230061	0.346528591	-6.078596472

APOO	0.28007664	15.22194023	0.027838923	0.97896232	1	-7.470690662
TRAP1	0.27708306	29.38522614	1.994846004	0.107831929	0.274717623	-5.734167644
SEC24C	0.276140937	23.76809558	1.176342619	0.296954241	0.531148164	-6.737262651
CAPN1	0.2760606	25.12855539	1.374290687	0.232777502	0.46432684	-6.511504316
DENR	0.275668554	23.43902833	0.839016901	0.443031107	0.61366169	-7.073624897
ATG3	0.275153853	22.7719313	1.703734246	0.15450574	0.351639218	-6.107170109
PITPNB	0.275025203	23.47272507	0.773866839	0.477027967	0.637055355	-7.129464124
DPY30	0.274207371	23.67144349	0.669458881	0.535453625	0.679500156	-7.211560368
RPAP3	0.272388587	15.22899562	0.027060677	0.979550257	1	-7.470716602
ANXA1	0.270486832	28.48836022	2.145061399	0.089816669	0.261829082	-5.539684971
MRPL24	0.270374874	7.456039797	0.027432689	0.979269214	1	-7.470704294
RAB1A	0.268797527	26.92838349	2.699322191	0.046906568	0.200164758	-4.829106398
RAB1B	0.268797527	26.92838349	2.699322191	0.046906568	0.200164758	-4.829106398
LGALS1	0.268625166	26.33795064	1.884381	0.123515677	0.301196698	-5.876638619
PFDN4	0.268295533	23.58425336	0.283665727	0.789036523	0.893758062	-7.422725202
EIF2S3	0.263315144	26.05161083	2.377038705	0.068079064	0.244072348	-5.239859922
PNP	0.262061846	25.95358777	1.47607272	0.20513748	0.425165331	-6.389659409
PPP1CB	0.260991721	25.52754249	1.781801376	0.140228517	0.329626142	-6.008038593
CACYBP	0.260832621	27.1370317	2.942473237	0.035791711	0.170433059	-4.526523388
PTBP1	0.259837999	27.18446863	1.352080449	0.239268692	0.473628213	-6.537627654
UBE2I	0.258513773	25.07678681	1.03960134	0.35025986	0.539740714	-6.8821609
FKBP8	0.258019103	23.719802	0.733630892	0.498972902	0.650159054	-7.162216435
ELOC	0.257352489	25.12058696	1.120247835	0.317885879	0.531148164	-6.797942435
PAFAH1B1	0.256010764	23.85275663	2.005945837	0.106376828	0.272931743	-5.71981545
ABCE1	0.255387025	25.62481853	1.73819427	0.148027865	0.343883808	-6.063521132
THYN1	0.25462665	7.098110747	0.027137596	0.979492147	1	-7.470714071
GMPR	0.251257514	23.96027359	0.659965938	0.541002709	0.684037044	-7.218547323
UBQLN2	0.250961665	23.79713631	1.121535961	0.317390835	0.531148164	-6.796567596
PRCC	0.249624824	14.79187624	0.0255358	0.980702292	1	-7.470765297
RAB2A	0.249322608	24.39052823	1.741735652	0.147378126	0.342800903	-6.059025218
AK2	0.24924902	26.34460326	0.76888638	0.479705183	0.637802859	-7.133591705
PDXK	0.248994103	23.7983228	0.582705392	0.587589627	0.727481464	-7.272286322
EIF4G2	0.248520816	23.9107685	1.976035237	0.110346338	0.278882146	-5.758478106
ZPR1	0.247402997	22.95240083	0.77502382	0.476407627	0.63683646	-7.128502339
ATP6V1A	0.247165064	24.47674024	1.881814721	0.123907388	0.301811739	-5.879938415
YWHAQ	0.247054498	28.10880549	1.119795707	0.318059799	0.531148164	-6.798424787
SNRPA1	0.244697046	24.08811216	1.630504036	0.169248663	0.371844341	-6.19925777
SRP54	0.244363246	22.56274038	0.847498743	0.438745327	0.611318596	-7.066109663
FHL3	0.243422194	24.18388193	0.801124269	0.462572619	0.629432916	-7.106514847
PSMD4	0.241633852	24.86270023	1.336032177	0.244065813	0.480125922	-6.556390193
CIAPIN1	0.241184462	24.05025314	1.69165969	0.156843545	0.355138598	-6.122419546
FKBP4	0.239995028	25.83568487	1.995754035	0.107712102	0.274717623	-5.732993738
SEH1L	0.239846135	23.52581316	1.088909963	0.330141209	0.531148164	-6.831109248
PPP1CC	0.238126918	25.53897489	1.793357992	0.138233535	0.326619258	-5.993292894

SERPINB6	0.237984747	24.47293878	2.094500653	0.095491189	0.264208166	-5.605182851
LAMP2	0.237978346	23.9328991	0.569588189	0.595745513	0.734014044	-7.280842271
ACTR2	0.235924275	24.4130649	1.136280351	0.311772915	0.531148164	-6.780767225
COPS2	0.234739046	23.06238158	0.542276359	0.612949643	0.749385817	-7.298112164
HNRNPA3	0.234202103	25.85174043	1.727816123	0.149948942	0.346319292	-6.076685893
RNH1	0.231152204	25.75781175	2.281020998	0.076288888	0.256131486	-5.363721435
DRG1	0.23087864	23.87545283	1.621094322	0.171243683	0.37394601	-6.211016815
HYOU1	0.229173158	25.16647849	1.904890609	0.120431428	0.295616525	-5.850247681
PGD	0.227547541	27.33257131	1.673024931	0.160522485	0.360161167	-6.145905366
NDUFB11	0.227272327	24.43166894	0.462388741	0.664928753	0.79198975	-7.344281558
GORASP2	0.22690207	23.9005517	0.82589428	0.449725522	0.617965301	-7.085142592
PSMD10	0.226849218	23.79116239	0.44780107	0.674674308	0.799307148	-7.351994105
FDPS	0.226358126	25.6430519	1.772951795	0.141776303	0.332911763	-6.019318853
CSE1L	0.226319624	25.68473966	2.598115598	0.052639278	0.216519388	-4.957068249
SH3BGRL	0.225566724	23.16509039	1.072808985	0.336597424	0.531148164	-6.847935331
PPP1CA	0.225345184	25.54536576	1.648619241	0.165473769	0.367553848	-6.176570051
EIF4A2	0.222983795	27.81685514	1.826213378	0.132720943	0.316334878	-5.951284679
MAP2K2	0.220335469	23.04395227	0.503355344	0.637972807	0.769381337	-7.321426837
DDX17	0.22027926	26.50645635	1.526061804	0.192762139	0.407758995	-6.328676838
MYL6	0.219351597	24.73476644	1.454024043	0.210840617	0.433199379	-6.416331584
BAG6	0.219126778	24.28499372	0.83596583	0.444580685	0.615040237	-7.076314727
RBM4	0.218684693	24.13863291	0.393996575	0.711250964	0.829792791	-7.378465864
DYNC1LI1	0.214770801	23.5867369	1.526289479	0.192707495	0.407758995	-6.328397554
DDX19A	0.212032162	23.99227406	1.954920578	0.113242899	0.283237697	-5.785745475
TIMM50	0.210492631	24.16851196	1.392534087	0.22757199	0.457826152	-6.489915357
EIF1AX	0.209370058	24.666205	1.548313187	0.187493606	0.401979045	-6.301319106
GNB2	0.20895425	24.17408096	1.527916014	0.192317562	0.407595674	-6.326401931
NUP58	0.207298882	23.24289078	1.18929531	0.292300681	0.531148164	-6.723023779
APEX1	0.204744713	25.02913896	0.634203257	0.55625726	0.698943862	-7.237091413
YWHAB	0.203007266	28.0987128	1.072681506	0.336648976	0.531148164	-6.848067956
ZNF207	0.202274722	24.16946145	0.833747118	0.445710152	0.616126682	-7.078266228
VDAC2	0.200796493	26.62230743	0.8445683	0.440222392	0.611680331	-7.068712385
USP10	0.200315438	23.27587433	0.470982056	0.659223406	0.787307178	-7.339632912
HEBP2	0.19961127	23.13594844	0.657093533	0.542689426	0.684998769	-7.22064523
RRM1	0.19957001	24.60112154	1.710916442	0.153132078	0.34994859	-6.098088078
ARPC1B	0.198558779	23.48617879	1.0933833	0.328366797	0.531148164	-6.826408271
RHOA	0.196966124	25.3837239	0.593484621	0.580940087	0.720573535	-7.265129869
RPL19	0.195937459	27.11990013	0.855837031	0.434563591	0.607622311	-7.058668062
AFG3L2	0.194705925	22.92626784	0.416216579	0.696028218	0.81762129	-7.36791353
DNAJC2	0.191112237	23.42453761	0.923085482	0.401977248	0.578089539	-6.996769166
FAU	0.188447522	26.8161292	0.498607366	0.641065084	0.7718521	-7.324165062
SLC7A5	0.187457201	23.9193988	1.144157358	0.308808029	0.531148164	-6.772278823
KRT18	0.186953223	25.06200905	1.304323891	0.253812756	0.492305779	-6.59317002
SNAP29	0.186538551	24.79968115	0.842108187	0.441465371	0.612887814	-7.070892297

HNRNPM	0.18641975	26.0553964	1.687911168	0.157576628	0.35553268	-6.127148723
HBG1	0.185908385	28.60366806	1.634142859	0.168483474	0.370530058	-6.194705649
HBG2	0.185908385	28.60366806	1.634142859	0.168483474	0.370530058	-6.194705649
TMEM14C	0.183334614	25.09932889	0.932742891	0.397463462	0.574574216	-6.987613725
DCTN1	0.182728014	23.37949297	0.879062862	0.423080177	0.59705105	-7.037663976
CNBP	0.182483409	25.7897824	0.996869188	0.368537589	0.552423998	-6.925216293
TXN	0.182012165	27.41246218	1.111142046	0.321404875	0.531148164	-6.807635503
GOT2	0.181823225	24.9179005	0.945890067	0.391385175	0.569763057	-6.975046146
PIH1D1	0.181808808	23.61301785	0.92226255	0.402363799	0.578089539	-6.997546313
CKAP5	0.181476516	23.72913883	1.533777474	0.190918852	0.406572872	-6.319204755
NAPA	0.181451366	23.53138357	0.61923198	0.565251725	0.707042603	-7.247583459
XPO7	0.178374411	23.52201268	0.610288273	0.570669959	0.712614878	-7.253750238
RPS4X	0.178191238	27.63461533	2.028387211	0.103498361	0.27114534	-5.690784672
TIPRL	0.177131715	23.40394519	0.403912339	0.704437856	0.824010333	-7.373823408
RPS10	0.176928107	26.33110824	0.390105312	0.713933268	0.832484457	-7.380258266
ATP5F1B	0.17651886	27.82838	1.826715358	0.132638512	0.316334878	-5.950641923
ATIC	0.176441531	26.07445632	1.414897244	0.221343522	0.449369876	-6.463295857
TSR1	0.176254169	23.40357703	1.334649265	0.244483433	0.480521468	-6.558002445
NPM1	0.174417306	29.30889979	1.333170651	0.244930702	0.480974538	-6.559725462
RPS21	0.173217107	26.17381547	0.592477633	0.581559258	0.720938544	-7.265803186
CIAO2B	0.172377249	15.15916854	0.01720202	0.986999289	1	-7.47098155
SF3B4	0.172133427	24.62784819	0.533939261	0.618260281	0.753801957	-7.303235543
NAP1L4	0.171774465	25.72727393	0.263478635	0.803619286	0.904734245	-7.429323909
PFDN2	0.168886303	24.55577336	0.657480254	0.542462131	0.684998769	-7.220363222
ADRM1	0.16883422	24.05611618	0.493960288	0.64409985	0.773826512	-7.326822554
TLN1	0.167847859	25.51242194	0.882502698	0.421400012	0.595596578	-7.034519109
DNM2	0.166121884	24.33068033	1.419444993	0.220097188	0.448069413	-6.45786222
ETFA	0.163177372	25.25422901	0.840212329	0.442425114	0.613205076	-7.072569048
RPS5	0.16270989	27.44585854	0.943159568	0.39264125	0.570576905	-6.977666035
SLC3A2	0.162673189	24.84961759	1.072127808	0.336872972	0.531148164	-6.8486439
PFKP	0.162531522	24.63368943	1.32591034	0.247138145	0.484380598	-6.56817365
PKM	0.162213228	27.79895476	0.754211605	0.487657934	0.641586878	-7.145633638
PSME1	0.161522935	23.83295747	1.357239804	0.237745599	0.472298553	-6.531575307
CCT7	0.161319123	27.07995158	1.185796739	0.29355107	0.531148164	-6.726877887
SYNGR2	0.159468218	23.16726758	0.261827603	0.804816177	0.90562226	-7.429842719
EIF3L	0.159094642	24.7886778	0.984958689	0.373773414	0.555527934	-6.93701126
ETFB	0.15790353	25.16108103	0.921273046	0.40282899	0.578089539	-6.998480132
GNB1	0.157700607	24.06169564	1.409465316	0.222841106	0.450760633	-6.469777006
PRDX3	0.15349922	26.39588754	1.061980876	0.341000837	0.532873843	-6.859166908
FARSB	0.152731085	24.2447065	1.065182959	0.339693489	0.53195473	-6.855852638
POLR2B	0.152447945	23.09526943	0.449970816	0.673220008	0.798756713	-7.35086126
RBM39	0.151087588	24.78360862	1.227576393	0.278932355	0.518384334	-6.680469402
PRMT1	0.150529801	25.65655972	1.727632005	0.149983254	0.346319292	-6.076919303
CAP1	0.148383728	26.06645874	1.010898017	0.362449881	0.548245593	-6.911207148

AGTRAP	0.147380156	23.34537514	1.679200516	0.159293646	0.358491481	-6.138128846
TOP1	0.146061427	24.53227431	0.460640541	0.666092681	0.792525287	-7.34521773
CDC5L	0.145437867	22.76084566	0.472470176	0.658238111	0.786977569	-7.338820004
NELFE	0.145280943	22.20032264	0.937341457	0.395328702	0.572937634	-6.983231416
NOMO3	0.145235815	22.51765581	0.29218485	0.782912012	0.889060089	-7.419799421
GOT1	0.144945754	25.23076049	1.167186722	0.300283989	0.531148164	-6.747277447
SGTA	0.144706101	24.40038657	0.486138585	0.649225993	0.778299557	-7.33124495
HADH	0.14328779	24.46046885	0.358435382	0.735939255	0.85126707	-7.39422417
METAP2	0.141689442	23.07724971	0.430563772	0.686286003	0.809175686	-7.360815135
HNRNPD	0.140835361	27.12875159	0.471672433	0.658766202	0.787184815	-7.339256072
ACTN1	0.14067886	25.71407118	0.799223794	0.46356969	0.630307072	-7.108134462
CBFB	0.138527229	23.50654012	0.979675063	0.376115958	0.557312575	-6.94221419
CLIC1	0.137837871	28.89077429	0.542992043	0.612495035	0.749242823	-7.297669102
MYDGF	0.136015275	23.65823793	0.492847391	0.644827824	0.773863138	-7.327455661
MDH1	0.135580955	27.26980153	1.444667763	0.213307212	0.437861891	-6.427605672
DNAJC11	0.134912369	23.29071466	0.748170918	0.490959483	0.644257299	-7.150538112
SEPTIN2	0.134299868	25.00023089	0.840532819	0.442262758	0.613205076	-7.072285792
LSM4	0.133399784	23.60225381	0.46144847	0.665554637	0.792309946	-7.34478548
ALDOC	0.132964951	28.67252446	1.106459094	0.323227993	0.531148164	-6.812602818
RCN2	0.130628785	7.412086389	0.013334079	0.989922308	1	-7.471053268
RPS24	0.128947599	26.44508009	0.578829831	0.589992042	0.729355065	-7.274831744
ATOX1	0.128602971	24.19284318	0.414590201	0.697136939	0.818490406	-7.36870414
SEPTIN7	0.128409746	24.32501198	0.866341807	0.429339789	0.602756087	-7.049218104
ARMC6	0.127632578	14.78043874	0.013051713	0.9901357	1	-7.471057792
U2AF2	0.127192356	25.60907152	0.741724932	0.494500459	0.647754733	-7.155737628
TUBG1	0.123282361	23.40539108	0.478861343	0.65401564	0.782772764	-7.335302323
ANXA11	0.123036474	24.9926937	0.389234328	0.714534309	0.832747706	-7.380657182
MICOS13	0.122811367	23.64798655	0.175537265	0.868150647	0.947502671	-7.452513267
WDR6	0.122646006	7.522202251	0.012336043	0.99067656	1	-7.471068824
HSD17B4	0.119593725	24.73221204	0.570857067	0.594953508	0.73385316	-7.280021987
UGGT1	0.119107974	23.10944736	0.281234206	0.790787851	0.895284817	-7.42354495
EIF3H	0.118402618	23.90111879	0.489524395	0.647004229	0.776055343	-7.32933841
ALDH1A2	0.117578517	25.69985997	0.428915753	0.687401524	0.810060532	-7.361641799
CALU	0.117275486	25.27274204	0.954669986	0.387368635	0.566625577	-6.966587505
EPB41	0.11443014	23.81663645	0.598217223	0.578035742	0.718980556	-7.261952332
HSP90AB1	0.114035974	30.51897844	1.04542023	0.347831848	0.536744694	-6.876211053
G6PD	0.113405027	25.97872023	0.846717888	0.439138533	0.611322714	-7.066803835
ARF5	0.112278867	25.9230075	0.735045235	0.498189296	0.649901263	-7.161088372
EEF1G	0.110883653	27.48643736	0.915447937	0.40557637	0.580440797	-7.003963463
SBDS	0.110714113	23.76618154	0.092302421	0.930357162	0.991495591	-7.4659926
TUBB6	0.108969229	29.49467785	0.720922504	0.506053631	0.655817724	-7.172275305
UBFD1	0.108546844	7.366502188	0.011148729	0.99157387	1	-7.471085756
BLVRB	0.106514385	27.77394944	0.574648985	0.592590568	0.731387051	-7.277561226
SSRP1	0.105523679	23.16207974	0.612196602	0.569511052	0.711969028	-7.252440807

PPIB	0.104301439	26.40213106	0.549598246	0.608308287	0.746590757	-7.293555159
DBNL	0.103852018	23.21198942	1.110881913	0.321505909	0.531148164	-6.807911748
CBFA2T3	0.10376929	7.129943744	0.011011628	0.991677485	1	-7.471087601
POLR1G	0.102928073	22.2882923	0.29166339	0.783286378	0.889060089	-7.419980907
TRMT112	0.102641113	24.77105513	0.433023443	0.68462281	0.807658433	-7.359575903
TM9SF2	0.101055369	23.3447555	0.264563975	0.802832825	0.904307634	-7.428981134
CCDC183	0.101001921	8.233559744	0.009281498	0.992985056	1	-7.47110892
TARS1	0.098989509	26.19852765	0.629866545	0.558852927	0.701106668	-7.240152311
MRPL41	0.098961535	14.79682268	0.010108334	0.992360159	1	-7.471099185
RPL22	0.098743577	27.70726159	0.355136888	0.738248721	0.852327738	-7.395614646
NNT	0.098623329	23.11391043	0.293051109	0.78229026	0.889060089	-7.419497243
DDX54	0.097987397	7.402801855	0.010014886	0.992430784	1	-7.471100327
TP53BP1	0.097708602	7.132974088	0.010364116	0.992166848	1	-7.471096005
CPSF6	0.09713516	23.98388178	0.642228281	0.551475086	0.694114132	-7.231380917
COX6B1	0.094906513	25.10518515	0.198086179	0.851465455	0.938133985	-7.44743641
CALM1	0.0931287	27.7062476	0.540182702	0.61428071	0.750599612	-7.299405345
TBL1XR1	0.092004384	14.80500985	0.009399113	0.992896166	1	-7.471107586
PDS5A	0.091534555	22.43323465	0.433005106	0.684635202	0.807658433	-7.359585166
USP14	0.089298363	25.02942372	0.503954684	0.637583072	0.769329438	-7.321079538
MTCH2	0.088873289	24.60982378	0.315316237	0.766375689	0.875553051	-7.411435665
PSMC6	0.087880206	25.21107862	0.783194153	0.472043977	0.634874809	-7.121679134
RPS25	0.08642782	26.8932254	0.537587326	0.615933145	0.751790786	-7.301002309
PCBP3	0.085912093	27.1082026	0.595141738	0.57992208	0.72051909	-7.264019714
UBE2S	0.08480929	22.80046753	0.271517439	0.797800668	0.902131325	-7.42675268
EIF4A3	0.083553883	26.79358085	0.784031508	0.471598447	0.634874809	-7.120976755
AIFM1	0.081130564	24.83361436	0.521166849	0.626448802	0.760027279	-7.310948432
RPS27A	0.081007309	27.24168877	0.67989656	0.529397317	0.675910614	-7.203783649
AIMP1	0.080542789	25.14602941	0.77524209	0.476290664	0.63683646	-7.12832077
SERBP1	0.079897498	26.87468972	0.531204434	0.620008267	0.754275408	-7.304900919
ATP5F1A	0.079161395	26.94895502	0.452451707	0.671559188	0.798159539	-7.349559825
SEPTIN9	0.078478886	24.41182949	0.362368973	0.733189351	0.849580767	-7.392550088
GTSF1	0.075067091	24.2925635	0.623869427	0.562455516	0.704736753	-7.244356046
COPS6	0.074561189	23.19923544	0.495415499	0.643148657	0.773102313	-7.325992775
EDC4	0.073482006	23.81547364	0.313616381	0.767586127	0.876087184	-7.412071089
CAVIN2	0.072642438	22.65841912	0.531985756	0.619508581	0.754080933	-7.304425903
PCBP1	0.072450856	27.60687978	0.6875046	0.525012702	0.672246501	-7.198053362
RPLP1	0.071819393	28.25622377	0.234918841	0.824408317	0.91884081	-7.437850958
RPE	0.070724301	15.09669728	0.007087115	0.994643528	1	-7.471130731
AGPS	0.06996688	23.67602164	0.218336024	0.836557708	0.926308161	-7.442364217
UBE2L3	0.069120543	28.22765983	0.196339394	0.852754913	0.939088413	-7.447851244
EIF4A1	0.069092043	28.01033386	0.475002407	0.656563352	0.785398425	-7.337431391
RPL30	0.069080558	27.48318357	0.229877265	0.828096179	0.919247933	-7.439257248
RIOK2	0.066271709	23.29051292	0.50533701	0.6366847	0.768663411	-7.320277113
SUPT5H	0.066191147	22.75667963	0.638384212	0.553762377	0.696597911	-7.234123794

MAT2B	0.065942571	23.70779295	0.260353283	0.805885487	0.906365888	-7.430303326
KIF5B	0.065563049	23.64215602	0.449113093	0.673794711	0.798756713	-7.351309684
COX5A	0.06512073	26.08165851	0.356359316	0.737392454	0.852060747	-7.395100751
CCT4	0.064479461	26.97863685	0.463054793	0.664485593	0.791886966	-7.343924033
BAG2	0.064252196	24.2329287	0.270930877	0.798224722	0.902131325	-7.426942824
RUVBL1	0.063703123	25.18131544	0.49747733	0.641802307	0.77190207	-7.324813343
ERLIN2	0.06369903	23.08007604	0.274301442	0.795789086	0.900487498	-7.425844766
UBE2D3	0.063196205	25.20096955	0.132782864	0.899998857	0.97229672	-7.460475579
NUP43	0.062269676	23.86555448	0.644550226	0.550096539	0.693165372	-7.229717475
NUP88	0.061518404	23.27707824	0.312975023	0.768043027	0.876087184	-7.412309981
POLR2E	0.059643591	25.30012832	0.185912405	0.860462966	0.946168148	-7.450252283
COPB2	0.059633939	24.73207319	0.512321884	0.632156331	0.764945402	-7.316192461
ARIH2	0.059233804	7.174112789	0.006247096	0.995278407	1	-7.471137532
YWHAH	0.059035766	27.98351446	0.311053939	0.76941225	0.876900762	-7.413022738
SYNCRIP	0.058395479	26.28951432	0.533335683	0.618645811	0.7538578	-7.303603742
MARS1	0.056622215	25.49929894	0.322178796	0.761496881	0.872810733	-7.40883696
RPL18A	0.056561167	26.65261182	0.523013358	0.625261075	0.759001272	-7.309843608
MTREX	0.05419588	23.83518528	0.264941274	0.802559491	0.904307634	-7.428861655
ARPC2	0.053364843	22.82277249	0.222555943	0.833460797	0.924262623	-7.441246345
XRCC5	0.052946076	26.63944741	0.257880607	0.807680001	0.907923973	-7.431070169
PRRC2C	0.052079602	23.86811	0.399234987	0.707647703	0.826893235	-7.376026702
NELFA	0.051901826	14.90466567	0.005269363	0.996017375	1	-7.47114437
COPS3	0.051806618	23.02011561	0.178566699	0.865904128	0.947502671	-7.451866312
ZYX	0.051788846	24.08834021	0.319529881	0.763378569	0.874064523	-7.409846389
MAP2K1	0.051669453	23.32082091	0.076997208	0.941874544	0.99953114	-7.467563552
DDX3X	0.050808677	25.52716362	0.136397702	0.897296644	0.970795345	-7.459887051
DDX1	0.050552683	24.10770413	0.46689712	0.661932193	0.789578865	-7.341852403
ADH5	0.050495559	25.69498832	0.363209834	0.73260212	0.849343837	-7.392189992
SEC61A1	0.050164121	23.92097125	0.175333593	0.868301735	0.947502671	-7.452556371
PSMB1	0.04880182	25.72474734	0.247781394	0.81502353	0.912941551	-7.434128335
RAC1	0.048391216	25.30409094	0.230467804	0.827663937	0.919247933	-7.439094065
SLC25A21	0.047598395	22.61344888	0.293124773	0.782237396	0.889060089	-7.419471507
QARS1	0.046461498	24.26478734	0.292091403	0.782979095	0.889060089	-7.419831967
ELOB	0.046334371	24.23275804	0.294327936	0.781374168	0.889060089	-7.419050275
CSTF2	0.045629222	22.83231054	0.174215422	0.869131332	0.947502671	-7.452792134
UCHL3	0.045307062	23.48117356	0.340735209	0.748369457	0.860430997	-7.401542886
GAPDH	0.044736549	30.58640376	0.512213944	0.632226168	0.764945402	-7.316255963
RPS19	0.044477133	26.93662723	0.318805076	0.763893774	0.874203345	-7.410121204
SH3BGRL3	0.044307329	25.7547644	0.352262418	0.740263922	0.853765927	-7.396816455
NUP155	0.037544802	23.65933321	0.231589888	0.826842829	0.919247933	-7.438782873
CHCHD3	0.035389503	24.3214813	0.182052762	0.863320809	0.946964348	-7.451108334
GNL3	0.032556267	22.80826264	0.300537984	0.776924509	0.88500795	-7.41684972
PDAP1	0.03231602	24.85426457	0.115004084	0.913311211	0.981130406	-7.463141636
ATP5PF	0.031486658	24.77827314	0.184441957	0.861551459	0.946521752	-7.450580508

FLNA	0.03135685	26.36704558	0.342166297	0.747361082	0.859717077	-7.400964222
SLC1A4	0.030714965	23.21163565	0.11432527	0.91382018	0.981130406	-7.463235893
ACTC1	0.030211664	30.5188137	0.211326401	0.841709503	0.929693075	-7.444174751
TOMM20	0.029776958	23.81068116	0.15271712	0.885118056	0.960273025	-7.457035116
EXOSC3	0.029252413	22.88844025	0.230516769	0.827628101	0.919247933	-7.439080516
LDHA	0.029075067	28.96039821	0.236737094	0.823079566	0.918257193	-7.43733647
IRF2BP2	0.028258053	23.78314233	0.047741703	0.963932941	1	-7.469777531
CDV3	0.028180015	25.21192243	0.098648503	0.92558726	0.989820786	-7.465258231
TRIM28	0.027529458	27.28560065	0.265522376	0.802138573	0.904307634	-7.428677314
PPM1A	0.027362061	7.420097776	0.002790101	0.997891212	1	-7.471156503
CLTC	0.027243508	26.56449134	0.236989053	0.822895493	0.918257193	-7.437264871
TOR1AIP1	0.026549983	22.95523545	0.229858059	0.828110238	0.919247933	-7.439262548
NECAP2	0.025923023	7.587962603	0.002584889	0.998046312	1	-7.471157173
CAD	0.025850867	24.64227785	0.08149961	0.938484564	0.997612715	-7.467130818
HADHA	0.02504308	25.81453898	0.096778499	0.926992443	0.990696335	-7.46547968
PHAX	0.023791186	7.230862228	0.002489466	0.998118435	1	-7.471157467
PTBP3	0.023178617	24.51414894	0.123078269	0.907261037	0.977856957	-7.461977958
CHCHD2	0.022116215	24.52088633	0.133909316	0.899156625	0.97229672	-7.460293864
MCM6	0.019728766	24.97588454	0.174961454	0.868577812	0.947502671	-7.452635001
PYCR2	0.017951414	24.81130125	0.152329094	0.885407215	0.960273025	-7.457106627
ARHGEF2	0.017933375	23.05270128	0.102228084	0.922898328	0.987897439	-7.464822562
SAR1A	0.017919545	25.09703638	0.069115998	0.947811915	1	-7.46826199
MPDU1	0.017648618	15.50294854	0.001722652	0.998698	1	-7.471159428
FIP1L1	0.013856525	23.07017266	0.078022679	0.941102311	0.999189487	-7.467467148
UBA5	0.013466211	15.29295866	0.001331991	0.998993266	1	-7.471160153
TRIM25	0.011044929	23.1378371	0.041898475	0.968343915	1	-7.470095451
EIF3C	0.008634292	24.63606399	0.082548463	0.937695068	0.997480996	-7.46702649
COX4I1	0.008085199	25.64523523	0.037469361	0.97168828	1	-7.470308837
UBE2K	0.008024711	25.17459648	0.073708883	0.944351322	1	-7.467864104
STAT3	0.005185084	23.69470727	0.017121211	0.987060355	1	-7.470983235
PRRC2A	0.005084749	22.79096215	0.011139875	0.991580561	1	-7.471085876
GGCT	0.004727088	23.34151208	0.026063592	0.980303543	1	-7.470748762
UQCRFS1	0.003143068	23.87596323	0.017752691	0.986583162	1	-7.470969863
CUL3	0.002231048	7.380701896	0.000228714	0.999827135	1	-7.471161198
EHD1	0.001457828	24.43560166	0.004426632	0.996654312	1	-7.471149331
LDHB	7.11E-15	29.07990293	8.37E-14	1	1	-7.47116123
ACTB	0	31.08696243	0	1	1	-7.47116123
CCDC25	0	0	0	1	1	-7.47116123
CRYBG3	0	0	0	1	1	-7.47116123
MRPL3	0	0	0	1	1	-7.47116123
NELFB	0	0	0	1	1	-7.47116123
PRSS38	0	0	0	1	1	-7.47116123
STH	0	0	0	1	1	-7.47116123
RAB35	-0.001606493	26.59433445	-0.011980264	0.990945437	1	-7.471074078

NACA	-0.002307646	27.10588836	-0.01840431	0.986090759	1	-7.470955557
CNP	-0.003039457	7.645822901	-0.000300784	0.999772664	1	-7.471161175
ILF3	-0.00306617	25.03175219	-0.027558569	0.979174115	1	-7.470700092
PABPC4	-0.003816216	26.86578331	-0.026560908	0.979927824	1	-7.470732873
HSBP1	-0.004076468	24.24622714	-0.017912244	0.986462594	1	-7.470966408
UBL4A	-0.004923572	22.47365163	-0.012771914	0.990347154	1	-7.471062179
PSMD13	-0.005098325	25.42714533	-0.030177362	0.977195806	1	-7.470608296
TUBA1C	-0.005524218	30.46455796	-0.037218141	0.971877994	1	-7.470320227
RPL4	-0.005807021	27.6888364	-0.059182231	0.955301148	1	-7.469035196
HGH1	-0.01065204	23.06490776	-0.04061433	0.969313482	1	-7.470159768
YWHAG	-0.010653952	28.12810259	-0.061080794	0.953869347	1	-7.468896658
NAA15	-0.010875387	25.02879516	-0.085340019	0.935594208	0.995882144	-7.466742337
CBX3	-0.015123662	25.07416299	-0.073985061	0.944143275	1	-7.467839365
DNAJB11	-0.015537798	23.64104332	-0.065433768	0.950587327	1	-7.46856254
CD81	-0.015675965	24.04581808	-0.045508291	0.965618747	1	-7.469903936
ATP6V0D1	-0.015865093	23.23481778	-0.062270589	0.952972166	1	-7.468807613
IQGAP1	-0.015937002	24.95341997	-0.114224521	0.913895725	0.981130406	-7.463249836
STRAP	-0.015986476	25.18983918	-0.115089569	0.913247119	0.981130406	-7.463129726
RPL18	-0.016565345	27.2853781	-0.064712101	0.951131367	1	-7.46861952
SEC11A	-0.016686955	23.18095533	-0.121800726	0.908217856	0.978318166	-7.462167305
HARS1	-0.017320792	24.45085775	-0.080929701	0.938913581	0.997612715	-7.467186948
RHOC	-0.017543695	25.36045141	-0.066300006	0.949934342	1	-7.468493313
CSTF1	-0.018181622	7.500188772	-0.00183418	0.998613706	1	-7.471159187
RPS20	-0.018359103	26.81162624	-0.119261041	0.910120494	0.979416768	-7.462537885
MRTO4	-0.018815603	23.93827081	-0.137234803	0.896671112	0.970591804	-7.459748528
RPL9P9	-0.019463497	26.09051	-0.110592533	0.916619803	0.982657752	-7.463744291
LRRC59	-0.021770156	26.39468696	-0.092896139	0.929910758	0.991495591	-7.465925957
HNRNPF	-0.022358346	26.74477776	-0.166826961	0.874617869	0.951827882	-7.454312534
MPP1	-0.022462031	25.47982294	-0.233755255	0.825259006	0.91884081	-7.438178171
MAPK1	-0.023752533	25.64068887	-0.113262905	0.914616828	0.981399778	-7.463382295
RPS8	-0.024292765	27.78233644	-0.174904491	0.868620073	0.947502671	-7.452647022
HNRNPR	-0.025956439	25.59152736	-0.199760979	0.850229642	0.937237742	-7.44703528
EEF1B2	-0.026686491	27.51499156	-0.242471952	0.818893156	0.91513638	-7.435688406
PPM1G	-0.026825813	24.79110877	-0.206211255	0.845474765	0.933387315	-7.44545939
VDAC3	-0.027454854	25.58303937	-0.167133458	0.874390105	0.951827882	-7.454250757
RPS29	-0.0275611	24.59598872	-0.187680912	0.859154315	0.945666381	-7.449854127
RPS9	-0.028153745	26.78666426	-0.335554166	0.752024999	0.863738858	-7.403618564
SNRPA	-0.029140315	24.30937616	-0.128571262	0.903149152	0.975225289	-7.461141479
TKT	-0.031848093	26.92047346	-0.186862821	0.859759617	0.945863455	-7.450038771
NUDT4	-0.032600428	22.99603671	-0.249699272	0.81362724	0.912436293	-7.433556718
ARF4	-0.033495211	25.85451678	-0.239887795	0.820778717	0.916611965	-7.436435793
RPL23A	-0.035783105	27.01242492	-0.234696847	0.824570594	0.91884081	-7.437913508
SF3B5	-0.035919985	23.6617251	-0.095776676	0.927745374	0.990696335	-7.465596581
GAGE4	-0.036303998	27.41747188	-0.074905234	0.94345014	1	-7.467756272

RAE1	-0.036327309	23.78463597	-0.085141952	0.935743249	0.995882144	-7.466762809
SRSF6	-0.036786137	24.5990819	-0.122960785	0.90734902	0.977856957	-7.461995453
TAF15	-0.037384311	24.35231225	-0.125716454	0.905285743	0.977056938	-7.461580738
EFTUD2	-0.037402708	26.07254851	-0.244530705	0.817391978	0.914747511	-7.435087391
NDRG2	-0.039265683	23.72396497	-0.15111612	0.88631126	0.960783921	-7.45732901
NUDC	-0.039345403	26.53542942	-0.254389044	0.810216299	0.909905255	-7.432140889
PFN1	-0.03991825	28.66195761	-0.214023447	0.839726175	0.928889523	-7.443484977
PCMT1	-0.041108859	26.05385427	-0.176117277	0.867720418	0.947502671	-7.452390247
ASH2L	-0.042618511	7.623877228	-0.004229636	0.996803203	1	-7.471150367
TUBA4A	-0.0426707	30.25627693	-0.326336005	0.758547618	0.870329454	-7.407236714
UCK2	-0.044364491	23.32413715	-0.216453997	0.837940002	0.927375992	-7.442856015
DHFR	-0.044672389	23.94908537	-0.132891208	0.899917842	0.97229672	-7.460458167
SRPK1	-0.044894723	23.6444158	-0.160867104	0.879049538	0.954777741	-7.455491528
H1-2	-0.045081828	28.06901626	-0.356015155	0.737633482	0.852060747	-7.395245601
CRYZ	-0.047001613	24.18540372	-0.411549135	0.699212423	0.819626184	-7.370174747
GABARAPL2	-0.047145939	23.33037345	-0.15653769	0.882272071	0.957809064	-7.456321392
IPO7	-0.048180724	25.12937114	-0.183173448	0.862490754	0.946521752	-7.450861594
TACC3	-0.048285013	23.85779506	-0.233971848	0.825100635	0.91884081	-7.438117383
CTNND1	-0.051641243	22.96824569	-0.243917092	0.817839311	0.914747511	-7.435267042
RPLP2	-0.051786773	28.71332493	-0.538088307	0.615613972	0.751790786	-7.300694577
PSMC5	-0.05229801	24.77790011	-0.481042195	0.652578228	0.781473874	-7.334092205
CIAO1	-0.053331099	15.43947518	-0.00522242	0.996052855	1	-7.471144669
IARS1	-0.056131333	25.09710445	-0.530004536	0.620776112	0.754795722	-7.305629214
CPSF1	-0.056617061	22.26154007	-0.24387158	0.817872493	0.914747511	-7.435280349
PEX14	-0.057414299	22.40876217	-0.254325293	0.810262634	0.909905255	-7.432160307
CHD4	-0.057504341	23.66548474	-0.43667345	0.682158552	0.805593308	-7.357724978
DUT	-0.057748265	25.86182521	-0.232451023	0.826212851	0.919247933	-7.43854305
RPL10A	-0.058662233	27.15832444	-0.336978383	0.751019369	0.863030544	-7.403050996
MSH2	-0.059226094	23.27083653	-0.248257433	0.814676881	0.912941551	-7.433986853
GGH	-0.060515719	7.386248633	-0.006199005	0.995314754	1	-7.471137896
SPTA1	-0.061634784	22.97024235	-0.223172828	0.83300837	0.924222787	-7.441081176
BANF1	-0.061656344	24.84215494	-0.420901314	0.692839511	0.814738143	-7.365620178
UBA2	-0.062754958	25.06343271	-0.493335234	0.644508657	0.773863138	-7.327178294
RRM2	-0.064077235	23.8499054	-0.451931627	0.671907174	0.798159539	-7.349833193
IMMT	-0.06584564	24.98507123	-0.289971676	0.784501361	0.889529136	-7.420567531
CHORDC1	-0.06770147	25.59520873	-0.296913379	0.779520439	0.887509417	-7.418139484
VPS26A	-0.067783646	23.89674205	-0.183806728	0.862021794	0.946521752	-7.450721506
EIF5B	-0.068398059	25.03877554	-0.450227732	0.673047916	0.798756713	-7.35072679
UROD	-0.06908719	25.22796125	-0.466504294	0.662193	0.789578865	-7.342064914
EIF2B3	-0.069250043	24.32102637	-0.353413792	0.739456438	0.853278126	-7.396336178
UBA6	-0.07052303	23.65581405	-0.314928875	0.766651455	0.875553051	-7.411580755
TRMT1	-0.071533145	22.42192855	-0.268980516	0.799635311	0.90299784	-7.427572206
NUP107	-0.071945852	22.91844206	-0.424430449	0.690442236	0.812780541	-7.363876854

HNRNPAB	-0.072563706	25.93176531	-0.52920579	0.621287561	0.755003887	-7.306113216
CDC42	-0.072699036	25.43756278	-0.369284882	0.728365831	0.845758126	-7.389564982
EIF5A	-0.072911239	28.11145756	-0.662887874	0.539290553	0.682260967	-7.216405489
CD63	-0.072943212	23.14647128	-0.091809305	0.930727947	0.991495591	-7.466047627
NONO	-0.073631222	25.45870211	-0.410703115	0.699790364	0.819870548	-7.370582082
PSMA7	-0.07368706	25.97375924	-0.698994446	0.518438848	0.667294549	-7.189301384
THOC2	-0.074205877	7.246863228	-0.007747531	0.994144395	1	-7.471124782
FUBP3	-0.074949448	23.64732955	-0.266021808	0.801776876	0.904307634	-7.428518569
ALDH1B1	-0.078794365	23.54526242	-0.386189547	0.716637312	0.834322243	-7.38204515
OCIAD1	-0.082156361	23.27949861	-0.449030987	0.673849737	0.798756713	-7.351352569
UBAP2L	-0.082465104	24.80008834	-0.650320201	0.546680861	0.689252744	-7.225562233
PSMD1	-0.083154535	25.45781655	-0.963716229	0.383265889	0.563223183	-6.957817866
USP39	-0.084045076	23.38533575	-0.270603499	0.798461434	0.902131325	-7.427048777
DNAJC8	-0.085468561	23.29201681	-0.211931132	0.841264682	0.929664507	-7.444020836
PSMD12	-0.086051345	25.07395837	-0.814800285	0.455445408	0.622694615	-7.094774844
USO1	-0.086087225	24.12191887	-0.815816865	0.454918975	0.622358326	-7.093896243
ACLY	-0.086958018	25.30275311	-0.672834692	0.533489692	0.678792217	-7.20905596
GLUD1	-0.08757672	24.69188212	-0.176268299	0.867608405	0.947502671	-7.45235815
PSMD14	-0.088231564	24.81123215	-0.397932939	0.708542498	0.827503054	-7.376635782
CHMP4A	-0.088462651	22.79997257	-0.502005813	0.638850869	0.77002177	-7.322207494
MCM7	-0.089621057	25.44516768	-0.610862805	0.570320892	0.712579988	-7.253356379
ILF2	-0.090687475	25.35161522	-0.546921877	0.61000238	0.747429752	-7.295227098
GEMIN5	-0.09117464	24.26864051	-0.183645589	0.862141116	0.946521752	-7.450757197
OSBP	-0.091318021	23.21564666	-0.532345355	0.619278683	0.754080933	-7.304207072
KHSRP	-0.092063741	25.90693814	-0.873322015	0.425896071	0.599279253	-7.042893085
H1-5	-0.092436895	24.25468877	-0.174782786	0.868710367	0.947502671	-7.452672693
LRPPRC	-0.092608486	26.12456521	-0.426947194	0.688735225	0.811201414	-7.362625415
NAA10	-0.092852814	24.21852104	-0.446157342	0.675777136	0.800186481	-7.352848972
OXNAD1	-0.093145886	7.577296996	-0.009300863	0.99297042	1	-7.471108701
PDLIM1	-0.096177635	25.01265606	-0.823108114	0.451156824	0.618966868	-7.087570744
RPL27	-0.096472535	27.01523708	-1.012349028	0.361825106	0.547821966	-6.909751073
AKR7A2	-0.098348846	23.5067875	-0.731557828	0.500123069	0.651275288	-7.163866787
RAC2	-0.099392606	25.19645376	-0.405130132	0.703603308	0.823468218	-7.373245838
EIF2A	-0.099428562	24.07650236	-0.37017246	0.727747837	0.845482958	-7.389178027
SNX9	-0.10258969	24.01186259	-0.412601026	0.698494176	0.819569501	-7.369667207
PSME3	-0.102974224	24.88632487	-0.497820899	0.641578116	0.77190207	-7.324616384
RPL36A	-0.104249798	26.29331211	-0.286622203	0.786909039	0.891803451	-7.421719299
CYC1	-0.104831481	24.31967146	-0.733894208	0.498826947	0.650159054	-7.162006547
PSMA3	-0.107924003	25.18263934	-0.825830141	0.449758432	0.617965301	-7.085198558
PSMC2	-0.108975822	25.47447102	-0.559243196	0.602226868	0.740355357	-7.287470737
CAPRIN1	-0.110773441	25.97476219	-0.376094944	0.723630354	0.841139735	-7.386573645
SNRPE	-0.112733388	26.42865941	-0.670481901	0.534857946	0.679500156	-7.210802513
KRT8	-0.113228803	25.66660724	-1.000214674	0.367078062	0.551113816	-6.921886859
EIF2S1	-0.113659223	25.62726681	-0.590015073	0.583075184	0.7224142	-7.267445633

ECI2	-0.11415602	23.11261789	-0.324465219	0.75987423	0.871400991	-7.407959266
SMC4	-0.114596973	23.37482261	-0.629017288	0.559362169	0.701256866	-7.240749661
SF3A2	-0.118709175	23.72838651	-0.620220536	0.564654903	0.706694433	-7.246897183
PPP2R1B	-0.121097385	24.54091691	-0.442610582	0.678159984	0.802223594	-7.354683744
PSMD3	-0.121302984	25.2713035	-0.91020488	0.408062165	0.582931455	-7.008878372
SNRPGP15	-0.121713296	24.56390926	-0.578272909	0.590337776	0.729376127	-7.27519632
CCT3	-0.121751194	27.4305217	-0.947893651	0.390465593	0.569279338	-6.973120489
EIF3M	-0.122226057	25.00142523	-0.601980282	0.575733015	0.716920068	-7.259410374
RPS18	-0.124179858	27.22809603	-1.007301225	0.364002524	0.548723914	-6.914810781
MCM5	-0.124278069	24.77384642	-0.838033874	0.44352991	0.613969351	-7.07449232
GNAI3	-0.125757509	23.96434203	-0.396798998	0.70932221	0.827977898	-7.377164712
ALDOA	-0.126022902	29.30969606	-0.845062368	0.439973091	0.611680331	-7.068274031
MRRF	-0.126997869	22.25464429	-0.326941458	0.758118485	0.870287077	-7.407002021
FAM136A	-0.128672983	22.46061834	-0.178389729	0.866035323	0.947502671	-7.451904405
THOP1	-0.129019605	23.12208512	-0.378617251	0.721880052	0.839544987	-7.385452675
EZR	-0.130815557	27.01971168	-1.098398133	0.326387539	0.531148164	-6.821124868
RDX	-0.130815557	27.01971168	-1.098398133	0.326387539	0.531148164	-6.821124868
BID	-0.130958018	24.20441161	-0.360612263	0.734416866	0.850133964	-7.393299853
DHRS1	-0.131007193	7.342322562	-0.013499745	0.989797109	1	-7.471050569
TIMM9	-0.131761337	23.36344279	-0.417294489	0.69529388	0.817191271	-7.367387959
VASP	-0.133253452	23.90151188	-0.412160799	0.698794725	0.819569501	-7.369879764
ATAD3A	-0.133871253	24.83355463	-0.454483791	0.670200439	0.796985409	-7.348488954
CCT8	-0.136847395	27.22115542	-0.930604292	0.398459449	0.575639009	-6.989646765
RAB6A	-0.137705176	26.75070901	-0.212346293	0.840959343	0.929664507	-7.44391492
LAMTOR1	-0.139525826	23.59888601	-0.740592676	0.495124348	0.648189338	-7.156647295
ARCN1	-0.140801368	23.43880656	-0.242263781	0.819044998	0.91513638	-7.435748902
CORO1B	-0.142279145	23.98900094	-0.175026956	0.868529217	0.947502671	-7.452621173
SRPRA	-0.143528232	22.97994197	-0.520546273	0.62684827	0.760096345	-7.311318963
FAM162A	-0.143830082	23.66988825	-0.365894173	0.730728886	0.848058262	-7.39103516
CSDE1	-0.145324848	24.25513157	-0.509169497	0.634197759	0.766912713	-7.318042128
PPIL2	-0.146093747	23.14545007	-1.299375116	0.255366649	0.493605047	-6.598874308
HSPA5	-0.147807881	28.92635769	-1.127236734	0.31520815	0.531148164	-6.790472322
HPRT1	-0.148494918	25.07829546	-0.386227288	0.716611227	0.834322243	-7.382028008
DDX23	-0.149080965	23.18697012	-0.547191533	0.609831564	0.747429752	-7.295058967
PSMF1	-0.151384873	23.56388789	-0.816790114	0.454415415	0.622052934	-7.093054325
RPL8	-0.152521889	26.79289826	-1.634957638	0.168312617	0.370530058	-6.193686009
MIOS	-0.152793369	7.363097701	-0.015700079	0.988134287	1	-7.471011556
RPL6	-0.15726049	26.94844856	-1.121031135	0.317584765	0.531148164	-6.797106512
SET	-0.157747857	28.3472292	-0.737682865	0.496730308	0.648760773	-7.158980059
HSPA8	-0.157760787	29.72907229	-1.636844294	0.167917663	0.370386972	-6.191324483
PPP6C	-0.157991805	23.49862493	-1.478051824	0.204633006	0.425165331	-6.387258315
LMNB2	-0.159413383	23.22976503	-0.54529697	0.611032302	0.74786579	-7.296238692
TPP2	-0.160776876	23.59527598	-1.466447502	0.207608363	0.428144013	-6.401320686
MAD2L1	-0.162376238	23.00129378	-0.342430253	0.747175157	0.859717077	-7.400857239

VCP	-0.162649147	26.70689585	-1.428596062	0.217609836	0.443820061	-6.446908224
IPO5	-0.164067173	25.00174629	-1.605018407	0.174707012	0.379329609	-6.231064007
RPS2	-0.164578031	27.34445086	-1.519763098	0.194279962	0.409797753	-6.336397963
PFDN5	-0.165156402	24.33990014	-0.596422405	0.579136112	0.719945677	-7.26315994
BLMH	-0.167150829	23.97778649	-0.252673822	0.811463243	0.910792583	-7.432661681
MCM3	-0.167518552	24.98508906	-1.177679152	0.296470978	0.531148164	-6.735797237
PGRMC2	-0.167565764	23.9118973	-0.507162064	0.635499708	0.768068547	-7.31921468
PFAS	-0.16787394	24.61193295	-0.602402304	0.575475134	0.716920068	-7.259124448
NUDCD1	-0.169777717	22.6943097	-0.513011445	0.63171029	0.764945402	-7.315786504
RPS6	-0.170257463	27.58405639	-1.70613442	0.154045289	0.351311918	-6.10413597
DARS1	-0.172816082	26.09964981	-0.716527801	0.508518841	0.657961112	-7.175721265
SURF4	-0.172927309	24.37970155	-1.151866328	0.305930802	0.531148164	-6.763940112
TUBB	-0.173980902	30.33673192	-1.261692824	0.26749382	0.50689051	-6.641976426
ZNRD2	-0.175367666	24.10631923	-0.665191148	0.537943507	0.680945033	-7.214711651
RECQL	-0.175991536	22.19567617	-0.506396423	0.635996678	0.76825075	-7.319660805
NXF1	-0.1762523	7.275756937	-0.018327603	0.986148724	1	-7.470957268
DHX15	-0.176955695	24.68625157	-0.594330687	0.580420187	0.720573535	-7.264563394
COPB1	-0.176996922	24.31168065	-0.441957987	0.678598899	0.80224345	-7.355019874
NDUFB10	-0.177028568	23.74203255	-1.159872886	0.302967949	0.531148164	-6.755247019
ALB	-0.1770734	24.93827435	-1.008025501	0.363689421	0.548723914	-6.914085783
MRPS9	-0.181875668	14.87345903	-0.018499634	0.986018727	1	-7.470953421
STT3A	-0.182084434	24.58842897	-0.822240713	0.451603136	0.618966868	-7.088325446
VKORC1L1	-0.183572018	7.156025143	-0.019407934	0.985332374	1	-7.470932515
EIF3E	-0.18445103	24.71469227	-0.812870548	0.456445995	0.623678364	-7.096440414
PRDX2	-0.184809953	28.1846339	-0.602785563	0.575241003	0.716920068	-7.258864637
KARS1	-0.185300591	26.07039077	-0.84175546	0.44164381	0.612887814	-7.071204467
DDX42	-0.186807008	23.54934345	-0.569594221	0.595741747	0.734014044	-7.280838376
SNRPD3	-0.187016743	25.49018932	-0.643737952	0.550578527	0.693378974	-7.230299957
PHB2	-0.187110177	26.21921003	-1.953226309	0.113478784	0.283237697	-5.787932408
AARSD1	-0.187517584	14.86108846	-0.019085725	0.985575848	1	-7.470940046
PCBP2	-0.188363424	27.32632224	-1.791921606	0.138479887	0.326619258	-5.995126552
PRMT5	-0.18844239	24.95104069	-1.480585601	0.20398892	0.425024801	-6.384182632
HUWE1	-0.188755551	23.0580893	-0.749035321	0.490486046	0.644016886	-7.149838185
XRCC6	-0.18981854	26.08556611	-1.584466795	0.17923724	0.387271115	-6.256611486
GARS1	-0.190797204	25.46182245	-1.038404494	0.350761059	0.540137953	-6.883382145
RPL23	-0.191342962	27.4073468	-0.467979509	0.661213856	0.789259573	-7.341266012
LYPLA1	-0.191589513	24.79521742	-0.826078744	0.449630883	0.617965301	-7.084981617
LAMTOR5	-0.192967621	23.43945607	-0.291004399	0.783759578	0.889142384	-7.420209812
SF3B3	-0.193407204	24.71479219	-1.081674928	0.333028842	0.531148164	-6.83868846
RUVBL2	-0.196698791	25.0250968	-1.253572372	0.270176431	0.508648889	-6.651185474
MAGOH	-0.196865541	25.00777401	-0.344848661	0.745472615	0.858880442	-7.399873396
RPS23	-0.197548187	25.90549267	-1.43149487	0.216827607	0.443039097	-6.443432697
ALDH18A1	-0.197567238	23.93300913	-0.545717337	0.610765758	0.74786579	-7.295977243

ERO1A	-0.197628242	22.8449831	-1.151661741	0.306006849	0.531148164	-6.76416181
PPIL4	-0.198757202	22.91735943	-0.609216623	0.571321427	0.713027135	-7.254484046
ACP1	-0.199559354	24.7756864	-0.86178484	0.431599787	0.605003112	-7.053327686
LARP4	-0.200079952	15.08276614	-0.020071409	0.98483103	1	-7.470916611
RCC2	-0.200335938	25.64212184	-1.203173711	0.287388046	0.527036426	-6.707676507
STX7	-0.200876023	22.83173428	-0.629884878	0.558841937	0.701106668	-7.240139409
ATP1A1	-0.204071447	24.63705921	-2.117906366	0.092817797	0.261829082	-5.574863115
AP1B1	-0.205068482	24.38795989	-1.728264125	0.149865487	0.346319292	-6.076117933
LYPLA2	-0.205420108	23.87611751	-0.635654467	0.555390444	0.698250083	-7.236063201
API5	-0.20721882	24.41216332	-1.694683457	0.156254734	0.354619661	-6.118602993
SPN	-0.208251872	24.3951926	-0.921567633	0.402690452	0.578089539	-6.998202195
PDIA4	-0.209194297	25.29944414	-1.545503299	0.188150945	0.402504379	-6.30478068
GSR	-0.211561226	25.20899773	-1.274676499	0.263255959	0.501429161	-6.627192926
PRKAR2B	-0.211707667	22.61908991	-0.266985815	0.801078887	0.904167879	-7.428211339
XPOT	-0.211766372	22.85595568	-1.031245408	0.353771895	0.54139299	-6.890668987
RPL35A	-0.211993526	25.78435185	-0.722458288	0.505194143	0.655187495	-7.171067118
RACK1	-0.212033807	27.62489108	-1.440057359	0.214532958	0.439971011	-6.433151176
FLNC	-0.214129394	25.44926597	-1.251657792	0.270812536	0.508862281	-6.653352504
S100A11	-0.215253222	25.71085258	-0.675340713	0.532034966	0.677718479	-7.207190147
TMEM43	-0.217127545	22.93241598	-0.42217534	0.691973606	0.814151342	-7.364992387
NAE1	-0.217179638	22.70826874	-0.921293748	0.402819253	0.578089539	-6.998460602
EXOSC6	-0.217905357	22.763068	-1.077951587	0.334523468	0.531148164	-6.84257728
SDF2L1	-0.21816756	24.76298644	-1.47430468	0.205589184	0.425165331	-6.391803476
RPS15A	-0.218366389	26.58637818	-1.943865724	0.114791406	0.285243146	-5.800011899
DHX30	-0.218940073	22.4288203	-0.499880622	0.640235001	0.771271155	-7.32343304
NPEPPS	-0.219266635	23.58715664	-1.688810041	0.157400521	0.35553268	-6.126014913
CERT1	-0.219760401	7.193999987	-0.023110477	0.982534719	1	-7.47083693
MARCKSL 1	-0.219801119	25.22547807	-1.643374969	0.166557744	0.369222412	-6.183144638
MCM2	-0.219874418	25.53759863	-1.924238508	0.117596008	0.290537986	-5.825322617
CCT2	-0.223084401	26.90774004	-1.807724866	0.135794341	0.322275554	-5.974938909
AP2M1	-0.223732548	24.13534092	-1.390574601	0.228125703	0.458109444	-6.492239724
PLD3	-0.223779126	22.98561359	-1.149222776	0.306914744	0.531148164	-6.76680309
STAU1	-0.223810192	23.0510807	-1.254434461	0.269890459	0.508648889	-6.650209185
AP2B1	-0.227245527	24.70477736	-1.100546232	0.325542934	0.531148164	-6.818857427
RPL21	-0.227695879	26.80013487	-1.545383522	0.188179016	0.402504379	-6.304928193
PFKL	-0.227737043	24.04688258	-1.978298238	0.110040582	0.278744352	-5.755554334
NSFL1C	-0.228586235	24.03108987	-1.554530906	0.186047151	0.400037431	-6.293652438
HNRNPA0	-0.228591284	25.49145574	-1.142613847	0.309387013	0.531148164	-6.773944703
ACADM	-0.228973724	23.47323154	-1.172349097	0.298402459	0.531148164	-6.741635962
ETF1	-0.2298082	25.35354695	-2.027430948	0.10361931	0.27114534	-5.692022061
RPSA	-0.230134892	28.13499232	-0.5823321	0.587820759	0.727481464	-7.272532133
CCT6A	-0.231995339	27.0993517	-2.092293502	0.095747523	0.264587489	-5.608041781
HNRNPA2	-0.232560142	27.55244423	-1.916457316	0.118727798	0.292676755	-5.835349985

B1						
TCERG1	-0.232996124	23.139852	-2.442435961	0.063044878	0.235120311	-5.155807891
TRMT10C	-0.23578284	23.64963507	-2.174359364	0.086695667	0.261829082	-5.501735224
YBX1	-0.235887853	27.66244136	-2.260305237	0.078198601	0.257646807	-5.390499798
TUBB2A	-0.238782331	30.08832917	-2.462941481	0.061552094	0.232681594	-5.129515239
TUBB4B	-0.240950301	30.28135875	-1.438513358	0.214944983	0.440408972	-6.435006859
EIF4G1	-0.240995916	25.67970746	-2.081067907	0.097062713	0.265586545	-5.622581493
PLRG1	-0.24114158	23.32175035	-0.519118476	0.627767914	0.76079574	-7.312169977
BZW1	-0.241366813	23.70608154	-1.483028091	0.20336992	0.424133321	-6.381216017
EIF2B4	-0.241983776	22.612034	-0.776542546	0.475594238	0.63683646	-7.127238168
PSMC1	-0.242017599	24.63688994	-1.913241492	0.119198889	0.292915099	-5.839492877
HMGB1	-0.244432422	27.65541736	-2.47958154	0.060369424	0.230171395	-5.108202687
LSM2	-0.244940201	24.83819989	-0.629743973	0.558926406	0.701106668	-7.240238568
RPS12	-0.245813175	27.57009097	-2.063048777	0.099214477	0.265979027	-5.645916591
MAVS	-0.246831918	22.98481375	-0.574594357	0.592624568	0.731387051	-7.277596777
WDR61	-0.24686559	15.39098759	-0.024266829	0.981661034	1	-7.470803667
TNPO1	-0.248072087	25.24482261	-0.79294023	0.466877853	0.632093932	-7.113468721
RAB11B	-0.248908906	24.58707962	-1.814527238	0.134655055	0.320256771	-5.966240371
TXNDC5	-0.249493237	24.14324636	-1.455743863	0.210390254	0.432674675	-6.41425634
ARFGAP1	-0.251162775	15.00901757	-0.025298258	0.980881759	1	-7.470772628
PPP2R2A	-0.251452613	23.87579814	-2.607614165	0.052069218	0.215562676	-4.945013727
ZC3HAV1	-0.251603023	23.86718999	-1.269061589	0.265080941	0.504039939	-6.633595061
TFG	-0.252099284	23.69788234	-1.154324954	0.305018227	0.531148164	-6.761274172
RPLP0	-0.252264292	27.6695123	-0.857039398	0.433963168	0.60756742	-7.057590643
NRDC	-0.252840089	23.59704722	-1.00734839	0.363982128	0.548723914	-6.914763579
RPS14	-0.253119592	27.72960178	-1.841024148	0.130311134	0.313161053	-5.932309226
NUP205	-0.253882986	23.16086493	-1.083022363	0.33248939	0.531148164	-6.837279185
YARS1	-0.255823976	26.07102417	-2.74533509	0.044534365	0.193630427	-4.771297807
PDCD6IP	-0.257852161	25.59897737	-0.981038651	0.375510228	0.556990104	-6.940873172
NCBP1	-0.259341291	24.03381627	-1.046234178	0.347493372	0.536595541	-6.875377161
NSDHL	-0.262833109	23.82721082	-0.562194936	0.600373148	0.738895184	-7.285590211
RPS11	-0.26619923	26.40019609	-1.837501874	0.130880071	0.313970677	-5.936823994
TXNRD2	-0.26767204	23.89233023	-0.383647544	0.718395288	0.835930333	-7.383196104
USP7	-0.267949731	23.81000598	-0.887075348	0.419174741	0.594784295	-7.030325114
PSMB5	-0.268253627	25.17603877	-0.82843617	0.448422751	0.617364205	-7.082922035
HNRNPU	-0.268258317	27.81878761	-1.474529012	0.205531818	0.425165331	-6.391531483
UPF1	-0.268674349	24.21265564	-1.757305017	0.144556209	0.33800867	-6.039238048
RAB14	-0.269799738	26.32482175	-2.28058401	0.076328644	0.256131486	-5.364286139
PARP1	-0.271235931	25.49464967	-2.44780054	0.062650514	0.234437591	-5.148926262
NMT1	-0.271352441	24.30605272	-1.654347233	0.16429797	0.365700903	-6.169383031
PAIP2	-0.27271471	23.54701696	-0.360589193	0.734432992	0.850133964	-7.393309676
ELAVL1	-0.27427577	23.78142955	-1.293063195	0.257361513	0.495303726	-6.606135382
PPP4C	-0.275334741	24.02701734	-0.893839398	0.4159002	0.591970842	-7.024093183
PHF5A	-0.277051955	23.40010832	-2.0821936	0.096929958	0.265586545	-5.621123533

BPNT1	-0.279955832	23.16290679	-1.548694454	0.187404589	0.401979045	-6.300849262
RPN2	-0.280214553	25.57689414	-1.265679422	0.2661859	0.504843172	-6.637444904
UBE2V1	-0.280537676	25.02642304	-1.958745671	0.112712255	0.282608467	-5.780807525
GSPT1	-0.282703189	25.44283112	-3.258617669	0.025541817	0.135916765	-4.144985253
TFRC	-0.284327938	27.61003853	-2.816917067	0.041107454	0.185066934	-4.681857895
RPL12	-0.284411118	27.74679702	-2.150246867	0.089255563	0.261829082	-5.53296769
HCFC1	-0.28491601	23.24578659	-0.797645698	0.464398859	0.631047806	-7.109477135
AKAP8L	-0.285601879	14.90668693	-0.028989539	0.978093098	1	-7.470650964
COLGALT 1	-0.286456984	23.15452467	-0.580013683	0.589257536	0.728853106	-7.274055759
PDIA3	-0.286721092	26.92765675	-0.882857152	0.421227183	0.595596578	-7.034194556
ALDH16A1	-0.286724351	7.480507764	-0.028995826	0.978088349	1	-7.470650743
PSMB4	-0.286982967	25.37769282	-1.133236611	0.312925349	0.531148164	-6.78403845
SOD1	-0.287685105	27.79060732	-2.710672024	0.046308511	0.198741724	-4.814824692
DHX9	-0.287784373	25.54733499	-1.626655947	0.170061675	0.373260987	-6.204068788
HDAC1	-0.287957385	24.39128309	-1.929441164	0.116845614	0.289375466	-5.818615832
XPO1	-0.288308268	25.76376164	-0.70568764	0.514636029	0.662784299	-7.184149166
MTX2	-0.289005024	14.89234582	-0.029351512	0.977819656	1	-7.470638143
GLOD4	-0.290190716	23.46486541	-1.129022345	0.314527229	0.531148164	-6.788559558
RPL27A	-0.292249409	26.98615598	-0.694964557	0.520737952	0.66831551	-7.192384369
CAVIN1	-0.292780787	22.78809486	-1.385589016	0.229540375	0.459836852	-6.498147719
UBLCP1	-0.293214314	22.21355571	-0.602466687	0.575435798	0.716920068	-7.259080813
DPP3	-0.293583275	23.82058097	-1.065656046	0.339500706	0.53195473	-6.855362468
SEC31A	-0.294300795	23.92880423	-0.756762328	0.486268714	0.641135042	-7.143553471
PRKDC	-0.295024961	25.74891464	-3.282949134	0.02490377	0.133805	-4.116224259
RPL24	-0.295242087	26.57745796	-0.935563343	0.39615303	0.573426989	-6.984927644
TOMM40	-0.296076588	25.79348592	-1.145313209	0.308375093	0.531148164	-6.771030521
EIF5	-0.296200804	25.41754395	-0.869494183	0.427781842	0.601551272	-7.046366118
TTC27	-0.296349623	22.80566641	-1.336644095	0.243881238	0.480125922	-6.555676562
SPCS3	-0.296499417	23.82938398	-0.864083561	0.430458586	0.603784831	-7.051256591
XPO5	-0.296944543	24.08563571	-2.142649801	0.090078917	0.261829082	-5.542809018
RPL36	-0.297538802	26.48431793	-1.223511932	0.280324713	0.518799448	-6.685020225
TIMM44	-0.297619465	24.07944506	-1.14561362	0.30826266	0.531148164	-6.770705968
PRPF8	-0.298656151	24.32449327	-1.30298981	0.254230775	0.492305779	-6.594708747
NMD3	-0.299269906	22.87624466	-0.4031679	0.704948254	0.824172906	-7.37417568
RPL7A	-0.299302769	27.31069289	-1.588400217	0.178361168	0.385753833	-6.251729127
NUFIP2	-0.300754144	22.63017343	-0.989929535	0.371580704	0.554090193	-6.932099764
ATP5MK	-0.300981494	15.57808872	-0.029218904	0.977919831	1	-7.470642858
NUP210	-0.301493991	23.67587854	-2.002883495	0.10677618	0.273282981	-5.723775577
ANP32B	-0.304483841	27.61016987	-0.671074878	0.534512877	0.679500156	-7.2103628
BYSL	-0.305511659	23.13801285	-1.847479759	0.129275073	0.312145144	-5.924031345
SMS	-0.311350966	23.64909509	-0.773112673	0.477432652	0.637055355	-7.130090459
NDUFA10	-0.312242708	7.409126388	-0.031879416	0.975910127	1	-7.470544171
PMPCB	-0.312301577	23.23357774	-0.764955341	0.481826118	0.637802859	-7.136835067

TMSB4X	-0.314735053	7.318880047	-0.032529786	0.97541888	1	-7.47051874
RPS3	-0.31511811	27.82025869	-2.765525681	0.043536246	0.191217674	-4.74600825
GAPVD1	-0.317488121	23.67400486	-1.993092572	0.108063717	0.274992418	-5.736434388
RAD23B	-0.317543253	25.28857602	-1.722224612	0.150994557	0.34740346	-6.083772033
RAB8A	-0.318857521	26.71218066	-0.791615269	0.467577677	0.632655406	-7.11458944
CEP170	-0.319249394	7.098993452	-0.034017602	0.97429513	1	-7.470458633
BUB3	-0.319891521	25.09072196	-2.883320676	0.03819251	0.177195928	-4.599452164
NDUFS3	-0.320381906	23.25165848	-0.764027016	0.48232799	0.637802859	-7.137599122
CAT	-0.321192896	23.70808474	-1.032820325	0.353107655	0.541161744	-6.889068647
MSH6	-0.322184602	23.64975429	-1.804025498	0.136418119	0.323064895	-5.979667252
RPL13	-0.324617163	27.25455104	-2.976414966	0.034491674	0.168245682	-4.484887006
EXOSC1	-0.324749807	22.7983295	-1.392633303	0.227543988	0.457826152	-6.489797631
RBM14	-0.325935294	23.1868919	-1.524436803	0.193152595	0.408195817	-6.330669799
PPA1	-0.325955209	27.22821108	-3.456462629	0.020850264	0.121117111	-3.913726478
DDX46	-0.326863078	23.83281015	-0.442520476	0.678220578	0.802223594	-7.354730182
EIF3K	-0.327248362	24.51884699	-1.231607433	0.277557721	0.516465703	-6.675948511
KHDRBS1	-0.328274172	25.60425706	-1.710948967	0.153125886	0.34994859	-6.09804693
EIF3D	-0.329148277	25.54175099	-1.326852505	0.246850629	0.484316131	-6.567078485
SRPRB	-0.329305804	23.70585718	-2.086548293	0.096418228	0.265120257	-5.615483328
TALDO1	-0.329841861	27.09975174	-2.932737553	0.036174811	0.171155448	-4.538494706
PRPF40A	-0.330914949	23.49550766	-2.653530042	0.049409028	0.207648926	-4.886871013
TBL2	-0.33103797	22.74194447	-0.855876465	0.434543888	0.607622311	-7.058632743
COX20	-0.331086755	7.175031704	-0.034904619	0.973625196	1	-7.470421519
LSM8	-0.332637652	7.710191699	-0.032635313	0.975339173	1	-7.470514565
COPA	-0.333350949	24.62341746	-1.670970706	0.160933374	0.360354347	-6.148490616
ATXN7L3B	-0.334105181	22.52150507	-1.694477641	0.15629474	0.354619661	-6.11886282
SAE1	-0.334433588	24.81416413	-1.168371091	0.29985138	0.531148164	-6.745984345
PSMB7	-0.337173141	24.55177746	-1.705023739	0.154258188	0.351436263	-6.105540135
HNRNPUL1	-0.339490859	24.22336865	-1.505819749	0.197682074	0.415394433	-6.353452763
SDHB	-0.339912542	23.90089996	-1.207702454	0.285801317	0.52545572	-6.702648459
CAPZB	-0.341484272	25.35070332	-3.385013888	0.022419633	0.128219502	-3.996553427
EMD	-0.343651214	24.11768293	-2.126614112	0.091843835	0.261829082	-5.563582545
NME2	-0.346170388	28.55685215	-2.626980257	0.05092818	0.212025573	-4.920464364
DLD	-0.346418115	25.6586039	-2.549322198	0.055679487	0.221818279	-5.019128306
RPA1	-0.347605225	22.96270739	-1.302926101	0.254250754	0.492305779	-6.59478221
CAPZA2	-0.347728638	24.45392021	-0.79381664	0.466415379	0.632093932	-7.112726629
RPS3A	-0.347839263	27.63513348	-2.316467769	0.073138208	0.252008826	-5.317942001
SSB	-0.347995172	26.81268078	-3.090620903	0.030495976	0.153783279	-4.345954936
HNRNPDL	-0.348433523	25.898443	-2.013182339	0.105439431	0.27197314	-5.710456023
PAFAH1B3	-0.351845695	24.23715061	-2.92043374	0.036665639	0.172375113	-4.553641894
CRKL	-0.353857024	25.8287361	-2.580071024	0.053741503	0.218410981	-4.979992699
PRPF3	-0.354616333	7.337203582	-0.036558011	0.972376514	1	-7.470349791
PSMA1	-0.357335827	26.09334889	-3.569740475	0.018614982	0.114832887	-3.784024867

RPL26L1	-0.358405196	25.62709591	-1.806266666	0.136039865	0.322513312	-5.976802894
FEN1	-0.361533955	25.94868388	-2.078879909	0.097321307	0.265955642	-5.625415262
SRSF5	-0.362199259	24.54373159	-1.225428676	0.279667303	0.518544617	-6.682875067
TCP1	-0.36492309	26.45620077	-4.241869531	0.009887426	0.079881106	-3.055443571
SHMT2	-0.365741479	25.29455615	-3.18110481	0.027703118	0.142796854	-4.237197557
SEC13	-0.367743862	24.65403348	-2.063405592	0.099171376	0.265979027	-5.645454563
PSMD8	-0.370683904	24.98457926	-2.252054309	0.07897368	0.257709698	-5.401169745
EPRS1	-0.372225462	26.45613537	-3.146230991	0.028743366	0.146624204	-4.278973608
RAB5C	-0.374051492	24.35262453	-3.225417597	0.026442949	0.138716084	-4.1843722
NDUFS8	-0.374780846	22.97634971	-2.744225243	0.044589972	0.193630427	-4.772689318
PRDX4	-0.375422031	26.33825492	-2.028498801	0.103484257	0.27114534	-5.690640276
VARS1	-0.379178743	25.59908842	-3.644131348	0.017297979	0.110937039	-3.699933103
SSBP1	-0.380173683	25.48286005	-1.224594214	0.279953335	0.518544617	-6.683809179
ERP29	-0.380235997	25.11683296	-1.984891166	0.109154917	0.277402806	-5.747034968
TPR	-0.382631768	23.88411277	-2.353365418	0.070009148	0.248560479	-5.270353125
SF3B6	-0.383665922	23.22880664	-0.709930577	0.512235544	0.661227849	-7.180862661
DDX39A	-0.387156555	27.0128474	-1.244253866	0.273285494	0.512179485	-6.661717423
RPL15	-0.387226111	26.60589324	-1.089672257	0.329838242	0.531148164	-6.83030896
PABPC1	-0.389027819	27.15256017	-3.901400646	0.013509264	0.095192709	-3.41576713
IPO8	-0.389497367	7.272807783	-0.040506776	0.969394691	1	-7.470165065
EWSR1	-0.391546469	24.67620187	-3.334843162	0.023603199	0.130556347	-4.055180947
TRNT1	-0.391915355	7.284782664	-0.040691113	0.969255507	1	-7.470155979
AGFG1	-0.393930534	23.0396465	-1.880303303	0.124138697	0.302043605	-5.881881582
SLC16A1	-0.394408626	23.64293205	-3.56879065	0.018632534	0.114832887	-3.785104124
NUP93	-0.394626878	24.53721961	-2.057722915	0.0998602	0.266333876	-5.65281256
PHB1	-0.395193902	26.14655874	-1.348022627	0.240473119	0.47474186	-6.542380897
PSMA6	-0.39593261	25.71081537	-2.474671119	0.060715789	0.230304846	-5.114489681
BRIX1	-0.396895171	22.90977957	-0.914619118	0.405968507	0.580440797	-7.004741706
RBM12	-0.397235135	23.01497538	-0.669075523	0.535676963	0.679500156	-7.211844116
DYNC2H1	-0.400438454	7.631981147	-0.039685457	0.970014846	1	-7.470205045
NANS	-0.403968528	24.93104426	-2.632435585	0.050611817	0.211901174	-4.913555804
SRRM1	-0.404338609	23.70756463	-0.77013091	0.479035154	0.637802859	-7.132562216
ST13	-0.404738978	26.26556804	-1.214163571	0.283551386	0.522591798	-6.695458184
EML4	-0.404972962	23.44104851	-0.558387836	0.602764695	0.740606233	-7.288014066
AARS1	-0.411294461	25.54864825	-2.444559297	0.06288846	0.234931804	-5.153083843
SNU13	-0.41509967	23.87165839	-1.194869345	0.2903185	0.529348194	-6.716870971
KRT19	-0.418105275	24.29381571	-1.903108662	0.120696163	0.295938989	-5.852541918
BRI3BP	-0.422156098	7.204277759	-0.044317669	0.966517528	1	-7.46996885
CMPK1	-0.422216681	25.22045026	-1.19866268	0.288976547	0.528203425	-6.712675161
REEP6	-0.423741154	23.68231561	-0.996503128	0.368697585	0.552423998	-6.925580162
RTN4	-0.426332922	26.225742	-1.925857737	0.117361919	0.290330098	-5.823235449
PA2G4	-0.42711241	27.52434101	-4.342063758	0.00904797	0.07663147	-2.952758722
RPL14	-0.428601354	26.93339747	-2.075084356	0.097771647	0.265979027	-5.630330882
EEF1A1	-0.428957712	30.45004318	-4.371231517	0.008819549	0.075260205	-2.923147411

CAPZA1	-0.433042708	25.9686587	-4.693926338	0.006696755	0.064662285	-2.603842954
EIF6	-0.436731728	25.9693772	-4.626385162	0.007086087	0.06612694	-2.669432657
GRSF1	-0.436959103	23.71679874	-1.148443867	0.307205197	0.531148164	-6.767645961
EIF4EBP1	-0.437333446	24.99644745	-0.441023268	0.67922782	0.802559389	-7.355500524
ARL1	-0.439703095	23.06467995	-1.712884647	0.152757825	0.349813843	-6.095597797
PTPN1	-0.440947873	23.77446477	-3.988672448	0.012450639	0.092711303	-3.321709114
SNRNP70	-0.44157788	24.61646763	-1.27974116	0.261619866	0.499380171	-6.621406704
PSMD6	-0.442823218	24.24135239	-1.852095177	0.128539615	0.311046244	-5.918110588
RPL26	-0.443956344	25.94484641	-1.455907681	0.210347405	0.432674675	-6.41405862
TMX2	-0.444939849	23.83626677	-2.82803169	0.040602499	0.184736741	-4.668026232
PRPS2	-0.446420633	24.53309472	-1.294575629	0.256882186	0.494921009	-6.604397003
FARSA	-0.447241023	24.700548	-4.593349968	0.007286226	0.067095841	-2.70175007
PSMB6	-0.448442878	25.523982	-1.979033899	0.10994138	0.278744352	-5.754603815
HADHB	-0.450297827	24.95659975	-3.603809377	0.017997901	0.113781604	-3.745406231
RPL32	-0.450900707	26.27704984	-4.68375188	0.006753756	0.064662285	-2.613682058
SNRNP200	-0.451709226	23.75071785	-2.936816779	0.036013729	0.170757404	-4.533477217
ARID1A	-0.453182407	7.101608808	-0.048258688	0.963542746	1	-7.469747409
SNRPD2	-0.45406953	25.40402472	-0.668721263	0.535883404	0.679500156	-7.212106207
ATXN2L	-0.454108232	24.44725913	-2.206151635	0.083441116	0.261533105	-5.460567741
GANAB	-0.45953526	25.95175356	-4.149677281	0.010741734	0.083934245	-3.151262665
RPL35	-0.461980948	26.2543044	-1.154899836	0.304805199	0.531148164	-6.760650366
SYNE2	-0.463067085	15.39183877	-0.045514475	0.965614078	1	-7.469903595
ABCF1	-0.463508421	24.88131699	-4.619920849	0.007124734	0.066149727	-2.67574423
HTRA2	-0.464111969	7.468531491	-0.046995872	0.96449588	1	-7.469820416
TRMT1L	-0.468247974	23.04160141	-2.043425435	0.101615988	0.268435569	-5.671321969
RPL7	-0.469600649	27.47249673	-4.451678218	0.008223891	0.072415932	-2.842129235
SMAP	-0.469761075	25.03136988	-2.825644712	0.04071035	0.184736741	-4.670995431
PAICS	-0.470217344	26.82359495	-3.784731558	0.015093127	0.101798322	-3.543355181
RPS27	-0.470302029	26.46367418	-1.101352322	0.325226486	0.531148164	-6.818005888
DDX6	-0.476648741	25.13859097	-2.041587607	0.101844062	0.268644992	-5.673700849
FUS	-0.47788412	25.58779365	-2.103695956	0.094431167	0.263575798	-5.593271677
RPL28	-0.478698373	26.12577767	-2.554152254	0.055369982	0.221380164	-5.012975035
CSNK2A2	-0.481725412	23.39285507	-2.513210954	0.058055335	0.224433428	-5.065198496
GMPS	-0.482359568	25.66101033	-3.484360412	0.020272209	0.119075217	-3.881599422
CARM1	-0.48289509	23.17998002	-1.673872162	0.160353331	0.360161167	-6.144838902
DAP3	-0.482975554	22.96538454	-1.895230033	0.121873997	0.298492015	-5.862682655
PNO1	-0.484024055	22.74549437	-3.3647193	0.022889967	0.128915829	-4.020222654
RPL3	-0.488094506	27.28093232	-3.289057199	0.024746486	0.133282652	-4.10901819
PPME1	-0.489515804	22.75200184	-1.530678663	0.191657056	0.406973212	-6.323010824
MAPRE1	-0.490945055	25.66865541	-3.784869101	0.015091136	0.101798322	-3.543203517
RPL5	-0.492200843	26.83279789	-4.815232616	0.006059156	0.060838312	-2.487654135
NME1	-0.492756952	28.63014543	-4.45446124	0.008204148	0.072415932	-2.839343477
FAF1	-0.493680389	23.43127736	-2.272804109	0.077040219	0.257071048	-5.374341104
NAMPT	-0.493911298	25.42998278	-1.672036932	0.160719973	0.36024002	-6.14714886

AP2A1	-0.49608028	24.34275338	-2.098328135	0.095048418	0.263614246	-5.600224991
U2AF1	-0.496593427	25.04570995	-1.112220583	0.320986278	0.531148164	-6.806489768
SHC1	-0.499535059	7.220564951	-0.052313745	0.960482603	1	-7.469499901
PDLIM5	-0.500618747	23.29604234	-3.498714501	0.019982016	0.118874246	-3.865116153
C7orf50	-0.501420028	14.26294314	-0.053118408	0.959875458	1	-7.469448416
SMARCD2	-0.501734983	14.68806885	-0.051624642	0.96100258	1	-7.469543368
TUBAL3	-0.501986613	27.97296409	-2.558642336	0.055083979	0.221370512	-5.007256797
PPP1R8	-0.502278323	23.03555887	-3.549762542	0.018988235	0.115584687	-3.80675475
RPS13	-0.50345133	26.81614318	-2.531309188	0.056850822	0.22439954	-5.042094649
ACBD3	-0.505871357	22.20542028	-3.330427355	0.023710774	0.130556347	-4.060359427
PSMC4	-0.506896453	24.65922867	-2.130704073	0.091390175	0.261829082	-5.558284134
PCYT1A	-0.508369969	15.00639878	-0.051209098	0.961316148	1	-7.469569301
CORO1C	-0.509187131	23.78379775	-1.742660647	0.1472089	0.342800903	-6.057850604
PPP5C	-0.509244553	23.78114569	-1.13508756	0.312224083	0.531148164	-6.782049748
ACAT2	-0.5095988	25.96797351	-2.431664405	0.063845029	0.236512206	-5.169631879
SRRT	-0.510021401	23.91960696	-2.516410138	0.05784037	0.22439954	-5.061112419
GAGE8	-0.513975273	27.76969084	-1.62465634	0.1704857	0.373821906	-6.206567586
CCNB1	-0.514158154	23.5667709	-2.045118814	0.101406324	0.268435569	-5.669129989
LIMS1	-0.51490737	21.96549104	-2.524051332	0.057330497	0.22439954	-5.051356465
JPT2	-0.51566658	24.84755291	-3.596392673	0.018130193	0.114292322	-3.753798011
LUC7L3	-0.516908171	23.70852023	-3.037094554	0.032299045	0.159425885	-4.410843275
UBXN4	-0.517759476	23.52590457	-0.902286942	0.411839412	0.586567172	-7.016263622
CYCS	-0.521600172	25.63418788	-2.305836965	0.074067928	0.253636932	-5.331665764
DDX39B	-0.523677743	27.07533437	-1.853296598	0.128348888	0.31092378	-5.916569041
SNRPD1	-0.524835058	25.8394489	-1.656638508	0.163830008	0.365365615	-6.16650639
RAB10	-0.527387753	26.74363831	-1.840463949	0.130401448	0.313161053	-5.93302736
ASNSD1	-0.5334631	7.961795904	-0.050667933	0.961724521	1	-7.46960276
DEK	-0.535616383	23.77236076	-1.887808971	0.122994463	0.300577878	-5.872229983
UQCRH	-0.537054489	24.06840046	-1.171753758	0.298618899	0.531148164	-6.742287237
PPAT	-0.537534512	23.14953946	-2.021932745	0.104317655	0.271706248	-5.699136089
KPNA3	-0.538898846	22.55084524	-1.149339745	0.306871148	0.531148164	-6.766676489
GLO1	-0.543193997	25.8428091	-4.751445179	0.006385011	0.063251515	-2.548494135
RCC1	-0.543430454	23.83056911	-2.899015242	0.037538196	0.175585062	-4.580057741
STMN1	-0.549667079	27.24366976	-5.780437725	0.002903637	0.042376182	-1.63213558
COTL1	-0.557802193	24.86320276	-2.15333807	0.088922872	0.261829082	-5.528963414
SRRM2	-0.55819777	24.70033268	-1.813425175	0.134838957	0.320350798	-5.967649991
ATP2A2	-0.559099689	24.93740402	-4.292191952	0.009454866	0.077616364	-3.003682384
LARP7	-0.560725748	22.12558587	-5.010277749	0.005178268	0.055243159	-2.305087327
RAP1B	-0.562368718	25.23725924	-5.408732946	0.003808438	0.046433648	-1.947730426
NUP133	-0.563838906	22.90905302	-1.318547735	0.249395878	0.485446888	-6.576719973
SEC63	-0.567496222	22.5533425	-1.722991256	0.150850754	0.34740346	-6.082800746
RTCB	-0.567950438	23.87414727	-2.498840575	0.059031927	0.226631173	-5.083563408
CCT5	-0.570574973	27.36268075	-3.927350507	0.013183864	0.094370948	-3.38767622
CCAR1	-0.570667018	23.13689989	-3.277312069	0.025049945	0.134058879	-4.122879639

SF3A3	-0.571307888	23.7587477	-3.897728883	0.01355606	0.095192709	-3.419750276
LSM7	-0.574642273	24.54610487	-0.925999612	0.40061084	0.577994443	-6.994013361
PSMA5	-0.574719875	26.79756647	-2.089205971	0.096107342	0.264921977	-5.612040986
IMPDH2	-0.577096949	25.39223143	-3.341859666	0.023433422	0.130363002	-4.046958682
PSMD11	-0.57909054	25.66441863	-0.822699184	0.451367193	0.618966868	-7.087926616
CFL1	-0.580081933	28.58960012	-3.105913515	0.030002129	0.151997088	-4.327490913
UCHL5	-0.582832253	24.80106049	-1.971602134	0.11094791	0.280083519	-5.7642049
PSMA2	-0.582926579	25.82377858	-3.887655293	0.013685421	0.095496692	-3.4306889
ACTL6A	-0.583935202	22.95070886	-1.842918862	0.130006157	0.313161053	-5.929880109
UGDH	-0.585074258	7.692721447	-0.05750422	0.956566782	1	-7.469154004
LASP1	-0.585897076	24.54660785	-1.637449564	0.167791154	0.370386972	-6.190566718
MMS19	-0.586019639	22.61789905	-0.875180692	0.424982769	0.598373582	-7.041202761
RFC5	-0.586171643	22.82180412	-1.601237036	0.175531835	0.38074794	-6.235771529
HBS1L	-0.588726591	23.19527952	-2.495902582	0.059233825	0.227011843	-5.087320203
GCN1	-0.592228108	23.77722383	-2.608602218	0.052010316	0.215562676	-4.943760314
RAVER1	-0.593067011	23.82534582	-2.381263539	0.067740801	0.244072348	-5.234421488
MYL12A	-0.593316784	24.45450386	-2.958061397	0.035187865	0.169368669	-4.507382079
TYMS	-0.59335069	24.3027909	-4.882211042	0.005737971	0.058947024	-2.424376363
ANP32A	-0.598068093	27.03101319	-0.909839384	0.408235908	0.582931455	-7.009220264
GLS	-0.601397126	23.21285219	-0.857012228	0.433976729	0.60756742	-7.057615001
PRPS1	-0.601510218	24.49328643	-1.317671501	0.249665866	0.485546499	-6.577735668
ANXA7	-0.603019238	24.25702184	-3.644989581	0.017283429	0.110937039	-3.698967985
ADSL	-0.603353117	24.04043913	-2.295676562	0.074968598	0.255087889	-5.344787106
HNRNPC	-0.603452532	24.94507401	-2.613097926	0.051743247	0.215015478	-4.938058428
TRIP6	-0.603721689	24.52628505	-2.671005135	0.048436808	0.204337029	-4.864800268
DCTPP1	-0.60513808	23.87230661	-1.26627881	0.265989767	0.504843172	-6.636762993
VBP1	-0.608914344	24.24126575	-4.868815189	0.005800563	0.059043347	-2.436982766
CAND1	-0.611127385	24.23375506	-2.794993879	0.042124373	0.187322612	-4.709184884
LCP1	-0.6113416	26.03996878	-5.967594124	0.002546176	0.040356889	-1.479325096
RNF126	-0.614296635	23.93888916	-2.254721804	0.078722188	0.257646807	-5.397719935
DDX50	-0.618891019	23.91386191	-0.65300307	0.545097526	0.687647192	-7.223619693
CYB5R1	-0.620328034	22.32886576	-1.618169788	0.17186855	0.37463292	-6.214667829
TPP1	-0.62074792	23.71598827	-1.514739726	0.195498928	0.411977322	-6.342548276
CA8	-0.62082888	24.03038019	-3.436202968	0.021282005	0.122661736	-3.937132758
RFC2	-0.621128588	23.36948933	-3.813676102	0.014680853	0.100545719	-3.51150412
VPS29	-0.625049065	24.02331639	-3.919270718	0.013284189	0.094783331	-3.396411455
RFC3	-0.633046909	22.93758582	-0.878743824	0.423236278	0.59705105	-7.037955214
KIF2C	-0.633436455	23.83445828	-3.052103445	0.031781481	0.158104138	-4.392607685
COPE	-0.637021994	23.92438499	-1.190059005	0.292028379	0.531148164	-6.722181679
CS	-0.637991243	26.38671581	-1.952289721	0.113609404	0.283237697	-5.789141269
MIF	-0.63819699	28.47349969	-2.566014389	0.054617962	0.220358652	-4.99787234
ATP5F1C	-0.638275091	24.72434923	-5.661725389	0.003161555	0.043088383	-1.73114214
RANBP9	-0.641774515	15.03570153	-0.06434761	0.951406156	1	-7.468648059
SLIRP	-0.642618041	24.12353141	-4.991184215	0.005257465	0.055819683	-2.322731912

SLC25A3	-0.643323038	26.29304512	-3.214205611	0.026755471	0.139367113	-4.197710597
TMEM165	-0.644967218	23.98412247	-1.620432455	0.171384899	0.37394601	-6.211843251
GART	-0.646656818	26.60621567	-5.569522185	0.003380893	0.044130601	-1.809183921
RHOG	-0.647929013	25.03876383	-2.217923257	0.082269862	0.260003361	-5.445329922
GTF3C5	-0.650597774	22.34632452	-1.716328046	0.152105314	0.349400043	-6.091239541
GAGE2C	-0.65662774	27.62306682	-1.894349211	0.122006425	0.298492015	-5.863816078
VCL	-0.657028794	25.91590288	-3.889438198	0.013662421	0.095496692	-3.428751747
TECR	-0.657447388	23.63973213	-3.867121464	0.013953597	0.096458043	-3.453034813
KPNB1	-0.659077324	26.20305641	-5.838553098	0.002786566	0.042351981	-1.584260811
PRDX1	-0.660735129	29.15862141	-5.215143875	0.004411755	0.050987939	-2.118799198
DBN1	-0.660809316	14.61330281	-0.068403753	0.94834869	1	-7.468321405
EEF1E1	-0.669154622	24.22822292	-6.458302434	0.001832007	0.035349763	-1.096726326
MESD	-0.671321899	23.58963898	-3.874206968	0.013860371	0.096113013	-3.44531662
GRB2	-0.672503013	24.02921076	-3.321921819	0.023919575	0.13119852	-4.070342327
PAFAH1B2	-0.682621673	24.13751135	-1.830506636	0.132017663	0.315336055	-5.945786543
SARNP	-0.682848175	23.5850104	-6.367077856	0.00194451	0.035957236	-1.165952221
RAD23A	-0.687856673	23.79050783	-5.484460136	0.00359951	0.045126058	-1.88208597
NUTF2	-0.688033446	24.78839694	-1.275903511	0.262858711	0.501102645	-6.625792098
SLC25A1	-0.695791521	24.53768585	-4.911757475	0.005602722	0.057825299	-2.396657847
HNRNPA1	-0.697982509	26.91218307	-3.425887465	0.021505794	0.123630456	-3.949074694
SF1	-0.699853691	24.57625304	-1.840757306	0.130354146	0.313161053	-5.932651302
TSN	-0.706842034	24.97391316	-2.40090782	0.066192075	0.240890331	-5.209149019
MYCBP	-0.71038253	22.84472471	-3.2972855	0.024536408	0.133120513	-4.099319628
PTPRC	-0.719789672	22.64807606	-2.407808957	0.065657287	0.2396275	-5.200276602
RAN	-0.719824658	28.0232163	-3.898173965	0.013550377	0.095192709	-3.419267337
DDX21	-0.721810386	25.60898538	-3.491810286	0.020120993	0.119062621	-3.873040507
AIP	-0.724428124	15.03514041	-0.07286708	0.94498549	1	-7.467938941
NDUFAF4	-0.724853239	7.381176092	-0.074213513	0.943971185	1	-7.467818831
PSMB3	-0.725408112	24.27522893	-1.145999327	0.308118357	0.531148164	-6.770289194
CNN2	-0.725920196	23.76336704	-4.367014921	0.008852147	0.075260205	-2.927420321
MDH2	-0.726377654	27.94243489	-6.27233942	0.002070227	0.036296778	-1.238744456
HEMGN	-0.728736931	25.91567778	-2.464819811	0.061417319	0.232568312	-5.127108396
RAP1A	-0.729441036	25.04071489	-6.242816791	0.002111378	0.036296778	-1.261618289
CLTA	-0.730776431	23.71295899	-4.005117432	0.012262189	0.092108371	-3.304117201
MOV10	-0.732305394	22.13982454	-2.347201649	0.070521484	0.24997951	-5.278297846
MCM4	-0.732579157	25.35436665	-2.343294129	0.070848409	0.250338566	-5.283335481
TARDBP	-0.732995656	24.66053786	-5.129598698	0.004714208	0.052043914	-2.195918207
DNAJC7	-0.734620161	23.54813092	-4.684193364	0.00675127	0.064662285	-2.613254822
RPL17	-0.736800912	26.54575062	-1.78275905	0.14006207	0.329584023	-6.006817279
PEBP1	-0.743868185	26.92292971	-4.177496949	0.010475121	0.082719905	-3.122212917
FXR1	-0.748153133	23.33228656	-2.646514854	0.049805443	0.208919242	-4.895740175
SART3	-0.748435293	23.02665059	-2.597265869	0.052690613	0.216519388	-4.958147064
PSMD2	-0.74967727	24.85969028	-4.258668449	0.009740489	0.079172695	-3.038122058
THRAP3	-0.750032114	23.6631768	-3.968799563	0.012682911	0.093131547	-3.343023697

ALDH4A1	-0.750584669	22.04720154	-1.339682957	0.242966575	0.478838438	-6.552130473
PSMG1	-0.752870714	23.60494876	-1.046504054	0.347381209	0.536595541	-6.875100586
PPIA	-0.755963848	29.8669709	-4.31191208	0.009291451	0.077219962	-2.983501956
UBR4	-0.760912754	23.68661848	-5.587372269	0.003337048	0.044027317	-1.793996338
ESD	-0.761110606	24.50596971	-1.052540508	0.344880516	0.536128553	-6.868902872
EIF4B	-0.76635212	25.18139965	-8.314584635	0.000622662	0.016637327	0.15026577
EIF3I	-0.770700043	26.15389356	-2.587587967	0.053279245	0.217901685	-4.970439084
NSF	-0.772109638	22.4782038	-2.292305086	0.075270103	0.255087889	-5.349142099
HAT1	-0.775880812	25.48542653	-5.723194025	0.003024739	0.042642192	-1.679672195
RRP1B	-0.775999749	22.66412117	-2.265230228	0.077739907	0.257646807	-5.384132026
QPRT	-0.778957958	24.56039998	-2.383975012	0.06752468	0.244034634	-5.230931714
HMGB3	-0.78341581	23.96784082	-1.350745038	0.239664429	0.473988741	-6.5391926
TMPO	-0.786880087	25.78880219	-4.098520401	0.011253071	0.087309667	-3.204989095
SRP9	-0.790824857	24.06537958	-1.537488216	0.190038581	0.405087043	-6.314643872
AASDHPPT	-0.794236829	23.64007382	-1.786911309	0.139342757	0.328239467	-6.001520603
PSMB2	-0.796494964	24.9245442	-3.546263336	0.0190545	0.115584687	-3.810742355
ARF6	-0.802229417	23.51926338	-3.577978912	0.018463542	0.114832887	-3.774669665
GALM	-0.804327995	22.29651503	-3.900764366	0.013517359	0.095192709	-3.41645722
NUP160	-0.81107996	22.87293938	-1.500101744	0.199094168	0.417965902	-6.360431577
HMGB2	-0.816059161	26.33056325	-7.67143086	0.000881587	0.021497164	-0.24981141
PPM1F	-0.817688673	23.60858459	-1.654288614	0.16430996	0.365700903	-6.169456614
HDAC2	-0.818000054	24.35215988	-2.424550818	0.064379596	0.237701036	-5.178765862
YME1L1	-0.820488982	23.40342862	-2.01658271	0.105001995	0.271706248	-5.706057456
FH	-0.820641277	26.31377876	-3.49594105	0.02003771	0.118886839	-3.868298511
CYB5R3	-0.823669519	23.91294619	-5.024108352	0.005121785	0.055171068	-2.292336725
MOGS	-0.833657981	22.46340351	-2.343762777	0.070809112	0.250338566	-5.282731249
HSPA14	-0.834431897	23.0926614	-1.289616688	0.258456922	0.496550571	-6.610093251
IDH1	-0.84087642	23.74478972	-9.863459476	0.000294978	0.011983694	1.001369825
SFPQ	-0.843651508	26.07115502	-1.434892628	0.215914209	0.441579383	-6.439355558
UBAP2	-0.845457023	23.13040918	-3.331969483	0.023673142	0.130556347	-4.058550615
BCCIP	-0.851324448	24.31633338	-1.493242862	0.200801113	0.420753229	-6.368790965
TBRG4	-0.853579425	24.18965946	-2.777412119	0.042960428	0.190154421	-4.731142489
ACO2	-0.855021159	23.92231922	-5.340757256	0.004008383	0.047564717	-2.007260378
PBK	-0.855678948	23.60643804	-3.033423463	0.032427096	0.159425885	-4.4153084
PNPT1	-0.859983271	22.55149784	-1.76439688	0.143289324	0.33575397	-6.030213795
PSAT1	-0.864970788	24.59842008	-2.845094444	0.039840881	0.183037089	-4.646822316
SERPINB1	-0.875455584	23.78457132	-1.294394287	0.256939614	0.494921009	-6.604605485
CBS	-0.877334232	25.01346008	-3.438101254	0.021241116	0.122661736	-3.934936956
FUBP1	-0.879433554	25.39852252	-7.282146848	0.001101989	0.024700132	-0.50754232
RBM25	-0.88025197	23.27332077	-4.289306447	0.00947906	0.077616364	-3.00664012
LAMTOR3	-0.883929358	23.6471354	-4.335648746	0.009099138	0.076771811	-2.959288248
NCKAP1	-0.895453942	22.37210841	-1.969716746	0.11120481	0.280413037	-5.766640196
ARL8B	-0.901967677	23.38068332	-3.345863413	0.023337174	0.130363002	-4.042270259

OAT	-0.904942176	26.09156272	-8.560149999	0.000548694	0.016453412	0.295223962
TXNRD1	-0.910519251	24.23041107	-2.005821965	0.106392951	0.272931743	-5.719975644
CSTB	-0.911893523	25.54488482	-6.882483467	0.001401057	0.029311382	-0.785552117
TSEN15	-0.916584786	22.97593356	-3.448108952	0.021027047	0.121825108	-3.923369938
PHF6	-0.917102498	23.35298242	-3.089976194	0.030516998	0.153783279	-4.346734081
PMPCA	-0.918588322	22.82425518	-1.930415906	0.116705586	0.289351616	-5.817359085
GIGYF2	-0.919528412	22.44480495	-3.694821271	0.016462453	0.108206126	-3.643127252
DDOST	-0.925232182	24.87405996	-5.841670946	0.002780446	0.042351981	-1.581703261
CTPS1	-0.934873438	24.10562347	-3.139019799	0.028964005	0.147410842	-4.287634157
RPL29	-0.935445618	26.6516779	-1.166982557	0.300358621	0.531148164	-6.747500285
BSG	-0.93848027	25.19078405	-4.65448866	0.006920923	0.065351182	-2.642062452
CYB5B	-0.938756479	25.37116958	-5.197619673	0.00447179	0.051290906	-2.134520102
NAP1L1	-0.940317036	26.6906283	-3.656178575	0.017095026	0.110543107	-3.686396019
UBR5	-0.943449924	15.04845124	-0.094802984	0.928477246	0.991000967	-7.465709039
ACIN1	-0.943783405	22.4786013	-2.517164531	0.057789808	0.22439954	-5.060149017
ACACA	-0.94722362	23.63078022	-4.91845361	0.005572597	0.057825299	-2.390392504
FKBP5	-0.961845453	24.13798943	-6.868255651	0.001413392	0.029311382	-0.795713121
DNM1L	-0.962654929	22.11840328	-5.544235924	0.003444186	0.044693849	-1.83076401
HNRNPL	-0.98495319	26.4611603	-3.204703876	0.027023641	0.140106213	-4.209028961
ESYT1	-0.98787933	24.32641278	-8.335348815	0.000615959	0.016637327	0.16268286
STAT5A	-0.988582482	23.80831882	-3.567780516	0.018651222	0.114832887	-3.786252063
TK1	-0.99017108	24.41767482	-5.6606425	0.003164031	0.043088383	-1.732052859
GNAI2	-0.995172366	23.27941595	-4.267420824	0.009664951	0.078847526	-3.02911422
SDHA	-0.999744041	24.92668854	-6.527558856	0.00175178	0.034399998	-1.044728905
TSNAX	-1.000213782	23.2039861	-1.641482833	0.166950605	0.369495044	-6.185515447
LBR	-1.000771709	24.54480565	-2.555753377	0.055267807	0.221370512	-5.010935751
ALYREF	-1.012276167	24.96760697	-4.587926051	0.007319721	0.067117603	-2.707071081
DDI2	-1.012577634	22.9446392	-1.324600629	0.24753835	0.484380598	-6.569695475
TNPO3	-1.01316207	23.73181177	-2.79716129	0.042022583	0.187322612	-4.70648062
LUC7L2	-1.026751434	25.29951815	-5.65590551	0.003174888	0.043088383	-1.73603833
SPCS2	-1.030489654	23.76405196	-3.126973739	0.029336911	0.148967058	-4.302118136
ECPAS	-1.038325053	23.29826378	-4.079845197	0.011446812	0.087703466	-3.224701938
HSP90B1	-1.05220973	28.28924822	-1.596028088	0.176674459	0.382478659	-6.242251201
STAT5B	-1.053628101	23.61551602	-4.180448191	0.010447296	0.082719905	-3.119138076
CORO7	-1.055318863	22.19535755	-4.757071364	0.00635545	0.063240998	-2.543105126
SLC2A1	-1.058719504	24.34488793	-2.524107764	0.05732675	0.22439954	-5.051284433
GSTK1	-1.067577971	24.18888426	-4.40031607	0.008598509	0.074819745	-2.893746107
KPNA4	-1.068653687	23.65149999	-3.249191327	0.025794037	0.136930544	-4.156151491
VAPA	-1.070551574	25.99715009	-8.422244996	0.000588839	0.016637327	0.214324017
MAP4	-1.072455252	25.00810432	-6.055051615	0.002397292	0.038922012	-1.40925682
TPT1	-1.080432858	26.38993733	-5.016761935	0.005151695	0.05522518	-2.299106298
RPA3	-1.080739381	24.09669894	-2.476438285	0.060590886	0.230224616	-5.112226892
RIC8A	-1.088876333	24.08315953	-1.687195803	0.157716925	0.35553268	-6.128050963
RANBP1	-1.093251599	27.8233821	-6.308581923	0.002021016	0.036296778	-1.210788157

NCLN	-1.096342966	22.88467614	-4.149611567	0.010742373	0.083934245	-3.151331424
SARS1	-1.097161808	24.88751245	-7.329153002	0.001072109	0.024577287	-0.475757219
POR	-1.098824108	23.42492423	-2.43579919	0.063536564	0.236160192	-5.164324392
SNRNP40	-1.098902237	23.43424984	-1.356165523	0.238061974	0.472466961	-6.532836334
CCDC86	-1.104630189	15.46199071	-0.108030466	0.918542217	0.983709064	-7.46408353
CPPED1	-1.115276872	22.92074808	-1.558813074	0.185057436	0.398295297	-6.288366907
ADD1	-1.11662525	22.80705225	-4.532960678	0.007669613	0.069182399	-2.76123258
RPS7	-1.118102255	27.47504393	-5.85150197	0.002761254	0.042351981	-1.573646167
CDK6	-1.121215467	23.6633308	-2.209041616	0.083151902	0.261351374	-5.456826499
GTF2I	-1.12600534	23.63677892	-4.000736551	0.012312063	0.092108371	-3.308799545
MYH10	-1.126763659	24.57248677	-5.717889789	0.003036262	0.042642192	-1.684096209
SNRPC	-1.129167838	25.88579583	-0.972635688	0.379255959	0.559553839	-6.949117648
ADK	-1.130135692	24.11196223	-1.477861538	0.204681457	0.425165331	-6.387489223
RPA2	-1.132637843	23.90279445	-1.914048036	0.119080553	0.292915099	-5.838453889
SMARCC1	-1.150068933	22.84679563	-5.070779189	0.004936506	0.053961116	-2.249498148
CERS2	-1.155919941	22.71317054	-2.065932945	0.098866658	0.265979027	-5.642181915
GALK1	-1.163110079	23.63050815	-11.4217959	0.000154071	0.008140071	1.728344489
NUP214	-1.166156486	24.01084974	-5.341614288	0.004005786	0.047564717	-2.006506234
TXNDC12	-1.177828794	23.57902598	-3.032564901	0.032457127	0.159425885	-4.416352936
MAPRE2	-1.180089272	23.49083855	-2.371136326	0.068554753	0.244964569	-5.247459599
DLST	-1.192747183	23.68903931	-1.696164441	0.155967168	0.354601584	-6.116733157
HINT1	-1.195287252	25.7460858	-3.819793962	0.014595402	0.100269957	-3.504788635
BUD31	-1.195629635	22.72090038	-2.943994251	0.035732275	0.170433059	-4.524654242
ABCF2	-1.205196801	24.19200043	-7.132855287	0.001203761	0.026446996	-0.609744719
H4C9	-1.215991618	25.09697571	-5.885926513	0.002695287	0.041824071	-1.54551952
PNN	-1.220476909	22.31098856	-2.975908459	0.034510675	0.168245682	-4.485507194
PSMD7	-1.222785336	25.07739169	-3.999327078	0.01232816	0.092108371	-3.310306636
FLOT1	-1.224997389	23.17862085	-1.825163474	0.13289352	0.316406353	-5.952628933
RFC4	-1.23354908	23.51225663	-4.171085287	0.010535873	0.082904614	-3.128897653
SYNE1	-1.235506467	8.163405738	-0.114187297	0.913923637	0.981130406	-7.463254984
RAB21	-1.240021629	23.172643	-4.917562349	0.005576596	0.057825299	-2.391226075
CUL1	-1.248347536	22.67443775	-2.95983916	0.035119737	0.169368669	-4.505201169
GDI2	-1.25868917	26.26032032	-11.48392822	0.000150397	0.008139758	1.755071852
EIF2B1	-1.266133122	23.16963232	-11.30743197	0.000161122	0.008201743	1.678729014
ABHD14B	-1.287514326	24.41695136	-1.746949938	0.146426789	0.342022152	-6.052402189
CDK1	-1.296881877	26.22789812	-1.242246929	0.273959407	0.513009218	-6.663980645
PAPSS1	-1.304353788	22.95219865	-2.501695911	0.058836441	0.226631173	-5.079912994
BCAT1	-1.308109803	24.20196605	-4.331323667	0.009133828	0.07677259	-2.963693993
HBA2	-1.317494991	24.92580103	-6.241122458	0.002113769	0.036296778	-1.262933806
EBP	-1.322946172	23.99677352	-4.961153704	0.005384953	0.056900998	-2.35058249
WDR77	-1.326559984	24.27763492	-2.726519503	0.045487727	0.196758805	-4.794907764
EIF4H	-1.335348768	25.01084363	-4.569948974	0.007432042	0.067588943	-2.724737316
CSNK2A1	-1.341803338	23.87845683	-2.9654787	0.034904606	0.169112053	-4.498285564
SF3B1	-1.345326983	24.71711149	-7.858108927	0.000794899	0.019598683	-0.130506499

PTGES3	-1.345664023	27.26026105	-12.01474417	0.000122973	0.006821915	1.97708994
HACD3	-1.36790559	23.94907346	-6.850612047	0.001428871	0.029358006	-0.808339501
PAK2	-1.372249914	24.09596588	-3.949592789	0.012912225	0.093193597	-3.363681846
YTHDF2	-1.377269772	23.92679223	-7.349888387	0.001059238	0.024577287	-0.461795353
NASP	-1.398523681	25.95654029	-7.095259299	0.001231149	0.026783516	-0.635787357
PICALM	-1.429592036	24.90992036	-6.463549664	0.001825776	0.035349763	-1.092769979
CDK2	-1.445312486	23.92811424	-1.915703114	0.118838106	0.292676755	-5.836321677
IARS2	-1.447906052	22.99056302	-6.062554064	0.002385012	0.038922012	-1.403285067
GDI1	-1.45047043	25.68241018	-9.644371805	0.000325632	0.012152462	0.889541044
SNAP23	-1.45840838	22.6999676	-3.928695161	0.013167253	0.094370948	-3.386223467
IGF2BP1	-1.461595441	25.21900929	-8.60098735	0.000537446	0.016336879	0.318936197
PSAP	-1.474486031	25.42311952	-8.246439166	0.000645284	0.016649824	0.109302802
PIN4	-1.476579146	23.80270552	-6.392899277	0.001911846	0.035696381	-1.14627205
UBE2N	-1.482151927	25.70811392	-4.368265941	0.00884246	0.075260205	-2.926152318
GEMIN4	-1.485441783	24.5111121	-1.215432861	0.283111293	0.522214429	-6.694043343
NSUN2	-1.488571627	23.71959493	-9.729887475	0.000313229	0.011983694	0.933499584
NUP98	-1.499839062	23.20573631	-1.032227076	0.35335774	0.541161744	-6.889671651
ANXA2	-1.502489721	26.68801987	-14.08574311	6.03E-05	0.004057182	2.74753874
TFB1M	-1.536112841	6.36361613	-0.181323984	0.863860702	0.947088388	-7.451267987
PPIL1	-1.538598968	23.69567168	-3.771410464	0.015287389	0.102796112	-3.558057818
THG1L	-1.550971965	8.256226323	-0.173328477	0.869789518	0.947502671	-7.452978084
RPL38	-1.559209682	24.665032	-3.976068279	0.012597372	0.093032371	-3.335220591
PPIH	-1.568418902	23.77325327	-3.702092408	0.01634649	0.108206126	-3.635011705
MPST	-1.589806455	16.77623108	-0.141984163	0.893123772	0.967696118	-7.458946696
USP15	-1.601651018	23.76985882	-7.21047101	0.001149503	0.025507465	-0.556370635
MYH9	-1.634295841	25.34785432	-3.480665444	0.020347696	0.119075217	-3.885847634
BOP1	-1.64775008	22.58468301	-4.499668378	0.00789114	0.070892464	-2.79425051
NUP153	-1.712825489	23.50560274	-2.000777601	0.10705173	0.273672567	-5.726498643
IDH2	-1.719611987	23.61708615	-5.055373437	0.004996772	0.054352141	-2.263606942
KTN1	-1.720782265	22.47971776	-6.163922361	0.002226245	0.037710217	-1.323194373
SNX5	-1.741610199	16.01582872	-0.205002395	0.846365313	0.933905832	-7.445758475
TGM2	-1.758981976	23.98518392	-13.02682474	8.57E-05	0.005431385	2.371327017
RPL31	-1.759127997	25.98390679	-3.693598352	0.016482051	0.108206126	-3.644493001
PDCD6	-1.768700335	23.75906847	-7.044447243	0.001269363	0.027185526	-0.671182878
DFFA	-1.818851851	23.96713652	-5.031537709	0.005091745	0.05511504	-2.285498043
PHACTR4	-1.837916375	8.371294824	-0.203556022	0.847431182	0.934617193	-7.446114055
RBBP4	-1.849373534	26.39443601	-10.1181976	0.000263609	0.011412944	1.128201607
TXLNA	-1.916099061	23.84951348	-1.758944676	0.144262279	0.337677213	-6.037152225
PHGDH	-1.936646715	23.19754018	-5.751812741	0.002963456	0.042425216	-1.655859084
SCAF4	-2.068907234	23.29679424	-14.93242262	4.64E-05	0.003217954	3.024840445
NDUFA9	-2.091947584	14.22207506	-0.218948767	0.836107814	0.926272211	-7.442203199
IGF2BP3	-2.134088665	24.13503564	-9.761821006	0.000308744	0.011983694	0.94981274
BLVRA	-2.140713355	23.30114989	-3.343228711	0.023400459	0.130363002	-4.045355244
RBBP7	-2.273835853	25.86224707	-9.268977513	0.00038764	0.012698543	0.691734782

RNPEP	-2.318340912	24.02889501	-4.392675365	0.008655939	0.074819745	-2.901457912
NUDT5	-2.401230252	23.85949487	-2.456110061	0.06204503	0.23339403	-5.138271124
LTA4H	-2.509538501	24.39521805	-17.12340866	2.50E-05	0.001791059	3.660070473
NSA2	-2.518261244	5.818928477	-0.32003291	0.76302109	0.874064523	-7.409655311
AHNAK	-2.659206116	23.83130686	-12.42197574	0.00010597	0.006237778	2.140105025
KEAP1	-2.721222096	5.839243608	-0.343399921	0.746492316	0.859608951	-7.400463553
ABCB10	-2.837285459	5.937348044	-0.351664917	0.740683116	0.853805629	-7.397065108
MRPS34	-3.16687043	20.69276108	-1.256217349	0.269299931	0.508575784	-6.648189087
CUL2	-3.184789945	22.04310051	-1.941260741	0.115159544	0.285837839	-5.803372609
COMT	-3.334098597	24.59940316	-4.078404242	0.011461922	0.087703466	-3.226225175
PCM1	-3.414233481	23.86730993	-1.83385363	0.131472083	0.314371285	-5.941498889
FAM98B	-3.667505556	24.36874924	-2.520713926	0.057552575	0.22439954	-5.055616901
KPNA6	-3.858337686	21.13707825	-1.428642758	0.217597214	0.443820061	-6.446852259
WDHD1	-4.156975655	17.10141353	-0.365147621	0.73124964	0.848219002	-7.39135714
DOCK11	-4.579471329	4.58528194	-0.676036013	0.531631833	0.677593933	-7.206671468
ANXA6	-4.75060984	4.688934213	-0.683817578	0.527134434	0.673408928	-7.200836867
MRPS21	-4.816279293	4.68068534	-0.692271656	0.52227825	0.669904876	-7.194436483
SIN3A	-4.886027473	4.698968423	-0.698019577	0.518994371	0.667564184	-7.190048507
KIFC1	-4.950557798	12.05244574	-0.54709904	0.609890151	0.747429752	-7.295116644
SURF6	-5.109220722	10.33195675	-0.536865953	0.616392902	0.751938345	-7.301444974
OXSR1	-5.185359637	20.72261747	-1.38561776	0.229532194	0.459836852	-6.498113681
SREK1	-5.270505148	9.739948972	-0.687540347	0.524992161	0.672246501	-7.198026316
RAB11FIP1	-5.359547835	5.009580701	-0.713759965	0.510075832	0.659206914	-7.177882943
SAAL1	-5.393346808	17.27126584	-0.775221707	0.476301586	0.63683646	-7.128337728
CASP6	-5.577490629	5.111744308	-0.724713527	0.503933909	0.65431793	-7.169289242
GTF3C1	-5.663519124	5.151215152	-0.728971923	0.501560438	0.652381367	-7.165920234
MDN1	-5.684888769	10.05457756	-0.621955262	0.563608589	0.70578299	-7.245690664
POLR2H	-5.758719044	19.85131018	-0.907966255	0.40912726	0.583453336	-7.010970933
MIA3	-5.987524018	4.137910542	-0.886988226	0.419217051	0.594784295	-7.030405165
NARF	-6.087059523	4.205975487	-0.887090315	0.419167473	0.594784295	-7.030311361
TBR1	-6.343608168	19.53771947	-0.828695698	0.448289901	0.617364205	-7.082695031
CCDC50	-6.43306575	18.02213367	-0.929453209	0.398996372	0.576039655	-6.990739715
DIS3	-6.434672733	18.26091203	-0.914713984	0.405923607	0.580440797	-7.004652654
YLPM1	-6.487002034	4.315339607	-0.909179204	0.408549885	0.583004626	-7.009837569
CWC15	-6.549774394	10.81624923	-0.678954257	0.529942132	0.676217131	-7.204489762
KBTBD3	-6.569760248	20.81960809	-0.794470393	0.466070623	0.632093932	-7.112172665
NOLC1	-6.595556295	18.61884255	-0.920864015	0.403021414	0.578089539	-6.998865944
AARS2	-6.611002748	10.83042792	-0.685026286	0.526438219	0.672906917	-7.199925698
RNPS1	-6.642675183	19.38359243	-0.885003042	0.420182064	0.595012125	-7.032227714
LAMTOR2	-6.681638025	11.22135968	-0.666359072	0.537261334	0.680621367	-7.213850906
VAMP7	-6.703881352	19.21921876	-0.90402175	0.411009427	0.585761027	-7.014649363
KNSTRN	-6.750199403	10.91082776	-0.695637415	0.520353583	0.668208681	-7.191870614
ODAD4	-6.754677296	11.34651558	-0.666153973	0.537381088	0.680621367	-7.214002151
DCUN1D1	-6.781813073	18.87902788	-0.936849218	0.395556762	0.572937634	-6.983701205

EBNA1BP2	-6.814158615	19.20450779	-0.923021785	0.402007158	0.578089539	-6.996829337
SCD	-6.841674108	19.23438203	-0.920208883	0.403329768	0.57815811	-6.999483642
WDR36	-6.841818926	10.72009539	-0.720306049	0.506398917	0.655817724	-7.172759689
DARS2	-6.847645417	18.87812656	-0.94707867	0.390839429	0.569450226	-6.973904103
ACO1	-6.852541986	18.39564625	-0.979418678	0.37622994	0.557312575	-6.942466195
LSM6	-6.875638815	19.36498491	-0.923450401	0.401805934	0.578089539	-6.996424399
MRPS36	-6.882926448	19.02894323	-0.944897085	0.391841581	0.569787987	-6.975999493
MRPS2	-6.887957828	18.91775011	-0.952158939	0.388513879	0.567552533	-6.969012004
AP1M1	-6.900389808	18.66871347	-0.968226751	0.381233712	0.560608089	-6.953424813
SLC25A10	-6.911805122	19.07715256	-0.945492904	0.391567671	0.569763057	-6.975427537
HCCS	-6.935496688	19.53884488	-0.922808843	0.402107159	0.578089539	-6.997030467
NDUFA8	-6.945635487	18.8001382	-0.968649951	0.381043502	0.560608089	-6.953011941
GOLGA2	-6.948452238	18.28875413	-1.003650277	0.365584294	0.549242755	-6.918460313
ELOVL1	-6.984921872	18.81025934	-0.975728956	0.377873488	0.558256505	-6.946088117
ESYT2	-6.998208768	10.67984838	-0.892021593	0.416778204	0.592840278	-7.025771255
NUDT3	-7.014759756	19.24197525	-0.953752374	0.387786818	0.566863603	-6.967473984
PRPF6	-7.037648163	19.20084285	-0.960263361	0.384827608	0.564396868	-6.961171636
ITGB1	-7.038447041	18.9305333	-0.976934651	0.377335767	0.557833489	-6.94490556
HEXIM1	-7.054878806	19.1966459	-0.96295646	0.383609078	0.563354431	-6.958556514
PRKRA	-7.075645824	10.96222406	-0.729564078	0.501231029	0.65233528	-7.165450515
CLPX	-7.090570837	18.73171098	-0.996762004	0.36858443	0.552423998	-6.925322845
ALDH2	-7.097506112	19.11411105	-0.974933591	0.378228558	0.558409296	-6.946867696
NIFK	-7.103608821	18.81211286	-0.995357797	0.36919856	0.552428594	-6.926718084
ERG28	-7.10803792	3.55401896	-1.069988948	0.337739454	0.531148164	-6.850867022
ARMC1	-7.116457275	19.02785072	-0.978357539	0.376702001	0.557639587	-6.943508749
APMAP	-7.116469139	3.55823457	-1.069989133	0.337739379	0.531148164	-6.85086683
DDX20	-7.121860131	18.45494996	-1.022719134	0.357386502	0.54504512	-6.89930645
ENY2	-7.132631417	18.97803701	-0.989934674	0.371578443	0.554090193	-6.932094678
PLAA	-7.139931254	18.7021158	-1.005342484	0.364850426	0.549026601	-6.916769812
PTCD3	-7.161544221	11.40573846	-0.707206825	0.513775625	0.662445155	-7.182974248
LSM12	-7.224662767	19.16253223	-0.993173309	0.370155655	0.553077698	-6.928886106
MAGED2	-7.23114659	3.615573295	-1.069991591	0.337738382	0.531148164	-6.850864277
REEP5	-7.232870007	19.76768796	-0.95825936	0.385736422	0.564982918	-6.963114459
PRPF4	-7.238189215	18.86476115	-1.015587479	0.360433988	0.547413899	-6.906496556
SCAMP3	-7.247329402	19.78404261	-0.959449871	0.385196312	0.564564475	-6.961960617
SMNDC1	-7.248828713	19.37568027	-0.984000025	0.374197538	0.555786035	-6.937956634
CCDC6	-7.251638605	18.11953962	-1.069099033	0.338100541	0.531148164	-6.851791213
NDUFA5	-7.251946478	19.42912995	-0.98179817	0.375173189	0.556862412	-6.940125697
MLEC	-7.256327925	11.29159556	-0.725848128	0.50330074	0.653878421	-7.168393137
NCBP2	-7.258394035	18.67489988	-1.032181611	0.353376912	0.541161744	-6.889717854
EMC3	-7.260025517	10.77450865	-0.765724998	0.481410318	0.637802859	-7.13620106
RCSD1	-7.278302214	3.639151107	-1.069992568	0.337737986	0.531148164	-6.850863262
VAMP8	-7.290417382	19.41658111	-0.988756604	0.372097126	0.554150015	-6.933260134
BST2	-7.299440872	19.2012311	-1.003737266	0.365546539	0.549242755	-6.918373457

PYM1	-7.304166195	19.05150684	-1.012121673	0.361922939	0.547821966	-6.909979308
SEC23IP	-7.30579082	11.41540685	-0.722482045	0.505180856	0.655187495	-7.171048413
CPSF7	-7.306173349	19.05373865	-1.014470551	0.360913265	0.547413899	-6.907619767
IDH3G	-7.307260151	19.03482886	-1.014793489	0.360774636	0.547413899	-6.907295092
PRPF31	-7.333670438	18.81762237	-1.035784632	0.351860317	0.540705017	-6.886052381
TRMT2A	-7.339971046	10.87629091	-0.767069035	0.480684847	0.637802859	-7.135092726
TRIR	-7.361732456	19.34332074	-1.005422651	0.364815691	0.549026601	-6.916689681
IST1	-7.368005281	18.27019427	-1.08054626	0.333481296	0.531148164	-6.839868129
MTX1	-7.387014532	19.30998686	-1.012271143	0.361858618	0.547821966	-6.909829263
RDH11	-7.389383225	19.36493719	-1.008671143	0.363410503	0.548723914	-6.913439219
MRPS17	-7.390857782	20.2825381	-0.950464603	0.389288211	0.568309566	-6.97064553
TMED7	-7.393552602	3.696776301	-1.069994877	0.337737049	0.531148164	-6.850860863
WDR44	-7.394715004	10.74907279	-0.783773157	0.471735874	0.634874809	-7.121193523
ODR4	-7.399476638	3.699738319	-1.069994993	0.337737002	0.531148164	-6.850860742
LAMP1	-7.41868457	19.70172068	-0.992661703	0.370380108	0.553077698	-6.929393412
ATP6V1F	-7.419675432	11.53539499	-0.726667546	0.502843815	0.653667502	-7.167745272
CMAS	-7.42140889	19.08084295	-1.032459673	0.35325967	0.541161744	-6.889435255
COASY	-7.423705415	11.02408147	-0.762993588	0.482887135	0.637813425	-7.138448837
GNB1L	-7.425852676	19.05801232	-1.034894146	0.352234624	0.540905627	-6.886959038
RAB3GAP2	-7.42755459	18.26765281	-1.091976289	0.328924005	0.531148164	-6.827888099
HBQ1	-7.429675647	10.99438767	-0.768247816	0.480049242	0.637802859	-7.134119434
METAP1	-7.436581268	18.54736023	-1.069980259	0.337742978	0.531148164	-6.850876048
CSMD2	-7.444580666	3.722290333	-1.069995866	0.337736648	0.531148164	-6.850859836
VPS45	-7.445447778	11.11792684	-0.760333329	0.484328684	0.639336913	-7.140632084
ZNF428	-7.445582754	18.73153997	-1.06138823	0.34124328	0.532877437	-6.859779658
ZMPSTE24	-7.453834139	19.88835355	-0.986608944	0.373044264	0.555074065	-6.935382484
TPMT	-7.462091652	18.66769864	-1.068614077	0.338297455	0.531148164	-6.852294654
PIP4K2A	-7.46871645	18.97652636	-1.047285717	0.347056515	0.536595541	-6.874299269
CNOT1	-7.472495183	18.16453258	-1.107022582	0.32300814	0.531148164	-6.812005752
DDX18	-7.482355389	18.88755449	-1.056792961	0.343128205	0.534692055	-6.86452379
HIBCH	-7.495716227	18.73480639	-1.069842348	0.337798915	0.531148164	-6.8510193
PPT2	-7.512936736	11.05112744	-0.773458067	0.477247282	0.637055355	-7.129803667
MRPS7	-7.524363273	18.90126358	-1.06289876	0.340625638	0.532662644	-6.85821748
POLR2A	-7.524926547	3.762463273	-1.069997382	0.337736034	0.531148164	-6.850858261
PHPT1	-7.552123022	20.00405495	-0.995839724	0.368987692	0.552428594	-6.926239377
DNAH17	-7.584579699	11.10621001	-0.777376766	0.475147891	0.636686697	-7.126542964
TRA2A	-7.596381422	19.4727669	-1.037092039	0.351311379	0.540235586	-6.884720357
PLCH1	-7.597182478	3.798591239	-1.069998704	0.337735497	0.531148164	-6.850856887
GMNN	-7.612135934	11.26694721	-0.768056053	0.480152599	0.637802859	-7.134277846
WDR18	-7.616662665	11.05783658	-0.784920961	0.471125542	0.634874809	-7.120230049
CTSB	-7.618153968	19.73118135	-1.023779076	0.356935446	0.544731606	-6.898235125
TRAPPC3	-7.619349519	19.00182708	-1.072893704	0.336563167	0.531148164	-6.847847187
SNX6	-7.622742284	11.46240702	-0.754415793	0.487546619	0.641586878	-7.14546732
YTHDF1	-7.648328648	11.16809671	-0.779836808	0.473833467	0.635693145	-7.124489543

NDUFS7	-7.65217773	11.38147364	-0.763772966	0.482465402	0.637802859	-7.137808091
SARS2	-7.653294798	18.88312485	-1.087614474	0.330656647	0.531148164	-6.83246855
LANCL1	-7.671307393	3.835653697	-1.070000022	0.337734963	0.531148164	-6.850855518
SPAG9	-7.672918173	19.40714965	-1.053043535	0.344672832	0.536128553	-6.868385425
PPP6R1	-7.674501666	18.98291928	-1.083102016	0.332457525	0.531148164	-6.837195844
GTF2F2	-7.689365715	11.10857188	-0.789297401	0.468803833	0.633927913	-7.116546591
ZC3HC1	-7.696626035	11.2173012	-0.781516803	0.472937386	0.634874809	-7.123084384
DSTN	-7.716920491	19.61423439	-1.047756063	0.346861265	0.536595541	-6.87381692
HSD17B12	-7.718552325	18.90313764	-1.092592218	0.328679982	0.531148164	-6.827240431
LSM14B	-7.722167899	10.87983342	-0.811808437	0.456997424	0.623688933	-7.097355871
UBE2E1	-7.726916454	19.3215749	-1.068316475	0.338418344	0.531148164	-6.852603532
SNX1	-7.761979683	18.95893245	-1.101211718	0.325281664	0.531148164	-6.818154446
RANBP3	-7.763781629	19.0529639	-1.095048037	0.327708593	0.531148164	-6.824655942
NF1	-7.772365118	3.886182559	-1.070001759	0.337734258	0.531148164	-6.850853714
CLASP1	-7.775019017	11.27354077	-0.786055629	0.470522774	0.634874809	-7.119276545
TMEM126A	-7.788180738	11.40063141	-0.777576454	0.475041095	0.636686697	-7.126376468
SPC25	-7.796734418	18.68386279	-1.128169867	0.314852148	0.531148164	-6.789472954
TMED2	-7.79776352	19.73054119	-1.053876693	0.344329083	0.536128553	-6.867528055
USP9X	-7.805998009	18.38828583	-1.152793159	0.305586501	0.531148164	-6.762935496
SUPV3L1	-7.817489195	18.62696762	-1.13641865	0.311720641	0.531148164	-6.780618473
TXLNG	-7.836798116	18.26411775	-1.167440344	0.300191303	0.531148164	-6.747000601
DAD1	-7.84377057	20.36974203	-1.02037874	0.358384171	0.545816386	-6.901669511
LDAH	-7.862896183	18.44333573	-1.159250616	0.303197296	0.531148164	-6.755923824
FBL	-7.873294299	19.38031697	-1.08935297	0.32996511	0.531148164	-6.830644201
ARPC5	-7.889002236	19.74142309	-1.068551707	0.338322787	0.531148164	-6.852359392
PTPN11	-7.910915553	18.72125189	-1.145528691	0.308294442	0.531148164	-6.770797726
DIMT1	-7.936691932	18.35349371	-1.180062595	0.295610937	0.531148164	-6.73318176
DPH3	-7.938439588	11.30636538	-0.802001727	0.462112814	0.629432916	-7.105766086
METTL7A	-7.954564396	19.32907378	-1.108541803	0.322416052	0.531148164	-6.810395131
LSM3	-7.970872145	20.61106986	-1.025845849	0.35605733	0.543765462	-6.89614416
COPS7B	-7.981335666	11.16087526	-0.818738053	0.453408828	0.621058141	-7.091367
LYPLAL1	-7.98998273	10.99469828	-0.833332747	0.445921339	0.616126682	-7.078630271
GHITM	-7.998172692	19.35331064	-1.11465293	0.320044011	0.531148164	-6.803903536
ZMIZ2	-8.004127398	4.002063699	-1.070005496	0.337732743	0.531148164	-6.850849832
SLC29A1	-8.013520077	18.89201141	-1.151271086	0.306152107	0.531148164	-6.764585078
RIOK1	-8.024055111	5.142938151	-1.39149299	0.227866023	0.45800245	-6.491150484
HSD17B11	-8.043130926	19.05530337	-1.144138629	0.308815048	0.531148164	-6.772299045
CZIB	-8.055173428	20.22001931	-1.063854082	0.340235516	0.532427792	-6.857228803
HGS	-8.059814653	18.84403201	-1.163088472	0.301785279	0.531148164	-6.751746478
TRA2B	-8.062901602	19.01480771	-1.149245474	0.306906284	0.531148164	-6.766778523
ATL2	-8.077720348	18.86217345	-1.16572389	0.300819088	0.531148164	-6.748873602
PTGES2	-8.084472626	19.24959214	-1.136115534	0.311835221	0.531148164	-6.780944485
ARFGAP2	-8.097437027	19.06818419	-1.152909749	0.305543214	0.531148164	-6.76280909

SDHC	-8.104864566	19.3037293	-1.137171861	0.311436084	0.531148164	-6.779808157
NOP53	-8.106571687	11.65683003	-0.793423614	0.466622732	0.632093932	-7.113059496
OSTC	-8.109469256	20.78402958	-1.037286496	0.351229795	0.540235586	-6.884522149
BCAP31	-8.12880031	20.50870374	-1.057645598	0.342777785	0.534521366	-6.863644473
UBE2G2	-8.13705319	20.0213276	-1.091299542	0.329192307	0.531148164	-6.828599471
ZRANB2	-8.140019321	19.39195926	-1.135983415	0.311885175	0.531148164	-6.781086569
LTV1	-8.146166204	18.65284848	-1.195118923	0.290230034	0.529348194	-6.716595125
SPTLC1	-8.148808263	19.36433015	-1.139676612	0.310491471	0.531148164	-6.777111354
UMPS	-8.154345052	19.41551821	-1.13693396	0.311525936	0.531148164	-6.780064127
EIF4E	-8.164393117	20.082697	-1.091868664	0.328966661	0.531148164	-6.828001248
PITHD1	-8.186541468	19.94186663	-1.104640957	0.323938263	0.531148164	-6.8145281
PGAM5	-8.196163065	19.25036545	-1.157043946	0.304011853	0.531148164	-6.758322288
IPO11	-8.219807535	19.29397197	-1.157483137	0.303849577	0.531148164	-6.757845123
BCAS2	-8.234797663	19.09697371	-1.175697544	0.297187742	0.531148164	-6.737969613
FECH	-8.245550195	19.59145436	-1.14066399	0.310119805	0.531148164	-6.77604736
RAB33A	-8.254073174	4.127036587	-1.070009178	0.337731249	0.531148164	-6.850846007
ANAPC7	-8.314139277	19.67880547	-1.146130101	0.308069445	0.531148164	-6.770147869
COPG2	-8.326488336	18.91559309	-1.206742247	0.286137075	0.52545572	-6.703715346
CARHSP1	-8.329920824	19.98512111	-1.125528218	0.315860897	0.531148164	-6.792300903
KRT14	-8.34351121	19.6592879	-1.151847338	0.30593786	0.531148164	-6.763960692
TMEM109	-8.382818354	20.2046191	-1.119453811	0.318191373	0.531148164	-6.798789464
SCP2	-8.403720146	18.8709248	-1.225588794	0.279612449	0.518544617	-6.682695792
SZT2	-8.413694005	4.206847002	-1.070011361	0.337730364	0.531148164	-6.85084374
RTN3	-8.4267371	20.51642133	-1.106117709	0.323361253	0.531148164	-6.812964461
DAZAP1	-8.488409472	20.31991892	-1.129430616	0.314371723	0.531148164	-6.788121975
LYAR	-8.51359729	19.55644773	-1.185963283	0.293491438	0.531148164	-6.726694554
GNL1	-8.527238782	18.92769139	-1.239942192	0.27473521	0.51359514	-6.666577469
TMUB1	-8.536165183	19.54548103	-1.19549421	0.290097055	0.529348194	-6.716180284
ARL6IP1	-8.545738159	19.94364424	-1.166778025	0.300433403	0.531148164	-6.747723503
CPOX	-8.577028598	19.18535909	-1.231285632	0.27766723	0.516465703	-6.67630969
CD59	-8.591398721	19.12905216	-1.238647046	0.275172061	0.513683986	-6.668035706
BRCA1	-8.597170304	20.6140569	-1.127567536	0.315081904	0.531148164	-6.790118093
TFAM	-8.639262168	19.71020626	-1.200923549	0.288179415	0.527180644	-6.71017112
ACAA1	-8.670486484	19.09904334	-1.255198353	0.269637296	0.508648889	-6.64934383
RRP1	-8.684669598	18.8000685	-1.282580056	0.260706939	0.499144691	-6.618158624
SRSF11	-8.687472995	19.55059844	-1.217182701	0.282505606	0.521964979	-6.692091611
COA7	-8.692226063	18.99221113	-1.269167348	0.265046459	0.504039939	-6.633474599
PTP4A2	-8.776698946	18.83248513	-1.296529884	0.256264075	0.494478246	-6.602149409
ASAH1	-8.813932588	19.70308531	-1.232401263	0.27728775	0.516465703	-6.67505734
APOBEC3<br>C	-8.868799842	20.16103588	-1.206723103	0.286143772	0.52545572	-6.703736613
IMPDH1	-8.937183466	11.64339102	-0.886238983	0.419581056	0.59483408	-7.031093365
PEX19	-9.032377584	18.94285314	-1.339639082	0.242979757	0.478838438	-6.552181696
LZIC	-9.086593907	12.82951818	-0.994418308	0.369609925	0.552671445	-6.927650867

SNCG	-9.146833065	18.90057118	-1.36564641	0.235283657	0.46824613	-6.521692806
CNPY2	-9.172095712	19.45366813	-1.319891897	0.248982245	0.485067254	-6.575161287
YTHDF3	-9.179568298	19.44349572	-1.320063625	0.248929447	0.485067254	-6.5749621
CMBL	-9.188486993	20.21408217	-1.258811695	0.268442769	0.507821402	-6.645247083
SRP14	-9.219355779	20.37559307	-1.250032898	0.271353483	0.509419102	-6.655190384
CHTOP	-9.31360466	19.06181154	-1.383522943	0.230129092	0.460465694	-6.50059352
RPL37A	-9.411087077	20.95509719	-1.238428844	0.275245723	0.513683986	-6.66828131
CDK5	-9.502596096	19.83428633	-1.348189852	0.24042337	0.47474186	-6.542185134
TRIM4	-9.559105586	4.779552793	-1.07002395	0.337725259	0.531148164	-6.850830663
AKR1C3	-9.613446411	19.55248366	-1.393427201	0.227320042	0.457826152	-6.488855497
OPA1	-10.15646852	17.94891675	-1.645870755	0.16604098	0.368444934	-6.180016407
CDK9	-10.25489984	19.23811404	-1.552416969	0.186537676	0.400703875	-6.296260057
AKR1C2	-10.56464616	21.02854246	-1.435307589	0.215802914	0.441579383	-6.438857376
MAGEB1	-10.9396783	17.83522164	-1.714877877	0.152379767	0.349400043	-6.093075226
MYL4	-11.39735761	19.86422583	-1.721774691	0.151079017	0.34740346	-6.084342011
FRG1	-12.98254797	8.774180808	-1.743626963	0.147032327	0.342800903	-6.056623384
TMOD3	-13.42239646	16.27591075	-2.202590206	0.083799025	0.261533105	-5.46517846
KIF2A	-13.69228169	14.29425973	-1.966573465	0.111634508	0.280540174	-5.770699868
SPAG5	-13.8859881	14.9063201	-2.320787901	0.072764046	0.252008826	-5.312366497
PLP2	-14.14724443	15.1230495	-1.878318198	0.124443186	0.302452825	-5.884433454
CLPB	-14.17289402	14.12323879	-2.154876129	0.088757837	0.261829082	-5.526971072
UBL5	-14.18399073	14.60934664	-2.018698979	0.104730727	0.271706248	-5.703319744
PCID2	-14.31982752	14.36214031	-2.12740697	0.091755702	0.261829082	-5.562555422
BTK	-14.35430382	14.63228003	-2.060007159	0.099582703	0.265979027	-5.649854973
CFAP20	-14.44922284	14.70326695	-2.065560149	0.098911542	0.265979027	-5.642664653
PER3	-14.4685577	14.70461133	-2.072139454	0.098122595	0.265979027	-5.634144669
BCL2L13	-14.4864792	14.73836793	-2.060616709	0.099508792	0.265979027	-5.649065722
REPIN1	-14.53539132	14.42920818	-2.170940958	0.087053704	0.261829082	-5.506162638
GTPBP4	-14.55436414	14.83557217	-2.059804514	0.099607288	0.265979027	-5.650117357
HPCAL1	-14.58981214	14.65089684	-2.122177197	0.092338723	0.261829082	-5.56933042
CYFIP1	-14.60156874	14.77703194	-2.089477838	0.096075601	0.264921977	-5.611688848
FUNDC2	-14.65372241	14.71289247	-2.007428461	0.106184053	0.272931743	-5.717898037
IDH3B	-14.68332937	14.99633348	-2.051933034	0.100567284	0.267256051	-5.660308667
TMEM97	-14.70008417	14.76451991	-2.120913785	0.092480166	0.261829082	-5.570967125
PELO	-14.70360118	14.4485541	-2.21633024	0.082427315	0.260003361	-5.447391818
SLC25A11	-14.78485549	15.12444452	-2.04594566	0.101304116	0.268435569	-5.668059658
MRPS27	-14.79885988	14.86364728	-2.120147018	0.092566122	0.261829082	-5.571960443
OXA1L	-14.81096481	14.62024412	-2.196295968	0.084435649	0.261829082	-5.473327783
ALG5	-14.84181227	14.73131657	-2.165650141	0.087611009	0.261829082	-5.513015424
SMC3	-14.91214046	14.62951527	-2.22388216	0.081683775	0.259679509	-5.437617682
TRAV18	-14.92503342	15.29979184	-2.037643482	0.102335372	0.269373891	-5.67880581
PTRHD1	-14.95632166	15.0497873	-2.112305419	0.093450131	0.261884575	-5.582118848
ARPC4	-14.98385512	15.44540435	-2.015295318	0.105167383	0.271706248	-5.707722817
S100A13	-15.02850283	15.61828747	-1.98409885	0.109260952	0.277402806	-5.748058903

IDE	-15.03042672	14.89003432	-2.180156696	0.0860921	0.261829082	-5.49422706
ATP5MJ	-15.03803191	15.46433593	-2.025183281	0.10390419	0.27157055	-5.694930394
CLASP2	-15.03931326	14.42750948	-2.311947921	0.073531923	0.252581018	-5.323776218
MACF1	-15.09748058	14.69323618	-2.259591156	0.078265352	0.257646807	-5.391423141
AP2S1	-15.10853429	15.18707341	-2.117724571	0.092838248	0.261829082	-5.575098622
U2SURP	-15.10937991	15.30641893	-2.084970787	0.096603269	0.265300313	-5.617526544
NOB1	-15.11821071	15.20644799	-2.115213155	0.093121278	0.26186033	-5.578352042
SLC37A3	-15.13045074	7.56522537	-2.139841885	0.090385303	0.261829082	-5.546446498
CELF1	-15.16807749	14.87411417	-2.225415042	0.081533743	0.259574428	-5.435633917
SMU1	-15.22834844	15.02953003	-2.197064277	0.084357659	0.261829082	-5.47233299
TSFM	-15.23317846	16.32467531	-2.296076944	0.074932881	0.255087889	-5.344269958
CD2AP	-15.23678791	7.618393956	-2.139200943	0.090455396	0.261829082	-5.547276802
CHCHD5	-15.25832027	14.86246074	-2.256615425	0.078544187	0.257646807	-5.395271105
RPIA	-15.26369453	15.3664228	-2.111078939	0.093589215	0.261884575	-5.583707672
UBE3A	-15.2814529	14.72955144	-2.298779787	0.074692249	0.255087889	-5.340779053
NUDCD3	-15.31129476	14.6553543	-2.339432618	0.071173118	0.250687538	-5.28831461
EPS15	-15.31926504	15.04449936	-2.217727668	0.082289176	0.260003361	-5.445583077
TOMM6	-15.32292807	15.80140985	-2.012487856	0.10552901	0.27197314	-5.711354325
RBM17	-15.32817014	14.96153248	-2.247428538	0.079411873	0.257755316	-5.407152706
MANSC4	-15.33433648	15.55140235	-2.081035348	0.097066556	0.265586545	-5.622623661
COX6C	-15.33666546	15.48094947	-2.100459225	0.094802845	0.263614246	-5.597464468
SMAP2	-15.35308231	15.09304765	-2.214904071	0.082568554	0.260003361	-5.449237808
DCK	-15.39288516	15.24929674	-2.179524853	0.086157661	0.261829082	-5.495045341
UFC1	-15.39302479	14.93927022	-2.273894421	0.076940061	0.257071048	-5.372931812
MRI1	-15.42841817	15.20530705	-2.203470434	0.083710411	0.261533105	-5.464038868
ELOVL5	-15.45295778	15.32659238	-2.174979429	0.086630893	0.261829082	-5.500932152
COX17	-15.51934457	15.16552506	-2.241614744	0.079966364	0.25791477	-5.414673226
SUCLG1	-15.52486478	15.76199735	-2.076523697	0.097600608	0.265979027	-5.628466817
GATAD2A	-15.53769345	14.87354312	-2.339442783	0.071172261	0.250687538	-5.288301502
HDDC2	-15.54789397	14.75504871	-2.37900465	0.067921429	0.244072348	-5.237329115
ATP1B3	-15.56220238	15.81447646	-2.072000422	0.098139197	0.265979027	-5.634324718
JAGN1	-15.57924077	15.39643725	-2.187453764	0.08533885	0.261829082	-5.484777298
FLOT2	-15.61491674	14.99299081	-2.323947826	0.072491694	0.252008826	-5.308288919
CHP1	-15.63130061	15.28081189	-2.239590014	0.08016046	0.258165546	-5.417292592
ACOT13	-15.66888645	15.77111875	-2.111581761	0.093532167	0.261884575	-5.5830563
MFAP1	-15.68120711	15.18453594	-2.281470014	0.076248063	0.256131486	-5.363141194
RHEB	-15.79401856	15.63292155	-2.183877541	0.085707116	0.261829082	-5.48940843
MOB1B	-15.79677778	15.71217049	-2.163038302	0.087887546	0.261829082	-5.516398452
PQBP1	-15.83487487	15.89654355	-2.122905478	0.092257295	0.261829082	-5.568386958
THOC3	-15.89226933	15.48364028	-2.25574288	0.078626152	0.257646807	-5.396399465
EIF2B5	-15.96173748	15.33563324	-2.322009543	0.072658621	0.252008826	-5.310790028
DHX16	-15.99817041	15.57335626	-2.258435272	0.078373533	0.257646807	-5.392917797
HNRNPLL	-16.06626471	15.28770134	-2.367622934	0.068839641	0.245587079	-5.251984298
GATD3B	-16.14816229	16.50570961	-2.048538623	0.100984309	0.26804328	-5.664703028

DHCR24	-16.16078759	15.05119122	-2.479766988	0.060356386	0.230171395	-5.107965289
FIS1	-16.18177151	15.84338112	-2.233228835	0.080773601	0.259387296	-5.425522759
CNIH4	-16.1997413	16.08512922	-2.170139591	0.087137869	0.261829082	-5.507200566
AMPD2	-16.22583603	13.80462609	-3.041034978	0.03216224	0.159303594	-4.406052659
MRPL47	-16.26927442	15.47029392	-2.372716003	0.068427086	0.244902746	-5.245425462
GNG5	-16.44606421	16.75814949	-2.061751632	0.099371334	0.265979027	-5.647596197
CISD1	-16.45127595	15.8959232	-2.29421375	0.075099252	0.255087889	-5.346676584
CTSZ	-16.56036471	15.66874259	-2.396966354	0.066499662	0.240890331	-5.214217754
PPT1	-16.68396887	15.77493309	-2.40021963	0.066245668	0.240890331	-5.21003396
PRKAB1	-16.6986568	15.40104369	-2.532693395	0.056759846	0.22439954	-5.040328777
RPL39	-17.04705471	16.86004521	-2.187994505	0.085283316	0.261829082	-5.484077068
IGF2BP2	-18.1656488	15.9479533	-2.831104842	0.040464119	0.184736741	-4.664204534
THUMPD3	-19.76766801	12.11540663	-9.349680917	0.000373181	0.012546799	0.734939345
TLE3	-19.90852655	12.15942928	-9.624538781	0.000328593	0.012152462	0.87928881
OSBPL9	-20.47889156	12.51837992	-9.505858606	0.000347008	0.012459833	0.817483588
AAAS	-20.48697255	11.39058184	-18.83774899	1.62E-05	0.001201742	4.087640151
RBM28	-21.85076755	10.92538377	-100.5937942	7.74E-09	9.04E-07	8.207206888
ATP2B1	-22.08120527	11.04060263	-256.631561	1.06E-10	7.02E-08	8.517416939
PCDHGA3	-22.23070842	11.11535421	-258.3945689	1.03E-10	7.02E-08	8.518227414
OSBPL8	-22.51135204	11.25567602	-166.0288205	7.81E-10	1.65E-07	8.435848996
KIFBP	-22.57026828	11.28513414	-189.9635251	4.21E-10	1.11E-07	8.468683485
OGT	-22.57691876	12.40635228	-19.47967223	1.40E-05	0.001068017	4.234386165
HSDL2	-22.59766756	12.43445733	-21.11703704	9.68E-06	0.00076697	4.579786283
LRRC40	-22.67350349	11.33675174	-148.9671479	1.28E-09	2.37E-07	8.402570207
MICOS10	-23.59959877	11.79979938	-195.8647109	3.66E-10	1.11E-07	8.475032725
BRD4	-23.68258728	11.84129364	-131.7793585	2.25E-09	3.56E-07	8.355821006

Table S3. All the DEPs between K562/Adr and K562/Adr-sgPI4KA cells.

ID	logFC	AveExpr	t	P.Value	adj.P.Val	B
TMED7	24.77560433	12.38780217	179.0566837	5.51E-10	1.62E-07	7.494008249
SCAM2	23.16127967	11.58063983	229.1668197	1.78E-10	1.38E-07	7.524649726
PNKD	22.97872667	11.48936333	170.8108892	6.84E-10	1.62E-07	7.486283565
DHC24	22.49470567	12.98906428	13.76424728	6.69E-05	0.004073982	2.683975683
ABCB6	22.20147539	11.10073769	146.1235096	1.40E-09	2.98E-07	7.455046912
AT2B1	21.99227039	10.99613519	190.8031176	4.12E-10	1.61E-07	7.503359942
COX20	21.65941611	13.96697306	7.359465052	0.0010529	0.013144961	-0.290600477
PCYOX	21.30038144	12.37226939	12.85199582	9.09E-05	0.00461475	2.372152531
CD97	21.17093244	12.32356433	13.00745766	8.62E-05	0.00461475	2.427236266
GLPK3	21.13456467	12.357564	12.60409918	9.92E-05	0.00461475	2.282576695
EMC3	21.121026	12.34876967	12.58006249	0.000100079	0.00461475	2.27377519
ADA10	21.009453	12.19855006	13.23775849	7.97E-05	0.004590471	2.507334679
SERC1	20.46292622	13.36650733	6.980593323	0.001318884	0.014721787	-0.545949839
TGO1	20.29911872	11.84338292	12.76723991	9.37E-05	0.00461475	2.341769829
FLOT2	20.29899611	13.25148283	6.959743429	0.001335738	0.014832256	-0.560375911

SEP15	19.80140822	13.02986211	6.761847801	0.0015093	0.01600909	-0.699319708
MMAB	19.40451433	12.87268794	6.545725944	0.001730769	0.017123894	-0.855357594
MDN1	19.26724478	14.5730445	4.167562688	0.01056707	0.048449448	-2.931127365
ESYT2	19.20135456	12.72983528	6.528387633	0.001750172	0.017148718	-0.868075753
DREB	18.18774378	12.66700578	10.85836793	0.00019277	0.005884492	1.584634052
KAD4	18.13128328	12.41238869	5.791765144	0.00287939	0.021769004	-1.437724738
AT5G1	18.12126522	12.62742617	10.75061075	0.000201486	0.005884492	1.53728641
ALG5	17.56340872	13.73180036	3.792841385	0.01497336	0.061355478	-3.32990357
LAPM5	17.37431406	16.78656425	2.295306462	0.074995608	0.193104633	-5.131284976
NOP56	17.3589745	13.61609058	6.714619889	0.001554646	0.016327611	-0.733028442
ACO13	17.358796	16.52672133	2.366955423	0.068888034	0.183898903	-5.039213212
ITM2B	16.96731489	16.54423067	12.4632831	0.00010434	0.00461475	2.230717016
CHTOP	16.92328	14.75056056	5.7554481	0.002954785	0.022181697	-1.467372078
M2OM	16.82581067	16.03595833	2.361416616	0.06934018	0.184140033	-5.046320637
AT1B3	16.77809589	16.33465161	2.259073899	0.07830765	0.19671707	-5.177939156
PLP2	16.69399056	16.29785161	2.24643502	0.079500217	0.197776504	-5.194225375
FUND2	16.68867256	15.52991033	2.48464885	0.060008656	0.168118863	-4.888682656
NAKD2	16.68853556	14.65529856	5.653631427	0.00317907	0.022975513	-1.551319382
JAGN1	16.58597633	15.78305483	2.369243439	0.068702204	0.18378055	-5.036277761
TM9S3	16.58459189	15.66597094	2.402727212	0.066044816	0.178916833	-4.993357149
NFIP2	16.51031289	15.76267	2.34933413	0.070337851	0.185594429	-5.061831258
MIC10	16.50788344	8.253941722	2.139926705	0.090369732	0.204813629	-5.331634939
HPCL1	16.46086867	15.36251367	2.469514766	0.061076207	0.170214998	-4.907981106
MLEC	16.34126878	15.70345406	2.321022952	0.072737775	0.188887865	-5.098207182
DHRS7	16.30978911	15.52856956	2.365516099	0.069005217	0.183898903	-5.04105998
S10AD	16.308216	16.17520156	2.175347381	0.086586239	0.20144574	-5.285914049
CD82	16.24397333	15.70505356	2.291303867	0.075353864	0.193793049	-5.136436108
TMEM9	16.20433306	15.16385681	2.454956016	0.062123039	0.171562589	-4.926563243
SIM20	16.13745667	15.54811589	2.308328762	0.073842855	0.191059426	-5.114531066
RM13	16.12069044	19.25373333	1.968800575	0.111323347	0.232005254	-5.552384483
NDUB7	16.00689189	15.37270039	2.323659762	0.072510509	0.188671256	-5.09481744
RCN2	15.98127494	15.30910997	2.336076576	0.071450706	0.187080646	-5.07885995
STX4	15.94708767	15.45589317	2.280201191	0.076357437	0.194343198	-5.150728369
NENF	15.92054378	15.39881856	2.289956632	0.075474869	0.193794083	-5.138170092
GAL3B	15.91703389	16.29599661	2.038004472	0.102283853	0.217851324	-5.463190507
RM02	15.90982944	14.57775428	3.962824025	0.012751096	0.055593737	-3.146288338
RRAS2	15.87962033	15.49680761	2.247069452	0.079439885	0.197776504	-5.193407724
CHID1	15.83273094	15.23483797	2.31478883	0.073278202	0.18982883	-5.106222844
NDUAC	15.72981033	15.21211806	2.288650223	0.075592411	0.193794083	-5.139851605
CHP1	15.71132022	15.20758211	2.286623869	0.075775123	0.193794083	-5.142459928
MA1A1	15.69768033	14.91433017	2.376011602	0.068155718	0.183007546	-5.027596315
MYADM	15.684249	15.39396639	2.222267044	0.081836021	0.198444023	-5.225382447
SCAM4	15.68053761	15.26550108	2.259348426	0.078281964	0.19671707	-5.177585468
CN37	15.61228211	15.3635025	2.209493187	0.083100627	0.200136416	-5.241857172

68MP	15.55991367	15.63921683	2.117452291	0.092862555	0.206447306	-5.360649404
IDH3B	15.52846278	15.3297425	2.194545111	0.084607459	0.200136416	-5.26114111
MCAT	15.39922467	16.994184	2.426783729	0.064205514	0.175720354	-4.962567314
F262	15.38996578	7.694982889	2.139968166	0.0903652	0.204813629	-5.331581414
MCCA	15.29280628	14.91104197	2.252201282	0.078953695	0.197337958	-5.186794376
APMAP	15.25901578	7.629507889	2.139639466	0.090401133	0.204813629	-5.332005752
NFS1	15.24745483	14.94527975	2.224773711	0.081590323	0.198444023	-5.222150063
ACAD9	15.23583172	15.29701942	2.121259336	0.092435129	0.206277438	-5.355734442
AP3S1	15.23162267	15.331258	2.112271617	0.093447619	0.207531587	-5.367337725
T126A	15.20854939	15.00793119	2.197889601	0.084267766	0.200136416	-5.256826078
OXSM	15.12940622	14.73719444	2.256127117	0.078583948	0.196875414	-5.181735811
SUCA	15.08913667	15.45327522	2.035358043	0.102614771	0.218338016	-5.466604314
ACSA	15.08141289	16.05209711	2.177465051	0.086365526	0.201236394	-5.283181069
ARPC4	15.05313506	15.39168119	2.045005186	0.101413941	0.217301028	-5.454158973
LAP4B	14.99819594	15.05782164	2.118374772	0.092758791	0.206431881	-5.359458466
PYC	14.98613306	14.64025025	2.242080246	0.079915688	0.198186315	-5.199838134
SURF6	14.75971783	15.06523503	2.054613876	0.100232802	0.215637067	-5.441761106
IDE	14.5879835	14.53502869	2.15341589	0.088908237	0.203819743	-5.314221605
RCN3	14.56478456	15.39010072	3.593631239	0.018176486	0.069698325	-3.550891608
UBP48	14.51672528	12.23373119	4.249382532	0.009819142	0.046315068	-2.846952534
PAXX	14.49927417	7.249637083	2.138937744	0.090477896	0.204813629	-5.332911649
HMGCL	14.44265828	16.13849831	2.389710849	0.067064161	0.180988343	-5.010033305
PRAF3	14.43362844	16.708272	2.192186293	0.084847931	0.200136416	-5.264184577
IPP2	14.43019772	7.215098861	2.13947638	0.090418967	0.204813629	-5.332216289
AIFM2	14.38822067	7.194110333	2.139872247	0.090375685	0.204813629	-5.331705241
SCMC1	14.06306222	16.38101944	72.20891266	3.53E-08	5.37E-06	7.118005519
OSTM1	13.65980233	8.615315944	1.844027469	0.129821436	0.258430721	-5.712519896
SRS11	13.605273	17.85970094	2.104220403	0.094364738	0.208266688	-5.377731821
DHB12	13.550898	16.35628789	2.051464838	0.100618267	0.216030358	-5.445824452
BRI3B	13.41895978	8.454267111	1.845557151	0.12957611	0.25831135	-5.71056427
YKT6	13.22262389	8.403854722	1.832975594	0.131608353	0.259877656	-5.72664233
VATL	13.18775722	9.759087833	1.609472663	0.173733814	0.325197973	-6.008723404
DJC10	13.09826828	15.61693075	2.129873719	0.091475768	0.205723984	-5.344613173
P66A	13.07681567	8.314919722	1.831469702	0.131853809	0.259877656	-5.728565653
PCCB	13.06740383	8.319759694	1.830506709	0.132011023	0.259877656	-5.729795465
GCDH	13.00178467	8.277404222	1.826586517	0.132653034	0.260336495	-5.73480084
SMHD1	12.93730997	8.233959986	1.8306178	0.131992877	0.259877656	-5.729653599
DRS7B	12.89485672	8.141251917	1.841383956	0.130246538	0.259035093	-5.715898965
ERAP1	12.86774933	8.177623	1.833136958	0.13158208	0.259877656	-5.726436222
ANXA6	12.85873933	8.203879333	1.827497987	0.132503473	0.260336495	-5.733637201
NUD19	12.85582444	8.182757222	1.830872611	0.131951265	0.259877656	-5.729328193
SMRD2	12.80712389	13.14736483	2.769882217	0.043319216	0.13174974	-4.529086741
CSN7B	12.74026494	11.24561758	2.127973196	0.091686499	0.205785744	-5.347066758
LDAH	12.66482489	16.52568444	2.208026818	0.083247148	0.200136416	-5.243748649

LYAG	12.60399033	8.088052944	1.818955496	0.1339121	0.262167674	-5.744539629
NRBP	12.45945597	15.65383538	5.157503766	0.004611553	0.028832346	-1.978620043
NLTP	12.21780878	16.52794289	2.076950643	0.097543541	0.212423729	-5.41293386
RPR1B	12.14263978	16.57773311	2.014848818	0.105218333	0.222324565	-5.493053862
CPZIP	12.10009344	6.050046722	2.033753348	0.102815983	0.218548032	-5.468674229
PTSS2	12.09153594	16.33092081	2.097516015	0.095135818	0.209318435	-5.386386947
SCLY	11.90230422	5.951152111	2.042800666	0.101687021	0.217667398	-5.457003124
STIM1	11.8044165	16.78764069	2.281077915	0.076277662	0.194343198	-5.149599587
TYB4	11.66190489	9.0232685	1.543540994	0.188604832	0.346642673	-6.090186207
SUMF2	11.64954944	8.948298833	1.553930525	0.186179794	0.343369655	-6.077416369
NDRG3	11.49636483	16.21832036	1.927481547	0.117121096	0.240329333	-5.605534138
RAB32	11.42078072	17.04177386	6.916506892	0.001371519	0.015072573	-0.590419278
NOP58	11.41972189	8.889416167	1.534382411	0.190768555	0.348516331	-6.101420984
VAMP4	11.41943839	6.46022275	1.896065264	0.121741972	0.246489918	-5.64587361
GRIA2	11.15771039	20.56500803	1.593235657	0.17728349	0.329908494	-6.02887611
NOP2	11.01744517	9.054240139	1.572967802	0.18181656	0.337365453	-6.053950472
SUV3	10.99174906	17.68576025	1.826102437	0.132732537	0.260336495	-5.735418812
EMC2	10.84404294	17.44557492	1.845156542	0.129640312	0.25831135	-5.711076451
GNAS2	10.82808406	17.72179497	1.782195035	0.14015343	0.272634226	-5.791362042
RT34	10.39919911	17.16453744	2.354605476	0.069900663	0.185072737	-5.055063236
PTTG	10.30529911	21.54594656	1.345241066	0.241295898	0.411883791	-6.328019502
MIC27	10.23973656	10.0653215	1.356422385	0.237980105	0.407645248	-6.314950722
CD59	10.23048739	19.59337675	1.510710304	0.196475673	0.355590946	-6.130360236
TM109	10.18904833	20.93252661	1.375928363	0.232299357	0.401672529	-6.292043338
GNA1	10.06094239	9.988534639	1.1962465	0.289824948	0.459411139	-6.497331117
MET7A	9.977888778	20.10980183	1.412084666	0.222111419	0.388786163	-6.249231791
TP53B	9.826527278	9.819902528	1.187022718	0.29310668	0.460923885	-6.507487292
OPA1	9.754273222	9.853922167	1.325615196	0.24722217	0.419031949	-6.350845054
CNPY2	9.645523278	19.39108481	1.416289576	0.220954901	0.388356017	-6.244224463
TOM6	9.643635167	12.87318425	0.862646715	0.431167398	0.552101437	-6.833803434
DHB11	9.602932	19.52440767	1.394452748	0.227024789	0.395438602	-6.270164681
RHAG	9.512914056	19.32798575	1.396389417	0.226480036	0.394812295	-6.267870533
ATP5I	9.503055889	20.87226217	1.260930989	0.267738519	0.441810001	-6.424992779
GHITM	9.490482333	19.86587917	1.343168829	0.241915218	0.412280772	-6.330436454
DAD1	9.321089556	20.920985	1.226456678	0.279309488	0.452154768	-6.463784118
TMED5	9.320664444	19.30777067	1.360346174	0.236826829	0.407517998	-6.31035371
TMED2	9.297220222	20.302154	1.270756993	0.264522705	0.438199228	-6.413840907
OSTC	9.280332889	21.18339978	1.200199249	0.288428847	0.458219301	-6.492966258
MIC26	9.258926222	19.75306067	1.309814633	0.252093019	0.425207528	-6.369113776
LFG3	9.192575444	19.81994794	1.292222245	0.257622376	0.431461827	-6.389337378
MTX1	9.174274	20.01737	1.272371757	0.26399768	0.438108121	-6.412004309
MTX2	9.158398889	18.91412833	1.36616436	0.235126559	0.405246421	-6.303527013
NFIP1	9.113890444	19.96655278	1.265082765	0.266375337	0.439901022	-6.420285838
S29A1	9.0685265	18.99031942	1.340662454	0.242666303	0.412820675	-6.333357614

NDUA5	9.045241889	20.13205794	1.23979582	0.274778694	0.448732547	-6.44883745
BAX	9.022876	19.75681467	1.266091104	0.266045237	0.439901022	-6.419141548
GLPC	8.986852333	20.29396694	1.215244355	0.283170839	0.454544056	-6.476284731
HEMH	8.974154	19.73766711	1.259271995	0.268285035	0.442027585	-6.426871529
ABHGA	8.943650444	20.01431578	1.230884655	0.277797912	0.450734512	-6.458831461
MPU1	8.930188778	19.77799117	1.247439538	0.272213033	0.446086232	-6.440236546
ARI1A	8.916559	6.173505056	1.423176282	0.219073344	0.386322887	-6.236011176
PLOD3	8.884628778	19.07975817	1.296158064	0.256375544	0.430387921	-6.384823773
FACE1	8.828189556	20.38735167	1.182529992	0.294717321	0.460998859	-6.512419187
RT17	8.762669889	20.77824194	1.143429391	0.309075462	0.467680797	-6.554916098
LYAR	8.730186889	19.39764344	1.2410193	0.274366531	0.448580863	-6.44746251
BST2	8.714941778	19.71823589	1.214130667	0.283557033	0.454544056	-6.477523199
MMGT1	8.709683	12.15460517	0.820650902	0.452418017	0.572436328	-6.870883493
LAMP1	8.688208778	20.15604172	1.175872128	0.297118896	0.461761342	-6.519709627
IDH3G	8.651860778	19.47422133	1.220449883	0.281372093	0.453428044	-6.470488314
GNA13	8.627395556	11.52024933	0.861746609	0.431614559	0.55234228	-6.834612254
UFL1	8.588878833	19.21572969	1.23179218	0.277489041	0.450734512	-6.457815299
TBL1R	8.583938111	18.80409711	1.253726248	0.270119467	0.444363198	-6.433143203
TFAM	8.550316111	19.43696528	1.205893287	0.28642849	0.457074423	-6.486665464
MACD1	8.539210389	19.43854064	1.200691053	0.288255572	0.458219301	-6.492422655
RM50	8.527434556	19.16188583	1.224269989	0.280058739	0.452680237	-6.466226606
BAP31	8.487809667	20.50261172	1.116733578	0.319234522	0.472644445	-6.583476039
LRIF1	8.473243889	4.236621944	1.070037571	0.337714519	0.474639437	-6.632495318
ITB1	8.460075556	19.40538722	1.192726358	0.291073423	0.459802616	-6.50121195
SCAM1	8.449091889	19.62601372	1.173425265	0.298005943	0.462408058	-6.522383459
C560	8.436080556	19.22625672	1.202685388	0.287553888	0.457841667	-6.490217092
SPRE	8.420629667	18.98674639	1.21850022	0.282044561	0.454013832	-6.47266076
YIF1B	8.416212111	19.33393672	1.190690181	0.291797814	0.459802616	-6.503454033
SCAM3	8.396564333	20.18296472	1.124140058	0.316386696	0.470256967	-6.57559044
CATB	8.381353111	19.92749744	1.139605734	0.310512668	0.467680797	-6.559029879
ELOV1	8.376780111	19.30789917	1.185565372	0.293628262	0.460998859	-6.509088176
RDH11	8.348905	19.6595475	1.153315529	0.305387052	0.467602559	-6.544244641
RM30	8.348463667	18.99168961	1.205542558	0.286551337	0.457074423	-6.487054011
SGMR2	8.345228667	11.45737456	0.835614315	0.444755388	0.565425455	-6.857826961
RT09	8.31639	18.605912	1.231131447	0.277713887	0.450734512	-6.458555164
DIC	8.315521444	19.59404539	1.150944444	0.306268069	0.467680797	-6.546808677
SNUT1	8.305257111	17.57786817	2.260625476	0.078162596	0.19671707	-5.17594022
PGAM5	8.295816222	19.04395667	1.191372141	0.291555018	0.459802616	-6.502703334
SC5A3	8.262551	11.34410372	0.835814012	0.444653806	0.565425455	-6.857651539
FKB10	8.2581585	18.78620564	1.205300188	0.286636259	0.457074423	-6.487322482
NSUN6	8.181292833	11.48128392	0.815458337	0.455100597	0.574477032	-6.875373457
TMM70	8.169362	19.14587744	1.158018536	0.303646288	0.46674397	-6.539150391
REEP5	8.154628222	20.044917	1.08927313	0.329991545	0.474639437	-6.612450987
RTN3	8.152900556	20.19375028	1.082211378	0.332808713	0.474639437	-6.619834178

RT36	8.150821333	19.45409289	1.134138791	0.31257789	0.467680797	-6.564898319
S39AE	8.130049389	19.01447475	1.161218481	0.302466962	0.465602573	-6.535677795
HIBCH	8.093130778	18.72794094	1.179405043	0.295842335	0.461739281	-6.515843766
LTOR2	8.082391111	11.82451389	0.778201594	0.474703085	0.585192204	-6.906958833
TMUB1	8.067678556	19.05652072	1.148864887	0.307042636	0.467680797	-6.549055079
RT25	8.055802333	18.77847861	1.168461697	0.299812701	0.463810991	-6.527798278
VATD	8.019086667	19.13146189	1.134018811	0.312623351	0.467680797	-6.565026933
S43A3	8.018577444	19.3720585	1.114529888	0.320086218	0.472680539	-6.585816562
G45IP	8.002089222	19.09499594	1.134459864	0.312456262	0.467680797	-6.564554101
S38A2	7.967676111	21.34456894	1.412343591	0.222040036	0.388786163	-6.248923623
HEXB	7.966907667	19.14911294	1.123794369	0.316519112	0.470256967	-6.575959148
NUCB1	7.966880778	18.92682767	1.139608713	0.310511546	0.467680797	-6.559026677
ASAH1	7.869851889	19.02694494	1.114754741	0.319999224	0.472680539	-6.585577868
DCTN4	7.847323333	3.923661667	1.070023357	0.337720284	0.474639437	-6.632510051
CHMP6	7.797630778	19.03555983	1.102306544	0.324846885	0.474639437	-6.598750727
SYSM	7.786836278	18.69380908	1.125912184	0.315708652	0.470256967	-6.573699306
IF4E	7.779820389	19.70584653	1.046809618	0.347249135	0.478871382	-6.65641389
CISD1	7.778455278	11.47141075	0.770836471	0.478651851	0.586645509	-6.91306961
A16A1	7.771218056	11.06832819	0.802029777	0.462094204	0.579365403	-6.886886189
SPTC1	7.726009778	18.89463478	1.098278669	0.326429233	0.474639437	-6.6029948
AK1C2	7.715766278	19.41644258	1.057195109	0.34295773	0.477275378	-6.645758863
KDIS	7.713890722	11.17697886	0.786693159	0.470180517	0.583948926	-6.899858356
TMA7	7.672069556	11.05108006	0.791940355	0.467402035	0.582408614	-6.895441462
KS6A3	7.669107222	18.72723172	1.1015232	0.325154092	0.474639437	-6.599576822
STX12	7.661139222	6.8863535	1.132945618	0.313030252	0.467680797	-6.566177025
MARCS	7.621875722	18.05170042	1.144660401	0.308614025	0.467680797	-6.55359006
RL37A	7.620045667	19.87090028	1.013747222	0.361218969	0.486800785	-6.68990076
PREB	7.619897556	11.37445989	0.760797797	0.484073071	0.589609205	-6.921326497
AP1M1	7.608444278	18.77482525	1.087238353	0.330801139	0.474639437	-6.614581277
B2L13	7.595244167	11.13387764	0.776445358	0.475642476	0.585192204	-6.908420011
ALDH2	7.589578444	19.14275611	1.057545527	0.342813734	0.477275378	-6.645398237
MCES	7.589068778	18.427175	1.109225826	0.322144401	0.473010924	-6.591439128
NDUA8	7.587867556	18.89071461	1.073669132	0.336244527	0.474639437	-6.628727294
ILVBL	7.570842	3.785421	1.070015927	0.337723297	0.474639437	-6.632517752
SUCB1	7.568106333	19.46841383	1.029828272	0.354365462	0.482068706	-6.673696689
JIP4	7.566589556	19.14905111	1.053059166	0.344661238	0.478085724	-6.650009812
RNPS1	7.558563278	19.66473225	1.018090098	0.359357081	0.485519199	-6.685540434
FSIP2	7.556997444	3.778498722	1.070015534	0.337723457	0.474639437	-6.63251816
RSU1	7.556591778	11.37526589	0.753616702	0.487978698	0.591491075	-6.92718155
PPM1A	7.549757111	11.08648706	0.77491851	0.476460292	0.585483195	-6.909688284
CATL1	7.542068556	18.93542361	1.064041619	0.340153788	0.475655674	-6.638699853
MOB1B	7.508567	11.48221661	0.740301166	0.495281478	0.595644216	-6.937923586
RT23	7.454037167	11.33951808	0.744749508	0.492833054	0.59470201	-6.934351638
ACOC	7.451607833	18.42648131	1.084469498	0.331905597	0.474639437	-6.617476345

RNF14	7.448279833	10.95752769	0.773101155	0.47743507	0.58634307	-6.91119537
PDIP2	7.440698	18.96269711	1.043707084	0.348540074	0.479720748	-6.659584483
TRIR	7.437965111	19.17889	1.029410524	0.354542088	0.482068706	-6.674119648
NUDT3	7.432387222	19.25567361	1.023376963	0.357101497	0.484930185	-6.680216529
ULK1	7.426896556	11.37402917	0.739171714	0.495904542	0.595644216	-6.938827845
SNAPN	7.414611556	3.707305778	1.070011357	0.337725151	0.474639437	-6.63252249
CRBG3	7.411009167	3.705504583	1.070011248	0.337725195	0.474639437	-6.632522603
RBM28	7.376202111	3.688101056	1.070010188	0.337725625	0.474639437	-6.632523701
MP3B2	7.372104889	19.567282	0.993337265	0.370078875	0.494829776	-6.710233892
UBFD1	7.362389889	10.92705444	0.765732149	0.481402731	0.588503797	-6.917278393
NIPA	7.349399278	10.84593475	0.770594521	0.478781982	0.586645509	-6.913269597
MARH5	7.347788111	19.49063283	1.485960879	0.202622651	0.364210086	-6.16045676
PRKRA	7.342986556	10.92778606	0.763388074	0.482669927	0.589264265	-6.919203976
RANB3	7.337824667	18.621129	1.049918293	0.345959768	0.478384526	-6.653231253
YIPF3	7.337056722	11.16198481	0.744689018	0.492866289	0.59470201	-6.934400323
TF65	7.333635444	18.57670772	1.051741264	0.345205585	0.478384526	-6.651362234
EBP2	7.314569889	19.26059217	1.002997648	0.365862738	0.49146875	-6.700642732
DAZP1	7.313593778	19.544967	0.981872334	0.375135472	0.498621463	-6.721538971
LSM12	7.302217	19.00689572	1.01749593	0.359611332	0.485555009	-6.686137683
OSBL8	7.276763889	3.638381944	1.070007077	0.337726886	0.474639437	-6.632526926
AUP1	7.272078667	19.15017167	1.002530134	0.366065842	0.49146875	-6.701108263
SYDM	7.218983389	18.86719525	1.010079317	0.362797849	0.488619718	-6.693574232
RRP44	7.1999945	18.37992031	1.042402165	0.349084271	0.479850203	-6.660916302
NELFB	7.171235111	3.585617556	1.070003633	0.337728283	0.474639437	-6.632530496
ELP4	7.169015667	11.28285483	0.715988767	0.50881834	0.607051315	-6.957147157
COXM2	7.154557056	11.14812053	0.724937778	0.503805212	0.602756854	-6.950130399
AP2S1	7.131855333	11.106168	0.725294164	0.503606302	0.602756854	-6.94984953
BCL7C	7.125409278	3.562704639	1.070002089	0.337728909	0.474639437	-6.632532095
P4HA1	7.113506333	18.773792	2.171031352	0.087037967	0.201648054	-5.291484357
GOGA3	7.104324389	11.28397253	0.708858423	0.512837958	0.610821524	-6.962688136
BAIP2	7.076713222	18.99043178	1.014194265	0.361026934	0.486800785	-6.689452463
RL39	7.069502222	11.77860833	0.672326933	0.533781549	0.631532887	-6.990372802
CHM2B	7.06549	3.532745	1.070000026	0.337729746	0.474639437	-6.632534234
DEST	7.052506667	19.09718444	0.967626594	0.381498838	0.503625711	-6.73546682
ZN428	7.026651333	18.224989	1.021749597	0.357794508	0.485046498	-6.681857138
HYES	6.977470444	19.23314778	1.882299343	0.123826717	0.24976212	-5.663526439
FBRL	6.963935472	18.68158835	0.976848163	0.377369522	0.499642453	-6.726466221
NEUA	6.955128944	18.64763958	0.980004239	0.375964847	0.498790948	-6.723372952
RHEB	6.953816722	11.12532303	0.703725175	0.515745596	0.61259034	-6.966649655
YLPM1	6.926749833	3.642014639	1.042770594	0.348930549	0.479850203	-6.660540382
PTN2	6.920588444	5.239246111	0.833975855	0.445589524	0.565703373	-6.859265094
TPPC3	6.868578111	18.409066	0.981276463	0.375399853	0.498661986	-6.722124207
STK33	6.846964222	5.202434	0.831858969	0.44666901	0.566507037	-6.861120085
AL4A1	6.844055778	19.65337561	1.357598443	0.237633879	0.407590852	-6.31357347

HSDL2	6.794445556	4.150393111	0.958479896	0.385631613	0.506884464	-6.744338717
ECSIT	6.774232222	19.57497533	1.340174081	0.242812911	0.412820675	-6.333926534
CMBL	6.763076389	18.80069819	0.93839533	0.394836181	0.51485672	-6.763622375
CPPED	6.6804485	19.16224458	1.742168543	0.147292258	0.283673978	-5.842152553
ABCBA	6.611813722	4.047115528	0.957298649	0.38616803	0.507276796	-6.745480402
HEXI1	6.528732833	18.72424386	0.903533847	0.411238279	0.531691941	-6.796432444
COMT	6.518185889	20.70358428	1.147828834	0.307429185	0.467680797	-6.550173421
TXND3	6.111081222	4.8464425	0.807050798	0.459469774	0.578070994	-6.88259827
ZNT7	5.944258333	4.7158775	0.806834287	0.459582708	0.578070994	-6.882783584
THTM	5.921644833	18.72079397	0.80126192	0.462496551	0.579365403	-6.88754016
CGNL1	5.895250556	4.660575611	0.808864299	0.45852466	0.577762751	-6.88104462
H4	5.895092278	27.16051369	47.32489425	2.44E-07	2.89E-05	6.610271622
SPD2B	5.861445944	12.29290697	0.803608552	0.461267777	0.579365403	-6.885540093
IMA7	5.833968694	19.00294399	1.161612906	0.302321884	0.465602573	-6.535249403
RRAGC	5.752618556	4.621864056	0.79954219	0.463398629	0.580132634	-6.889003115
RM19	5.731081444	4.632972056	0.795998847	0.465261458	0.581440463	-6.89200992
SNR40	5.663847417	20.21269079	1.000420887	0.366983351	0.492390501	-6.703206838
SREK1	5.601823111	4.568342889	0.790954227	0.467923265	0.582717524	-6.896273247
MOGS	5.569742722	20.54573242	1.445270809	0.213141009	0.376954088	-6.209558932
ARL3	5.564415778	19.50548528	1.040113322	0.35004056	0.480235827	-6.663249856
AMRP	5.540725278	11.91415453	0.596254315	0.579236418	0.671523677	-7.044108955
GALT5	5.506912167	4.461589194	0.794722498	0.465933849	0.581598926	-6.89309053
ARMX3	5.506446833	4.508068417	0.788716066	0.469107891	0.583851735	-6.898158189
RASN	5.477621833	11.65318564	0.60614618	0.573187771	0.668408042	-7.03742928
HDHD5	5.433739444	12.17323828	0.567871136	0.596815632	0.686581112	-7.062750405
PRPF3	5.419908833	4.531082528	0.776715507	0.475497887	0.585192204	-6.908195413
SEC63	5.407026444	20.65633111	1.352285695	0.239201747	0.409292235	-6.319791076
HDDC2	5.373951778	4.425074	0.785397517	0.470868482	0.583997443	-6.900945543
PNPT1	5.365230111	20.44407483	1.39793678	0.226045692	0.394700586	-6.266036638
PPT1	5.347314611	9.98410275	0.56697325	0.597377094	0.686581112	-7.063327289
ARM10	5.328758778	11.70840778	0.582675	0.5876057	0.680116912	-7.05312525
WDR44	5.328585944	4.427852639	0.780176993	0.473648114	0.585192204	-6.905312295
ARV1	5.320148667	4.375299889	0.786112808	0.470488581	0.583948926	-6.900345503
MANF	5.276211389	20.48181875	1.337217668	0.243702211	0.414002481	-6.33736863
SERA	5.218219111	19.99490622	2.110024856	0.093702581	0.207799203	-5.370238308
TSNAX	5.081844556	21.07493383	1.208640339	0.285467962	0.456920191	-6.483620192
NDUA9	5.049374889	11.66606183	0.551114023	0.607347514	0.692441123	-7.073385599
GLSK	4.896239944	21.20325992	1.137196664	0.311421228	0.467680797	-6.56161781
GCP2	4.890993722	2.445496861	1.069866127	0.337784055	0.474639437	-6.632673016
CGL	4.760737722	20.37718569	1.208794813	0.285414036	0.456920191	-6.483448843
STRBP	4.710039056	21.24811369	1.077401835	0.334739398	0.474639437	-6.624846412
APOC3	4.557519111	25.99977922	38.65931075	6.14E-07	6.89E-05	6.232260885
ERG7	4.492114333	21.67126306	2.064575369	0.099023803	0.21389944	-5.428906083
KTN1	4.433038389	12.34549047	0.602702928	0.575288629	0.669208784	-7.039765035

TM214	4.394714389	19.88616186	1.151970416	0.305886572	0.467602559	-6.54569957
TF2H4	4.385220889	5.383338222	0.570811768	0.594979092	0.686044037	-7.060855531
BRAT1	4.207966333	5.245771833	0.563315158	0.599667915	0.688471726	-7.065669384
UBL4A	4.200105056	20.29798808	1.03211675	0.353399215	0.481993104	-6.671377776
THIK	4.199073611	12.71020281	0.456111304	0.669111177	0.745801114	-7.128305901
ZN548	4.183757667	5.267728056	0.558502943	0.602689687	0.690453742	-7.068730312
RAB9A	4.176505556	5.166804444	0.567040714	0.597334896	0.686581112	-7.063283971
AIF1L	4.163698278	5.274165194	0.555600416	0.604516781	0.691061543	-7.070565465
PELO	4.137347111	5.235126889	0.556119218	0.604189957	0.69105847	-7.07023806
CD63	4.115560333	24.72936183	4.714363183	0.006582236	0.036736457	-2.387644833
CPT2	4.071018444	8.669740778	0.553556673	0.605805306	0.69142233	-7.071852635
CLP1L	4.049681556	5.195271556	0.549519858	0.608355298	0.692849089	-7.07438287
SHC1	4.009081167	5.133698583	0.550399957	0.6077988	0.692585271	-7.073832614
SMCA5	3.963198611	5.156639861	0.542810257	0.612607956	0.695833864	-7.078552466
VKORL	3.959680111	5.137084833	0.544188046	0.611733237	0.695581472	-7.077699923
VPS51	3.919582556	5.083315389	0.544349959	0.611630492	0.695581472	-7.077599611
PEX19	3.882509667	8.315744333	0.60088312	0.576400954	0.669693097	-7.040994935
NSF	3.779655222	21.04188961	1.551926149	0.186645213	0.343930504	-6.079882006
IST1	3.734188556	13.98480483	0.392334002	0.712394647	0.785328536	-7.159853327
SPCS1	3.717124389	21.83636336	1.461385245	0.208913138	0.373346865	-6.190171432
NOMO3	3.711282111	21.94875517	1.484508338	0.202989233	0.364287075	-6.162217856
FAHD1	3.690578444	21.82923172	1.474129265	0.205627624	0.368092438	-6.174784269
H2A2C	3.687533556	28.25038711	18.38880633	1.81E-05	0.00167848	3.923298994
SCRB1	3.674522556	14.39837506	0.373788411	0.725230972	0.797417448	-7.168196884
NDUV1	3.472377611	21.77910831	1.417015361	0.220755871	0.388326334	-6.243359595
H2AZ	3.374056333	26.99925539	14.77367198	4.87E-05	0.003286451	2.999307886
MAGD2	3.055783889	1.527891944	1.069464887	0.337946841	0.474639437	-6.633088826
PTPRC	3.045215111	20.93467922	1.193346372	0.290853171	0.459802616	-6.50052884
ICT1	2.925189722	21.15698014	1.285116025	0.259887947	0.433553289	-6.397470697
ECHD1	2.8431555	21.16492869	1.217545322	0.282374457	0.454013832	-6.473724129
T106B	2.834091889	6.332700722	0.330449199	0.755632804	0.823624304	-7.186205639
GTR1	2.811922333	24.9138565	6.161281067	0.002229411	0.019517681	-1.144622362
RM01	2.632138222	20.83706744	1.151774307	0.30595946	0.467602559	-6.545911612
3HIDH	2.617372333	22.96351417	12.71791567	9.53E-05	0.00461475	2.323972666
ZRAB2	2.607977778	16.38929444	0.326100286	0.758713277	0.82655938	-7.187896395
RT35	2.578778444	6.221687111	0.344563686	0.745671619	0.814868891	-7.180571415
PPME1	2.542098333	21.17035906	1.049282957	0.346222948	0.478384526	-6.653882174
SYUG	2.530466167	6.194414972	0.340806061	0.748318008	0.817322742	-7.182093266
EMAL2	2.503854111	6.137517278	0.302448345	0.775556343	0.839451241	-7.196716314
HEBP1	2.501509667	13.48796194	0.273066337	0.796680094	0.858706754	-7.206782879
H2B1K	2.477361444	27.44491739	4.746938036	0.006407106	0.035947237	-2.356649367
JAM1	2.436926	23.50886756	6.139653612	0.002262258	0.019517681	-1.161359279
PTCD3	2.425642167	8.952631083	0.263379747	0.803689809	0.864950364	-7.209883693
S12A2	2.399120944	6.132447583	0.290503578	0.784118001	0.846124286	-7.200928238

ALBU	2.380562556	25.76151539	8.829056749	0.00047926	0.008504064	0.591538825
OXA1L	2.367700778	6.131340389	0.286886462	0.786717726	0.848397669	-7.202171447
SYIM	2.365805944	22.98456358	12.24843185	0.000112769	0.00471417	2.15018954
ARFG1	2.352453056	13.53502264	0.255230957	0.809603368	0.869997168	-7.212408077
ECI1	2.296829222	25.00613817	16.21799332	3.20E-05	0.002620348	3.403326515
LAMP2	2.281705	24.91165506	5.033859278	0.005080946	0.030774364	-2.090048923
VAS1	2.259889556	6.093242	0.312764937	0.768191337	0.834327015	-7.192947384
ATP5J	2.229432	25.62538	18.95288509	1.58E-05	0.0015303	4.043747658
BMP2K	2.204111056	6.02335375	0.272351938	0.79719632	0.858828982	-7.207015273
TM175	2.196462944	6.058176583	0.269927118	0.798949397	0.860282886	-7.207799669
NU188	2.160193611	11.33754864	0.258723757	0.807066815	0.867708749	-7.211335488
SMCA4	2.140235111	20.6853065	0.960537264	0.384698812	0.505970307	-6.742348022
TXD12	2.126339333	23.70358044	5.198223092	0.004468374	0.028437535	-1.942367307
COX7R	2.107211722	21.85138736	0.726573643	0.502892647	0.602341081	-6.948840268
TRPV2	2.048536111	24.50509517	5.556843543	0.003411353	0.023845915	-1.63227197
STOM	2.044616778	24.84253517	7.088491289	0.001235641	0.014317318	-0.471929261
MPCP	2.015286444	26.739277	9.254427177	0.000390111	0.007712787	0.818965734
PLD3	2.006356111	23.53698928	8.6496787	0.000524132	0.009011694	0.492168414
P66B	2.005264444	13.68655267	0.213862625	0.839843478	0.896169316	-7.224029518
KTAP2	1.984813111	24.63948689	7.711259664	0.000861782	0.011567564	-0.064479962
QCR2	1.980223444	26.18496194	16.00001781	3.40E-05	0.002620865	3.345600231
CATA	1.974768444	24.25130089	12.36171023	0.000108226	0.00461475	2.192860443
GRPE1	1.917532556	24.91692728	9.86686809	0.000294365	0.00689654	1.127484526
DHE3	1.893730361	25.33121104	3.761466972	0.015431336	0.062428098	-3.364291929
ERP29	1.881599444	25.58855061	9.692173624	0.000318451	0.007300408	1.041648777
SPNS1	1.861665444	24.02267783	10.13003724	0.000262102	0.006838858	1.25368229
KISHA	1.848119444	14.13900411	0.207848009	0.844268527	0.898642286	-7.225552127
SCPDL	1.843321278	22.88205203	9.251218977	0.000390704	0.007712787	0.817292208
SFXN1	1.824837556	25.653198	10.02229761	0.000274765	0.006838858	1.202461581
TM9S4	1.808548889	22.731866	5.182887994	0.004521667	0.028521285	-1.955994698
ODC	1.806463722	23.15195092	11.61708415	0.000142784	0.005222238	1.90328378
UCRI	1.796839222	24.49305406	7.705671338	0.000864473	0.011567564	-0.067993101
SPCS2	1.794058167	23.84568069	4.139673836	0.01083715	0.049474957	-2.960053771
CALU	1.791219222	25.9495135	15.953157	3.44E-05	0.002620865	3.333046258
FLOT1	1.786145778	23.06547178	12.36666897	0.000108032	0.00461475	2.194717425
SCOT1	1.786058	25.61907933	11.54635497	0.000146719	0.005222238	1.874625493
OAT	1.784918778	26.27691672	13.81211856	6.58E-05	0.004073982	2.699605845
MPPA	1.775138167	22.77353447	7.33346191	0.001068957	0.013144961	-0.307724324
NDUBB	1.771497	25.15351483	3.429203353	0.021430005	0.078773744	-3.738006887
ATP5L	1.765892667	26.04113011	5.383677714	0.003879517	0.025847282	-1.779976081
IDHP	1.74622	23.22803967	6.064675529	0.002380705	0.020141523	-1.219778001
LETM1	1.743282667	25.26379522	9.362181323	0.000370801	0.007712787	0.874817231
DHE4	1.732681028	25.25068638	3.446837571	0.02105058	0.077646778	-3.717736737
ECH1	1.717491889	24.60178639	6.94293343	0.001349515	0.014907591	-0.572035955

CMC2	1.711530667	24.52576189	8.883170104	0.000466644	0.008457539	0.6211037
IF2B3	1.704211944	23.59990381	3.833988872	0.014396516	0.060301319	-3.285038784
ATPK	1.699103556	26.03476533	4.950959033	0.00542754	0.032143097	-2.165911346
ANXA2	1.683735333	26.53338978	11.16472971	0.000170373	0.005635059	1.716340477
RM39	1.680125889	22.5937245	6.654427741	0.001614833	0.016550457	-0.776303672
TIM50	1.673207222	24.8405265	7.694363819	0.000869951	0.011567564	-0.075109265
AATM	1.656076222	25.55826789	8.313897055	0.000622583	0.009851962	0.300311969
DECR	1.651595333	25.547359	5.809354601	0.0028437	0.021708241	-1.423420969
ATPO	1.651386667	26.27205089	11.25697689	0.000164256	0.005558616	1.755177089
MDHM	1.648588667	28.13135067	10.01107062	0.000276127	0.006838858	1.197089021
TM14C	1.647117	25.73514794	7.011307565	0.00129452	0.014607995	-0.524771279
CH082	1.634984222	22.59862778	7.258609214	0.001116849	0.013302353	-0.357341462
S2540	1.634108	26.57316589	9.34567205	0.000373683	0.007712787	0.866304872
STML2	1.634036111	25.43335894	10.97873529	0.000183569	0.005841314	1.636887466
LAS1L	1.633658333	7.447484722	0.208626957	0.843695062	0.898628019	-7.225357336
THIM	1.632730889	24.03944867	6.554130444	0.001721456	0.017123894	-0.849203489
VDAC3	1.632689833	26.12617514	11.62805415	0.000142185	0.005222238	1.907710086
TOM22	1.630512778	25.46115694	10.07889641	0.000268023	0.006838858	1.229445149
TM192	1.629699556	23.64016744	9.153285305	0.000409352	0.007933977	0.765906413
P5CR1	1.620066444	25.58579378	8.350387843	0.000610862	0.009851962	0.321541494
NNTM	1.6127795	23.66419058	5.184188673	0.004517118	0.028521285	-1.954837665
SQSTM	1.601091889	25.07363094	8.297487381	0.000627942	0.009851962	0.290734356
COX41	1.600281333	26.19000067	7.639432175	0.000897164	0.011592405	-0.109825174
MYDGF	1.594517556	24.23660533	5.934984518	0.002603597	0.020625011	-1.322296293
ETFA	1.593245444	25.86080317	9.333584914	0.000375811	0.007712787	0.860062386
SPCS3	1.591104389	24.19705781	4.857094927	0.005854387	0.033825348	-2.252943837
VAPA	1.578215667	25.98033728	8.963129353	0.000448728	0.008392001	0.664446033
OST48	1.575642444	24.92242456	5.930365432	0.002611985	0.020625011	-1.325982445
EFGM	1.569760111	24.18637339	4.807995047	0.006093474	0.034829188	-2.298957557
ATPA	1.562735278	27.44848858	7.654072327	0.000889813	0.011567564	-0.100549128
RBMX	1.560944	24.57955856	12.4544738	0.00010467	0.00461475	2.227448778
CY1	1.557468222	24.769844	11.1055401	0.000174444	0.005635059	1.691223693
VKOR1	1.555624056	22.93890853	11.91707454	0.000127449	0.005140803	2.022564771
CK5P3	1.554607167	22.69995664	4.188587942	0.010368672	0.047848501	-2.909399013
AT5F1	1.553630889	26.20094544	3.325099491	0.023837557	0.084844192	-3.858655899
RPN1	1.553380556	25.44400306	11.67184344	0.000139824	0.005222238	1.925329384
COX5B	1.550385889	25.46995739	7.622633449	0.00090569	0.011592405	-0.120490223
GBRL2	1.545918111	23.78815072	3.819498837	0.014596645	0.060662861	-3.300807397
ODPB	1.544039333	24.03407967	3.923160843	0.013233072	0.05673143	-3.188726228
DHCR7	1.541919667	24.51493983	5.204631676	0.004446325	0.028437535	-1.936681504
AFG32	1.5377125	23.45057719	3.027129675	0.032643579	0.107070939	-4.21304828
CISY	1.528300889	26.57264956	6.000897837	0.002487337	0.020240467	-1.269958028
THIL	1.527680222	25.61442789	11.75486515	0.000135478	0.005222238	1.958519296
CD81	1.526708	24.52813578	7.347169799	0.001060456	0.013144961	-0.298690068

HACD3	1.522554444	23.711334	6.626595968	0.001643606	0.016607431	-0.796433159
K132L	1.520624111	24.58949461	4.385763285	0.008706199	0.042871021	-2.708909126
RRFM	1.517358389	22.28471558	2.693545071	0.047209148	0.138636231	-4.624475728
TM165	1.516165667	24.14630672	6.639485741	0.001630204	0.016550457	-0.787101093
SNG2	1.509342667	23.62073256	2.3762403	0.068137336	0.183007546	-5.027303016
ISOC2	1.505352556	25.51372006	10.00631121	0.000276706	0.006838858	1.194809436
ASPH	1.501703722	22.99997069	3.944868265	0.012966703	0.055961559	-3.165469674
CLPP	1.497369889	24.59500761	6.363775026	0.001948006	0.01805717	-0.990344311
COX5A	1.494005444	26.62966217	10.79706893	0.000197671	0.005884492	1.55776624
ATPB	1.490825222	28.47049594	8.308096608	0.000624471	0.009851962	0.296928692
ERO1A	1.483859722	23.13785725	7.883479254	0.000783565	0.011137064	0.042580516
TKFC	1.478403222	4.210894389	0.301522904	0.77621837	0.839623321	-7.197048471
PPIB	1.475446556	26.92059617	12.99371553	8.66E-05	0.00461475	2.422400408
S61A1	1.474066389	24.41346942	8.078150375	0.000705206	0.010440962	0.160849236
MGST3	1.473258667	25.650105	3.337639237	0.023531636	0.084536772	-3.844034518
DDRGK	1.465075222	23.04608817	10.09064983	0.000266648	0.006838858	1.235027523
H15	1.464997	24.66185461	2.127885147	0.091696274	0.205785744	-5.34718043
PDIA6	1.463657889	26.96195239	11.6460019	0.000141211	0.005222238	1.91494117
F162A	1.461901556	24.04560922	5.514037453	0.003520508	0.024056807	-1.668438318
ECHB	1.460413889	25.17817283	14.08461042	6.03E-05	0.003896713	2.78725645
TMED9	1.454171	24.68613917	10.43519315	0.000229885	0.006448881	1.395519046
AT1A1	1.453681222	24.98625661	13.54224709	7.19E-05	0.004260626	2.610565077
PEX14	1.448042278	22.62841619	6.387685466	0.001917666	0.01800208	-0.972411866
PDIA4	1.438516111	25.63301128	9.278061617	0.000385773	0.007712787	0.831275353
LAT1	1.431875111	24.45016022	7.330763006	0.00107064	0.013144961	-0.309504946
PHB	1.422508222	26.39572378	4.76956806	0.006288683	0.035563588	-2.335205218
NUCB2	1.422018722	22.85516708	5.497713404	0.003563228	0.024230851	-1.68228966
MCFD2	1.408153667	24.53414006	2.891981778	0.037825139	0.118173355	-4.377993945
VAT1	1.402984556	26.29033839	6.043717048	0.002415132	0.020240467	-1.236218238
NB5R3	1.402773444	23.91788017	7.589777883	0.00092265	0.011708863	-0.14141546
ERP44	1.401808111	25.60877961	7.173124141	0.001174738	0.013686019	-0.414602602
ETFB	1.39804	25.65644289	4.629240882	0.007067274	0.037690357	-2.469356482
NDUS2	1.397544944	23.01706364	4.501066304	0.007879734	0.040480944	-2.594376201
SNP23	1.397539556	14.49725556	0.171637972	0.871043613	0.919794444	-7.233815543
RM38	1.395990556	22.64544361	3.796729962	0.014917708	0.061280451	-3.325652267
GANAB	1.395634667	26.17203856	10.78137197	0.00019895	0.005884492	1.550857978
LMAN2	1.394806667	24.99235078	3.456136511	0.020853628	0.077187388	-3.707067351
ATP6	1.393329444	25.08757194	5.625964621	0.003243488	0.02299771	-1.574344132
MBOA7	1.392561889	25.0827495	7.830202076	0.000806812	0.011316599	0.009708413
FUMH	1.387966333	26.35537761	6.068157902	0.002375042	0.020141523	-1.217051062
VDAC2	1.385250222	27.119995	5.411435518	0.003799549	0.025473704	-1.756047983
ODP2	1.380756111	25.34472506	8.696578498	0.000511927	0.008873406	0.518354648
CUL4B	1.380485111	22.26031878	6.441315301	0.001851649	0.017623735	-0.932404943
GLU2B	1.376777444	26.54716206	12.65064314	9.76E-05	0.00461475	2.299560837

QCR7	1.373232444	24.52578633	5.902720648	0.002662869	0.020872193	-1.348094351
VDAC1	1.370612889	27.46353483	10.34112938	0.000239277	0.006619607	1.352299706
4F2	1.361019556	25.32430622	8.285720193	0.000631819	0.009851962	0.283854537
IDHC	1.354509778	23.70025456	7.757627607	0.000839832	0.011567564	-0.035426126
SSBP	1.351752556	25.70901317	4.086338505	0.011376543	0.051278623	-3.015707733
ILF3	1.347947556	25.42310733	2.453083046	0.062259147	0.171716044	-4.928955023
AGK	1.345369306	22.27657746	2.802323323	0.041776361	0.128154247	-4.488759487
NCLN	1.344931833	22.55058319	8.989738958	0.000442951	0.008357266	0.678779912
CALX	1.342076556	28.11322583	9.36572009	0.000370187	0.007712787	0.876639761
RTN4	1.341618444	26.44732778	8.702450322	0.000510423	0.008873406	0.521622843
RAB2A	1.338866111	24.90432006	6.191916011	0.002183855	0.019363099	-1.121001388
RM44	1.337846111	22.90677328	4.653709196	0.006923648	0.037690357	-2.445761885
MIC19	1.336024111	24.73294872	6.549109022	0.001727013	0.017123894	-0.852879521
MPRI	1.335125556	24.22836033	3.920923646	0.013260886	0.05673143	-3.191127283
K2C1	1.332639889	22.55908317	3.385884004	0.022396077	0.081324511	-3.788007059
FKBP2	1.319913889	24.48541083	5.870688608	0.002723317	0.021267807	-1.373824461
SSRG	1.311141667	25.71105617	9.51390412	0.000345533	0.007673706	0.952299444
ACON	1.308976222	23.85889111	4.892234452	0.005690086	0.033055215	-2.220219243
SDCB1	1.304996333	24.33984183	4.316048211	0.009255432	0.044559642	-2.779122836
COX2	1.303027333	25.77122	6.239367722	0.002115472	0.018950363	-1.084612983
SAC1	1.293580389	22.90270325	3.836920157	0.01435642	0.060251747	-3.281852873
RENBP	1.287764167	22.83758303	4.065032342	0.011600771	0.051959754	-3.038063288
TOM40	1.286672389	26.02764564	4.535397276	0.007651773	0.039500193	-2.560654664
ATPD	1.286032667	26.10305967	4.031150996	0.011968094	0.053158285	-3.073758669
PLCA	1.284090111	23.22631439	3.273886244	0.025135395	0.087994518	-3.918620859
MAOM	1.280919889	23.79679672	2.436400189	0.063486131	0.174423235	-4.950270841
MPPB	1.279606056	23.39085008	3.461502875	0.020740937	0.076903788	-3.700916259
SERPH	1.276316444	26.69532522	11.32669589	0.000159808	0.005554115	1.784283016
GLYM	1.269452	25.46820333	10.56087286	0.000218023	0.006197658	1.452583112
PSB3	1.266247444	24.2295335	1.980870862	0.109687571	0.228819864	-5.53684124
VA0D1	1.249259389	23.52951986	4.226471492	0.010022123	0.046849576	-2.870419747
GNAI2	1.247089278	23.04850181	4.580047927	0.007366826	0.038684907	-2.51705515
PGRC1	1.244076889	24.73452511	3.284759473	0.024853188	0.087658486	-3.905856029
EBP	1.237231667	23.66045028	4.351091008	0.008974414	0.043783639	-2.743737956
HCDH	1.235639111	24.86782289	3.707057441	0.0162648	0.064454559	-3.424296893
ACADM	1.229389556	23.67083189	6.841962338	0.001435953	0.015516992	-0.642626503
PRDX3	1.227934111	26.8129065	8.187664489	0.000665277	0.010204107	0.226138173
TM9S2	1.221219222	23.72276128	3.332973174	0.023644939	0.08458223	-3.849472336
USMG5	1.220723222	15.78922506	0.116825909	0.911944966	0.95614067	-7.243354517
FUS	1.220342778	25.69268783	5.575524127	0.003364984	0.023599167	-1.616559238
ADRO	1.218782	26.09992433	5.46820572	0.003642027	0.024572157	-1.707411266
RIDA	1.214385889	23.83106028	9.882658021	0.000292298	0.00689654	1.135160598
TGFB1	1.211024778	23.50808761	6.290036666	0.002045263	0.018555319	-1.046022256
NDUV2	1.206763778	23.53990122	6.226686688	0.002133493	0.019031826	-1.094313864

RNH2C	1.204346	6.962338111	0.157045886	0.881892994	0.930329472	-7.236705607
SC61B	1.196214222	24.869103	4.907589801	0.005620009	0.032737318	-2.205972891
LTOR5	1.194006778	23.62364961	4.131076966	0.010922031	0.049755919	-2.968994647
LSR	1.189222278	23.00211642	5.517371905	0.003511857	0.024056807	-1.665612998
ATD3B	1.188387444	24.748599	7.302372501	0.001088542	0.013144961	-0.328273684
SAM50	1.1797625	22.95338208	3.522597338	0.019506386	0.073606397	-3.63120583
QCR1	1.179104222	26.32903822	5.937916538	0.002598289	0.020625011	-1.31995772
TRXR2	1.177146556	23.99847606	1.489516806	0.201727973	0.363553709	-6.156142962
DLDH	1.175657111	25.80807444	4.308932753	0.00931375	0.044665224	-2.786330403
ODO1	1.170293	23.09881817	2.06850634	0.098551012	0.213527194	-5.423832802
QCR6	1.168551	24.10703828	2.750850747	0.044254141	0.133262469	-4.552803965
IR3IP	1.164932	24.59753911	5.186729518	0.004508246	0.028521285	-1.952578069
CH10	1.164559444	27.81967994	4.412940568	0.008502546	0.042423778	-2.681735098
AL1B1	1.158039222	23.78308672	5.643944539	0.003201449	0.022995256	-1.559370499
PHB2	1.154311667	26.47304528	4.93519828	0.005496567	0.032308727	-2.180440153
SDHA	1.151566778	24.72327283	6.309078418	0.002019601	0.018519974	-1.031589736
CPT1A	1.151554889	23.88771878	3.860758243	0.014035144	0.059136218	-3.255994368
UGGG1	1.145615778	23.43497233	7.018963567	0.001288531	0.014607995	-0.519505621
PDIA1	1.143389667	26.90967572	4.455253443	0.008196534	0.041607169	-2.639646603
RAP2B	1.130828056	22.64636286	7.689226508	0.000872454	0.011567564	-0.078345705
GRP75	1.121529444	28.23952939	9.464672146	0.000353497	0.007712787	0.927304334
TXND5	1.119036444	24.30355478	2.524756891	0.057278129	0.162605819	-4.837631489
NDUS1	1.111456111	23.19914283	7.305564343	0.001086512	0.013144961	-0.326160123
MDR1	1.111347444	27.81186883	9.699987199	0.000317324	0.007300408	1.045523992
MTCH2	1.109859778	24.92773	6.24992504	0.002100608	0.01889661	-1.076549799
ECHM	1.101188556	25.1751815	5.080011119	0.004899385	0.030015773	-2.04821789
STING	1.099700278	22.36551997	4.365813732	0.008859351	0.043321416	-2.728926698
CH60	1.098964278	29.34778992	9.991536466	0.000278516	0.006838858	1.18772523
SUCB2	1.096918889	24.06782711	5.987542353	0.002510384	0.020325424	-1.28052346
PRDX5	1.096487889	26.11246794	9.614553216	0.000329915	0.00740399	1.002966374
ANX11	1.093509889	25.32145139	7.942587194	0.00075871	0.011003882	0.078794315
RT31	1.090745167	22.76483764	3.486102295	0.020233287	0.075414978	-3.672777304
TOM70	1.086243556	24.55580822	5.848812409	0.002765543	0.021362816	-1.3914644
ACADV	1.085586222	24.56512644	5.864037686	0.002736073	0.02128944	-1.37918163
PDIA3	1.081214556	27.04919394	3.277198719	0.025049034	0.08798112	-3.91473021
SAP	1.078979556	24.94831311	5.944212789	0.002586935	0.020625011	-1.31493913
HBA	1.075439	24.52693217	3.708989241	0.016234325	0.064454559	-3.422158418
PGRC2	1.074381333	24.07672144	2.192761008	0.084789273	0.200136416	-5.26344304
MIC60	1.065152667	25.20889278	4.943267917	0.005461095	0.032252231	-2.172997027
ENSA	1.064377778	24.40784778	1.536199254	0.190337375	0.348516331	-6.099193943
CALR	1.064340333	28.53036261	6.271983235	0.002069954	0.018699751	-1.059741003
GAG2B	1.059807111	27.51845189	6.578999023	0.001694251	0.017038413	-0.831035068
TIM44	1.059770667	24.18345778	3.467975017	0.020605961	0.076536425	-3.69350368
AIFM1	1.058803111	25.128389	6.458435483	0.001831151	0.01750679	-0.919695693

VIME	1.049510889	26.83239544	8.447121151	0.000581056	0.009720668	0.377365222
RAB7A	1.03785	25.21372522	5.242968424	0.004317089	0.02801646	-1.902780417
P5CR2	1.037457111	25.05498833	7.94651983	0.000757091	0.011003882	0.081194252
ATPG	1.028066222	24.64556989	6.789129532	0.001483829	0.015897097	-0.679945104
TOIP1	1.019167333	23.15800578	5.679645847	0.003119887	0.022857726	-1.529753462
SATT	1.013232389	23.42567053	6.147277054	0.002250614	0.019517681	-1.155453906
ODPA	1.011269889	24.2042485	2.703010349	0.046706146	0.137622352	-4.612611551
AAAT	1.006275556	26.79732889	8.438616895	0.000583605	0.009720668	0.372483716
RB27B	0.995944833	23.26417825	4.977712554	0.005312707	0.031857178	-2.141326867
RPN2	0.983999444	25.66074739	3.587463642	0.018287697	0.06999887	-3.55783347
SSRA	0.979644111	24.79437828	3.287775488	0.024775556	0.087597821	-3.902318513
TXTP	0.978116556	24.4103745	5.237122012	0.004336506	0.02801646	-1.907938022
PSME1	0.970564222	24.12015867	6.142854081	0.002257361	0.019517681	-1.15887932
GLRX5	0.970408	25.57979689	3.531018706	0.01934297	0.073218309	-3.621642753
IFM1	0.966096667	28.02664333	7.214328692	0.001146395	0.013429204	-0.386922252
PLCE	0.965431111	22.59313144	5.694591146	0.003086481	0.022690954	-1.517400349
ABCBB	0.95831	27.80881889	2.623208188	0.0511429	0.148753974	-4.712947493
LMAN1	0.953213833	25.68056247	2.832143764	0.040412619	0.124542318	-4.451804961
SCYL1	0.944874389	22.87450947	2.385873816	0.067367944	0.181407981	-5.014951325
NSDHL	0.940773778	23.88272967	6.003521999	0.002482838	0.020240467	-1.267884416
PPIF	0.933920556	25.09785028	1.659100526	0.163322054	0.308964169	-5.946793799
TIM23	0.926973444	23.63164006	3.477255207	0.020414189	0.075956461	-3.682886465
ANXA7	0.925077333	24.136478	4.614679116	0.007154421	0.037943347	-2.483439341
NDUS8	0.924822889	22.893066	6.188558751	0.002188793	0.019363099	-1.123585055
ACSM3	0.922815778	24.26647856	5.398101874	0.003837715	0.025648926	-1.767529885
LBR	0.922609778	24.21605289	2.167427597	0.087417099	0.201921186	-5.296135606
H12	0.919751889	28.3185755	2.484984344	0.059985226	0.168118863	-4.888255059
SMD2	0.917570889	25.37118489	1.374083846	0.232830941	0.40226545	-6.294215307
ATD3A	0.914367222	24.94667989	3.725015933	0.015984044	0.063984465	-3.404439845
ECHA	0.913165833	26.03603336	3.029211727	0.032570325	0.107036849	-4.210526939
DEK	0.910262444	23.675896	6.147808513	0.002249805	0.019517681	-1.155042457
SNAA	0.907413667	23.7971065	2.713595551	0.046150739	0.136467926	-4.599355665
TMX1	0.905216111	23.94142217	3.298517846	0.024501305	0.086777581	-3.889729971
GELS	0.902731333	23.587561	2.3855981	0.067389832	0.181407981	-5.015304754
MPRD	0.897922222	24.46340867	3.649752648	0.017199784	0.06679406	-3.488000664
DCTP1	0.8964735	23.68113069	1.501513898	0.198738035	0.359380399	-6.141563057
COTL1	0.885196111	24.75021517	4.255791581	0.009763228	0.046153441	-2.840402234
LONM	0.880995667	26.10462517	3.529661634	0.019369196	0.073218309	-3.623183053
MCCB	0.872300889	23.25628222	5.660550785	0.003163198	0.022938567	-1.545575344
ERG1	0.868500667	23.36960856	5.759561562	0.002946128	0.022181697	-1.464006305
HCD2	0.866828111	26.5014195	4.820798732	0.006030031	0.034559209	-2.286926132
STT3A	0.865839556	24.65573656	3.797648255	0.014904601	0.061280451	-3.324648664
GALT2	0.862259361	23.09999688	1.554676172	0.186006949	0.343347892	-6.076498881
STX7	0.85843	22.78827222	4.426620411	0.008402162	0.042064414	-2.668098637

NTF2	0.853801667	24.5909125	1.74084811	0.147534041	0.283883192	-5.843824347
HYOU1	0.849671111	25.42589889	7.656378952	0.000888661	0.011567564	-0.099089218
LCAP	0.844876722	22.33700364	3.163036743	0.028232486	0.095999459	-4.049773555
GHC1	0.841216667	22.56925822	6.694175219	0.001574782	0.016458017	-0.747687611
GLOD4	0.837843611	23.44319792	3.164107111	0.02820056	0.095999459	-4.048498351
MINP1	0.809628056	22.58605847	1.902164858	0.120830041	0.245576403	-5.638047063
SFPQ	0.808351444	25.79885806	5.113573948	0.004772176	0.029577733	-2.017976403
OCAD1	0.801812778	23.32140028	3.819996262	0.014589721	0.060662861	-3.300265531
CLPT1	0.800019667	24.46340961	1.951748557	0.113678406	0.235075035	-5.574330514
TOM7	0.792279333	24.08868	2.971067214	0.034688374	0.111211447	-4.281167653
TMEDA	0.790147	24.16325983	2.178490413	0.086258876	0.201236394	-5.281857803
BCLF1	0.786616	23.00733189	4.185996997	0.010392883	0.047856643	-2.912072932
RT29	0.782843056	22.72360036	2.466724328	0.06127533	0.170547001	-4.911541397
CD44	0.778377056	22.27020181	4.099327424	0.011242338	0.051049304	-3.00211361
NDUS6	0.772220111	15.11210306	0.077302955	0.941643965	0.983628091	-7.247995267
RALA	0.770426556	23.29995339	4.97611289	0.005319491	0.031857178	-2.142794091
HMGA1	0.770214444	25.66914667	2.908353529	0.037150242	0.116993081	-4.357880664
COR1C	0.768182667	23.629765	2.663068732	0.048870438	0.142784505	-4.662743958
CISD2	0.768137917	24.06812404	2.497803461	0.059097509	0.16644107	-4.871923735
RAB5C	0.762653	24.26646406	7.010712951	0.001294986	0.014607995	-0.525180468
BROX	0.762288	22.64215967	3.787571665	0.015049165	0.061355478	-3.33566866
SLIRP	0.756356444	23.90504189	2.900204178	0.037484464	0.117352244	-4.367887994
VATH	0.751231	23.65482139	2.680255645	0.047925657	0.140353709	-4.641150226
RAB35	0.7486225	26.68138069	3.120694603	0.029529274	0.099143957	-4.100354275
CCD47	0.743254111	24.00176039	2.901455329	0.037432931	0.117352244	-4.366351019
ILEU	0.740709056	23.37320581	0.980184934	0.375884557	0.498790948	-6.723195656
TPP1	0.740485889	23.45735994	1.511879256	0.19618993	0.355432045	-6.128934626
NONO	0.737455556	25.523115	3.596728131	0.018120941	0.069610532	-3.547408203
TIM9	0.735737556	23.37739811	2.285245843	0.075899653	0.193794083	-5.144233825
AT2A2	0.733464111	24.74448483	3.392097042	0.02225448	0.081105215	-3.780817925
HMGB3	0.726350444	23.65721556	3.128730405	0.029278015	0.098610945	-4.090734675
MIA40	0.725983222	23.94296128	1.760028441	0.144061692	0.278964149	-5.81951621
RL1D1	0.725787556	24.21648744	2.197336572	0.084323834	0.200136416	-5.257539574
SARNP	0.722786333	23.28932039	3.200477303	0.027139897	0.093175928	-4.005269198
NUP54	0.718916	22.72293333	5.525296147	0.0034914	0.024056807	-1.658904184
HM13	0.716936667	25.00380478	3.747964743	0.015633436	0.063113575	-3.379139081
ISOC1	0.710929111	23.95038533	4.40931083	0.008529418	0.042423778	-2.685358014
NDUBA	0.701453111	23.71889689	3.692076579	0.016503398	0.06494956	-3.440900508
ACOX1	0.697814889	24.03098367	4.653743276	0.00692345	0.037690357	-2.445729083
PLIN2	0.696873611	22.25436297	1.665968577	0.161931755	0.307151693	-5.938186202
AL9A1	0.689223222	23.8568585	2.446078374	0.062771104	0.172697849	-4.937902377
RM10	0.683358444	7.458604111	0.069251981	0.947709142	0.986464229	-7.248709629
NCPR	0.682076333	22.83996906	1.195636371	0.290040991	0.459411139	-6.498004191
RM53	0.681884667	14.85404178	0.069449626	0.947560196	0.986464229	-7.24869303

BPNT1	0.681421	23.03441583	4.625584121	0.00708904	0.037690357	-2.472890094
LMNA	0.676466667	26.42203644	5.62158011	0.003253839	0.02299771	-1.578001446
SMD1	0.674523889	25.65650528	3.006303148	0.03338675	0.108506939	-4.238302568
TGM2	0.671384667	23.11956456	3.949662031	0.012908727	0.055824352	-3.160343765
MIRO2	0.664076333	23.46067206	5.812472416	0.002837429	0.021708241	-1.420889311
KAD3	0.661308778	24.15389183	2.057015468	0.09993989	0.21539283	-5.438662088
TWF2	0.652047611	15.57842608	0.063253917	0.952230468	0.989354463	-7.249190922
GNAI3	0.651584	23.94612489	2.181839216	0.085911554	0.201057555	-5.277536181
RB11B	0.649660111	24.51167017	3.572703003	0.018557072	0.070649423	-3.574471471
EFTU	0.642675722	27.23513947	4.968625349	0.005351385	0.031958413	-2.149666362
ADT2	0.642097889	27.87446683	3.233527213	0.026215559	0.090897577	-3.966158059
ATP5H	0.639681889	25.72394094	0.927523502	0.399893248	0.519544427	-6.773945602
CPNE3	0.633458111	24.52424183	3.043085404	0.032086952	0.1057332	-4.193741741
S35U4	0.631491111	23.17939911	6.021571463	0.002452158	0.020240467	-1.25364257
TM205	0.631070222	23.84684622	3.763958197	0.015394381	0.062396995	-3.361555706
RM11	0.628534167	22.68088214	1.511751408	0.196221162	0.355432045	-6.129090563
VAMP8	0.628303111	15.893339	0.059785703	0.954845765	0.991588491	-7.24944937
GTPBA	0.626467444	22.47415111	1.707003371	0.153872292	0.293956743	-5.886586614
NH2L1	0.625547556	23.70230367	1.640776558	0.167090871	0.315254635	-5.969716009
SC22B	0.617332444	23.87997611	5.619970218	0.003257649	0.02299771	-1.579344909
ECI2	0.615071556	23.03696778	2.178024796	0.086307288	0.201236394	-5.282458697
RM12	0.607181778	26.61781556	1.629158542	0.16952588	0.31900192	-5.984216047
UBXN4	0.603575278	23.26495914	1.591897402	0.177579281	0.330077617	-6.030534555
ACL6A	0.602487444	22.55638017	1.897691077	0.121498197	0.246230187	-5.643787761
DCXR	0.600713778	24.82078311	5.601976443	0.003300604	0.023224053	-1.594382111
LMBD1	0.599029667	8.185611833	0.055335884	0.958202228	0.993095842	-7.249759659
AN32A	0.597537056	26.75433208	0.910258813	0.408032059	0.528187219	-6.790169785
RM04	0.594274778	23.43838561	3.50420771	0.019868821	0.074500673	-3.652127318
RAB14	0.590100722	26.25055692	3.673889731	0.016798547	0.065431923	-3.461105048
CYB5B	0.5889105	24.92020769	2.36650041	0.068925055	0.183898903	-5.039797017
TFR1	0.585287111	27.47596022	5.334536664	0.004026059	0.026657013	-1.822576274
DJB11	0.585122556	23.6456845	1.621027666	0.171251297	0.32139768	-5.994347945
RM20	0.584924556	15.18526417	0.058280307	0.955981155	0.991597768	-7.249557023
GSTO1	0.582721667	26.57947661	2.729595386	0.045325245	0.135060623	-4.579343456
EMC1	0.58043	23.12154511	1.956762112	0.112980561	0.234313771	-5.56787963
RAB1B	0.580011	27.03776556	4.560768031	0.0074883	0.038755487	-2.535845422
RAB1A	0.580011	27.03776556	4.560768031	0.0074883	0.038755487	-2.535845422
ENPL	0.579411389	27.88688542	0.820881224	0.452299309	0.572436328	-6.870683845
ARRB1	0.577723778	15.08875589	0.057923867	0.956250005	0.991597768	-7.249582111
NDRG2	0.577306556	23.70381917	2.953824833	0.035346264	0.112474978	-4.302205748
RAB8A	0.575375889	26.56797578	2.722257858	0.04570174	0.135516146	-4.588517384
HNRL2	0.572485	23.55087094	2.396098425	0.066561785	0.179860236	-5.001848331
LRC59	0.572077444	26.40716872	1.413202117	0.221803507	0.388786163	-6.247901663
RBM25	0.569728278	22.78018303	1.17585714	0.297124322	0.461761342	-6.519726015

NPC2	0.568361222	24.3585815	1.431939996	0.21670138	0.382773274	-6.225537342
ARL1	0.564658278	22.78763097	2.469918746	0.061047439	0.170214998	-4.907465724
RAB10	0.563277222	26.48686883	2.763307257	0.043639675	0.13215879	-4.537275617
HXK1	0.558589333	23.12702167	3.334700855	0.023602914	0.084573801	-3.84745851
HTAI2	0.557419667	23.21710994	1.786890601	0.139339692	0.271936564	-5.785390116
QOR	0.556370111	24.16845839	2.741745255	0.044709463	0.133598768	-4.564166591
EMC4	0.555717556	7.671747556	0.054773788	0.958626286	0.993095842	-7.24979715
GSTK1	0.549274111	23.64984828	0.998756523	0.367708708	0.493053437	-6.704860786
RHG01	0.544769556	23.47072889	2.056372284	0.100018247	0.21539283	-5.439492066
RM49	0.544599556	23.12637133	1.485684614	0.202692323	0.364210086	-6.160791756
SYPL1	0.538476333	23.96570917	1.191472372	0.291519348	0.459802616	-6.502592981
ANXA5	0.536471556	25.19424967	4.648949497	0.006951314	0.037690357	-2.450344859
DYR	0.535709889	23.91720439	2.222221534	0.081840489	0.198444023	-5.225441136
SSA27	0.531244889	24.01889489	2.046198281	0.101266476	0.217203347	-5.452619661
ROA2	0.525818222	27.41507322	2.796528695	0.042047342	0.128440473	-4.495953262
DHSO	0.524999556	23.62841667	2.718617162	0.04588985	0.135884944	-4.593071571
SRP09	0.511938111	23.63956717	1.029503471	0.354502782	0.482068706	-6.67402555
LTOR1	0.511850889	23.49749111	2.108769189	0.0938454	0.207799203	-5.371859376
RPOM	0.507717	22.90616461	3.5459601	0.019056919	0.072294218	-3.604703152
RALB	0.502402611	23.00529458	2.422508334	0.06452823	0.176377162	-4.968036382
ADT3	0.494869889	27.70370428	2.396699662	0.066514712	0.179860236	-5.00107805
MAVS	0.482717	22.70796844	0.976817072	0.377383381	0.499642453	-6.72649666
F210B	0.471640667	7.439613778	0.047944529	0.963779654	0.996667789	-7.250222113
RER1	0.457619333	24.29372011	3.134373805	0.029103021	0.098176647	-4.083984657
LMNB2	0.454575222	23.06059206	1.363975082	0.235764968	0.406018508	-6.306097206
NDUB6	0.450690556	24.58347894	1.094293105	0.328001618	0.474639437	-6.607185406
KAP0	0.445586222	23.53879356	2.863587793	0.039028814	0.121230304	-4.41296055
LEUK	0.442511444	24.23431017	2.068905516	0.098503138	0.213527194	-5.423317614
LYRM7	0.440106778	23.73194028	1.311175107	0.251670093	0.425142468	-6.36754465
CSN8	0.439825222	23.40294283	2.584707995	0.053450418	0.154203372	-4.761594011
ENOA	0.435493833	30.13948581	3.892533027	0.013619853	0.057614141	-3.221665592
SSRD	0.428239444	24.88252272	1.535742364	0.190445715	0.348516331	-6.099754066
VAMP3	0.426869444	23.21271828	0.743513679	0.493512387	0.594781464	-6.935345671
STMN1	0.420053889	26.86796894	3.708062445	0.016248937	0.064454559	-3.423184294
PFKAP	0.419055556	24.63981567	1.398630681	0.225851175	0.394684184	-6.265213982
GTR3	0.414265722	24.36775319	1.359676247	0.237023355	0.407527253	-6.311138975
ARHG2	0.411390389	22.95459931	0.948163623	0.390337162	0.511492826	-6.754277778
LC7L3	0.409712889	23.36364378	2.18243096	0.085850338	0.201057555	-5.276772556
VATB2	0.405797111	24.99771144	3.597569766	0.018105879	0.069610532	-3.546461787
ANXA1	0.405784111	28.73859406	3.347325359	0.023298425	0.084047786	-3.832757035
SYAC	0.401694667	25.26984433	2.504670144	0.058627992	0.165337141	-4.863181422
BLMH	0.401268056	23.80682075	0.611775506	0.569763749	0.665290361	-7.033586228
NDUB1	0.398226667	23.48852689	0.928974906	0.399215084	0.518979609	-6.772572168
RAP1B	0.398089889	24.87324794	3.501752473	0.019917797	0.074500673	-3.654924606

TECR	0.395016333	23.20850306	0.97433822	0.378489744	0.500272867	-6.728921561
CYC	0.392930222	25.29974633	1.535068322	0.190605658	0.348516331	-6.100580309
VATA	0.390974	24.51879044	2.485067581	0.059979414	0.168118863	-4.888148972
BOLA3	0.390600056	7.596133639	0.038894966	0.970611582	0.998554062	-7.250698265
OCTC	0.387077444	23.79081161	2.190506479	0.085019633	0.200136416	-5.266352026
DDX18	0.384044944	15.09967564	0.038485833	0.97092053	0.998554062	-7.250717449
PTBP3	0.381864111	24.44087483	1.409099561	0.222935988	0.389909374	-6.252783023
ELAV1	0.381783333	23.55179556	1.629534259	0.169446576	0.31900192	-5.983747545
RT26	0.381406556	7.302753167	0.039504713	0.970151157	0.998554062	-7.250669299
SRPRB	0.380887667	23.4397195	1.826386786	0.132685831	0.260336495	-5.735055817
RS27A	0.376752667	27.165793	1.529102858	0.19202702	0.349502444	-6.10788779
TMX2	0.376535556	23.51935244	2.363729454	0.069150982	0.184057296	-5.043352567
ERH	0.375968222	25.09006133	0.840775407	0.442135777	0.563102436	-6.853283373
SMRC2	0.375537556	23.02149189	2.964228353	0.034947635	0.111399077	-4.289507133
GMPR1	0.371522444	23.99264222	1.328182662	0.246439133	0.418318655	-6.347867351
P5CS	0.37049	23.73968633	1.752716135	0.145375461	0.280997718	-5.828789451
RBBP7	0.367489556	24.63378844	1.556730282	0.185531621	0.343347892	-6.073970693
NDUS7	0.355264444	7.595137778	0.035382989	0.973263761	0.999266671	-7.25085634
NIT2	0.351426889	22.45623017	1.742789913	0.14717862	0.283673978	-5.841365753
DHX9	0.346556333	25.3064745	2.403576007	0.065978939	0.178916833	-4.992270095
FDFT	0.342500778	23.7282195	0.801228571	0.462514032	0.579365403	-6.887568553
DC1I2	0.336663	24.73207183	0.812970902	0.456389945	0.575753469	-6.877516799
PLOD1	0.336092722	7.347106194	0.034603889	0.973852178	0.999266671	-7.250889383
SC23B	0.335268833	22.84687481	1.187805118	0.292827006	0.460923885	-6.506627403
ARFP1	0.334423944	21.94646397	1.738876662	0.147895791	0.284322657	-5.846319922
BID	0.333830333	24.02210561	0.848803769	0.43808462	0.559279288	-6.846174993
SF3B3	0.332110111	24.50785817	0.943897418	0.392296862	0.513191779	-6.758366944
ADX	0.330153056	22.70383831	0.599287253	0.57737753	0.670180342	-7.042070874
UBQL2	0.328930778	23.81786172	1.343509766	0.24181322	0.412280772	-6.330038913
SMU1	0.326993389	7.404533639	0.033406537	0.974756519	0.999266671	-7.250938731
TCP4	0.323985	26.77488972	2.572871204	0.05418302	0.155895005	-4.776579917
RT18B	0.323694	23.35560278	2.27331574	0.076987137	0.195400686	-5.159594498
RM37	0.317416667	22.68739833	1.232450547	0.277265166	0.450734512	-6.457077887
NUCKS	0.314838667	23.21336911	1.091349807	0.329167068	0.474639437	-6.610274424
PP4R2	0.311679722	22.64579214	0.779104797	0.474220508	0.585192204	-6.90620639
ADAS	0.311655778	23.591958	0.886889733	0.419260532	0.539121504	-6.81179273
ILF2	0.30763	25.18527456	1.323959492	0.24772838	0.419505088	-6.352763962
GBB1	0.304376889	24.02048156	1.785148432	0.139641037	0.271997161	-5.787606163
IDH3A	0.301249556	23.961505	1.377633771	0.231808897	0.401149812	-6.290034108
ACSL3	0.300315667	23.89555206	1.112659032	0.320810854	0.472680539	-6.587801512
HS71A	0.299529611	29.74750725	2.202880528	0.083763593	0.200136416	-5.25038728
TOP1	0.295900111	24.47589672	1.919485958	0.118279346	0.241775232	-5.615807205
FAAA	0.292076444	24.18560467	2.038530872	0.102218167	0.217851324	-5.462511446
RHOG	0.291582444	24.58016411	0.776385485	0.475674526	0.585192204	-6.908469781

BAF	0.289278889	24.67189633	0.500028407	0.640136386	0.721719078	-7.104070848
PPIH	0.288874667	22.78434222	0.685261344	0.526299605	0.623372644	-6.980706645
AFAD	0.288128111	22.26543333	1.27209877	0.264086371	0.438108121	-6.412314876
VRK1	0.288043889	23.58796839	0.940761607	0.393742453	0.51377923	-6.761364716
RB27A	0.287817333	23.63577778	2.712308399	0.046217878	0.136477169	-4.600966887
RBP56	0.282691333	24.196326	0.97645663	0.377544086	0.499642453	-6.726849507
HEM6	0.281507944	14.73757581	0.028880114	0.978175638	0.999266671	-7.251109591
DHB4	0.278026444	24.64662289	1.032198531	0.353364726	0.481993104	-6.671294848
SBP1	0.276166222	23.64634022	1.174271486	0.297698898	0.462268062	-6.52145908
LAP2B	0.275927333	25.25725067	1.139728232	0.310466532	0.467680797	-6.558898206
LAP2A	0.275927333	25.25725067	1.139728232	0.310466532	0.467680797	-6.558898206
C1QBP	0.273121722	28.33374369	0.487673403	0.648216078	0.728901201	-7.111091952
CATD	0.272719444	24.54430083	0.84891829	0.438027042	0.559279288	-6.84607324
LC7L2	0.271519111	24.64359367	1.940458015	0.115266683	0.237209043	-5.588852772
LPPRC	0.267538111	25.98427906	1.4593795	0.209434886	0.373652869	-6.192588821
TKT	0.264872333	26.71991922	2.067015971	0.098729979	0.213697782	-5.42575629
FCL	0.261838222	24.30558767	1.09854644	0.32632383	0.474639437	-6.602712936
FAF2	0.260526667	23.45027067	0.758174017	0.485497416	0.590378451	-6.923470772
PHF6	0.260435556	22.68445722	0.731997522	0.499875417	0.599063737	-6.944546245
TSN14	0.257455333	7.417177667	0.026259952	0.980155082	0.999266671	-7.25119715
COPZ1	0.250409333	24.32882478	0.557087736	0.603580116	0.690731512	-7.069626133
LSM4	0.247407778	23.50625633	0.891267493	0.417138492	0.536716515	-6.807772063
RT07	0.244955444	15.02236489	0.024674645	0.981352809	0.999266671	-7.251246089
ISCU	0.244934944	7.320281194	0.025313823	0.980869892	0.999266671	-7.251226724
HBAZ	0.241498278	29.16545947	0.785986842	0.470555467	0.583948926	-6.900451202
PPM1F	0.239385778	22.97093289	0.481095097	0.652541172	0.731906624	-7.114765721
GMFG	0.238308556	23.09393417	0.696471034	0.519874301	0.616447169	-6.972208431
CX6B1	0.235724111	25.01397583	0.531695873	0.619691472	0.702756499	-7.085360172
AP3D1	0.234265333	21.9718035	1.009326978	0.36312242	0.488652809	-6.69432667
GRHPR	0.233360389	24.68074947	1.044152051	0.348354674	0.479720748	-6.659130109
AMPL	0.233136667	25.78015556	2.030427874	0.103234304	0.218782839	-5.472963579
IF2B	0.230116333	25.49458783	1.288299056	0.258870855	0.432872676	-6.393830182
RL35	0.230026667	25.87138089	0.453874677	0.670605498	0.746595782	-7.129485996
RBBP4	0.228453444	25.3139205	1.072555727	0.336694622	0.474639437	-6.629883347
OSBP1	0.226467667	22.97365572	1.779712948	0.140585557	0.27322553	-5.794517711
SF3B5	0.223904444	23.47418478	0.565602763	0.598234707	0.687196334	-7.064206287
AN32B	0.221066556	27.32979939	0.483224473	0.651139406	0.731031708	-7.113581461
FABP5	0.219964222	25.012405	0.576837798	0.591226554	0.68245534	-7.056946035
6PGL	0.218586444	24.43909933	0.601279932	0.576158293	0.669693097	-7.040727024
HEBP2	0.218507222	23.03539083	0.936009736	0.395941351	0.515899825	-6.765894574
EWS	0.215944444	24.31301122	1.129251215	0.314434588	0.469121443	-6.570131482
ESYT1	0.215286889	23.66288022	0.940704361	0.393768884	0.51377923	-6.76141938
ACTC	0.212110833	30.38602653	1.571923313	0.182053284	0.337510958	-6.055240138
GRB2	0.210983222	23.5129055	0.846796017	0.439095028	0.560233752	-6.847957288

DPP2	0.210918167	7.486299472	0.021315731	0.983890712	0.999266671	-7.251339715
HNRPC	0.209780111	24.47218294	0.770819667	0.478660887	0.586645509	-6.913083501
PDC6I	0.205681333	25.31633044	0.559991528	0.601753955	0.689752383	-7.067785904
GLYC	0.198249444	24.18204828	0.384290694	0.71794859	0.789817541	-7.163518468
TGT	0.198039722	14.54886858	0.020593962	0.98443609	0.999266671	-7.251358049
P4R3A	0.194342444	23.11372089	0.645302381	0.549647397	0.646358659	-7.010078906
SORCN	0.192769278	28.34253886	1.482717163	0.203442173	0.364792862	-6.164388702
RAC2	0.191940444	24.96174356	0.440703159	0.679441269	0.75446291	-7.136327842
MCM5	0.189823889	24.52472872	0.788067814	0.46945142	0.583938406	-6.898703378
CLAP1	0.187385833	7.287147472	0.01945533	0.985296477	0.999266671	-7.251385688
CCHL	0.186196694	15.9818391	0.017628611	0.986676851	0.999266671	-7.251426746
SERC	0.181857222	23.97147828	0.48968951	0.646893735	0.727798123	-7.109957022
VP26A	0.178501556	23.66432544	0.513228607	0.63156748	0.713945847	-7.096395799
ATLA3	0.176653111	23.74339978	0.596818532	0.578890321	0.671487576	-7.043730481
NNRE	0.175767667	24.69261606	1.251930092	0.270716088	0.445001311	-6.435171592
ICAL	0.175722556	24.5561785	1.357862351	0.237556251	0.407590852	-6.313264344
GT251	0.174381222	22.74376061	0.320907292	0.762398455	0.828879912	-7.189887295
TMM43	0.173716556	22.51986494	0.310675836	0.769680486	0.835093535	-7.193720376
STAT3	0.171969111	23.502566	0.541174741	0.613647272	0.696643228	-7.079562018
PER3	0.1703855	7.395638194	0.017431074	0.986826125	0.999266671	-7.251430944
LYPA1	0.170108944	24.50577219	0.706754542	0.514028264	0.611879486	-6.964314573
SNAG	0.168791333	22.93884489	1.242856752	0.273748607	0.447914067	-6.445396335
PTN1	0.167610556	23.35052883	0.990002969	0.371543534	0.496011782	-6.713530443
ROA3	0.16709	25.812665	1.295175762	0.256686198	0.430570396	-6.385950871
NQO2	0.164715333	23.57523156	0.413486659	0.69788787	0.771730777	-7.149876893
WASF2	0.163045667	10.32501717	0.019761334	0.985065248	0.999266671	-7.251378414
GGCT	0.161070667	23.11979856	1.113537352	0.320470475	0.472680539	-6.586869865
S10AG	0.160924278	7.205871417	0.016896733	0.987229916	0.999266671	-7.251442062
NAT10	0.158479944	22.71916681	1.073454535	0.336331238	0.474639437	-6.628950167
CPSF6	0.158411333	23.83825156	0.953910576	0.387710008	0.508988754	-6.748749808
CSN3	0.157988667	22.77730811	0.460618874	0.666105009	0.743137561	-7.125911529
GPKOW	0.155860889	14.53367039	0.016224879	0.987737629	0.999266671	-7.25145555
TFG	0.147181111	23.35662167	0.479274305	0.65374112	0.732794988	-7.115774612
RT16	0.146538722	23.27057292	0.706003711	0.51445353	0.611879486	-6.964894076
TERA	0.143633667	26.3941235	1.096386673	0.327174837	0.474639437	-6.604985237
SRSF3	0.143385	26.16756306	0.656904305	0.542797527	0.640422981	-7.001700704
FN3K	0.141648222	23.72939322	0.503959063	0.637577897	0.719595594	-7.101804118
GRSF1	0.141619	23.25934706	0.334820969	0.752541446	0.821094352	-7.184484484
PLIN3	0.140627	25.51649283	1.081487804	0.333098554	0.474639437	-6.620589089
ACTZ	0.140170222	24.59200511	0.568262903	0.596570756	0.686581112	-7.062498449
PLSL	0.134331778	25.54771011	0.96089609	0.384536313	0.505970307	-6.742000534
TRA2A	0.130151444	15.5594185	0.012658325	0.990432944	0.999266671	-7.251517989
GORS2	0.129659778	23.800183	0.893700198	0.415962992	0.535850814	-6.805531779
CATC	0.127027111	23.64409056	0.646775337	0.548774557	0.646044923	-7.009022101

LIS1	0.126436889	23.77289917	1.076908787	0.334937872	0.474639437	-6.625359491
SODC	0.125333333	27.44383944	0.96359255	0.383317004	0.505087672	-6.739386533
RM21	0.120846889	14.98584267	0.012201682	0.99077805	0.999266671	-7.25152487
PNCB	0.118716	23.62572511	0.633149182	0.556884413	0.65342739	-7.018721711
RM22	0.117642	15.29592511	0.011638495	0.991203679	0.999266671	-7.251533008
DNPEP	0.117397667	22.75917844	0.351917837	0.740504096	0.810449041	-7.17754716
EI2BE	0.116659333	7.183738944	0.012287096	0.990713499	0.999266671	-7.251523602
DHPR	0.113800222	23.30770578	0.290371665	0.784212753	0.846124286	-7.200973841
LYRIC	0.112183333	24.12428178	0.623254345	0.562822907	0.659076058	-7.025656656
MRP	0.111759667	24.89422372	0.822708486	0.451358381	0.571774253	-6.869098465
ROA1	0.104976667	26.32335889	0.873991594	0.42556259	0.54623687	-6.82355729
USO1	0.101909	23.85468517	0.841234323	0.441903425	0.563102436	-6.852878373
CCAR1	0.100815	22.54550361	0.529220319	0.621275796	0.704178626	-7.086859557
PO210	0.099571889	23.2846595	0.339786707	0.749036607	0.817688707	-7.182503374
RRBP1	0.099353889	22.75305806	0.360190899	0.734709829	0.805347741	-7.174072774
SYYC	0.097608556	25.75023594	0.935624446	0.396120081	0.515899825	-6.766261184
SCFD1	0.096674028	22.06295949	0.464478574	0.663536683	0.740659795	-7.123844256
G6PD	0.091053444	25.84071739	0.560913593	0.601174786	0.689459196	-7.067199817
HDAC2	0.090967333	23.70212644	0.242710281	0.818718275	0.876258716	-7.21613644
RAP1A	0.088846444	24.43970022	0.437103071	0.681866814	0.755989401	-7.138165707
KHDR1	0.086796	25.21973122	0.249524139	0.813753627	0.873136755	-7.214130063
FUBP3	0.085594611	23.37345442	0.400320457	0.706900363	0.78048243	-7.156143934
PSB2	0.083853111	24.28743644	0.361172604	0.734023614	0.805347741	-7.173655429
SNX9	0.083007333	23.72856033	0.537490266	0.61599249	0.698933469	-7.081826507
RU17	0.082140556	24.15669017	0.219293	0.835854153	0.893253661	-7.222618251
DYL2	0.082014333	23.34138017	0.414151176	0.697434508	0.771629669	-7.149555595
NDK3	0.081142278	7.165980417	0.008567599	0.993524567	0.999364661	-7.25157062
PEBP1	0.080658778	26.30177994	0.665028687	0.538035261	0.63515569	-6.995760322
PRDX4	0.080106778	25.9414405	0.431088135	0.685929308	0.760083828	-7.141205414
CAP1	0.079843222	25.93351483	0.513378284	0.631470697	0.713945847	-7.096307749
TBA1C	0.078079472	30.2164949	0.739958021	0.495470714	0.595644216	-6.938198429
TBA1A	0.078079472	30.2164949	0.739958021	0.495470714	0.595644216	-6.938198429
LMNB1	0.077618111	25.71429661	0.208438726	0.843833628	0.898628019	-7.225404472
BIP	0.07566	28.85957911	0.69168812	0.522609113	0.619345541	-6.975848083
DYHC1	0.074862	25.81821644	0.607778139	0.572193775	0.667715998	-7.036318274
NEK9	0.070897778	4.924496222	0.010892483	0.991767483	0.999266671	-7.251543196
COPE	0.067811889	23.34467361	0.136638487	0.897116124	0.943057369	-7.240321299
PPIL1	0.067569444	22.60536494	0.096385164	0.927287639	0.97005753	-7.245989619
PTH2	0.064400667	23.23318944	0.212135267	0.841113628	0.897075665	-7.22447116
IF2A	0.062243667	25.33226461	0.355022354	0.738327391	0.808481766	-7.176252327
FKBP3	0.061079222	24.13397272	0.245126552	0.816956611	0.875692053	-7.215431147
HSP72	0.059539222	29.35027339	0.577234676	0.590979927	0.68245534	-7.056687307
ROA0	0.058710222	25.13965122	0.068941349	0.947943242	0.986464229	-7.248735622
SPS1	0.058671944	22.08337769	0.184590447	0.861440731	0.912365444	-7.231038084

TM10C	0.057775444	23.28501683	0.390803318	0.713450013	0.786085493	-7.160556295
GSHR	0.056619222	24.85042017	0.429641266	0.686908373	0.760773325	-7.141930814
THG1	0.052993667	7.398735444	0.005419506	0.995903874	1	-7.25159731
VAPB	0.050843444	25.06329194	0.136128956	0.897496905	0.943057369	-7.240405202
CD2B2	0.047643444	14.64633939	0.00492254	0.996279483	1	-7.251600425
EXOS1	0.046914556	22.28523406	0.215211313	0.838852162	0.89574152	-7.223682252
SRP54	0.044065667	22.34114267	0.182959408	0.862648486	0.913190949	-7.231398793
BCAT1	0.042869333	23.25406578	0.150411348	0.886835986	0.933695962	-7.23793568
SRRM1	0.040158889	23.186044	0.068704397	0.948121819	0.986464229	-7.248755372
CSTF2	0.035345056	22.52264753	0.110599829	0.91661386	0.959833374	-7.244210382
CSTF1	0.034097944	7.360058694	0.003505432	0.997350545	1	-7.251607665
RINI	0.030293333	25.64131633	0.233034846	0.825784822	0.883378445	-7.218892362
HNRPD	0.029157667	26.88449894	0.227670676	0.829710919	0.88713324	-7.22037311
PSD10	0.027647167	23.64120681	0.035125142	0.973458498	0.999266671	-7.250867357
FUBP1	0.023480333	24.68720672	0.152022192	0.885635284	0.933353646	-7.237641852
RM14	0.020908111	22.45840111	0.150437028	0.886816842	0.933695962	-7.23793102
6PGD	0.017099889	27.1649875	0.116498363	0.912190489	0.95614067	-7.243400704
PRDX6	0.016468333	28.09731861	0.127727766	0.903779799	0.949191395	-7.241743752
LEG1	0.013191333	26.20062489	0.071545277	0.945981055	0.986464229	-7.248514121
RCC2	0.013059333	25.28563333	0.026092299	0.980281744	0.999266671	-7.25120247
HSP7C	0.013016222	29.40292478	0.126385709	0.904784238	0.949778432	-7.241949742
PSB4	0.012254333	24.96138694	0.070090067	0.947077573	0.986464229	-7.248638917
PCNP	0.012083889	23.0398295	0.068171694	0.948523297	0.986464229	-7.248799524
IF1AX	0.007875778	24.49122189	0.05015534	0.962111134	0.995738319	-7.25009072
SURF4	0.003328667	24.01496522	0.014356117	0.989149869	0.999266671	-7.251490189
TMM33	0.001348778	23.60122161	0.004079842	0.9969164	1	-7.251605024
KINH	0.001076556	23.39202861	0.006660866	0.994965655	1	-7.25158822
ISLR2	0	0	0	1	1	-7.251615113
EXOC5	0	0	0	1	1	-7.251615113
AHDC1	0	0	0	1	1	-7.251615113
ACTB	0	30.84931	0	1	1	-7.251615113
RM46	-0.000844722	15.10720386	-8.46E-05	0.999936063	1	-7.251615109
CLH1	-0.006495778	26.28159478	-0.046487857	0.964879136	0.996667789	-7.250305453
SYFB	-0.006892889	24.03813078	-0.030304227	0.977099845	0.999266671	-7.251058511
HSP76	-0.007809333	29.41901644	-0.076099437	0.942550352	0.984092728	-7.248107032
THOP1	-0.0143535	22.73910019	-0.041499388	0.968645055	0.998554062	-7.250571394
PIMT	-0.017325333	25.78197256	-0.054035902	0.959182988	0.993189961	-7.249845786
GBB2	-0.019359278	23.99776875	-0.114955928	0.913346828	0.956882279	-7.243616468
STAM1	-0.025070889	7.268828222	-0.002609756	0.998027507	1	-7.251610985
SRP19	-0.026217111	23.299215	-0.140772045	0.894028241	0.940339522	-7.23962915
GARS	-0.031614333	25.08568394	-0.14450575	0.891240932	0.937870517	-7.238986379
G3P	-0.032985389	30.34074003	-0.302295669	0.775665547	0.839451241	-7.196771179
KPCA	-0.036012333	23.88606594	-0.244190186	0.817639152	0.875983252	-7.215705274
PELP1	-0.041099778	14.55475967	-0.004272096	0.996771093	1	-7.25160405

SEC13	-0.041386111	24.17537361	-0.193538906	0.854822485	0.907158556	-7.229003027
UBQL1	-0.042507333	24.22804022	-0.199544755	0.850388304	0.904302699	-7.227584048
HNRPL	-0.044748	25.69104178	-0.108401485	0.918263327	0.961088569	-7.244501443
HDGR2	-0.046439111	23.18503489	-0.086515138	0.934709655	0.976863228	-7.247081856
ATRAP	-0.048575222	23.10318872	-0.385654203	0.717005643	0.789187419	-7.162902154
RFC4	-0.052644167	22.50128375	-0.15344878	0.884572222	0.932694351	-7.237379047
UBE2K	-0.053026667	24.87346667	-0.397193574	0.709049062	0.782449586	-7.157604557
NUP93	-0.056829778	24.04007811	-0.311559489	0.76905046	0.834834818	-7.19339402
SEPT7	-0.058313333	24.09301922	-0.389503744	0.714346619	0.786666835	-7.161151108
SRSF2	-0.060804333	25.73660128	-0.259446344	0.806542398	0.867582438	-7.211111824
COX1	-0.061578333	26.77609361	-0.16233405	0.877957557	0.926636392	-7.235687551
LA	-0.064687667	26.29790328	-0.474996813	0.656564871	0.73480121	-7.118131134
PNPO	-0.065047056	15.30233242	-0.008282924	0.99373972	0.999364661	-7.251573527
VPS28	-0.069002222	22.19513194	-0.437083895	0.681879746	0.755989401	-7.13817546
PLSI	-0.070363111	24.05728811	-0.177698162	0.866547292	0.916408148	-7.232540825
UBR4	-0.072983778	22.96319678	-0.442020084	0.678555118	0.754431048	-7.135652086
DC1L1	-0.074124222	23.373249	-0.475685413	0.656109846	0.734677621	-7.117753068
IF2B1	-0.075360778	24.17863083	-0.444244703	0.677059553	0.75338777	-7.134506362
ERLN2	-0.077630778	22.75875144	-0.122886636	0.907404027	0.952059737	-7.242476646
UB2V2	-0.078469444	24.2645215	-0.344538815	0.745689121	0.814868891	-7.18058154
GDIB	-0.079115333	25.334512	-0.639929561	0.55283906	0.649753515	-7.013916737
RD23A	-0.080701667	23.11471239	-0.56773885	0.596898331	0.686581112	-7.062835446
TLN1	-0.081128222	25.29625844	-0.366089403	0.730591098	0.802070145	-7.17154906
TBA4A	-0.081774	29.84050756	-0.599170513	0.57744901	0.670180342	-7.042149485
MFAP1	-0.082135278	7.150652472	-0.008691031	0.993431279	0.999364661	-7.251569328
MCM3	-0.082338444	24.57248967	-0.639350556	0.553183746	0.649779574	-7.014328725
NUP43	-0.082549	23.57833828	-0.638868576	0.553470782	0.649779574	-7.014671438
NDC1	-0.084942056	7.489676972	-0.008742312	0.993392522	0.999364661	-7.251568786
NUP98	-0.085029889	21.94995561	-0.248863026	0.81423488	0.873213665	-7.214327099
NAA50	-0.085844611	23.75823147	-0.66885608	0.535801629	0.633297959	-6.992940986
IPYR2	-0.087884111	24.55394817	-0.402279476	0.705555796	0.779401532	-7.155223403
TOM34	-0.089576444	22.91591133	-0.467619096	0.66145086	0.738806439	-7.122150603
U2AF2	-0.090422333	25.37583217	-0.833621639	0.445770012	0.565703373	-6.859575729
SRPRA	-0.092415389	22.52056353	-0.440754493	0.679406715	0.75446291	-7.136301535
NFU1	-0.093679222	22.51533872	-0.309645085	0.770415648	0.835466003	-7.194099937
CHMP5	-0.097521667	22.77669783	-0.243297314	0.818290164	0.876240397	-7.215965715
SYCC	-0.097609556	23.632802	-0.59105411	0.582432471	0.674861972	-7.047582802
EZRI	-0.099478389	26.58248347	-0.622555029	0.563244173	0.659076058	-7.026143303
RADI	-0.099478389	26.58248347	-0.622555029	0.563244173	0.659076058	-7.026143303
DPM1	-0.100599556	23.33966122	-0.292707528	0.782535558	0.845598484	-7.200163378
PRS4	-0.101252889	24.18700333	-0.500736888	0.639674801	0.721580252	-7.103663455
S10AB	-0.104971444	25.29250572	-0.299729569	0.777501886	0.840585203	-7.197689346
GALE	-0.105693778	23.95456356	-0.476407592	0.655632818	0.734529253	-7.117356032
PSA6	-0.106095389	25.19138969	-0.619198074	0.565269264	0.66108287	-7.028472956

PBDC1	-0.106145861	21.64966451	-0.62452757	0.562056448	0.658770944	-7.024769449
TALDO	-0.106342556	26.56821194	-0.744980991	0.492705883	0.59470201	-6.934165302
ERI2	-0.110075667	15.09535306	-0.011034654	0.991660036	0.999266671	-7.251541307
HYPK	-0.111210778	24.01607928	-0.48346708	0.650979804	0.731031708	-7.113446235
GDIR1	-0.112886944	26.52037586	-0.796957025	0.464757163	0.581440463	-6.891197825
CLIC1	-0.117536833	28.82065842	-0.523786651	0.62476163	0.707753345	-7.090128891
NELFE	-0.121320833	21.73996992	-0.441797495	0.678704854	0.754431048	-7.135766434
CAPZB	-0.124820333	24.8404735	-1.02079541	0.358201379	0.485046498	-6.682818329
SYNC	-0.127491111	25.95839267	-0.56849903	0.596423194	0.686581112	-7.062346517
NECP2	-0.129161389	7.401993472	-0.013202723	0.990021522	0.999266671	-7.251509455
AP2M1	-0.129803611	23.68616875	-0.605443316	0.573616214	0.668408042	-7.037906993
ACTN1	-0.131227556	25.46502656	-0.468292808	0.661003867	0.738806439	-7.121785926
THOC4	-0.132516	24.12082144	-0.625549938	0.561441495	0.658412138	-7.02405594
RBM14	-0.133581667	22.6030355	-0.554030147	0.605506645	0.69142233	-7.071554805
UBC12	-0.133921556	24.70623778	-0.490179219	0.646572768	0.727798123	-7.109680713
RPB3	-0.1349585	14.80960508	-0.013789408	0.989578145	0.999266671	-7.251499857
ESTD	-0.135466889	23.76276322	-0.199481786	0.850434762	0.904302699	-7.227599147
DUS3	-0.135561722	14.61122831	-0.014033583	0.989393615	0.999266671	-7.251495739
RRS1	-0.135822056	22.58207331	-0.676077447	0.531604572	0.629306468	-6.987585453
TBB3	-0.138416944	29.35371264	-0.895629844	0.415032456	0.535623	-6.803751718
SRC	-0.139117167	7.242049917	-0.014534302	0.989015211	0.999266671	-7.251487069
RHOA	-0.141584778	25.1418725	-0.951024717	0.389027419	0.510090072	-6.751528494
STIP1	-0.145828889	27.34779356	-1.316293046	0.250085074	0.423159824	-6.361635231
CBX3	-0.148905111	24.71316667	-0.875858904	0.4246456	0.545388205	-6.821861668
EFTS	-0.149324889	7.557011778	-0.015231184	0.988488567	0.999266671	-7.251474496
NPL4	-0.151081222	23.08536706	-0.872934796	0.426082249	0.546574822	-6.824515777
MAT2B	-0.151144222	23.39098644	-0.522250956	0.625748891	0.708495292	-7.09104747
RAC1	-0.153249111	24.98045433	-0.45476214	0.67001236	0.746325157	-7.129018384
PPGB	-0.153436222	14.96875267	-0.015511577	0.988276672	0.999266671	-7.251469271
SPTC2	-0.153464222	22.58492983	-0.360599303	0.734424319	0.805347741	-7.173899283
NUP62	-0.153923333	24.41344178	-0.506006399	0.636247586	0.718474498	-7.100617173
SDF2L	-0.154354222	24.29660011	-1.301682007	0.254635145	0.427803097	-6.378478281
APEX1	-0.157863556	24.78140844	-0.368102431	0.729187867	0.800942057	-7.170678925
SP16H	-0.158606	23.54897322	-1.022708491	0.357386026	0.485007643	-6.680890639
KPCB	-0.159250889	24.15438511	-0.747263002	0.491453485	0.594653138	-6.932325937
COPG1	-0.160337778	23.61434111	-1.053485215	0.34448542	0.478085724	-6.649572383
CRKL	-0.160825889	25.31641428	-1.12183468	0.317270703	0.470743312	-6.578048118
TPIS	-0.163282167	28.13034614	-1.02050138	0.358326835	0.485046498	-6.683114404
ACTN4	-0.166067	26.31949017	-1.444934501	0.213230127	0.376954088	-6.20996271
THYG	-0.166930222	8.563805444	-0.014748551	0.988853299	0.999266671	-7.251483266
CYFP1	-0.169630278	7.237914028	-0.017731894	0.986598804	0.999266671	-7.251424533
XRP2	-0.169970167	0.084985083	-0.905038488	0.410519167	0.531084262	-6.795034039
PP1R8	-0.171038333	22.35880956	-0.926832639	0.40021638	0.519647578	-6.774598837
HAP28	-0.173742667	24.509869	-0.602592098	0.575356333	0.669208784	-7.03984003

UBA1	-0.175044111	25.67710094	-1.446900347	0.212709715	0.376954088	-6.207601986
TMCO1	-0.177614389	23.07262892	-0.194246554	0.854299694	0.907055252	-7.22883805
COPB	-0.179304778	23.85898972	-1.018358154	0.359242427	0.485519199	-6.685270917
ZCH18	-0.180587333	23.43417289	-0.56137719	0.60088372	0.689459196	-7.066904826
PPAC	-0.181410333	24.31592172	-0.581195133	0.588522354	0.680808279	-7.054097046
XPO7	-0.185893444	23.21039983	-0.480997749	0.652605296	0.731906624	-7.114819748
PA1B3	-0.186551667	23.69789483	-0.782415854	0.472454553	0.584943733	-6.903442301
SYAM	-0.188530667	7.282842333	-0.01958574	0.985197934	0.999266671	-7.251382602
HNRDL	-0.188786333	25.37645661	-0.667322151	0.536696056	0.633925757	-6.994072507
ALDOA	-0.190247333	29.039263	-1.091181015	0.329234014	0.474639437	-6.610451425
AIMP1	-0.191876111	24.81158128	-1.769361124	0.142402685	0.276253435	-5.807670373
ASH2L	-0.1933275	7.414412583	-0.019727683	0.985090676	0.999266671	-7.25137922
FAKD4	-0.197302778	23.37413194	-0.579988437	0.589270464	0.680937834	-7.054887881
FUBP2	-0.200376778	25.51192828	-1.279122935	0.261813092	0.436082431	-6.404313724
NU155	-0.200567778	23.29365344	-0.851042437	0.436960139	0.5585126	-6.844184098
SMAP	-0.200867333	24.41314333	-1.368656506	0.234401839	0.404324208	-6.300599164
PYM1	-0.202746056	15.04651608	-0.020336226	0.984630842	0.999266671	-7.251364442
HEMGN	-0.204670667	25.18463167	-0.668743046	0.535867504	0.633297959	-6.993024439
FA49B	-0.206145667	24.26901061	-0.654296946	0.544331903	0.641878107	-7.003594361
LKHA4	-0.206819833	22.71504197	-0.993074907	0.370193946	0.494829776	-6.710493538
F10A1	-0.207079667	25.72805606	-1.00606234	0.364533689	0.490028893	-6.697587619
NSF1C	-0.207906111	23.53300661	-1.234803947	0.276466263	0.450266647	-6.454440329
SYLC	-0.208112444	25.00086344	-1.873752024	0.125139945	0.251696569	-5.67447942
RUXF	-0.208510667	24.79006478	-1.146954176	0.307755858	0.467680797	-6.551117117
RIC8A	-0.208996611	23.13043119	-0.323945328	0.760241635	0.827159006	-7.188726272
PP4C	-0.211031222	23.50367139	-0.467500172	0.66152978	0.738806439	-7.122214926
RL7A	-0.214143	26.75939861	-1.170409282	0.299102599	0.463625532	-6.525675101
PYRG1	-0.216760667	23.24460767	-0.720400399	0.506342577	0.605113438	-6.953696739
LASP1	-0.224548667	23.86937511	-0.941819413	0.393254324	0.513736653	-6.760354226
AP1B1	-0.225958389	23.90336203	-1.88023124	0.124143135	0.250163672	-5.666177171
PRS7	-0.226201833	25.03816414	-1.063746701	0.340274159	0.475655674	-6.639004491
CHM4B	-0.228359778	23.61454078	-1.072781179	0.336603441	0.474639437	-6.629649317
DRG1	-0.230389556	23.60324844	-0.855530864	0.434712389	0.555972893	-6.840181
PAIP1	-0.23221	22.75898389	-0.915619239	0.40549086	0.525216594	-6.785154862
KCRB	-0.233904111	27.60143706	-1.063303226	0.340455234	0.475655674	-6.639462484
RL17	-0.234517222	25.76701794	-0.518536165	0.628140801	0.710826002	-7.09325958
MDHC	-0.236517444	26.91760094	-0.491687757	0.645584598	0.727092638	-7.108827989
SYMC	-0.237718889	25.14425656	-1.417054953	0.220745018	0.388326334	-6.243312411
T2FA	-0.238910333	22.76197161	-1.891533237	0.122424218	0.247401358	-5.651686936
UB2V1	-0.239796444	24.48743822	-1.522114632	0.193705425	0.351771691	-6.116436545
TR150	-0.240413556	22.87434667	-1.148978763	0.307000175	0.467680797	-6.548932124
SYWC	-0.243880111	26.29712028	-1.214492111	0.283431642	0.454544056	-6.477121322
SAR1B	-0.245587889	24.43276706	-1.699660801	0.155283773	0.29638765	-5.895840336
RCC1	-0.247446111	23.14876272	-0.933446896	0.39713145	0.516901253	-6.768331231

SDHB	-0.247519889	23.33010483	-0.803791038	0.461172324	0.579365403	-6.885384373
MTPN	-0.248998889	26.29012689	-0.512121835	0.632283396	0.714376364	-7.097046167
PARK7	-0.250517111	27.45498122	-1.561206394	0.184500018	0.341749816	-6.068458022
EPN4	-0.250931667	22.81299639	-1.910495539	0.119596125	0.243765717	-5.627353324
IF2G	-0.251525111	25.82434478	-1.747089699	0.14639473	0.282455714	-5.835919716
SRP68	-0.253274333	23.59137039	-1.356191954	0.238047999	0.407645248	-6.315220514
TADBP	-0.254313667	23.89377617	-0.759975037	0.484519384	0.589609205	-6.921999513
TXD17	-0.254570222	24.99749833	-1.061538046	0.3411768	0.47603988	-6.641284318
CC124	-0.256358111	22.75517628	-0.900775892	0.412559014	0.533076253	-6.798991465
TBB2A	-0.256669	29.48320494	-1.612344749	0.173113403	0.324606662	-6.005152858
AMPB	-0.259369778	22.35664872	-0.357826067	0.736364037	0.806746211	-7.175073709
PPP5	-0.259632778	23.11732928	-1.15664568	0.304153513	0.466782272	-6.540638624
RUVB1	-0.260013556	24.80148022	-1.892545836	0.122271435	0.24732704	-5.650388192
LAGE3	-0.261106	15.87832033	-0.024881221	0.981196734	0.999266671	-7.251239884
MCTS1	-0.267376222	23.86437833	-0.989169021	0.371910623	0.496015142	-6.714353834
PGM2	-0.267896111	23.35325106	-0.551385294	0.607176125	0.692441123	-7.073215647
PGK1	-0.268887778	27.55387633	-2.566872173	0.054558577	0.156763999	-4.78418014
DYLT1	-0.269340667	22.60844817	-1.00896003	0.363280817	0.488652809	-6.694693538
CAND1	-0.269999444	23.51523317	-1.221350303	0.281062021	0.453428044	-6.469484412
ROAA	-0.270409	25.51134794	-1.966268301	0.111669791	0.232273164	-5.555644478
COR1B	-0.270780333	23.45732017	-0.308540511	0.771203775	0.8358955	-7.19450534
PTSS1	-0.272225778	5.096058	-0.058718144	0.95565092	0.991597768	-7.249525995
SIAS	-0.273571111	24.31086467	-0.652951253	0.545124958	0.642457938	-7.004569262
UBC9	-0.274382722	24.79708214	-0.982021296	0.375069404	0.498621463	-6.721392632
DCNL1	-0.275073167	15.13676719	-0.027487242	0.979227885	0.999266671	-7.251157173
IF5	-0.2757	24.86811278	-0.778705947	0.474433566	0.585192204	-6.906538747
THIO	-0.276782778	27.09156861	-0.557245329	0.603480921	0.690731512	-7.069526476
SAHH2	-0.277036556	23.35342406	-0.409091704	0.700889972	0.774648741	-7.151989827
COPA	-0.277553667	24.04201306	-1.533651069	0.190942393	0.348535258	-6.10231721
PHF5A	-0.280166889	22.82082789	-1.307388492	0.252848884	0.426145312	-6.371910182
C1TC	-0.282023444	24.22372361	-1.710273027	0.153247983	0.293289677	-5.882463138
SYRC	-0.283952778	24.85831283	-2.578470806	0.053835065	0.155103186	-4.769488869
ABC3C	-0.284674222	15.40779211	-0.027957792	0.978872402	0.999266671	-7.251141361
FSCN1	-0.287869444	27.08953161	-1.774463301	0.141504052	0.274760145	-5.801189438
PRP19	-0.290527778	24.87168033	-2.549207659	0.055681363	0.158919232	-4.806579345
PNPH	-0.291547333	25.69335356	-1.549020679	0.187321921	0.344879392	-6.083454373
SRP14	-0.292221944	15.41285103	-0.028623922	0.978369175	0.999266671	-7.251118519
KPYM	-0.292246111	27.53508006	-2.723121493	0.045657244	0.135516146	-4.587437278
FKBP8	-0.292731667	23.42709394	-0.891996143	0.416786125	0.536586968	-6.807101496
OTUB1	-0.294516444	23.91224511	-2.232960109	0.080793475	0.198444023	-5.21159485
RHOC	-0.294859556	24.93083067	-1.718210993	0.151743066	0.290670455	-5.872445299
PSB7	-0.297330778	23.97088817	-0.992203627	0.370576305	0.495030502	-6.711355498
CMIP	-0.299624	7.308358333	-0.031014228	0.976563522	0.999266671	-7.251032126
CYTB	-0.302769556	24.65197522	-1.228518013	0.278604874	0.45170007	-6.461479644

VPS29	-0.303099333	23.28158056	-1.048399227	0.346589307	0.478472747	-6.654787181
RU1C	-0.303304444	24.84809444	-0.253234412	0.81105453	0.87111751	-7.213014824
MTDC	-0.304100222	23.67772922	-1.488293735	0.202035261	0.36379998	-6.157627103
AP2A1	-0.304776111	23.66261861	-1.90040971	0.12109171	0.245684089	-5.640299424
NADC	-0.306217111	23.74593211	-0.499069513	0.64076142	0.722041938	-7.10462141
CLPX	-0.309815722	14.77141997	-0.031697435	0.976047452	0.999266671	-7.251006161
TBB6	-0.310522889	29.17274522	-2.018684441	0.104726118	0.221504051	-5.488108253
PSMD2	-0.311517333	24.050798	-1.420935746	0.219683787	0.387079201	-6.238684985
PA2G4	-0.311588722	26.84330197	-2.702468175	0.046734795	0.137622352	-4.613290861
SUMO2	-0.317210778	26.63821472	-2.28573216	0.07585568	0.193794083	-5.143607793
NHRF1	-0.319073778	24.19122978	-0.751712869	0.489018003	0.59225155	-6.928726585
HDGF	-0.321603722	26.51605831	-0.697206007	0.51945494	0.616292672	-6.971647343
TRI25	-0.323097889	22.63344539	-0.751396453	0.489190891	0.59225155	-6.928983074
GDIA	-0.323497222	24.50887217	-1.445637559	0.213043866	0.376954088	-6.209118565
PSA7	-0.326392333	25.52848361	-1.942926136	0.114917494	0.236749089	-5.585678824
CALM1	-0.327151778	27.34663967	-1.611152699	0.173370632	0.324803327	-6.006635012
MARE3	-0.327943667	23.29552783	-1.038617014	0.350666939	0.480779362	-6.664773687
HNRH2	-0.327952556	25.81929761	-1.916973425	0.118645801	0.242291999	-5.619034533
ZC3H4	-0.328766556	14.59685939	-0.034077453	0.974249781	0.999266671	-7.250911293
NDUS3	-0.331180333	23.7929765	-1.30539255	0.253472317	0.426526608	-6.374208972
FLNA	-0.334712222	25.95662522	-2.253089295	0.078869894	0.197337958	-5.185650095
SYFA	-0.334837778	24.032426	-2.625817823	0.050990563	0.148513497	-4.709655586
DHX15	-0.335036	24.15285833	-2.030891743	0.103175844	0.218782839	-5.472365277
DPP3	-0.335116222	23.23135	-1.152157188	0.305817169	0.467602559	-6.545497605
TCPB	-0.338123667	26.31108517	-2.999830567	0.033621626	0.108831872	-4.246163623
RL18	-0.342277667	26.8089885	-1.455140914	0.210541643	0.375058026	-6.197693354
HBE	-0.343312889	28.665227	-1.074701332	0.335827728	0.474639437	-6.627654919
OSGEP	-0.344148333	23.41802906	-1.384480967	0.229849674	0.399044437	-6.281956833
HCFC1	-0.345298333	22.61571828	-1.835804456	0.131148544	0.259874696	-5.723028686
PRP8	-0.349337222	23.72222561	-1.460131308	0.209239172	0.373616344	-6.191682857
NU214	-0.351614	22.94341167	-1.311994955	0.251415553	0.425073718	-6.36659871
SET	-0.352007333	27.92841656	-2.540025778	0.056275114	0.160184971	-4.818233805
GFPT1	-0.3539425	23.75038808	-1.811834618	0.135098198	0.264004912	-5.753621693
SYTC	-0.355697556	25.82843289	-2.512915152	0.058069688	0.164415106	-4.852689664
SYEP	-0.358300778	25.84909706	-2.964011233	0.034955902	0.111399077	-4.289772
WDR1	-0.358909667	25.43063494	-2.445996356	0.062777126	0.172697849	-4.938007163
MAP4	-0.359027556	24.01742733	-2.007406176	0.106180465	0.223471619	-5.502648917
EMD	-0.359462333	23.49030961	-2.080347134	0.09714143	0.21199539	-5.408549787
UBE2N	-0.361769444	24.51069106	-0.778018728	0.474800833	0.585192204	-6.907111094
XRCC6	-0.364414556	25.56770183	-2.586456599	0.053343133	0.154102383	-4.759381362
MEP50	-0.367920778	23.10994739	-0.586541758	0.585214798	0.677717517	-7.050575995
TPR	-0.370369722	23.22685158	-1.286718019	0.259375587	0.433376764	-6.395638978
RL36A	-0.373243	25.77822761	-1.256733488	0.26912329	0.443066296	-6.429743983
HNRPU	-0.374015667	27.24546094	-2.517318574	0.057773932	0.163795245	-4.847088744

ACTBL	-0.374520444	30.39620978	-1.857249231	0.12771687	0.255673584	-5.695609119
DNJA2	-0.376707444	24.12750928	-3.046246772	0.031977953	0.105537145	-4.189920753
HDAC1	-0.377906556	23.7845265	-2.005611983	0.106413802	0.223521444	-5.504961703
DDX6	-0.378783	24.4357915	-1.555863363	0.185732081	0.343347892	-6.075037816
AP2B1	-0.384353944	24.12234381	-1.602335041	0.175285339	0.326953931	-6.017589287
TXNL1	-0.384506444	25.20908656	-1.736605999	0.148313575	0.284868956	-5.849193567
RL35A	-0.386406667	25.23106578	-1.055970695	0.343461281	0.477394246	-6.647018377
VATF	-0.386549833	7.547272306	-0.038740928	0.9707279	0.998554062	-7.250705512
EF1A2	-0.386794444	29.082048	-1.993590329	0.107991365	0.226117961	-5.520454984
NDKB	-0.3887695	28.05991319	-2.945241171	0.035679053	0.113196042	-4.312694155
VINC	-0.389600333	25.12915283	-1.217691112	0.282324066	0.454013832	-6.473561804
CAH1	-0.389689667	24.28103161	-1.604451781	0.174823771	0.326378528	-6.01496114
PCBP1	-0.391880111	27.18309994	-2.853723857	0.039457061	0.122093547	-4.425132317
TBB5	-0.3923015	29.71859469	-1.766955968	0.14282834	0.276827291	-5.810724301
SAR1A	-0.393422333	24.63371161	-1.967732035	0.111469396	0.232082766	-5.553760137
RANG	-0.395053889	26.76203639	-3.796785298	0.014916918	0.061280451	-3.325591786
MGST1	-0.395627	23.84313572	-0.648721936	0.54762247	0.645044809	-7.007622395
NU107	-0.396004	22.342972	-0.801629155	0.462304092	0.579365403	-6.887227451
PUR9	-0.397377333	25.72851222	-2.298249892	0.074733341	0.192662012	-5.127497398
CSK2B	-0.39752	24.39903022	-1.142499848	0.309424304	0.467680797	-6.555916873
RL32	-0.398096556	25.62107206	-1.019973422	0.358552196	0.485046498	-6.6836459
RL15	-0.398897889	25.9161585	-1.272747291	0.263875718	0.438108121	-6.411577026
AN32E	-0.40019	24.73908756	-0.815442726	0.45510868	0.574477032	-6.875386925
HS90A	-0.400434778	30.12220461	-2.617285819	0.051490508	0.149560985	-4.720420841
BUB3	-0.400766111	24.44258306	-2.793489238	0.042190272	0.128683348	-4.499728272
UB2G2	-0.400819222	15.57657683	-0.038933309	0.970582629	0.998554062	-7.250696457
TAGL2	-0.401061667	27.79310694	-2.199181799	0.084136916	0.200136416	-5.255158959
CPSF5	-0.402711111	24.90908778	-1.453081185	0.211081525	0.375647589	-6.200171942
TCPH	-0.405286778	26.62795939	-3.015243576	0.033065387	0.107956209	-4.227453919
RUVB2	-0.406117222	24.44566872	-1.903950528	0.120564431	0.245504648	-5.635755299
NDKA	-0.406573056	28.05101142	-3.075193292	0.030999317	0.102944773	-4.155000898
RL5	-0.406733	26.08844428	-1.886852235	0.123133103	0.248598273	-5.65768967
NUP88	-0.4091815	22.81632525	-1.900263747	0.121113498	0.245684089	-5.640486727
DYL1	-0.410280889	25.47977278	-1.137945831	0.311138433	0.467680797	-6.560813348
PHP14	-0.41105	15.83895411	-0.039259663	0.970336195	0.998554062	-7.250680994
CP51A	-0.411387667	24.17910694	-3.831447633	0.014431381	0.060328834	-3.287801864
TBCD	-0.412434556	22.31762239	-1.954796395	0.113253631	0.234651839	-5.570409058
PSB1	-0.413375667	25.29238117	-1.863409535	0.126748504	0.254212427	-5.687724476
ELOC	-0.413630333	24.76742183	-1.723374256	0.150772319	0.289280613	-5.865923948
LDHA	-0.413758333	28.72889728	-2.966842127	0.034848287	0.111395542	-4.286319072
MP2K2	-0.413968333	22.5761275	-0.611710695	0.569803095	0.665290361	-7.033630645
UBP5	-0.415068333	23.89185883	-1.666850983	0.161754003	0.307087742	-5.93707968
IMA3	-0.415316167	22.60991981	-0.88555024	0.419911542	0.539633157	-6.813020174
MCM2	-0.416513667	24.94337594	-2.323016636	0.072565868	0.188671256	-5.095644175

SF01	-0.416585444	23.73777239	-0.893773227	0.415927744	0.535850814	-6.80546446
SVIP	-0.416586722	7.983513694	-0.042338056	0.968011854	0.998554062	-7.25052879
RL36	-0.417866778	25.84652406	-0.942373083	0.392999028	0.51371792	-6.759825016
SRRT	-0.419352278	23.17435819	-0.808074759	0.458935951	0.577939425	-6.881721351
SAE2	-0.420437333	24.53601489	-2.083626408	0.09675488	0.211570672	-5.404316883
PRDX2	-0.422041667	27.70302083	-0.961135683	0.384427842	0.505970307	-6.741768464
HXK2	-0.423034778	22.92221006	-1.546327572	0.187951347	0.345739666	-6.086763796
RAE1L	-0.426010778	23.2825885	-1.348368407	0.240364094	0.410951282	-6.324368903
TPM3	-0.4278305	26.53336531	-0.783296254	0.471985817	0.584943733	-6.902705832
FAD1	-0.433221056	15.14147492	-0.046674793	0.964738033	0.996667789	-7.250294902
MCM6	-0.433234778	24.48111883	-3.337804547	0.023527633	0.084536772	-3.843841927
PFD6	-0.433300222	24.33915033	-2.415534612	0.065058468	0.177145151	-4.976959947
RU2A	-0.433500333	23.72607306	-1.816841447	0.134263096	0.262613689	-5.747236495
RL12	-0.434665667	27.12711906	-2.133905201	0.09103048	0.205155373	-5.339408546
SR140	-0.435504389	7.419707028	-0.04439305	0.966460435	0.997817747	-7.250420799
HMGB1	-0.436620889	27.04973322	-1.84557285	0.129573595	0.25831135	-5.710544198
PEBB	-0.438149222	23.06550517	-1.835756823	0.131156272	0.259874696	-5.723089539
IF4B	-0.438710444	24.29176211	-1.178228102	0.296267052	0.461761342	-6.517132309
SND1	-0.439936444	25.76387567	-3.509458931	0.019764537	0.074448751	-3.6461477
SEP11	-0.440450222	24.00618233	-2.862237506	0.039087126	0.121230304	-4.414626017
CDC37	-0.441636556	26.07551528	-3.028851634	0.032582981	0.107036849	-4.210962963
UCHL3	-0.442920667	22.99664667	-2.039002363	0.10215937	0.217851324	-5.461903213
CAZA1	-0.449770889	25.27477822	-3.899919942	0.013525373	0.057328223	-3.213707684
RL30	-0.455292778	27.02230394	-2.002521757	0.10681697	0.224147421	-5.508944851
PSMD1	-0.456359	24.91213361	-4.432303505	0.00836087	0.042040977	-2.662441766
MOES	-0.458688778	27.07739017	-3.22976337	0.026318983	0.091091026	-3.970603889
UBXN1	-0.458960222	23.99554656	-2.752493241	0.044172564	0.13321682	-4.550755365
K1C19	-0.459471	23.57767839	-1.397138894	0.226269558	0.394768165	-6.266982372
KAD2	-0.459509778	25.96011811	-2.86144897	0.039121224	0.121230304	-4.415598719
PSMD9	-0.461830889	23.15598033	-2.976669363	0.034477624	0.110869222	-4.274341045
HINT1	-0.462662889	24.64213778	-1.707519191	0.153773628	0.293956743	-5.885936208
SEPT6	-0.465405556	23.62157989	-3.800106937	0.014869574	0.061280451	-3.321962223
TNPO3	-0.466825944	22.64690519	-1.124278922	0.316333517	0.470256967	-6.575442311
PTBP1	-0.469842833	26.77796747	-1.68208049	0.15871722	0.302129566	-5.917960374
CBS	-0.470598111	24.05495128	-1.542073993	0.188949766	0.346977521	-6.091987173
AL1A2	-0.473857778	25.27356789	-1.100716122	0.325470874	0.474639437	-6.60042759
HNRL1	-0.474423222	23.53971906	-1.530013794	0.191809298	0.349502444	-6.10677251
2AAA	-0.474608222	25.51174111	-2.869576967	0.038771357	0.120672312	-4.405576253
DDX23	-0.475038778	22.545358	-1.490256253	0.201542414	0.363526588	-6.155245472
MATR3	-0.476906778	24.17329628	-2.940834969	0.035851262	0.113573388	-4.318082
BOLA2	-0.480110889	24.46439011	-1.455016087	0.210574323	0.375058026	-6.197843601
PSA5	-0.482184222	25.97756878	-1.604849242	0.174737238	0.326378528	-6.014467545
TM243	-0.484012222	7.703250778	-0.047518844	0.964100947	0.996667789	-7.250246733
TBCA	-0.486349667	25.41711839	-1.624940569	0.170418757	0.320116996	-5.989473734

SAHH	-0.486776556	26.69843883	-3.213560394	0.026769591	0.092201563	-3.989767101
RL27A	-0.488602889	26.30014733	-1.181602042	0.295050988	0.460998859	-6.513436613
ADHX	-0.489178	25.22615656	-4.62651143	0.007083513	0.037690357	-2.471993831
U520	-0.490202333	22.98145883	-2.742800393	0.044656432	0.133598768	-4.562849397
EF1B	-0.492131	26.98059828	-3.140049825	0.028928221	0.097741628	-4.077200341
SRSF7	-0.493463889	25.87989206	-3.149789575	0.028631064	0.097045197	-4.065569861
RL7	-0.495863778	26.66966144	-4.307843759	0.009322713	0.044665224	-2.78743417
SC24C	-0.496950667	23.37888878	-2.135417753	0.090864017	0.205076008	-5.337455863
HNRH3	-0.503079556	23.40368039	-0.929302321	0.39906223	0.518979609	-6.772262139
BLVRB	-0.503666556	27.36224661	-2.902126426	0.037405323	0.117352244	-4.365526696
IQGA1	-0.505456556	24.40855839	-3.1875037	0.027512914	0.094002455	-4.020666851
HMGB2	-0.506788778	25.4186195	-2.752413974	0.044176497	0.13321682	-4.550854223
RL18A	-0.510077333	26.10897456	-2.48298219	0.060125203	0.168224321	-4.890807005
1433Z	-0.510861778	28.427573	-3.50054182	0.019941999	0.074500673	-3.656304269
RTRAF	-0.512813278	23.32006675	-0.994364223	0.369628741	0.494829776	-6.709217122
RD23B	-0.517063333	24.594142	-1.447168241	0.212638892	0.376954088	-6.20728019
SNP29	-0.517073222	24.35860617	-1.923962571	0.11762938	0.240862844	-5.610055983
VASP	-0.521632333	23.29463706	-3.995471558	0.012369722	0.054710186	-3.111541729
PSMD3	-0.522765778	24.66963656	-3.365813574	0.022860637	0.08274852	-3.811271502
IF4G2	-0.525325556	23.50496122	-0.921547258	0.402695503	0.521912956	-6.779585342
GSTP1	-0.525592389	28.77084925	-3.225506696	0.026436514	0.091349509	-3.975634419
ATX10	-0.527576667	23.42941833	-1.863878938	0.126675031	0.254212427	-5.687123541
NUDC2	-0.527924444	23.93535544	-2.63642225	0.050376725	0.146926371	-4.696286008
HSPB1	-0.528029944	27.75069931	-4.408357627	0.008536492	0.042423778	-2.68630975
MTA2	-0.528838111	24.08785217	-0.817374175	0.454109428	0.574235647	-6.873719317
TBB4B	-0.529201944	29.52837414	-3.107873024	0.029935293	0.100093988	-4.115722433
TCPQ	-0.529458333	26.58410228	-2.891202718	0.037857599	0.118173355	-4.378951931
K1C18	-0.529655778	24.60747178	-0.982478982	0.374866469	0.498621463	-6.720942913
RLA2	-0.531300056	28.33826025	-4.628408914	0.007072219	0.037690357	-2.470160261
TCPA	-0.531750111	25.77137194	-3.144488897	0.028792351	0.097436972	-4.071897801
CAN2	-0.539792778	23.4014675	-1.757928236	0.14443777	0.27943859	-5.822180369
HS90B	-0.539796111	30.05252394	-3.557131733	0.018846238	0.071622422	-3.592060414
RCOR1	-0.541121444	22.30776733	-2.470002771	0.061041457	0.170214998	-4.907358529
ARK72	-0.542220722	22.90868164	-1.528680773	0.192127985	0.349502444	-6.108404486
SF3B1	-0.545762222	23.49742833	-2.990905783	0.033948576	0.109498281	-4.257012502
CUTA	-0.546877889	25.20508494	-0.869248288	0.42789894	0.548575189	-6.827852865
RS28	-0.549754722	26.93662953	-2.761996866	0.043703861	0.132165436	-4.538908284
RACK1	-0.551533667	26.96970983	-4.377207643	0.008771496	0.042990411	-2.717486627
AK1A1	-0.551862611	22.77152181	-2.612268477	0.051787061	0.150217707	-4.726754971
PRS6A	-0.552883333	24.89571111	-1.856116927	0.127895702	0.255791404	-5.697057993
ACLY	-0.553122111	24.70495217	-2.870736615	0.03872173	0.120672312	-4.404147015
PDC10	-0.553667889	24.22813283	-1.250221241	0.271284842	0.44524964	-6.437100074
E41L3	-0.555624833	22.77988103	-3.689991265	0.016536933	0.06494956	-3.443214519
RO60	-0.556890778	23.2813585	-2.607083786	0.052095511	0.150907104	-4.733303071

RSSA	-0.559471444	27.55063039	-2.08869196	0.096161008	0.210704286	-5.397778019
SRRM2	-0.559806444	23.86893689	-2.185812054	0.08550147	0.200538102	-5.272409495
PPP6	-0.560167222	22.84971361	-3.325208111	0.023834887	0.084844192	-3.858529144
PDLI1	-0.561364889	24.40074456	-1.347606251	0.240590866	0.411009397	-6.325258917
SEPT2	-0.562707667	24.51174694	-2.792123899	0.042254655	0.128695606	-4.501424396
SEPT8	-0.564192444	23.61335322	-1.370087909	0.233986551	0.403934677	-6.298916505
SYVC	-0.564433889	24.85494061	-3.688739006	0.016557109	0.06494956	-3.444604442
DNPH1	-0.565871889	23.64070006	-2.122433697	0.092303708	0.206277438	-5.354218323
YBOX1	-0.572247444	26.9871785	-4.630154035	0.007061851	0.037690357	-2.468474383
PARP1	-0.573329889	24.79807861	-3.609791373	0.017888795	0.068967291	-3.532731228
RS20	-0.574242889	26.206472	-2.595735153	0.052777848	0.152676217	-4.747645521
URP2	-0.575530222	25.98893467	-2.258933261	0.078320813	0.19671707	-5.178120347
RBM8A	-0.577950222	23.97568089	-2.356334718	0.069757895	0.184979892	-5.052843369
SEPT9	-0.580826889	23.88389656	-2.463872623	0.061479566	0.170892353	-4.915180498
RL19	-0.583209889	26.62760883	-3.194828983	0.02730158	0.093430127	-4.011969788
RL6	-0.584234111	26.25014372	-3.255189766	0.025629301	0.089283775	-3.94061197
PFD3	-0.585576444	23.369331	-4.560605504	0.007489334	0.038755487	-2.536004052
SRC8	-0.585909444	24.28745506	-4.336578633	0.009089557	0.044143363	-2.758369528
LDHB	-0.587302111	28.72517783	-5.229724924	0.004361223	0.028091017	-1.914469971
PRDX1	-0.587862778	28.48003806	-2.341415488	0.071000246	0.186419365	-5.072001195
TCOF	-0.588829944	23.19733892	-3.959327428	0.012792752	0.055614234	-3.150019637
UB2D3	-0.589924111	24.66459917	-1.234218579	0.276664779	0.450266647	-6.45509661
PR40A	-0.590097056	22.73333181	-3.330861199	0.023696429	0.084624432	-3.851934728
PSA1	-0.597819778	25.36846	-2.018933895	0.104694191	0.221504051	-5.487786593
SBDS	-0.600165111	23.24689989	-2.550856177	0.0555755	0.158919232	-4.804487726
PP14B	-0.602917778	23.90226189	-1.499637512	0.199202778	0.359610773	-6.143846012
RLA0	-0.603048111	26.97030572	-1.986920108	0.108877374	0.227575061	-5.529048966
ASML	-0.603707667	24.74231661	-2.119572206	0.092624285	0.206347937	-5.357912556
COPB2	-0.606294667	24.17161911	-1.642356476	0.166762479	0.314913733	-5.967742135
K2C8	-0.606388222	25.05097044	-4.381518622	0.008738522	0.042927488	-2.713163262
NUP85	-0.607450611	22.63100642	-3.720005061	0.016061811	0.064126929	-3.409975336
IMB1	-0.608234889	25.32686667	-4.294520168	0.009433205	0.045034731	-2.800953045
G6PI	-0.609553	26.98415794	-3.582494845	0.018377866	0.07021794	-3.563430398
PIPNB	-0.611990222	23.00794367	-1.601102963	0.175554565	0.327169871	-6.019118596
MTNB	-0.613823944	23.27702792	-0.894528307	0.415563447	0.535850814	-6.804768194
TTC1	-0.614004889	23.486025	-2.742813407	0.044655778	0.133598768	-4.562833151
RTCB	-0.615810222	23.00190911	-4.568929042	0.007436595	0.038755487	-2.527885044
RL13	-0.617859333	26.45775611	-3.183229531	0.027637089	0.094275637	-4.025745126
SPEE	-0.620390333	25.61818161	-4.091192127	0.011326175	0.051159757	-3.010624903
NQO1	-0.621366778	24.43792317	-3.384442115	0.022429086	0.081324511	-3.789676331
PUR6	-0.623343778	25.97769833	-4.337679329	0.009080764	0.044143363	-2.757258678
SYIC	-0.623387444	24.47624928	-5.660839517	0.003162538	0.022938567	-1.545335779
PCNA	-0.624045333	26.33081656	-3.574238152	0.018528843	0.070649423	-3.572739471
XPO2	-0.624084111	25.23167117	-2.460075502	0.061752682	0.170982752	-4.920027057

NAA10	-0.625580444	23.58827356	-4.047878953	0.011785063	0.052547061	-3.056112765
FA98A	-0.631063278	22.46397836	-2.204879055	0.083562624	0.200136416	-5.247809138
TBB4A	-0.635108278	29.47542097	-3.304553089	0.024348758	0.086519252	-3.882665246
TPM4	-0.635997444	25.34917183	-0.796434855	0.465031934	0.581440463	-6.891640477
PSB5	-0.63747	24.44132833	-4.240150444	0.009900348	0.046595013	-2.856399098
TTL12	-0.638096444	24.12742156	-4.647145357	0.006961835	0.037690357	-2.452082865
ARP2	-0.639119444	23.93812006	-3.233348672	0.026220455	0.090897577	-3.966368903
SF3A3	-0.640587556	22.87577522	-3.397898587	0.022123192	0.080903337	-3.774110338
PSMD5	-0.641883111	24.35480544	-3.011259062	0.033208173	0.108256613	-4.232287492
MAGB2	-0.643277389	23.24178264	-0.988940296	0.372011357	0.496015142	-6.714579584
HEM3	-0.643812667	24.73539611	-2.086654171	0.096399442	0.211009867	-5.400408529
NACA	-0.645573333	26.46947611	-5.035691514	0.005073587	0.030774364	-2.088382761
ARF4	-0.647665	25.26539661	-4.435627476	0.008336829	0.042019196	-2.659135363
DCUP	-0.648013222	24.59226006	-4.582620495	0.007350793	0.038684907	-2.514552013
NUP35	-0.649863111	22.81340922	-2.72808453	0.045402482	0.135060623	-4.58123193
1433E	-0.650521778	28.03901911	-2.662723495	0.048889629	0.142784505	-4.663178047
TCEA1	-0.654658111	23.3314465	-2.951758907	0.035426037	0.112560821	-4.304729193
PFD2	-0.657770556	24.04299861	-1.878460648	0.124414708	0.25047418	-5.668446298
LGUL	-0.661364222	24.982938	-3.627803201	0.017574344	0.068124549	-3.51253852
NP1L1	-0.662556889	25.62770789	-2.838665418	0.040121113	0.123968425	-4.443738038
ACINU	-0.662564556	16.06416217	-0.073724796	0.944339022	0.985477629	-7.248322423
SH3L3	-0.662781278	25.19671881	-3.007209745	0.033354001	0.108506939	-4.237201958
RL26	-0.662884778	25.1435305	-1.786471452	0.139412131	0.271936564	-5.785923309
MGN	-0.662890111	24.30275894	-0.841335268	0.441852329	0.563102436	-6.852789267
EF1A1	-0.663452778	29.54318528	-4.960401937	0.005386675	0.032044104	-2.157222855
RS21	-0.664078889	25.64522689	-2.258768817	0.078336207	0.19671707	-5.178332211
PDCD5	-0.664803889	24.23636106	-2.269786083	0.077312131	0.195590625	-5.164140247
NTM1A	-0.665313333	23.11980911	-4.426232293	0.008404991	0.042064414	-2.66848514
NPM	-0.665664833	28.90416092	-4.224220135	0.010042334	0.046849576	-2.872730082
HNRPR	-0.669453111	24.979063	-3.683105915	0.016648221	0.065126618	-3.450859867
PRP6	-0.669620889	15.14743244	-0.066842539	0.949525111	0.98702464	-7.248908192
PABP4	-0.672630139	26.21547046	-4.070792084	0.011539648	0.051794798	-3.032012913
SKP1	-0.672683556	24.33958144	-5.099089947	0.004826586	0.02965499	-2.031008655
PPCE	-0.673783167	22.92915119	-2.089261769	0.09609445	0.210704286	-5.397042466
ARHG1	-0.674865389	22.55687186	-5.809566656	0.002843273	0.021708241	-1.423248744
HBG1	-0.675111083	28.28920365	-3.99108878	0.012420136	0.054710186	-3.116196614
HBG2	-0.675111083	28.28920365	-3.99108878	0.012420136	0.054710186	-3.116196614
RL4	-0.677147556	27.09015689	-6.016255606	0.002461146	0.020240467	-1.257833248
PIHD1	-0.679934611	23.07598697	-2.929876865	0.036283646	0.114602567	-4.331492819
FKBP5	-0.681669667	23.02824517	-3.968276647	0.012686455	0.055425249	-3.140473541
THUM1	-0.681915667	23.71306828	-3.814383091	0.014668075	0.06084112	-3.306382457
PGAM1	-0.684807556	27.08059656	-2.971747967	0.034662686	0.111211447	-4.280337877
ASNS	-0.687963556	25.29290156	-2.541367053	0.056187943	0.16015066	-4.816530854
NASP	-0.689309333	24.63334133	-3.982034948	0.012525044	0.055016196	-3.125822063

UFM1	-0.689432111	25.30597239	-1.485354907	0.202775503	0.364210086	-6.161191527
NUCL	-0.698986444	27.01693244	-5.066964945	0.004949916	0.030238457	-2.06001366
PICAL	-0.699288111	23.57405606	-3.004170799	0.033463922	0.108592209	-4.240891693
DPY30	-0.699990222	23.17096856	-2.041430011	0.101857203	0.217812996	-5.45877141
EDF1	-0.701295222	24.88577372	-5.021012739	0.005132897	0.031000954	-2.101743783
DDB1	-0.701814333	24.25894317	-4.329808579	0.009143866	0.044306188	-2.765206047
PGP	-0.702155833	22.41094692	-1.679286959	0.159269879	0.302822541	-5.921470448
ACY1	-0.703438611	23.54452069	-1.381827404	0.230607061	0.399718906	-6.285089039
FHL3	-0.704956667	23.68178933	-3.624129196	0.017637961	0.068247065	-3.516653217
RL34	-0.706159333	25.52570944	-5.280245737	0.004195687	0.027608656	-1.869999133
HNRH1	-0.706632778	26.42579017	-3.283916139	0.024874946	0.087658486	-3.906845433
SPB9	-0.707351778	23.501065	-4.230830802	0.009983125	0.046778071	-2.865948441
DOPD	-0.709636833	23.67155158	-0.94565893	0.391486727	0.512684092	-6.756680032
PTPA	-0.710540556	23.68248383	-5.242369808	0.004319073	0.02801646	-1.903308301
RS15	-0.710892111	25.61331617	-1.950445918	0.113860467	0.235223367	-5.57600638
SYQ	-0.714134	23.66074678	-4.675999019	0.006795803	0.037535365	-2.424343054
PSA	-0.714162778	22.83332806	-2.796463571	0.042050399	0.128440473	-4.496034135
PURA2	-0.714493111	23.59968656	-3.336760415	0.023552928	0.084536772	-3.845058439
BACH	-0.715038778	23.84765694	-4.212551694	0.010147853	0.047135563	-2.884716614
RS8	-0.715535556	27.16417567	-6.655206018	0.001614037	0.016550457	-0.775741872
PSD12	-0.718246556	24.39200639	-5.721882281	0.003026574	0.02256111	-1.494911177
FAKD2	-0.718911389	7.757898083	-0.089727182	0.932293388	0.974815843	-7.246739298
NP1L4	-0.719234111	25.15517294	-1.118972446	0.318371283	0.471693937	-6.58109546
DIAP1	-0.720263444	25.07417861	-4.740599829	0.006440746	0.036041127	-2.362668446
ZPR1	-0.723066222	22.39280611	-2.511593688	0.058158772	0.16444894	-4.854370827
RL28	-0.726659	25.27416717	-4.562290774	0.007478621	0.038755487	-2.534359376
ERF1	-0.727263333	24.59589833	-5.622306385	0.003252121	0.02299771	-1.577395467
HSP74	-0.727687	26.61815594	-3.791324985	0.014995128	0.061355478	-3.331562068
RL3	-0.728369444	26.34749583	-3.122110498	0.029484824	0.099143957	-4.098658631
PROF1	-0.729779889	28.18924417	-2.327838259	0.072151954	0.188053749	-5.089446525
VTA1	-0.730804444	23.22425644	-4.09492229	0.011287644	0.051093963	-3.006721067
NDUB4	-0.731869056	23.13110847	-2.071023814	0.098249498	0.213307463	-5.420583642
ATX2L	-0.738335778	23.57849878	-0.984823728	0.373828264	0.498080559	-6.71863685
PSMD6	-0.739169111	23.379283	-4.573485687	0.007407909	0.038755487	-2.52344465
PSME2	-0.739689667	24.3181445	-2.811048938	0.041372022	0.127096759	-4.47793488
SEH1	-0.739987833	22.99996253	-3.746206356	0.015659982	0.063113575	-3.381074737
ACADS	-0.741208667	0.370604333	-1.059095442	0.342177464	0.476811995	-6.643802306
UBA6	-0.742474444	22.96316622	-3.066640837	0.031284861	0.103409805	-4.165305725
ARP3	-0.743302778	24.68838461	-4.638320054	0.007013573	0.037690357	-2.460591427
TIF1B	-0.746279667	26.61124006	-5.054018967	0.005000672	0.030461237	-2.071741404
GSHB	-0.748416556	24.3164425	-3.409447969	0.021864482	0.080094633	-3.760772817
AATC	-0.750751222	24.64551528	-3.22095459	0.026562868	0.091637597	-3.98101711
ACPH	-0.750785	23.37078394	-1.910960671	0.119527623	0.243765717	-5.626756106
HPRT	-0.752459833	24.35263797	-1.838654838	0.130686929	0.259668716	-5.719386735

SPB6	-0.753850444	23.94143833	-1.992964228	0.108074204	0.226117961	-5.521261737
SNRPA	-0.755760778	23.64160317	-3.07311399	0.031068465	0.103013946	-4.157505276
PABP1	-0.759367361	26.25883907	-5.978360207	0.002526378	0.020325424	-1.287798989
RL14	-0.761964667	26.04256989	-3.270613279	0.025221062	0.088149679	-3.922466741
RL9	-0.761996556	25.45091528	-3.920794228	0.013262497	0.05673143	-3.191266205
RL27	-0.764745667	26.26630261	-4.662716014	0.006871649	0.037690357	-2.437098414
DENR	-0.768208944	22.89470519	-2.005611841	0.106413821	0.223521444	-5.504961886
TCPE	-0.768297	26.3727815	-5.202360903	0.004454123	0.028437535	-1.938695557
RL10A	-0.769099556	26.42945667	-3.198946898	0.027183596	0.093175928	-4.007084243
JUPI2	-0.770296667	23.92714544	-4.791585599	0.006175944	0.035112299	-2.314410924
IF6	-0.772240889	25.111344	-4.267769301	0.009659731	0.045765661	-2.8281773
1433B	-0.772441667	27.64961961	-2.966789253	0.034850294	0.111395542	-4.286383554
RBM4	-0.773732389	23.58292069	-5.516738374	0.003513499	0.024056807	-1.66614969
EIF3J	-0.773882444	24.18483444	-3.204711425	0.027019414	0.092911922	-4.000249398
RAN	-0.774013556	27.028891	-2.032255508	0.103004177	0.218729985	-5.47060625
BT3L4	-0.782710222	24.38838211	-2.012016464	0.105583382	0.22287502	-5.496705556
PIN4	-0.796401667	22.3314195	-1.449602599	0.211996374	0.376646891	-6.204355012
SYDC	-0.797359444	25.36377661	-1.871011234	0.125564127	0.252311704	-5.677990305
OLA1	-0.798473667	25.41205017	-2.417712604	0.064892353	0.177145151	-4.974172621
RL11	-0.798849111	26.44858133	-2.430408191	0.063933326	0.175200322	-4.957931954
DD19A	-0.799400556	23.42626961	-2.727714532	0.04542142	0.135060623	-4.581694445
PLEC	-0.799922667	23.36908889	-4.440005391	0.00830529	0.041959429	-2.654783093
NMT1	-0.802092111	23.49669417	-4.075972989	0.01148499	0.051658224	-3.026574982
CPNS1	-0.802233333	24.86675978	-4.293457349	0.009442085	0.045034731	-2.8020326
DDX21	-0.805867111	24.574146	-2.405438911	0.06583461	0.178801769	-4.98988444
PCBP2	-0.806886667	26.49663333	-3.250399464	0.025757635	0.089584466	-3.946254937
1433T	-0.810443056	27.67132175	-3.020759521	0.032868873	0.107644296	-4.220766265
YBOX3	-0.815680556	25.3751505	-6.75048938	0.001520059	0.016043395	-0.707407119
UBP7	-0.815721278	22.99265147	-2.818281622	0.041040209	0.126259345	-4.468969538
PUR4	-0.818999444	23.83369717	-3.01602696	0.033037396	0.107956209	-4.226503866
TPD54	-0.821793111	24.06999344	-5.240407046	0.004325584	0.02801646	-1.905039475
TCPG	-0.823655333	26.65841844	-6.381725362	0.001925176	0.01800208	-0.976876305
IF5A1	-0.824472333	27.4840235	-4.851221368	0.005882395	0.033895315	-2.258430482
1433F	-0.827373	27.41446039	-2.22884377	0.081193089	0.198444023	-5.216902044
FEN1	-0.831690222	25.10274511	-1.038090078	0.350887752	0.480779362	-6.665309993
1433G	-0.832889	27.54284728	-2.559326293	0.055035094	0.15792035	-4.793744967
RS23	-0.837222889	25.14081833	-3.954956575	0.012845046	0.055661866	-3.154686566
PPM1G	-0.841497333	24.07177067	-5.674665209	0.003131115	0.02286143	-1.533876146
ICLN	-0.848698556	24.18159306	-5.852055209	0.002759234	0.021362816	-1.388846081
ABCF1	-0.849476778	23.94801872	-4.704872447	0.006634321	0.036930477	-2.396703846
KCRU	-0.855966333	24.78926317	-4.643681419	0.006982089	0.037690357	-2.455421142
DDX17	-0.855976556	25.88751506	-7.899670443	0.00077666	0.011113015	0.052527059
NCBP1	-0.857636667	23.19940856	-3.68778516	0.016572496	0.06494956	-3.445663315
TNPO1	-0.863315667	24.40712883	-2.93176994	0.036208528	0.114534988	-4.329174864

GNPI1	-0.864943667	23.61567806	-1.604955808	0.174714045	0.326378528	-6.014335199
EIF1B	-0.86585	25.25093644	-3.786497434	0.015064672	0.061355478	-3.336844409
COF1	-0.875695778	27.71586422	-1.837316578	0.130903447	0.259856749	-5.721096739
U5S1	-0.877578889	25.38709833	-8.487630827	0.000569097	0.00962948	0.400549306
GAPD1	-0.882658556	22.79388383	-3.410631338	0.021838172	0.080094633	-3.759407404
CYBP	-0.885541667	26.50778528	-6.340574507	0.001977997	0.018255797	-1.007801112
SH3G1	-0.885626444	23.06333789	-6.012376245	0.002467731	0.020240467	-1.260893473
CSN6	-0.8876905	22.47783886	-5.45784257	0.003670193	0.024684073	-1.716259665
HUWE1	-0.887906556	22.16210889	-2.415753993	0.065041714	0.177145151	-4.976679174
EF1G	-0.888513111	26.82485956	-6.769238226	0.001502348	0.01600909	-0.694064233
INO1	-0.888606333	25.08775761	-1.098094878	0.326501597	0.474639437	-6.60318824
SF3A1	-0.894129667	23.7943915	-2.699133385	0.046911444	0.137951999	-4.617469877
PP1G	-0.897324444	24.94909567	-3.112042305	0.029802569	0.09990421	-4.110722432
DDX1	-0.899898333	23.40899439	-3.149977527	0.028625364	0.097045197	-4.06534556
ITPA	-0.903502333	23.89960528	-1.378110547	0.231671958	0.401149812	-6.28947221
HNRPQ	-0.903544444	25.61682167	-7.064402621	0.001253665	0.014447638	-0.488363131
PRKDC	-0.906972556	24.89015994	-6.861945816	0.00141833	0.015427958	-0.628579949
QCR9	-0.907337111	25.44942467	-1.412455961	0.222009063	0.388786163	-6.248789875
MARE1	-0.908786333	24.6922965	-6.052077338	0.002401328	0.020235701	-1.229654439
GON7	-0.911063111	23.01240444	-6.863453324	0.001417011	0.015427958	-0.627521833
IF2P	-0.919050778	24.25894494	-3.49998998	0.019953041	0.074500673	-3.656933223
TPP2	-0.921599333	22.77670733	-7.672023564	0.000880897	0.011567564	-0.089198715
MCM4	-0.923504667	24.25036378	-2.190402817	0.085030241	0.200136416	-5.266485782
PSA3	-0.923872667	24.38902022	-4.401250527	0.00858945	0.042587693	-2.693410202
HNRPK	-0.926445556	27.84319933	-3.672683637	0.016818337	0.065431923	-3.462446798
MCM7	-0.927184667	24.65988044	-5.102326895	0.004814363	0.02965499	-2.028093725
PUR2	-0.9288265	25.57525642	-6.984120728	0.001316058	0.014721787	-0.543513174
VPS35	-0.931542444	24.13008822	-4.219384382	0.010085907	0.046950119	-2.877695135
PSA2	-0.934384444	24.80093144	-6.373404737	0.001935718	0.018021621	-0.983115054
DYN2	-0.935697778	23.66660611	-2.166065821	0.087560828	0.202034292	-5.297893252
NUDC	-0.943769667	25.75495106	-6.098179707	0.002326882	0.019843649	-1.193597293
MYL6	-0.944054111	24.09248361	-4.824081741	0.006013889	0.034559209	-2.283844841
SYSC	-0.954196556	23.59183394	-2.122126127	0.092338109	0.206277438	-5.354615401
SMD3	-0.955532889	24.64138633	-8.338199981	0.000614747	0.009851962	0.314461362
DNJB1	-0.956303778	24.47631533	-7.298044017	0.001091303	0.013144961	-0.331141304
ZYX	-0.957304222	23.35950878	-4.912156073	0.005599367	0.03270644	-2.201742687
CAZA2	-0.957626833	23.51279958	-1.823918412	0.133091848	0.260801305	-5.738206608
IDI1	-0.960689556	22.97131167	-1.541057864	0.189189055	0.347117956	-6.093234313
ANK1	-0.964469222	23.97204561	-3.852229683	0.014149114	0.059498838	-3.265235489
SYHC	-0.966074444	23.68271089	-5.113516108	0.004772392	0.029577733	-2.018028391
EM55	-0.968637	24.71137472	-5.200782122	0.004459554	0.028437535	-1.940096248
PFD4	-0.971199778	22.94917122	-3.744245697	0.015689643	0.063113808	-3.383233631
EHD1	-0.972909778	23.67202556	-4.001608883	0.012299526	0.05451682	-3.105028407
RS7	-0.976040222	26.10654322	-2.052286445	0.100517544	0.216030358	-5.444764316

RBM39	-0.980343	24.08563517	-4.474029954	0.008064909	0.041036722	-2.621054868
PSME3	-0.984254111	24.06579706	-7.014231474	0.001292229	0.014607995	-0.522759637
FKBP4	-0.986752333	25.2193605	-3.932069832	0.013122984	0.056521622	-3.179172513
HNRPF	-0.988919333	25.92805978	-5.697264214	0.003080551	0.022690954	-1.515193712
XRCC5	-0.990607556	25.879337	-5.148006106	0.004645728	0.028961091	-1.987107279
LRC47	-0.992470444	23.54715056	-5.531520077	0.003475432	0.024056807	-1.653640299
CPIN1	-0.996742778	23.39944806	-5.28738295	0.004172909	0.02754378	-1.863743208
CT45A	-0.9974775	23.05286169	-4.315626648	0.009258875	0.044559642	-2.779549642
XPO1	-1.000467	24.85043961	-2.341995541	0.070951493	0.186419365	-5.071256112
API5	-1.001937889	23.53493839	-3.396038301	0.022165192	0.080918134	-3.77626058
CLIC4	-1.005180444	24.37958989	-3.903332018	0.013481991	0.057258177	-3.210034756
RENT1	-1.007394722	23.30371297	-4.098207729	0.011253833	0.051049304	-3.003284446
EIF3L	-1.015185778	24.07912322	-6.486920793	0.001797644	0.017341981	-0.898615654
ENOG	-1.020133889	27.50622372	-3.969684153	0.012669831	0.055425249	-3.138973301
MK01	-1.033687444	24.85769939	-3.69794947	0.01640938	0.064786662	-3.434387228
PSDE	-1.034849222	23.97265872	-3.451426605	0.020953117	0.077421223	-3.712469699
NRDC	-1.036161111	22.66575289	-7.260663201	0.0011155	0.013302353	-0.355973482
TCPZ	-1.036666722	26.13914025	-5.475995989	0.003621023	0.024508006	-1.700768469
PUS7	-1.0396065	22.92160236	-1.500433546	0.199005486	0.359559064	-6.142877613
SNX2	-1.040561111	22.58773956	-6.177329287	0.002205406	0.019429447	-1.132235804
RL21	-1.043646778	25.85653972	-5.495635927	0.003568709	0.024230851	-1.684054808
RS15A	-1.059337111	25.67145944	-7.318971114	0.001078032	0.013144961	-0.317292062
TSN	-1.062090222	23.80995733	-2.510381606	0.058240615	0.164462239	-4.855912969
ALDOC	-1.063266333	28.13126972	-4.763861411	0.0063183	0.03563655	-2.340605987
ANM1	-1.063403778	24.95004822	-7.913939414	0.000770636	0.011101323	0.06127605
RS24	-1.067859556	25.702273	-5.250529433	0.004292131	0.02801646	-1.896116803
PRS8	-1.083163556	23.92764556	-4.689697482	0.006718618	0.037205439	-2.411215262
LRRF1	-1.084395	22.40475139	-3.354518557	0.023126957	0.083570631	-3.824391402
PSA4	-1.085247	26.0941465	-2.227166818	0.081356503	0.198444023	-5.219064283
RLA1	-1.092291611	27.60617719	-3.435065013	0.021303014	0.078442186	-3.731263655
RS3	-1.117375667	26.85177172	-4.958287426	0.005395794	0.032044104	-2.159167373
PRS10	-1.120429222	24.4169685	-10.09351585	0.000266314	0.006838858	1.236387664
DJC11	-1.123358333	22.5028885	-2.799788158	0.041894673	0.128332532	-4.491906271
ADRM1	-1.1335445	23.30032219	-2.038948757	0.102166053	0.217851324	-5.461972366
GTPB1	-1.136645556	22.35671589	-1.661747687	0.16278476	0.308221234	-5.943477181
EF2	-1.137786	28.33320967	-7.23632312	0.001131603	0.013355696	-0.37220788
PSD13	-1.138296889	24.58070156	-3.503153498	0.019889833	0.074500673	-3.653328282
HMCS1	-1.138932222	25.70585667	-5.796962957	0.002868788	0.021766035	-1.433494121
TRIP6	-1.140541	23.38093561	-9.348620382	0.000373167	0.007712787	0.867826246
IPO5	-1.144182111	24.06142917	-9.987018271	0.000279072	0.006838858	1.185556527
DCTN2	-1.144189	23.09809506	-2.681867932	0.047838083	0.140289948	-4.63912621
GRN	-1.14894	23.49577606	-1.958948402	0.112677668	0.233913133	-5.565066132
FLNC	-1.149851222	24.48653194	-8.918887615	0.000458536	0.008427578	0.640514925
RS3A	-1.150768111	26.6019895	-6.028145263	0.002441097	0.020240467	-1.248464561

PPIA	-1.153726111	28.70999972	-9.887416089	0.000291679	0.00689654	1.137471026
BAG2	-1.154008667	23.41467856	-6.68341672	0.001585504	0.016489241	-0.755417942
GUAA	-1.157710333	24.5636795	-8.814844127	0.000482642	0.008504064	0.583742361
IMDH2	-1.165435389	24.24280886	-8.942531497	0.000453263	0.008403094	0.65331977
ARF5	-1.167507667	25.15681483	-3.919539004	0.013278135	0.05673143	-3.192613734
HAT1	-1.179179111	24.22902656	-7.661032974	0.000886343	0.011567564	-0.096144896
LRBA	-1.184075111	22.41955378	-2.768244001	0.043398813	0.131803802	-4.531126593
UB2L3	-1.186249333	27.50585244	-2.903498269	0.037348958	0.117352244	-4.363841817
ARC1B	-1.203958167	22.70360358	-4.476544422	0.008047473	0.041036722	-2.618569109
PSD11	-1.204394	24.50365356	-5.71705305	0.003037073	0.02256111	-1.498884264
RL24	-1.210323333	25.52678089	-2.980008897	0.034352685	0.110634327	-4.270273653
PUF60	-1.212941	25.19577672	-5.376276352	0.003901177	0.025910622	-1.786372705
BAG6	-1.215752222	23.508341	-2.921105785	0.036633999	0.115537998	-4.342238765
RS6	-1.215993444	26.61122694	-3.904790548	0.013463496	0.057258177	-3.208465281
UBP14	-1.216320778	24.19020994	-8.876833893	0.0004681	0.008457539	0.617651654
RS12	-1.216899222	26.55766994	-7.677687802	0.000878106	0.011567564	-0.085622637
TIM13	-1.232705778	23.95308822	-4.774892273	0.006261199	0.035502331	-2.330170535
MIF	-1.233250667	27.37663578	-3.082428714	0.030760066	0.10246947	-4.146291179
CAN1	-1.235727222	24.37302361	-5.099231869	0.004826049	0.02965499	-2.030880822
ANM5	-1.241616667	23.952222	-3.907965583	0.013423338	0.057237112	-3.205049887
RS14	-1.251605667	26.70975217	-4.797908481	0.006144011	0.035024149	-2.308451955
RS2	-1.253373778	26.32548478	-8.281923776	0.000633076	0.009851962	0.281632808
TBAL3	-1.261659889	26.85720972	-3.72423618	0.015996116	0.063984465	-3.405300976
PAIRB	-1.266459889	25.96562983	-6.519835446	0.001759838	0.017148718	-0.874360155
RL22	-1.269086778	26.86722106	-3.781819838	0.015132415	0.061452018	-3.341966179
DDX5	-1.270196667	25.93151189	-10.67325985	0.000208037	0.005993728	1.502962643
DX39B	-1.271067389	25.87879619	-3.27407606	0.025130437	0.087994518	-3.918397866
RS11	-1.274767	25.38840706	-11.54197062	0.000146967	0.005222238	1.872842181
MYH9	-1.275368222	23.61892556	-5.923959393	0.002623672	0.020640846	-1.331098635
RL23A	-1.276509	26.03712083	-2.269514561	0.077337194	0.195590625	-5.164489954
ATOX1	-1.289736389	23.33415169	-3.811957955	0.014702079	0.060863751	-3.309026774
RL38	-1.294751444	22.96239128	-2.549457304	0.055665317	0.158919232	-4.806262582
EIF3M	-1.300920667	24.00503989	-6.518349837	0.001761524	0.017148718	-0.875452576
RL8	-1.302353444	25.74826717	-4.6608504	0.006882382	0.037690357	-2.438891951
SGT1	-1.303447333	23.53944922	-8.108486488	0.000693862	0.01041771	0.179023152
GLRX3	-1.305685056	24.38302392	-3.107315921	0.029953079	0.100093988	-4.116390729
BASI	-1.307600667	23.78400233	-3.61291394	0.017833817	0.068879886	-3.52922691
RS9	-1.312202	25.79932589	-10.89193672	0.000190149	0.005884492	1.599273701
GEMI5	-1.314195556	23.294516	-6.508340906	0.001772932	0.01718132	-0.88281829
DX39A	-1.324299611	25.86517319	-3.278882656	0.025005262	0.087972307	-3.912752991
CDV3	-1.324896889	24.29266178	-3.785446314	0.015079864	0.061355478	-3.33799504
ACBP	-1.326527667	25.72583017	-1.18183249	0.294968093	0.460998859	-6.513183984
SYK	-1.333497889	25.07047817	-5.980581509	0.002522497	0.020325424	-1.28603806
IF4A1	-1.335077667	27.20137861	-7.618046259	0.000908035	0.011592405	-0.123406474

CHRD1	-1.336105889	24.62384994	-4.878735505	0.005752548	0.033327264	-2.232770217
RS26	-1.338314778	25.80456978	-9.876746452	0.00029307	0.00689654	1.132288346
PFD5	-1.340862222	23.315096	-5.805741236	0.002850989	0.021708241	-1.426356424
EIF3G	-1.341086778	24.57602306	-9.265327809	0.000388103	0.007712787	0.824647319
GTSF1	-1.341334778	23.3891895	-8.334841348	0.000615823	0.009851962	0.312508429
TCPD	-1.344198889	26.03656578	-3.10010929	0.030184246	0.100708625	-4.125039814
IF4A3	-1.344443778	25.85676133	-10.31295116	0.000242181	0.006619607	1.339266539
EF1D	-1.353225444	26.40074328	-4.443584985	0.008279607	0.041929031	-2.651226585
TRAP1	-1.360494111	28.6768415	-2.064658685	0.099013757	0.21389944	-5.428798559
HNRPM	-1.362496444	25.23993833	-12.69252898	9.62E-05	0.00461475	2.314779201
PGAM4	-1.364274667	26.52048444	-3.676440305	0.016756787	0.065431264	-3.458268357
RS27	-1.366925444	25.25362639	-3.263868476	0.025398662	0.088625117	-3.930397325
DNJA1	-1.372012222	24.94951878	-7.956486696	0.000753005	0.011003882	0.087271375
G3BP1	-1.373886333	25.19349739	-9.082440652	0.000423514	0.008061889	0.72836888
SF3B2	-1.376265278	23.48863314	-6.134383677	0.002270349	0.019517681	-1.16544524
AIMP2	-1.377516389	24.14284492	-1.643820751	0.166458706	0.31461876	-5.965912312
STRAP	-1.382526222	24.210383	-5.960968412	0.002557006	0.0204945	-1.301605451
METK2	-1.392858722	25.36310142	-4.276233472	0.009587372	0.045524002	-2.819551592
SAE1	-1.397000667	23.66760989	-4.517092291	0.007772337	0.040025657	-2.578613132
NAA15	-1.397689444	24.04296494	-9.10687247	0.000418564	0.008039443	0.741349187
RIR1	-1.400179	23.7185975	-6.641768571	0.001627845	0.016550457	-0.78545005
RS13	-1.426469778	25.56841878	-5.164722462	0.004585777	0.028755518	-1.972177279
DDX46	-1.436760778	22.67855356	-5.010913687	0.005174181	0.031162015	-2.110953134
PSMD4	-1.440271444	23.98208806	-5.531711296	0.003474943	0.024056807	-1.653478651
STXB2	-1.444995889	23.39617994	-1.181322118	0.295151709	0.460998859	-6.513743446
RL13A	-1.446140333	25.38339494	-8.085262252	0.000702526	0.010440962	0.165115977
PUR8	-1.458382389	22.73328625	-4.235392945	0.009942503	0.046690343	-2.861272199
CND1	-1.463333111	22.48496389	-4.491212404	0.007946638	0.040726518	-2.604087234
UBAC1	-1.467649778	23.36709733	-4.589481298	0.007308238	0.038567237	-2.507881086
RS4X	-1.475276222	26.72928233	-11.13948342	0.000172095	0.005635059	1.705646355
KCY	-1.479431389	23.99345142	-2.765824132	0.04351669	0.131973803	-4.534140325
DCTN1	-1.481043389	22.44716108	-8.21222836	0.000656699	0.010145523	0.240661878
CNBP	-1.488413111	24.89251311	-8.605601199	0.000535924	0.009140728	0.467423868
SRP72	-1.492255444	23.68937661	-7.858888828	0.000794193	0.011213372	0.027435501
RS5	-1.507420111	26.4996265	-2.328311681	0.072111453	0.188053749	-5.088838061
RS17	-1.508878778	26.2125975	-9.27336539	0.000386631	0.007712787	0.828832066
RS19	-1.521399556	25.88146011	-10.84655676	0.000193703	0.005884492	1.579470705
SRSF1	-1.524557222	25.46152861	-4.475789887	0.0080527	0.041036722	-2.619314931
UBP2L	-1.532606556	23.71136039	-6.009923354	0.002471905	0.020240467	-1.262829298
RL26L	-1.535180111	24.41612372	-2.749261784	0.04433322	0.133312307	-4.554786105
RBP2	-1.537847333	22.88525611	-4.385668474	0.008706919	0.042871021	-2.709004119
LARP1	-1.542921333	23.75343244	-4.597912449	0.00725634	0.038388381	-2.499692651
EIF3B	-1.543736778	25.06231917	-11.29962572	0.000161517	0.005554115	1.773006901
CAPR1	-1.545234444	24.89405678	-4.75199472	0.006380417	0.03589195	-2.351851373

JUPI1	-1.558854222	25.31361011	-4.697136156	0.006677139	0.037072032	-2.40409774
PPID	-1.562757611	23.79499386	-4.925343917	0.005540255	0.032450064	-2.189541575
HS105	-1.563527889	25.56304728	-4.290802923	0.009464306	0.045039955	-2.804729572
TMOD3	-1.569803667	8.048405722	-0.173828394	0.869417779	0.918697735	-7.233359839
PRS6B	-1.591243778	23.33233844	-3.070396307	0.031159108	0.103154066	-4.160779477
BIEA	-1.618356722	15.93406886	-0.181934455	0.863407667	0.913541016	-7.23162385
DPYL2	-1.621093722	15.39224508	-0.190862584	0.85680046	0.908805264	-7.229621613
TP4A2	-1.633806722	8.323476694	-0.173617715	0.869574123	0.918697735	-7.233403918
EIF3C	-1.636110778	23.54438828	-11.90980556	0.000127797	0.005140803	2.01971718
CLIC2	-1.641415111	24.59631389	-6.479366844	0.001806458	0.017348505	-0.904197785
IF4G1	-1.641825056	24.47394031	-9.632719005	0.000327188	0.00740399	1.01204974
EIF3D	-1.642709444	24.28261161	-7.232951479	0.001133856	0.013355696	-0.374460777
RS25	-1.656275222	25.78691683	-3.828224371	0.014475747	0.060395874	-3.291307971
AHSA1	-1.658620222	25.34881633	-6.303361148	0.002027265	0.018519974	-1.035919079
TEBP	-1.665872222	25.462155	-4.639735416	0.007005245	0.037690357	-2.459226106
EIF3F	-1.667597667	23.87025861	-9.36820729	0.000369756	0.007712787	0.877920273
ZCCHV	-1.671654889	22.63020511	-4.164962094	0.010591918	0.048459162	-2.933819646
PACN2	-1.692943444	15.51990372	-0.197116836	0.8521801	0.905472528	-7.228162816
VP33B	-1.694649889	8.103617722	-0.18548477	0.860778689	0.912117378	-7.230838963
IPO4	-1.7102205	22.83611297	-5.738393468	0.002991003	0.022374805	-1.481347972
NEDD4	-1.722078667	0.861039333	-1.068054282	0.338519674	0.474818385	-6.634549912
CNDP2	-1.742092889	24.46558356	-3.075206009	0.030998895	0.102944773	-4.154985583
APT	-1.754265667	24.54032639	-8.838145262	0.000477113	0.008504064	0.596517549
NOSIP	-1.755408889	23.08850244	-6.407314946	0.00189318	0.017938937	-0.957734276
RL10	-1.760504333	25.99499183	-8.136606045	0.000683544	0.0104094	0.195808433
CDK1	-1.780105778	24.42756344	-7.540321465	0.000948905	0.011970799	-0.173079634
RM41	-1.786225611	8.512284139	-0.185852915	0.860506199	0.912117378	-7.230756721
EIF3A	-1.796243222	24.26939939	-7.710848795	0.000861979	0.011567564	-0.064738174
BUD31	-1.800696778	15.61761106	-0.208585713	0.843725423	0.898628019	-7.225367668
CCAR2	-1.807505222	23.30855706	-9.157811125	0.000408466	0.007933977	0.768293971
IPO7	-1.810641111	23.926686	-3.980647237	0.012541215	0.055016196	-3.127298521
RAGP1	-1.862549111	23.76621522	-8.120774442	0.000689331	0.01041771	0.186365313
PCY1A	-1.862603222	8.400178944	-0.196840208	0.852384317	0.905472528	-7.228228317
CDC42	-1.867373222	24.19537239	-2.461394501	0.061657659	0.170941649	-4.918343388
NAMPT	-1.875660889	23.96457244	-6.020844675	0.002453385	0.020240467	-1.254215337
ODO2	-1.881773778	22.96854744	-4.203716474	0.010228618	0.04732612	-2.89380655
FLNB	-1.891109944	23.71349581	-6.542034145	0.001734879	0.017123894	-0.85806311
KPYR	-1.895640389	25.54768947	-1.427779113	0.217824434	0.384438489	-6.23051321
PYR1	-1.901839333	23.42688033	-15.17920629	4.31E-05	0.003111281	3.11803465
EIF3I	-1.937105667	24.55990983	-5.531649638	0.0034751	0.024056807	-1.653530773
GPDM	-1.948110889	16.21232856	-0.214979632	0.839022428	0.89574152	-7.223742058
TCTP	-1.954165778	24.62661289	-7.698941714	0.000867728	0.011567564	-0.072227028
ABCE1	-1.992409333	24.50136844	-5.697517432	0.00307999	0.022690954	-1.514984723
KAD1	-2.014204222	24.37649178	-1.981603137	0.109589155	0.228819864	-5.535898053

MSMO1	-2.022746944	16.26635819	-0.252440806	0.811631596	0.87129837	-7.213254713
GIPC1	-2.056262028	20.30613063	-0.951313419	0.388895459	0.510090072	-6.751250767
ERF3A	-2.070668167	23.98233903	-6.104590225	0.00231675	0.019836592	-1.188602062
AIP	-2.142841444	9.395896222	-0.291820095	0.783172591	0.845858138	-7.200472023
EIF3E	-2.162891333	23.25749122	-4.203209609	0.010233275	0.04732612	-2.89432839
DDX3X	-2.166288222	24.20921344	-17.59623557	2.21E-05	0.001884997	3.744351961
RS16	-2.174770111	25.95783894	-8.387991943	0.000599061	0.009851962	0.343320306
HS74L	-2.324013111	25.18888	-3.298456413	0.024502863	0.086777581	-3.889801911
UAP1	-2.343037778	23.61030522	-5.272448566	0.004220741	0.027688061	-1.876841029
RNT2	-2.416128722	20.91249231	-1.048046456	0.346735645	0.478472747	-6.655148315
IMA1	-2.493309556	25.05742522	-14.72879287	4.93E-05	0.003286451	2.985899613
BTF3	-2.547673556	24.85079967	-19.15065542	1.51E-05	0.001529492	4.084690399
RS18	-2.584093444	25.54854339	-17.74674528	2.13E-05	0.001884997	3.779244734
EI2BD	-2.5880965	20.77689781	-1.175769687	0.297155985	0.461761342	-6.519821629
DHX30	-2.592942889	15.4527185	-0.305363145	0.773472648	0.837928702	-7.195663753
RPIA	-2.690975389	6.301318639	-0.31599901	0.765888323	0.832249697	-7.191740953
DNM1L	-2.6959855	8.581380528	-0.372640552	0.726028975	0.797883389	-7.168700832
RUXE	-2.774614167	24.72893303	-5.171774563	0.004560761	0.028683017	-1.965889833
NB5R1	-2.809862667	16.37105944	-0.351300655	0.740937164	0.810506944	-7.177803291
HMOX2	-2.935319944	21.26747058	-1.259913451	0.2680736	0.442020816	-6.426145245
RDH10	-2.939465	24.1081875	-1.590757489	0.177831623	0.330258729	-6.031946893
CLN6	-2.9719075	8.993467306	-0.323683239	0.760427604	0.827159006	-7.188826845
RL23	-3.004692778	25.54732728	-5.636821672	0.003218025	0.02299771	-1.565297756
HGS	-3.039057556	9.138700111	-0.324105684	0.760127863	0.827159006	-7.1886647
DUT	-3.085669667	24.04589828	-4.934200943	0.00550097	0.032308727	-2.18136068
MAP11	-3.087598167	9.116246083	-0.331185569	0.75511173	0.823477344	-7.185917241
S38A1	-3.104599667	1.552299833	-1.06948541	0.337938513	0.474639437	-6.63306756
UBR2	-3.130977444	1.565488722	-1.069496103	0.337934174	0.474639437	-6.63305648
SLK	-3.148222667	20.43338722	-0.715266862	0.509224283	0.607195845	-6.957710164
DBNL	-3.162624889	21.38266489	-1.342351617	0.242159869	0.412368084	-6.331389167
ABCA4	-3.165209222	1.582604611	-1.069509583	0.337928704	0.474639437	-6.633042511
PLCB3	-3.178743778	4.780099667	-0.75432293	0.487593578	0.591491075	-6.926607645
ASPC1	-3.179555222	1.589777611	-1.069515104	0.337926464	0.474639437	-6.63303679
RT22	-3.234105944	16.69839147	-0.395770507	0.710027993	0.783124512	-7.158265759
ECM1	-3.270476278	20.30349658	-0.744648449	0.49288858	0.59470201	-6.934432973
WDR5	-3.284724444	16.07538267	-0.423422042	0.691124883	0.765045821	-7.145023238
SYMPK	-3.291987667	21.4522015	-1.387096438	0.229105495	0.398087136	-6.278867206
GCN1	-3.352160833	21.50466069	-1.445522573	0.213074319	0.376954088	-6.209256636
TIMP1	-3.3595925	1.67979625	-1.163153871	0.301755681	0.465602573	-6.533574977
ZFR	-3.3943985	16.04397614	-0.43994444	0.67995208	0.754637082	-7.136716324
DNJC7	-3.394946889	21.16022411	-1.51289756	0.195941347	0.355432045	-6.127692445
RS10	-3.582291722	24.36708269	-5.643115515	0.003203373	0.022995256	-1.560060054
VIGLN	-3.675854722	21.39517553	-1.593009001	0.177333552	0.329908494	-6.029157024
MTCH1	-3.684318944	16.26287097	-0.469622023	0.660122445	0.738395096	-7.12106503

SC31A	-3.788899056	21.61964769	-1.626941185	0.169994669	0.31960197	-5.986980419
T2FB	-3.912129222	5.087042056	-0.543101677	0.612422879	0.695833864	-7.0783723
F120A	-3.936616111	9.121406944	-0.455990134	0.669192088	0.745801114	-7.128369969
MSI2H	-4.030490111	5.201215889	-0.54671325	0.610132004	0.694501566	-7.076132466
MSH6	-4.044676833	21.17989981	-2.00950101	0.105908717	0.223119944	-5.49994845
SMRC1	-4.322825	17.44681039	-0.57540618	0.592116717	0.683113008	-7.057878038
GCP60	-4.3648215	16.98927169	-0.603669493	0.574698393	0.669173662	-7.039110503
FIS1	-4.494367333	5.41547	-0.579984147	0.589273125	0.680937834	-7.05489069
AP3B1	-4.555440889	13.00205811	-0.484052669	0.650594656	0.731031708	-7.113119582
GALM	-4.566793667	16.80847233	-0.645968684	0.549252446	0.64625067	-7.0096011
MOV10	-4.8674655	12.84763603	-0.722961992	0.50490898	0.603738613	-6.951685535
DNLI1	-4.994890556	5.639233944	-1.554913814	0.185951896	0.343347892	-6.076206445
RUFY1	-5.031955111	9.731022833	-0.554777808	0.605035213	0.691283534	-7.07108405
KAP3	-5.339073111	17.26576961	-0.739427248	0.495763527	0.595644216	-6.938623355
FAS	-5.364281111	25.30503633	-15.12432604	4.38E-05	0.003111281	3.102219733
ARMC1	-5.729650556	10.13854278	-0.605261046	0.573727353	0.668408042	-7.038030799
F1142	-5.751560222	6.027435667	-0.865944147	0.429532385	0.550338368	-6.830835239
NU160	-5.891775944	15.20189608	-0.809305229	0.458295091	0.577762751	-6.880666475
EMAL4	-5.981791944	19.89601597	-0.754248662	0.487634068	0.591491075	-6.926668018
THIC	-6.224634833	22.34680269	-2.999329946	0.033639871	0.108831872	-4.246771879
GTF2I	-6.346341778	19.5683	-0.815562437	0.455046698	0.574477032	-6.875283651
MPC2	-6.349015556	11.50336111	-0.611923112	0.569674147	0.665290361	-7.033485054
COQ9	-6.444346333	19.73957283	-0.83489314	0.445122388	0.565554786	-6.858460217
GBF1	-6.6964015	18.03205214	-0.761431987	0.483729256	0.589609205	-6.920807346
CTBL1	-6.737026556	10.85181356	-0.698374723	0.518788581	0.615844796	-6.970754146
OXSR1	-6.743079111	10.63283139	-0.83067221	0.447275068	0.566938433	-6.862158504
SFR15	-6.7663405	18.40044308	-0.963887342	0.383183897	0.505087672	-6.739100462
ISG15	-6.767147778	10.79818544	-0.705767793	0.514587204	0.611879486	-6.965076058
MYCBP	-6.86283	18.63285222	-0.965739116	0.382348642	0.504435214	-6.737302149
TRFL	-6.865333556	19.30191378	-0.92579491	0.400702149	0.519961644	-6.775579422
SC11A	-6.883095778	19.41280456	-0.922290713	0.402346032	0.521777214	-6.778885107
ARI1	-6.917973333	10.851621	-0.719512022	0.506840434	0.605369079	-6.954392912
QCR10	-6.926547667	18.61248783	-0.977043937	0.377282261	0.499642453	-6.726274532
RAB21	-6.927449444	18.69210306	-0.972755398	0.379197602	0.500897948	-6.730467823
ABHEB	-6.953587889	19.95484306	-0.898956341	0.413432226	0.533880984	-6.800676783
RT18A	-6.999914556	18.71506372	-0.984372974	0.374027662	0.498080559	-6.719080445
MESD	-7.025860722	19.42923725	-0.943769349	0.392355816	0.513191779	-6.758489507
SPT6H	-7.081966222	11.32502622	-0.703700343	0.51575969	0.61259034	-6.966668763
PP2BA	-7.097611111	18.42378922	-1.020676233	0.358252225	0.485046498	-6.682938341
TM256	-7.147183722	3.573591861	-1.070002827	0.33772861	0.474639437	-6.632531331
AMOL2	-7.188577	3.5942885	-1.070004209	0.337728049	0.474639437	-6.632529898
SNX5	-7.193565889	11.32471606	-0.716472708	0.508546338	0.607051315	-6.956769483
TPMT	-7.200784889	11.0515555	-0.737386535	0.496890494	0.596188935	-6.940254876
MMPOS	-7.213171167	11.09717503	-0.735341068	0.498021942	0.597178167	-6.941886639

IMPA1	-7.220089111	18.441531	-1.041204833	0.349584242	0.48017164	-6.662137417
RDH13	-7.229157222	11.00846739	-0.743791999	0.493359336	0.594781464	-6.935121919
EPS15	-7.247895444	3.623947722	-1.07000615	0.337727262	0.474639437	-6.632527887
EXOS6	-7.260201111	18.64650611	-1.034442706	0.352419453	0.481450779	-6.669017616
MXRA7	-7.261011556	19.56875589	-0.974831802	0.378269233	0.500272867	-6.728439043
LTOR3	-7.279154333	19.24224961	-0.997294473	0.368346888	0.493598721	-6.706312232
CHM4A	-7.283051278	18.74612247	-1.031682993	0.353582183	0.481993104	-6.671817547
C1TM	-7.293811111	19.34993633	-0.993830101	0.369862799	0.494829776	-6.709746029
NCBP2	-7.301241444	11.09459244	-0.745689187	0.492316968	0.59470201	-6.933594947
RT30	-7.306996778	3.653498389	-1.070008037	0.337726497	0.474639437	-6.632525931
CBPD	-7.328914278	3.664457139	-1.070008725	0.337726218	0.474639437	-6.632525218
NUP37	-7.335438278	18.80562669	-1.035149991	0.352121984	0.481450779	-6.668299278
GOGA2	-7.3371055	10.81002708	-0.771998302	0.47802733	0.586645509	-6.912108613
PDCD6	-7.354920111	18.8683975	-1.034405569	0.352435077	0.481450779	-6.669055325
ULA1	-7.3567165	18.51802436	-1.060579471	0.341569198	0.47627569	-6.642272894
SPF27	-7.3629085	10.96008114	-0.763047999	0.482853973	0.589264265	-6.919482957
WDHD1	-7.366175944	11.08949436	-0.753563205	0.48800788	0.591491075	-6.927225007
CSTF3	-7.3727335	18.32834253	-1.077410653	0.33473585	0.474639437	-6.624837235
DIP2B	-7.388948389	10.99013119	-0.763821136	0.48243563	0.589264265	-6.918848573
CIA2B	-7.407058944	11.29034258	-0.742782418	0.49391468	0.594929999	-6.935933249
FA98B	-7.410782556	18.40572378	-1.078802327	0.334176197	0.474639437	-6.623388262
AR6P1	-7.414729556	11.76128178	-0.710551983	0.511881213	0.610022776	-6.96137609
SNX1	-7.424493889	11.13616044	-0.756557727	0.486376364	0.590857213	-6.924788819
ACTY	-7.429121667	18.95781556	-1.040763812	0.349768554	0.48017164	-6.66258698
FAF1	-7.429901111	19.15853122	-1.027961917	0.355155148	0.482594503	-6.675585495
VATC1	-7.431944167	18.90484303	-1.045453918	0.347812724	0.479338544	-6.657800038
ECM29	-7.441000556	18.67803161	-1.064085901	0.340135717	0.475655674	-6.638654107
RFA1	-7.443558778	18.69776794	-1.063355575	0.340433855	0.475655674	-6.639408428
SPART	-7.446834444	18.78874144	-1.049768716	0.346021713	0.478384526	-6.653384521
CPSF7	-7.449036	11.42962489	-0.73733799	0.496917325	0.596188935	-6.940293644
CSN2	-7.455910222	19.10581456	-1.035656398	0.351909133	0.481450779	-6.66778477
DNJC5	-7.467367278	18.63618519	-1.072108683	0.336875487	0.474639437	-6.630347314
SSRP1	-7.477153333	19.16448889	-1.034234931	0.352506879	0.481450779	-6.66922858
RPTOR	-7.48445	3.742225	-1.070013435	0.337724308	0.474639437	-6.632520335
CDK5	-7.487920389	11.08696992	-0.767759922	0.480308506	0.588177906	-6.915608967
SART3	-7.506660611	18.48017469	-1.089239381	0.330004959	0.474639437	-6.612486339
SF3B6	-7.510076778	18.82113928	-1.062443754	0.340806401	0.475834478	-6.640349769
MK14	-7.517951778	10.78938383	-0.794743332	0.465922867	0.581598926	-6.893072902
EXOS3	-7.518823944	18.81573519	-1.068612398	0.338292928	0.474812721	-6.633971961
MOT1	-7.524812444	19.39703333	-1.029916308	0.354328249	0.482068706	-6.673607541
MARE2	-7.530195944	18.74504986	-1.071142451	0.337266694	0.474639437	-6.631349727
DPOD2	-7.536640222	10.84085261	-0.792946282	0.466870788	0.582086854	-6.894592166
OPLA	-7.547615889	11.27280183	-0.760319429	0.484332529	0.589609205	-6.921717869
TRM1L	-7.5514405	18.66577736	-1.084828366	0.331762268	0.474639437	-6.617101364

SH3L1	-7.563069333	19.19335822	-1.05015867	0.345860241	0.478384526	-6.652984917
MK67I	-7.569449389	11.22138658	-0.766420508	0.481031073	0.588503797	-6.916712062
DDAH2	-7.579686667	19.20141894	-1.050209816	0.345839067	0.478384526	-6.652932499
LACB2	-7.582664111	11.3101285	-0.761094518	0.483912186	0.589609205	-6.921083641
NDUF2	-7.592061667	19.16481594	-1.055898524	0.343490982	0.477394246	-6.647092589
PLAP	-7.603351889	11.05464289	-0.782767989	0.472267031	0.584943733	-6.90314781
RBM12	-7.614885444	18.62699411	-1.099066661	0.326119139	0.474639437	-6.60216522
8ODP	-7.620171111	11.03787972	-0.786931209	0.470054198	0.583948926	-6.899658457
RT06	-7.644136	18.91757756	-1.083586793	0.332258372	0.474639437	-6.618398373
P5CR3	-7.651681389	18.22135425	-1.13626476	0.311773323	0.467680797	-6.562618086
NUP50	-7.652881778	18.27509861	-1.133295849	0.31289741	0.467680797	-6.565801766
PDS5A	-7.663758694	18.22305304	-1.138811895	0.310811796	0.467680797	-6.559882993
FIP1	-7.666888722	18.91907764	-1.087278938	0.330784975	0.474639437	-6.614538811
LARP7	-7.671799611	11.00713492	-1.144668324	0.308611056	0.467680797	-6.553581523
ARPC2	-7.681691611	18.66827058	-1.108594274	0.322390244	0.473046111	-6.59210758
COPD	-7.717963722	19.13533353	-1.076653581	0.335040644	0.474639437	-6.625625011
MD2L1	-7.721965722	18.69812731	-1.111385167	0.321305087	0.472760864	-6.589151974
NUDT4	-7.741864944	18.77995619	-1.110979345	0.321462678	0.472760864	-6.589582011
VPS25	-7.752333	3.8761665	-1.070020894	0.337721283	0.474639437	-6.632512605
SPTA1	-7.773744722	18.70057625	-1.121789477	0.317288058	0.470743312	-6.578096279
TI17A	-7.775182278	19.48561197	-1.065636785	0.339503356	0.475655674	-6.637051237
PSMF1	-7.801148111	19.30198417	-1.083638512	0.332237694	0.474639437	-6.618344363
ENOPH	-7.805485889	18.90967594	-1.112972913	0.320689178	0.472680539	-6.587468622
PFKAL	-7.806585667	19.75753994	-1.053561823	0.344453815	0.478085724	-6.649493718
PUR1	-7.810337333	18.61628411	-1.135260792	0.312153041	0.467680797	-6.5636952
AGM1	-7.825245333	11.45597456	-0.777626578	0.475010502	0.585192204	-6.907437521
ATG3	-7.8305365	18.66468436	-1.136012719	0.31186861	0.467680797	-6.562888539
PITH1	-7.843263778	11.74912211	-0.757791459	0.485705349	0.590378451	-6.923782937
AGFG1	-7.845332167	18.58551919	-1.144777645	0.308570108	0.467680797	-6.553463724
GALK1	-7.860079444	18.79163617	-1.13090992	0.313803381	0.468507569	-6.568356915
TE2IP	-7.869732778	19.34903317	-1.092468994	0.328723479	0.474639437	-6.609100399
RPAP3	-7.869774389	11.19413147	-0.803226166	0.461467838	0.579365403	-6.885866306
ARPC3	-7.870555889	19.08922928	-1.110804473	0.321530607	0.472760864	-6.58976729
RAVR1	-7.886050222	19.30615356	-1.097827758	0.326606794	0.474639437	-6.603469353
SPSY	-7.895845278	19.25749153	-1.102002713	0.324966009	0.474639437	-6.599071179
TBCB	-7.898091444	19.03061017	-1.12035229	0.317840287	0.471234695	-6.579626929
EI2BA	-7.898540278	18.18409403	-1.18687931	0.293157968	0.460923885	-6.507644871
VAMP7	-7.908706889	11.73157711	-0.766186225	0.481157543	0.588503797	-6.916904857
CAH2	-7.914776333	19.01145906	-1.124955265	0.316074624	0.470256967	-6.574720696
RFC2	-7.949987611	18.76106653	-1.150036682	0.306605965	0.467680797	-6.547789543
MAP2	-7.953061111	18.852346	-1.143201306	0.309161025	0.467680797	-6.555161702
PURA	-7.987388167	11.47699436	-0.793902924	0.466365992	0.581797715	-6.893783725
SPC24	-7.991945722	19.31142203	-1.114715128	0.320014548	0.472680539	-6.585619921
RBM27	-7.9997285	18.85282603	-1.151989005	0.305879664	0.467602559	-6.54567947

LSM6	-8.010800333	11.73373317	-0.777168698	0.475255402	0.585192204	-6.907818502
ASF1A	-8.030675111	18.51661578	-1.185013212	0.293826099	0.460998859	-6.509694449
CSK22	-8.041665556	18.814102	-1.16276782	0.301897439	0.465602573	-6.533994576
H1X	-8.055456889	19.89446844	-1.086247137	0.331196152	0.474639437	-6.615618176
TXLNA	-8.065600333	18.51932772	-1.191274122	0.291589904	0.459802616	-6.502811247
DDX42	-8.074506444	19.13129678	-1.144040349	0.30884637	0.467680797	-6.554258075
2ABA	-8.085213389	19.43195008	-1.124092877	0.316404766	0.470256967	-6.575640766
DERL1	-8.085983167	18.71282931	-1.178159678	0.296291761	0.461761342	-6.5172072
SYAP1	-8.105862778	18.9750385	-1.162408725	0.302029354	0.465602573	-6.534384809
RN126	-8.112438056	19.28454575	-1.135464423	0.312075991	0.467680797	-6.563476776
NDUB8	-8.134895444	19.07174417	-1.160184066	0.302847741	0.465852369	-6.536800916
ACPM	-8.135242778	21.23387494	-1.014182193	0.361032119	0.486800785	-6.689464571
KDM1A	-8.135438222	18.55005678	-1.202094429	0.287761648	0.457841667	-6.49087084
SNW1	-8.136255667	18.59343778	-1.198083273	0.289175442	0.459063322	-6.495303775
CA123	-8.136462167	11.93586892	-0.775636927	0.476075361	0.585347561	-6.909091771
SF3B4	-8.154353333	20.35424967	-1.072021245	0.336910872	0.474639437	-6.630438047
CND3	-8.155961528	18.82155049	-1.183038385	0.294534661	0.460998859	-6.511861593
CHIP	-8.162247333	19.41177944	-1.139227667	0.310655096	0.467680797	-6.559436217
TIM8A	-8.180215889	19.51995517	-1.134419372	0.312471599	0.467680797	-6.564597515
ACSL4	-8.208667556	18.50311967	-1.215691571	0.283015893	0.454544056	-6.475787245
STK26	-8.272427667	19.0935835	-1.183358191	0.294419811	0.460998859	-6.511510774
NIBL1	-8.274453278	18.81037114	-1.205568779	0.286542152	0.457074423	-6.487024964
YMEL1	-8.297624889	18.51916567	-1.234804672	0.276466018	0.450266647	-6.454439516
KITH	-8.319629222	19.48562894	-1.161280102	0.302444292	0.465602573	-6.535610871
BZW1	-8.351365194	19.11659685	-1.191752982	0.291419508	0.459802616	-6.502284008
CSN4	-8.351422556	18.74033961	-1.225703717	0.279567276	0.452228705	-6.464625409
LSM3	-8.361929111	12.25331878	-0.776803258	0.475450927	0.585192204	-6.908122445
SMC4	-8.388133889	18.81270594	-1.227567642	0.278929531	0.451882796	-6.462542354
VMP1	-8.389788944	19.92584497	-1.136188938	0.311801986	0.467680797	-6.562699451
MRT4	-8.419016556	19.44439417	-1.182501594	0.294727528	0.460998859	-6.51245033
ASNA	-8.423683	19.19818817	-1.202120814	0.287752369	0.457841667	-6.490841654
GBG5	-8.450466444	4.225233222	-1.070037108	0.337714707	0.474639437	-6.632495798
EIF2A	-8.519013333	19.49360978	-1.195885073	0.289952909	0.459411139	-6.497729852
EIF3K	-8.542185778	19.81131322	-1.175777973	0.297152985	0.461761342	-6.519812571
PSB6	-8.546076667	20.74804767	-1.109376095	0.32208593	0.473010924	-6.591280047
SGTA	-8.554973444	19.91660006	-1.170071947	0.299225484	0.463625532	-6.526042985
RFA3	-8.560357944	18.96727136	-1.245772048	0.272770843	0.446657018	-6.442115062
GAR1	-8.561181889	11.72333961	-0.837907479	0.443589971	0.564617205	-6.855810695
PSMD8	-8.612025556	20.20970111	-1.156173809	0.304328029	0.466782272	-6.541149928
CKAP5	-8.643639056	19.22129369	-1.2394946	0.274880256	0.448732547	-6.449175857
NDUS5	-8.651241833	18.77133008	-1.281520192	0.261041435	0.435137091	-6.401578287
EDC4	-8.683612444	19.23167989	-1.247605211	0.27215767	0.446086232	-6.440049839
PIN1	-8.693333	18.94827861	-1.272769235	0.263868592	0.438108121	-6.411552057
RAD50	-8.713041556	18.98955589	-1.271557778	0.264262216	0.438108121	-6.41293025

PSMG2	-8.734267833	18.80555353	-1.293279181	0.257286992	0.431238889	-6.388125904
PEPD	-8.74344	19.858693	-1.207529305	0.285856089	0.457074423	-6.484852269
COA6	-8.779151	18.71744239	-1.310579916	0.251855036	0.425142468	-6.368231214
DNJC9	-8.871569111	19.32303678	-1.274022722	0.263461887	0.438108121	-6.410125393
PCKGM	-8.884356611	19.51341836	-2.741102526	0.044741801	0.133598768	-4.564969015
IFT25	-8.914725389	19.52318453	-1.265499236	0.266238951	0.439901022	-6.419813268
CNOT1	-8.9236385	4.46181925	-1.480851277	0.20391506	0.365333536	-6.16664913
HPBP1	-8.953845222	19.79191372	-1.251118134	0.270986193	0.445102129	-6.436088067
FKB1A	-8.971157889	20.92294483	-1.16795465	0.299997822	0.463810991	-6.528350728
TR112	-8.987162222	20.05213522	-1.235854736	0.276110239	0.450266647	-6.453261855
SRSF6	-9.053166222	19.77706411	-1.269360158	0.264977656	0.438612082	-6.415428742
RBM3	-9.065212	21.12926344	-1.168081432	0.299951525	0.463810991	-6.528212605
UCHL5	-9.1002405	19.68422608	-1.285920315	0.259630596	0.43346314	-6.396551206
DUS3L	-9.177546694	16.75637568	-1.535199861	0.190574435	0.348516331	-6.100419077
BZW2	-9.284819222	19.85999372	-1.305383201	0.25347524	0.426526608	-6.374219736
G3BP2	-9.327431667	19.7015375	-1.325516114	0.247252435	0.419031949	-6.350959917
RS30	-9.335281167	21.93213147	-1.157164115	0.303961879	0.466782272	-6.540076731
RBM26	-9.351604278	12.04294331	-1.074869903	0.335759702	0.474639437	-6.62747973
ML12A	-9.416896833	19.17453136	-1.392954866	0.227446982	0.395850584	-6.271938175
IMA5	-9.557733417	12.20997115	-1.083353444	0.332351686	0.474639437	-6.618642043
MVD1	-9.810660333	9.848640278	-1.413204885	0.221802744	0.388786163	-6.247898366
NUDT5	-9.957160722	12.35942075	-1.114279658	0.320183055	0.472680539	-6.586082162
FPPS	-10.01713244	20.49842067	-1.383845703	0.230030775	0.399044437	-6.282706907
HTSF1	-10.04394706	12.77686664	-1.220486024	0.281359641	0.453428044	-6.470448026
EMC10	-10.30032944	5.150164722	-1.784817503	0.139698354	0.271997161	-5.788027064
UBP15	-10.31797561	17.48533064	-1.697244259	0.155751198	0.297013913	-5.898883949
GLGB	-10.38631167	10.10881061	-1.221709238	0.280938504	0.453428044	-6.469084122
PAPS1	-10.53120717	12.76010636	-1.30308856	0.254193765	0.427398349	-6.376860568
DSRAD	-10.6824395	12.73759997	-1.357888944	0.237548431	0.407590852	-6.313233194
DDI2	-10.72116489	13.02116333	-1.288441765	0.258825341	0.432872676	-6.393666863
ZW10	-10.81092789	17.45053106	-1.834244865	0.131401836	0.259877656	-5.725021038
KAP2	-10.96304417	12.51689631	-1.451317885	0.211544782	0.376158027	-6.202292804
2A5G	-11.19965989	12.93974317	-1.388989174	0.228568398	0.397477834	-6.276629866
CCD50	-11.21870278	8.738509389	-1.532910365	0.191118616	0.348558503	-6.103224772
AKP8L	-11.24732106	8.805131028	-1.526156881	0.192732811	0.350303796	-6.111493161
PRC2C	-11.26564222	17.98260167	-1.847174199	0.129317298	0.25831135	-5.708496704
IPYR	-11.29099122	21.09686061	-3.390299817	0.022295333	0.081115443	-3.782896886
NU133	-11.64240561	16.41926614	-1.924799439	0.117508292	0.240862844	-5.60898069
RAB18	-11.90033589	16.95400328	-1.858302196	0.127550804	0.255581121	-5.694261662
RECQ1	-12.10056467	13.45558467	-1.66214442	0.16270439	0.308221234	-5.942980004
MUC18	-12.18883267	13.49649678	-1.678805463	0.159365333	0.302822541	-5.922075311
PDLI7	-12.20448439	13.49168653	-1.684947808	0.158151994	0.301322656	-5.914356192
PDLI5	-12.22416989	16.61104906	-2.060304234	0.099540258	0.214797398	-5.434418083
TIAR	-12.36212961	13.45019408	-1.751310567	0.145629404	0.281233595	-5.83057111

ADK	-12.43675489	14.79074433	-1.461550408	0.208870231	0.373346865	-6.189972319
ELP1	-12.55520839	16.04878464	-2.747689172	0.044411642	0.13336003	-4.556748142
NUBP2	-12.64393056	16.58696372	-2.170898143	0.08705195	0.201648054	-5.291656282
FR1OP	-12.93025661	16.94377792	-2.186720935	0.085407952	0.200538102	-5.271236684
PABP2	-12.99287811	14.39997239	-1.634661075	0.168368173	0.317383682	-5.977351834
UBCP1	-13.29939667	9.8116	-2.215846382	0.082469033	0.198895902	-5.233662781
MRE11	-13.33371456	14.9442445	-1.722790131	0.150881821	0.289280613	-5.866661919
HINT2	-14.08823256	14.80157606	-1.942822511	0.114932133	0.236749089	-5.585812091
NOL9	-14.22341767	14.85616572	-1.964947419	0.111850955	0.232423232	-5.557344825
PMM2	-14.27430078	14.38097656	-2.107815816	0.093953992	0.207799203	-5.37309018
PP1R7	-14.31182222	14.42054994	-2.107780754	0.093957988	0.207799203	-5.373135444
PDXD1	-14.37479444	15.07958333	-1.943693465	0.11480916	0.236749089	-5.584691983
RNH2A	-14.44993433	14.51546772	-2.120717019	0.092495887	0.206277438	-5.356434584
UN13D	-14.45274294	12.20061797	-2.831897357	0.040423679	0.124542318	-4.45210986
CPSF3	-14.49447028	10.41244436	-2.996834669	0.033730976	0.108961275	-4.249804169
ADDA	-14.50974133	14.52614372	-2.135098957	0.090899075	0.205076008	-5.337867423
SHLB2	-14.69718117	14.61866486	-2.162967841	0.087888758	0.20257171	-5.301891908
AHNK	-14.79237189	14.68912239	-2.169448502	0.087204271	0.201648054	-5.293527268
CH033	-14.81367439	14.63463136	-2.190223549	0.085048589	0.200136416	-5.266717094
SRA1	-14.81370256	14.71065172	-2.169770463	0.087170416	0.201648054	-5.293111724
REEP6	-14.81824117	15.72792447	-1.902942312	0.120714323	0.245575321	-5.637049293
NDUAA	-14.82335989	14.94402306	-2.104821337	0.094295952	0.208266688	-5.376956025
UBF1	-14.82767789	14.56102267	-2.219628532	0.08209551	0.198444023	-5.228785038
MEMO1	-14.83449378	15.04448611	-2.080291672	0.097147981	0.21199539	-5.408621376
NEUL	-14.84024442	14.62207704	-2.204581367	0.083592526	0.200136416	-5.248193156
BAG3	-14.84760639	14.90950686	-2.120952568	0.092469492	0.206277438	-5.356130485
AGRA3	-14.86702922	15.08847206	-2.078125081	0.097404298	0.212337386	-5.411417952
ARMT1	-14.90918933	15.13002878	-2.075301578	0.097739418	0.2126331	-5.415062363
XPOT	-14.91102033	14.93575211	-2.13214183	0.091224961	0.205376575	-5.341685043
PRP31	-14.91963206	7.459816028	-2.140024965	0.090358993	0.204813629	-5.33150809
AP1G1	-14.92329361	14.54373447	-2.247413525	0.079407186	0.197776504	-5.192964291
CSK21	-14.92504244	15.39126144	-2.010354425	0.105798221	0.223107624	-5.498848262
ARP5L	-14.93124989	14.86094994	-2.160355172	0.088166348	0.202773089	-5.305264251
RNZ2	-15.03750733	14.83797817	-2.197266692	0.084330921	0.200136416	-5.257629731
NU153	-15.04523728	14.75600642	-2.22460464	0.081606869	0.198444023	-5.222368076
PRP4	-15.04619489	7.523097444	-2.13998227	0.090363659	0.204813629	-5.331563207
VATG1	-15.05512633	15.76929961	-1.953899434	0.113378465	0.234682416	-5.571563172
TTC27	-15.05879356	14.72548133	-2.237494659	0.080355734	0.198444023	-5.205749058
VATE1	-15.06455356	15.91185989	-1.923336731	0.11772002	0.240862844	-5.610860096
WDFY1	-15.0682155	7.53410775	-2.137507941	0.090634523	0.204912835	-5.334757479
NMD3	-15.11850422	14.76926589	-2.241786239	0.079943823	0.198186315	-5.200217096
FXR1	-15.13869183	15.05658292	-2.161962047	0.087995509	0.202598732	-5.303190142
TS101	-15.15504144	7.577520722	-2.139979622	0.090363948	0.204813629	-5.331566624
HBS1L	-15.161867	14.96177389	-2.1972302	0.084334623	0.200136416	-5.257676812

SPT5H	-15.17899561	14.85078964	-2.236705003	0.080431778	0.198444023	-5.206767011
RM15	-15.19692856	15.0138515	-2.192479318	0.084818018	0.200136416	-5.263806495
PPIL4	-15.20063211	14.87403356	-2.235845785	0.080514609	0.198444023	-5.207874657
EXOS9	-15.21167856	15.09239628	-2.174044399	0.086722346	0.20144574	-5.287595659
DNJC8	-15.24459056	15.29772939	-2.123310823	0.092205682	0.206277438	-5.353085939
CTND1	-15.26950889	14.97171678	-2.226501686	0.081421417	0.198444023	-5.219921917
UGPA	-15.27326489	15.45288211	-2.09069117	0.095927703	0.210659553	-5.395197273
CLCA	-15.27621411	15.41332739	-2.101884846	0.094632588	0.20864186	-5.38074697
STK24	-15.28869033	7.644345167	-2.140016054	0.090359967	0.204813629	-5.331519593
GDS1	-15.31456878	14.68049739	-2.332545589	0.071750346	0.187465365	-5.083396968
PHOCN	-15.32722361	15.06368997	-2.216116163	0.082442328	0.198895902	-5.233314838
TOM20	-15.33729667	15.87395478	-1.999961903	0.107152172	0.224629725	-5.512244107
DFFA	-15.35031689	15.03026878	-2.229138938	0.081164363	0.198444023	-5.216521467
ARF6	-15.354847	15.12499017	-2.205127551	0.083537672	0.200136416	-5.24748858
PPIL2	-15.36883772	15.06998169	-2.226612929	0.081410556	0.198444023	-5.219778477
CPSF2	-15.36902822	15.08659456	-2.220268607	0.082032479	0.198444023	-5.227959588
CSN1	-15.37634661	15.05608247	-2.223097021	0.081754579	0.198444023	-5.22431216
WDR82	-15.37833278	7.689166389	-2.138785344	0.090494577	0.204813629	-5.333108392
SNUT2	-15.39962756	15.33178411	-2.156892922	0.088535663	0.203403054	-5.309733324
ADPPT	-15.40734394	15.18305953	-2.198618379	0.084193942	0.200136416	-5.255885847
PSPC1	-15.41474811	15.23017772	-2.187949697	0.085281696	0.200463701	-5.269651128
RN114	-15.42847872	15.11268175	-2.230724607	0.081010232	0.198444023	-5.214477018
TIM8B	-15.44084744	15.22471372	-2.196453759	0.08441342	0.200136416	-5.258678559
RPB2	-15.55420072	15.08526742	-2.276362378	0.076707815	0.194923791	-5.155671219
RFA2	-15.55459261	15.18692803	-2.236735831	0.080428808	0.198444023	-5.206727269
TBG1	-15.65458622	15.34713956	-2.227379399	0.081335768	0.198444023	-5.218790178
PCY2	-15.69488056	15.32133917	-2.24674628	0.079470611	0.197776504	-5.193824224
TACO1	-15.69641239	15.27553769	-2.259692342	0.078249798	0.19671707	-5.177142389
UBE2O	-15.70718217	15.09565969	-2.317012136	0.073084965	0.18955857	-5.103363954
CAH8	-15.71236622	15.58883011	-2.173886722	0.086738832	0.20144574	-5.287799157
DNJC2	-15.73191033	15.36947817	-2.243229457	0.079805818	0.198186315	-5.198356884
CHD4	-15.78518489	15.464321	-2.230683999	0.081014175	0.198444023	-5.214529374
NUP58	-15.80867856	15.14233161	-2.336968656	0.071375221	0.187080646	-5.077713811
4EBP1	-15.81849511	16.59875789	-1.934032293	0.116181055	0.238860183	-5.597114396
PAK2	-15.83879922	15.16525911	-2.335313429	0.07151535	0.187080646	-5.079840471
EI2BB	-15.84816633	15.12787661	-2.35346825	0.069994728	0.185072737	-5.056523215
PLRG1	-15.85562683	14.95822136	-2.412680096	0.065276891	0.177513178	-4.980613573
DDX24	-15.86272844	15.34973533	-2.287588702	0.075688066	0.193794083	-5.141217972
BCCIP	-15.929192	15.59794633	-2.224563067	0.081610939	0.198444023	-5.222421684
RM45	-15.93589367	16.19915039	-2.071585017	0.098182418	0.213307463	-5.419859316
IF4H	-15.93845089	16.09310156	-2.09798984	0.095081101	0.209318435	-5.385775264
MAGC1	-15.95644122	15.66479006	-2.219894849	0.082069278	0.198444023	-5.228441591
LYPA2	-15.99835478	15.50202094	-2.279863594	0.07638818	0.194343198	-5.151163034
E41L2	-16.01444311	15.323879	-2.341947938	0.070955493	0.186419365	-5.071317258

CLCB	-16.01723339	15.45869142	-2.299495836	0.074622627	0.192609492	-5.125894249
RUXGL	-16.03038589	16.21965983	-2.090560135	0.095942976	0.210659553	-5.395366425
SMC2	-16.04596728	15.18792631	-2.342555716	0.070904446	0.186419365	-5.070536579
PDXK	-16.06910056	15.59711417	-2.270251261	0.077269214	0.195590625	-5.163541129
UFD1	-16.07414689	15.92317044	-2.180361414	0.086064638	0.201194964	-5.279443257
COX6C	-16.08469689	16.03781544	-2.152477448	0.089009083	0.203831756	-5.315433001
NDUA3	-16.08508978	15.88542178	-2.194414412	0.084620764	0.200136416	-5.261309741
CLU	-16.17964011	15.37936283	-2.374334499	0.068290687	0.183139301	-5.02974725
PSMG3	-16.18863733	16.122745	-2.154353952	0.088807554	0.203808079	-5.313010708
SRPK1	-16.20900056	15.23657961	-2.431185483	0.063875119	0.175200322	-4.956937992
XPO5	-16.28402594	15.52091403	-2.361238107	0.069354806	0.184140033	-5.046549731
ZC3HF	-16.32584144	15.73464928	-2.300551596	0.074528952	0.19260088	-5.124535865
LSM7	-16.48946078	15.71459772	-2.352760613	0.07005333	0.185072737	-5.057431723
CIRBP	-16.52468589	15.43670317	-2.462274033	0.061594385	0.170941649	-4.917220763
ELOB	-16.52630056	15.71765972	-2.3717432	0.068499805	0.183469328	-5.033071015
EIF3H	-16.58083089	15.395805	-2.496382879	0.059195163	0.166496157	-4.873732849
SNX3	-16.61656678	16.18334694	-2.257548014	0.078450589	0.196772536	-5.179905078
RL29	-16.67676483	17.53718964	-1.931846894	0.116493776	0.239272379	-5.599923612
RL31	-16.71811389	16.48646061	-2.196870325	0.084371135	0.200136416	-5.258141113
EI2BG	-16.93017311	15.54717422	-2.557682726	0.055139497	0.158007269	-4.795828996
OGFR	-16.94286672	13.36647886	-3.70067912	0.016365893	0.064734849	-3.431361789
PSMD7	-17.00671806	15.68241886	-2.532391654	0.05677414	0.161389954	-4.827929604
ATG4B	-17.03699939	13.39759092	-3.734223406	0.015842286	0.063607821	-3.39427873
PRC2A	-17.09291511	11.69558667	-4.397991605	0.008613863	0.042609644	-2.696668601
U2AF1	-17.10014489	15.97660411	-2.45888148	0.061838842	0.170999237	-4.921551314
NUDC1	-17.24734622	13.58173656	-3.711915366	0.016188291	0.064454559	-3.418920364
BRE1A	-17.69606594	13.74307847	-3.86701151	0.01395226	0.058903401	-3.249225918
GID8	-17.80222622	8.901113111	-4.626852361	0.007081482	0.037690357	-2.471664345
UN45A	-18.09382322	13.93595894	-3.958053425	0.012807969	0.055614234	-3.151379631
ACACA	-18.17990194	13.75014136	-4.170265188	0.010541321	0.048435551	-2.928330686
JMJD6	-18.44201644	14.09649333	-4.045965145	0.011805836	0.052547061	-3.058129386
TACC3	-18.62909933	14.23584789	-4.046496662	0.011800062	0.052547061	-3.057569259
CSN5	-18.68663944	9.343319722	-4.32297139	0.009199101	0.044472753	-2.772117382
EXOS2	-18.79456894	12.57378503	-6.299337362	0.00203268	0.018519974	-1.038968111
SPTB2	-19.78085356	13.05071822	-6.646919991	0.001622535	0.016550457	-0.7817262
SF3A2	-20.04019494	13.36684453	-6.395407135	0.001907989	0.01799926	-0.966633382
RIR2	-20.35138856	13.35909883	-6.836214106	0.001441071	0.015516992	-0.646673959
RPA34	-21.86135722	10.93067861	-100.3517229	7.82E-09	1.39E-06	7.328249652
EXOSX	-22.20782711	11.10391356	-133.4622139	2.12E-09	4.11E-07	7.432123963
CD11A	-22.22483089	11.11241544	-58.4265048	9.30E-08	1.17E-05	6.90504049
PI4KA	-22.43498633	11.21749317	-65.55595963	5.49E-08	7.31E-06	7.029901722
PRPK	-22.64606944	11.32303472	-171.1676124	6.78E-10	1.62E-07	7.486640537
TCRG1	-22.69759433	11.34879717	-186.8981063	4.53E-10	1.61E-07	7.500442548
CAB39	-22.81280033	11.40640017	-211.1350929	2.59E-10	1.38E-07	7.516062163

RPAC1	-22.97195944	11.48597972	-212.6184288	2.51E-10	1.38E-07	7.516850884
GRWD1	-23.37428944	11.68714472	-215.6776761	2.35E-10	1.38E-07	7.518427137
MIC13	-23.393328	11.696664	-32.37527082	1.38E-06	0.000147233	5.813773027
PA1B2	-23.50192833	11.75096417	-66.87856031	5.01E-08	7.12E-06	7.049287609
TTC4	-23.69087733	11.84543867	-76.85983703	2.65E-08	4.35E-06	7.167716955

Table S4. Guide RNA（gRNA) and primers.

	**gRNA used for establishment of PI4KA knockout cells**	
PI4KA gRNA-1	ATGTCTAAGAAAACCAACCG	
PI4KA gRNA-2	ATAGAAGCCCCGCGAGGCGC	
	**gRNA used for generation of PI4KA-overexpressing Cells**	
PI4KA gRNA-1	GCGGTTGCGCGCGCCGGATG	
PI4KA gRNA-2	GGAGAGAGGGGATGGACAGT	
	**Primers used for qRT-PCR**	
Gene	(Forward)	(Reverse)
PI4KA	5’-CAGCTCTGACCAAGTGGAGAT-3’	5’-GCGGATGGTTGCATTTGGAA-3’
GAPDH	5’-CCACCCATGGCAAATTCCATGGCA-3’	5’-TCTAGACGGCAGGTCAGGTCCACC-3’
